# Supplementary figures and images for: Plectin-mediated cytoskeletal crosstalk as a target for inhibition of hepatocellular carcinoma growth and metastasis (part 1 of 2)
Source: eLife. 2025 Mar 7;13:RP102205. doi: 10.7554/eLife.102205 (PMC11893104; doi:10.7554/eLife.102205)

vimentin

GAPDH

E-cad

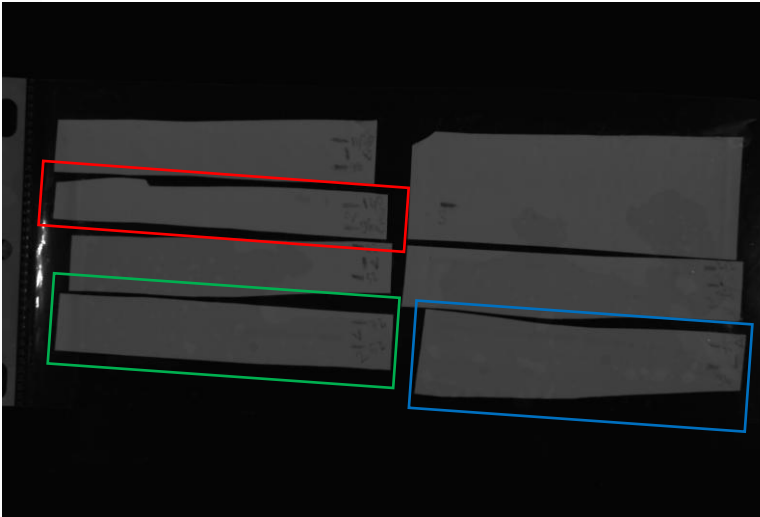

plectin

Huh7 HepG2 Hep3B PLL SNU-182 SNU-398 SNU-423 SNU-475 SK-Hep1

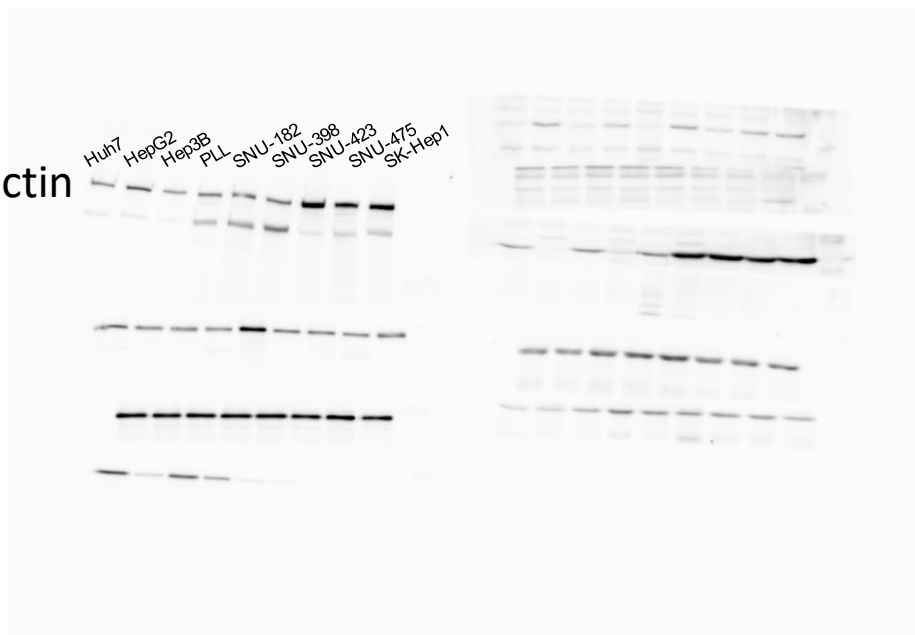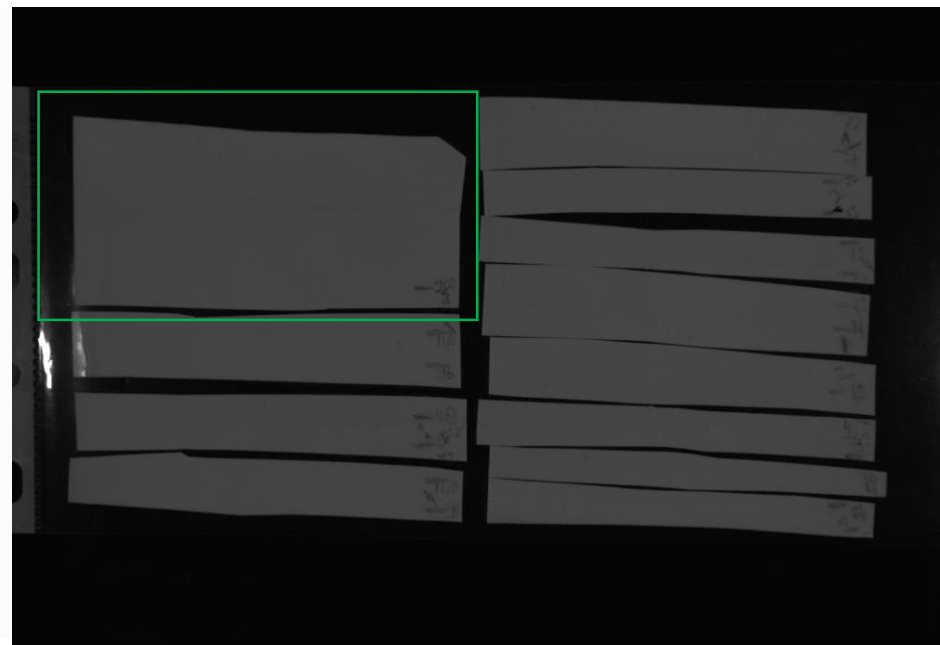

Supplement: Figure 1—source data 1. [file elife-102205-fig1-data1.pdf]

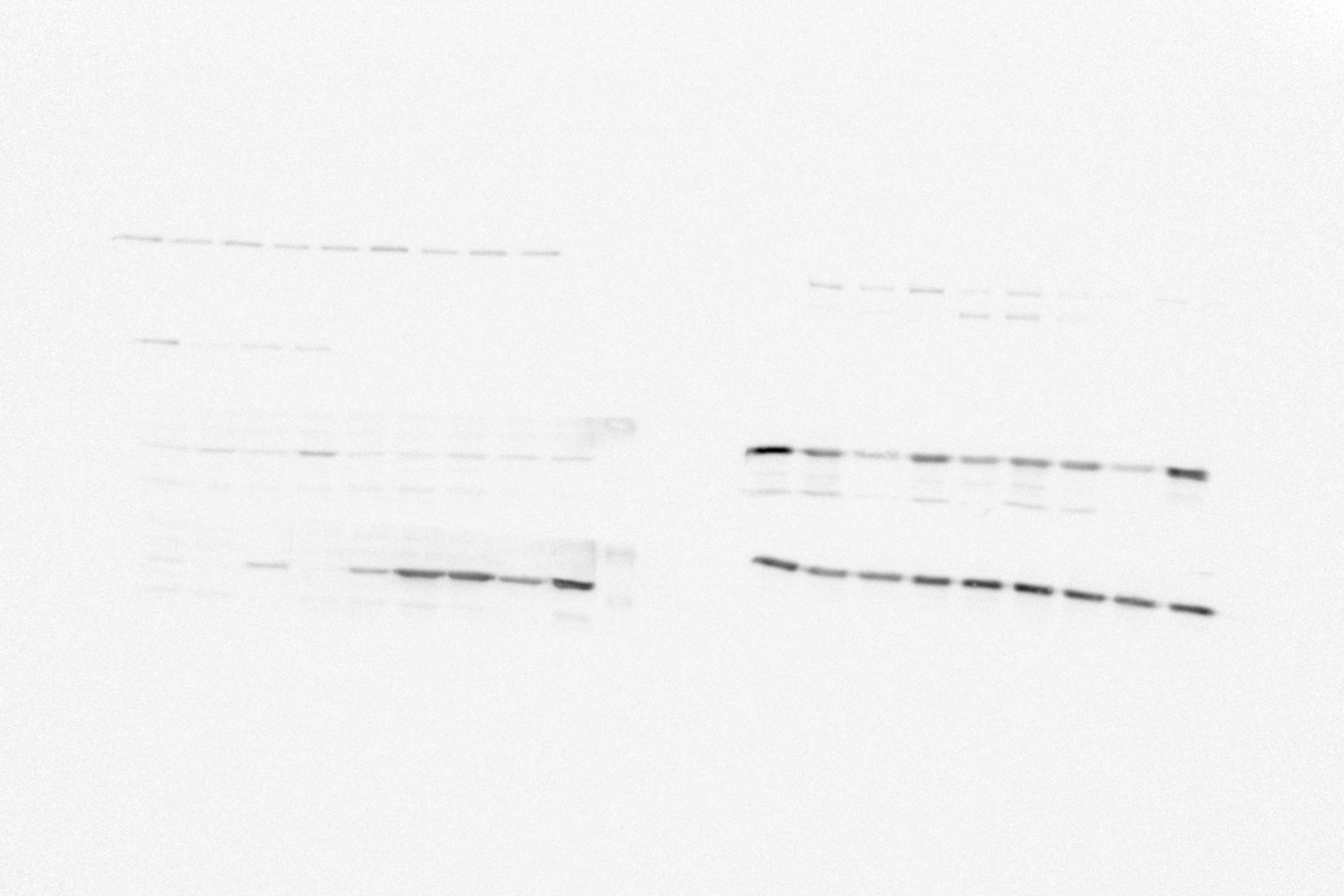

Supplement: Figure 1—source data 2. [file elife-102205-fig1-data2.zip › Figure 1-source data 2. Original files for western blot analysis displayed in Figure 1D/F1D E-cad vimentin GAPDH raw images/160617_HCC-01-1s.tif]

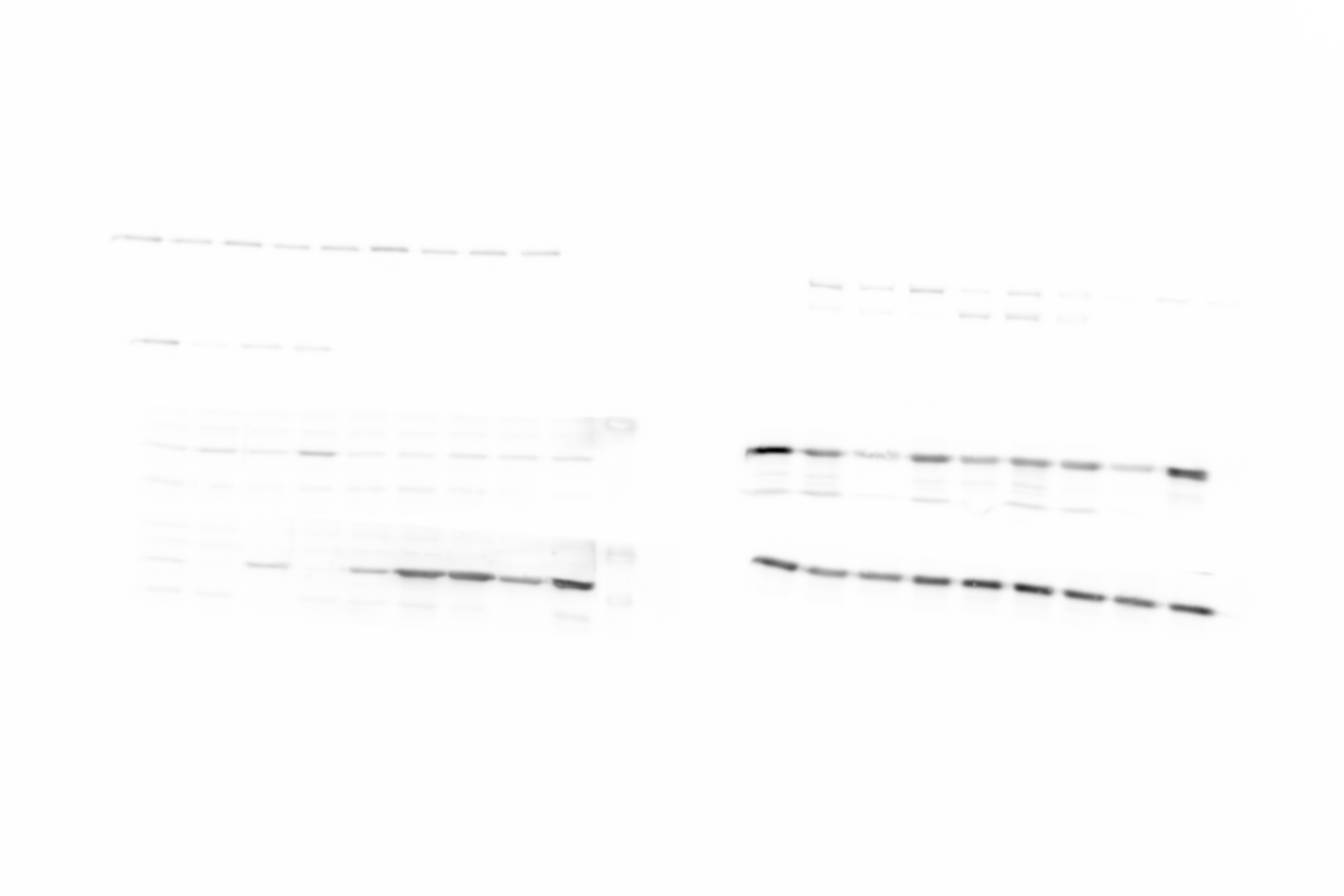

Supplement: Figure 1—source data 2. [file elife-102205-fig1-data2.zip › Figure 1-source data 2. Original files for western blot analysis displayed in Figure 1D/F1D E-cad vimentin GAPDH raw images/160617_HCC-02-10s.tif]

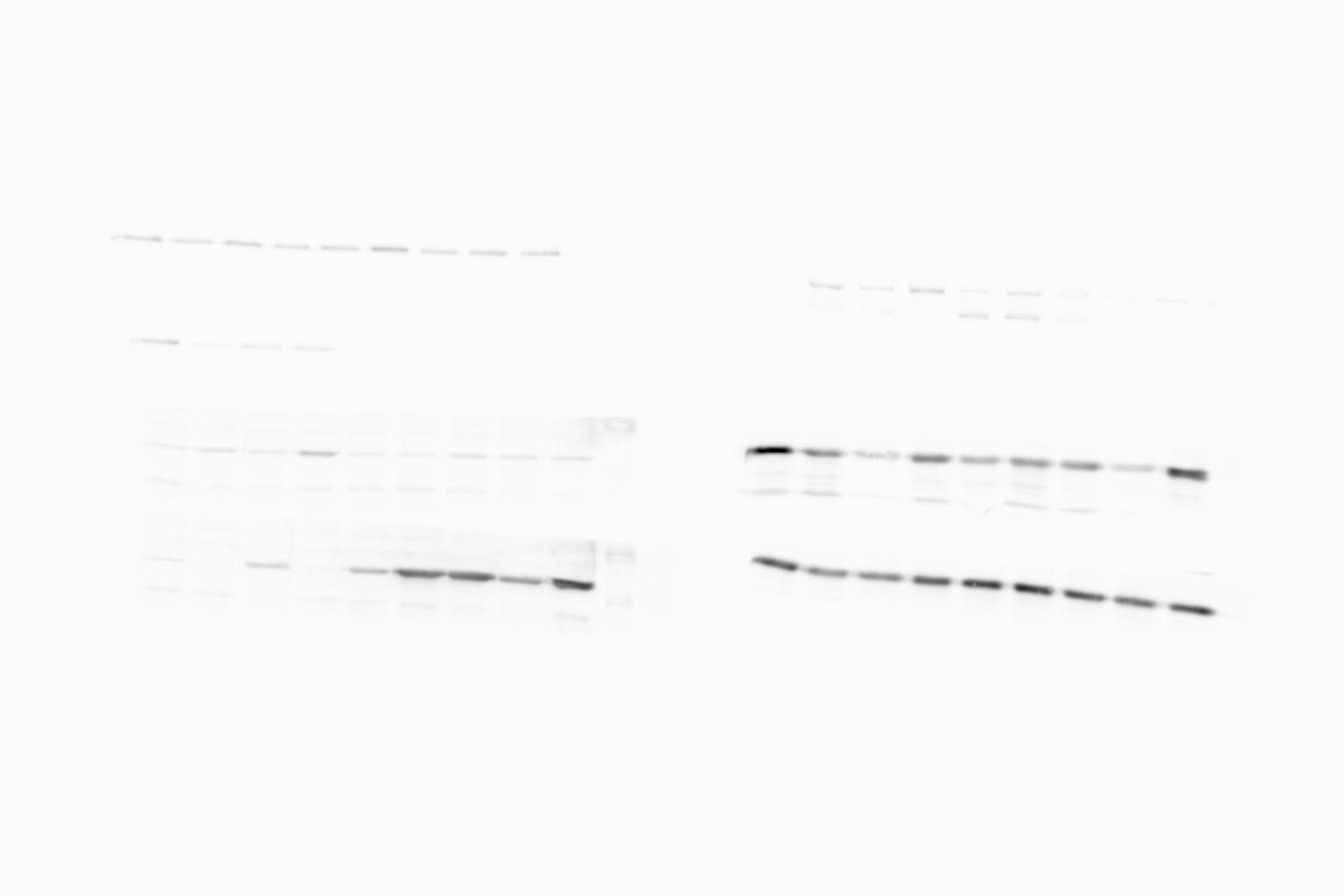

Supplement: Figure 1—source data 2. [file elife-102205-fig1-data2.zip › Figure 1-source data 2. Original files for western blot analysis displayed in Figure 1D/F1D E-cad vimentin GAPDH raw images/160617_HCC-03-30s.tif]

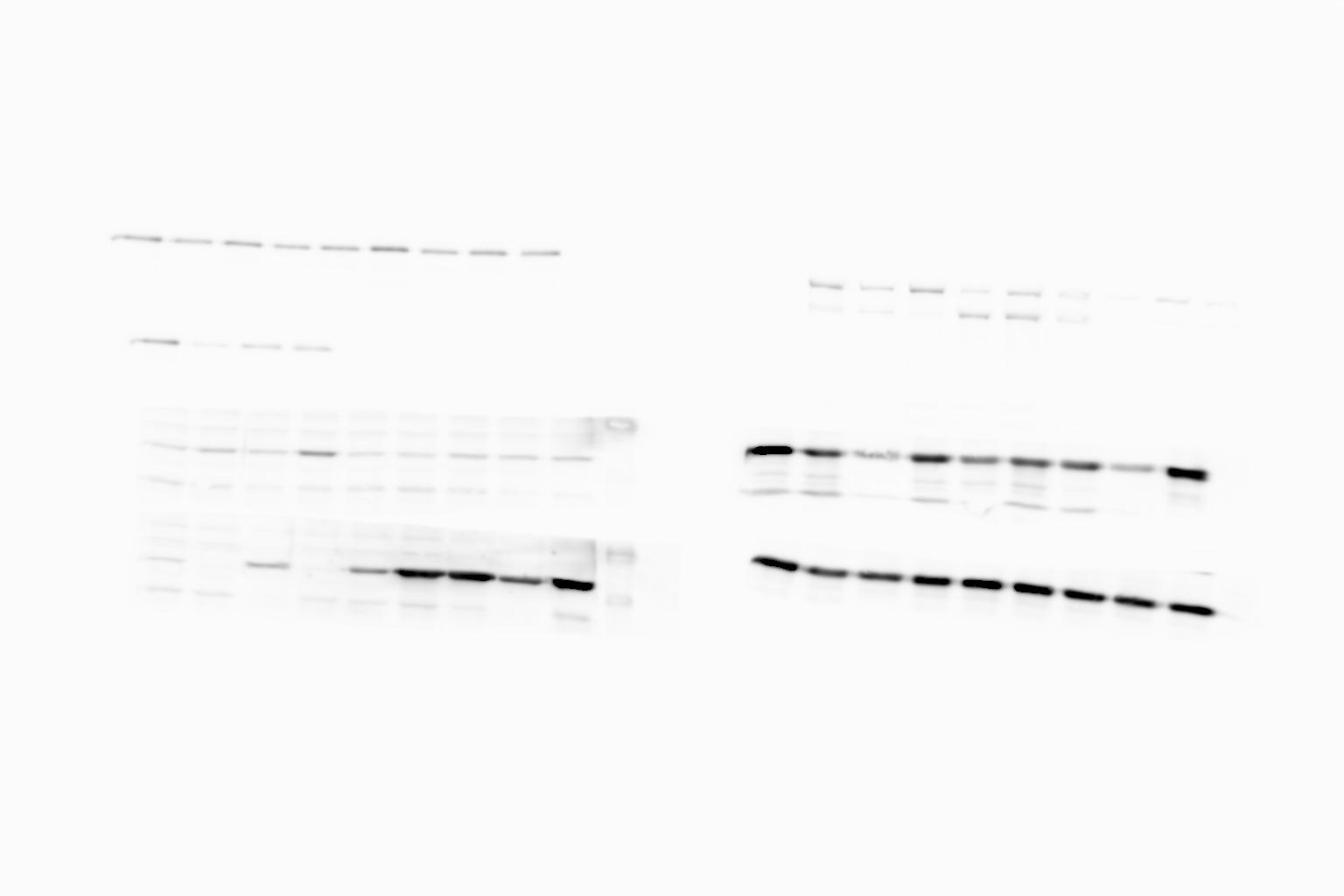

Supplement: Figure 1—source data 2. [file elife-102205-fig1-data2.zip › Figure 1-source data 2. Original files for western blot analysis displayed in Figure 1D/F1D E-cad vimentin GAPDH raw images/160617_HCC-04-1m.tif]

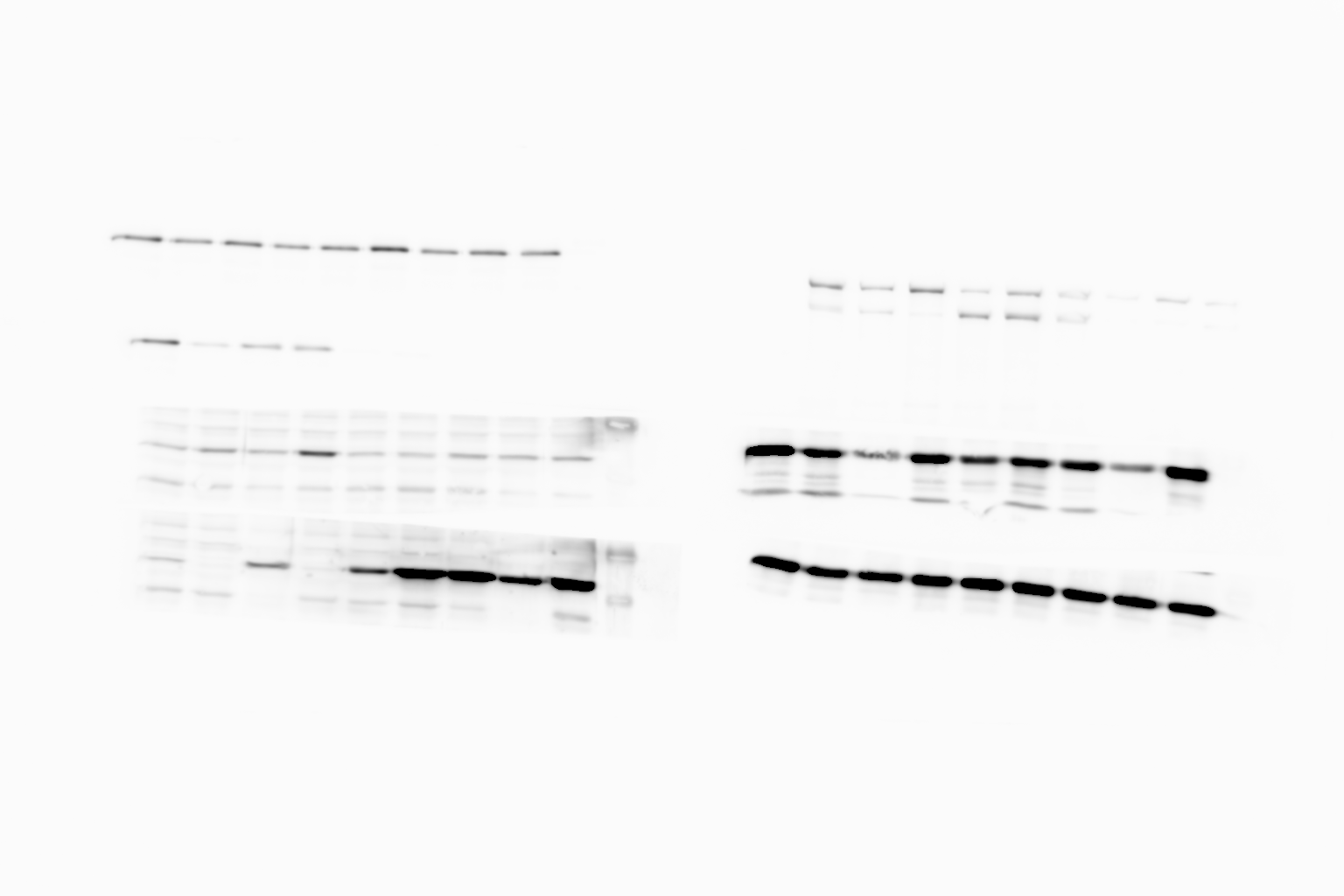

Supplement: Figure 1—source data 2. [file elife-102205-fig1-data2.zip › Figure 1-source data 2. Original files for western blot analysis displayed in Figure 1D/F1D E-cad vimentin GAPDH raw images/160617_HCC-05-2m.tif]

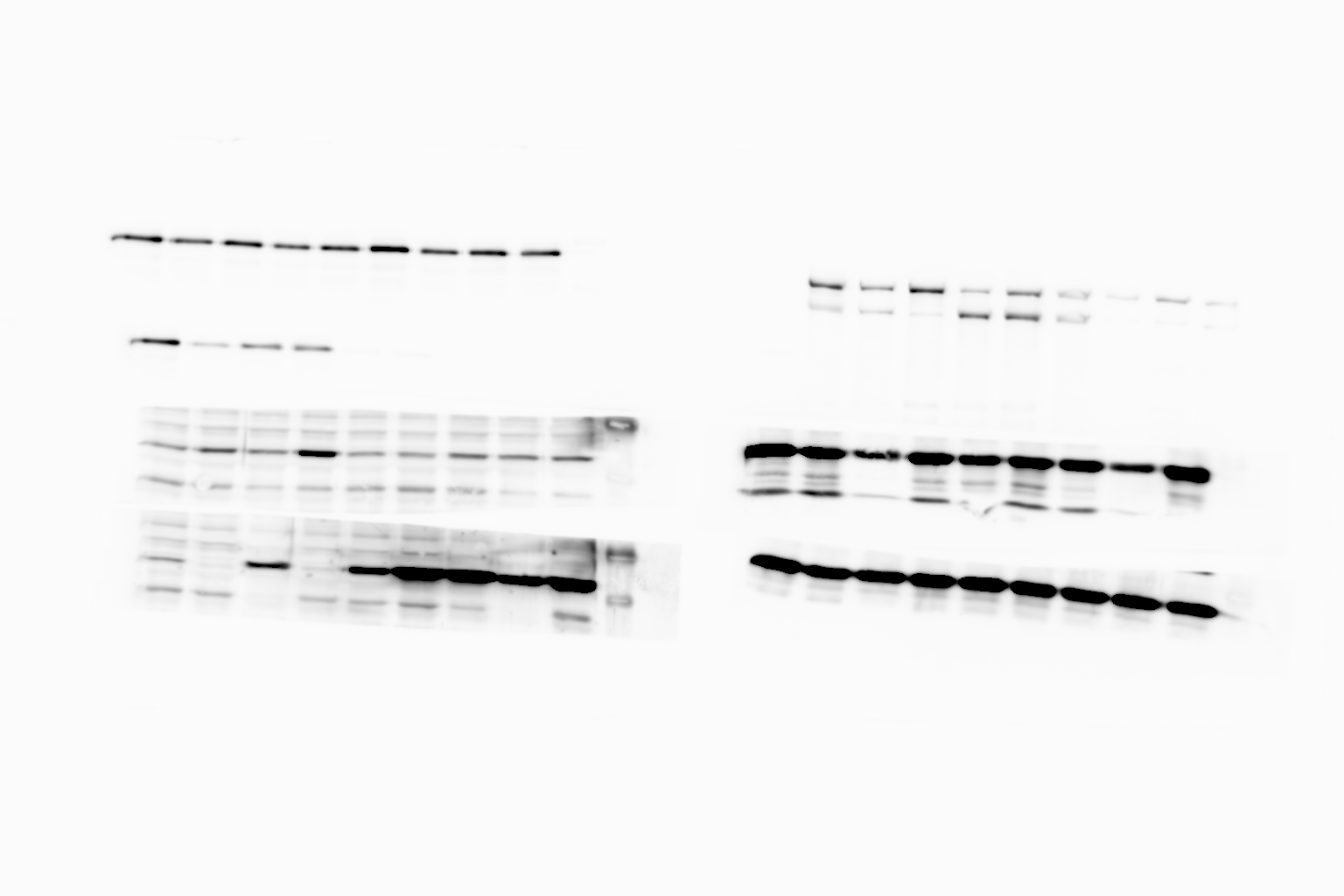

Supplement: Figure 1—source data 2. [file elife-102205-fig1-data2.zip › Figure 1-source data 2. Original files for western blot analysis displayed in Figure 1D/F1D E-cad vimentin GAPDH raw images/160617_HCC-06-5m.tif]

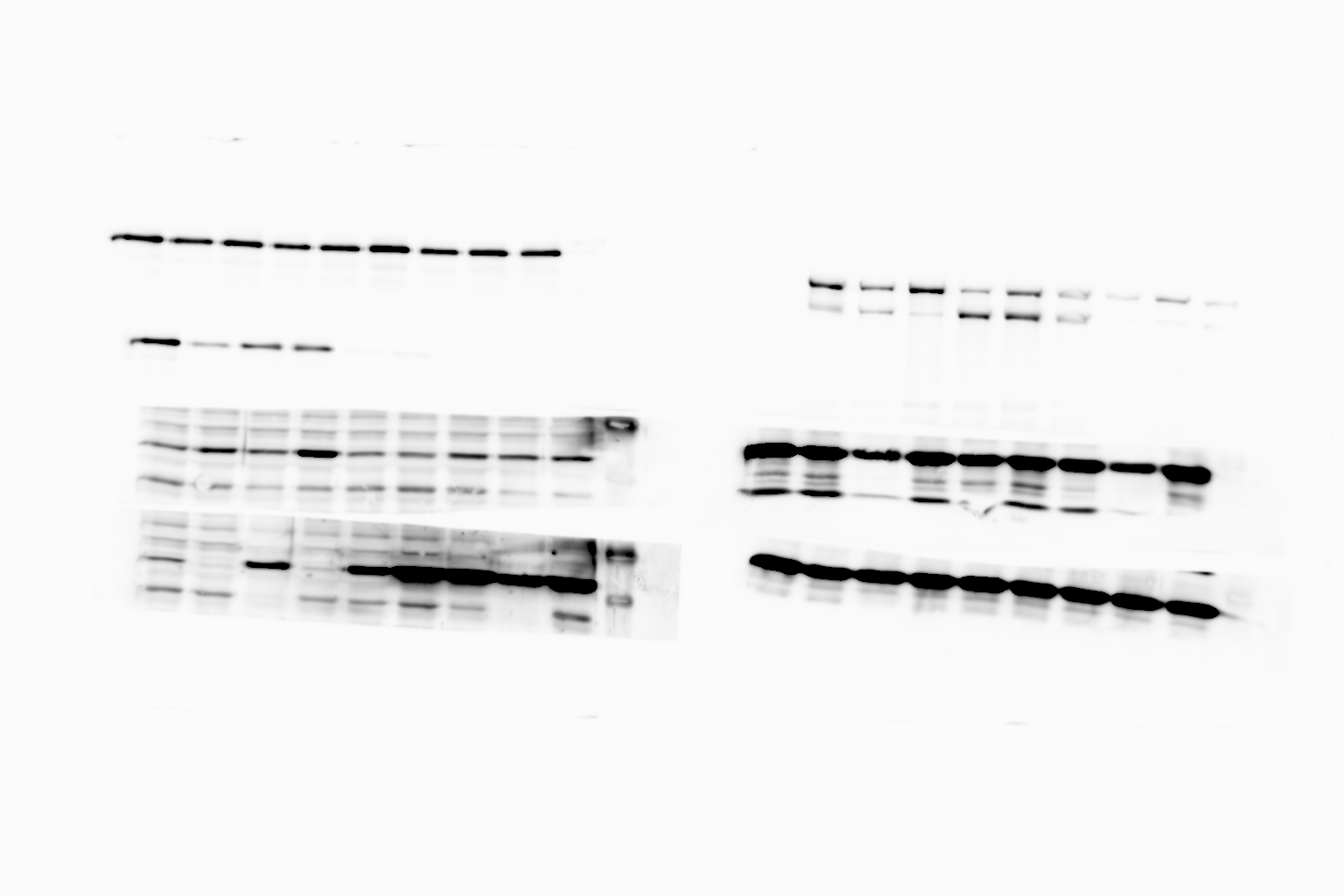

Supplement: Figure 1—source data 2. [file elife-102205-fig1-data2.zip › Figure 1-source data 2. Original files for western blot analysis displayed in Figure 1D/F1D E-cad vimentin GAPDH raw images/160617_HCC-07-10m.tif]

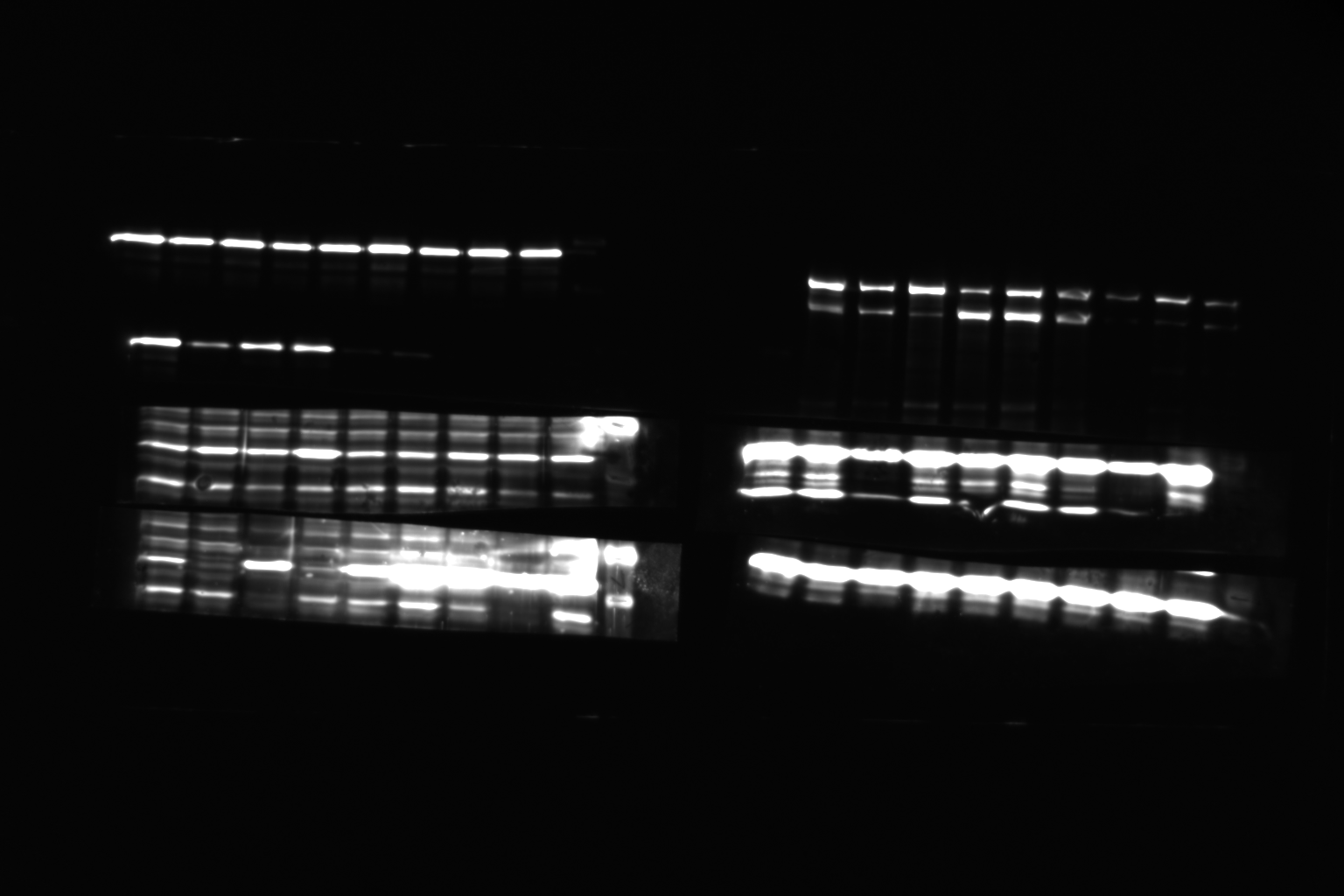

Supplement: Figure 1—source data 2. [file elife-102205-fig1-data2.zip › Figure 1-source data 2. Original files for western blot analysis displayed in Figure 1D/F1D E-cad vimentin GAPDH raw images/160617_HCC-SUM-1121s.tif]

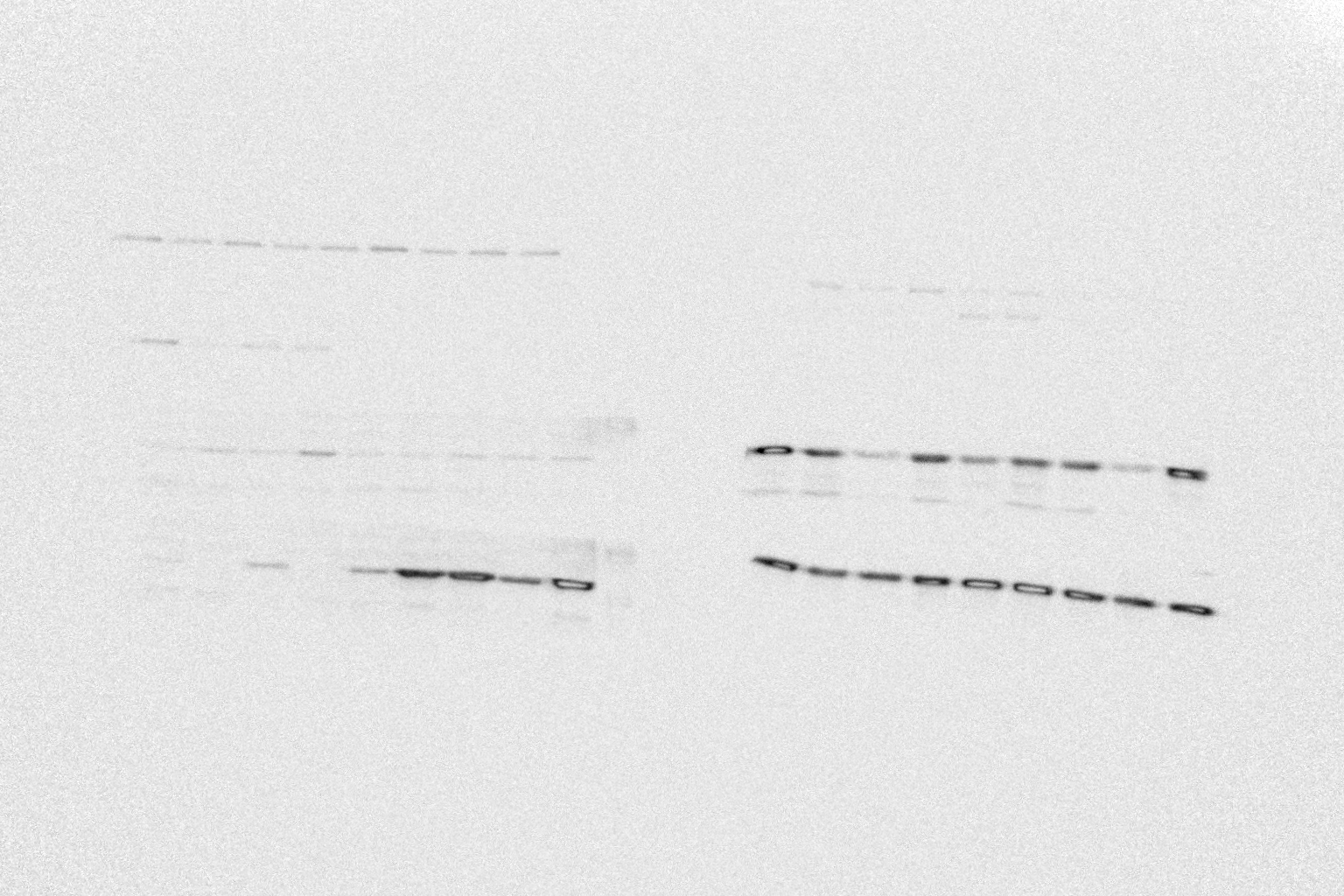

Supplement: Figure 1—source data 2. [file elife-102205-fig1-data2.zip › Figure 1-source data 2. Original files for western blot analysis displayed in Figure 1D/F1D E-cad vimentin GAPDH raw images/fdfdf.tif]

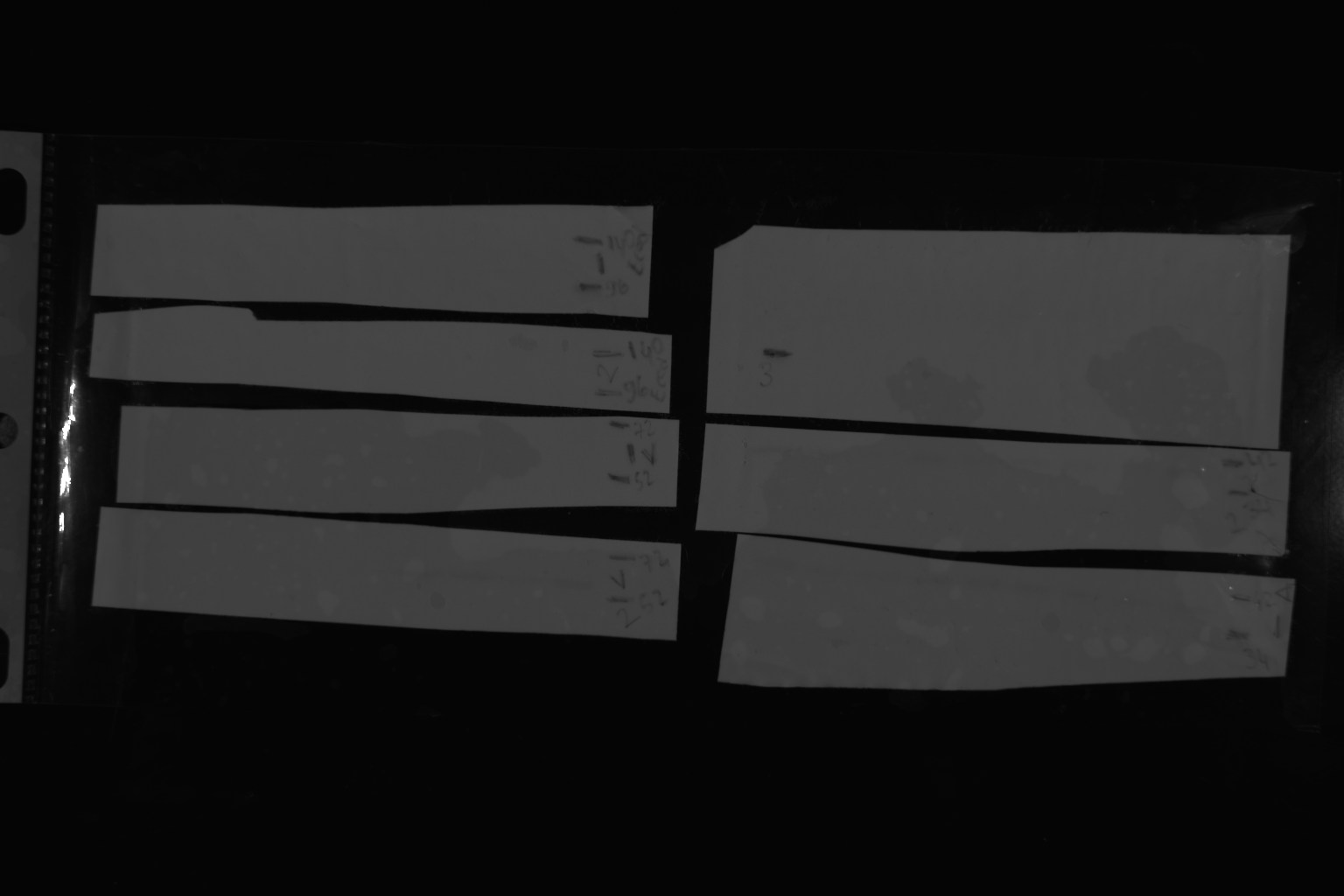

Supplement: Figure 1—source data 2. [file elife-102205-fig1-data2.zip › Figure 1-source data 2. Original files for western blot analysis displayed in Figure 1D/F1D E-cad vimentin GAPDH raw images/New Folder/V_fdfdf.tif]

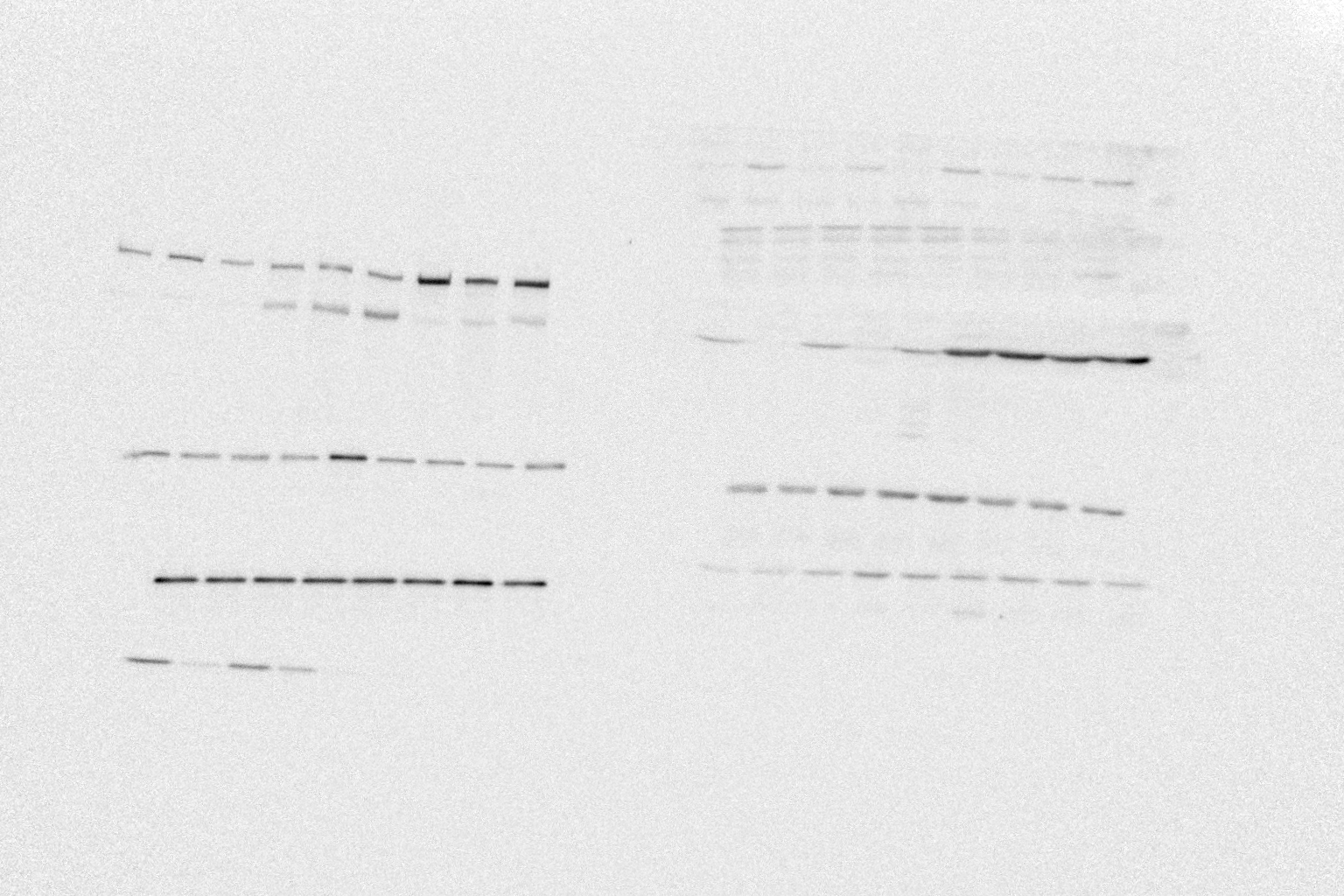

Supplement: Figure 1—source data 2. [file elife-102205-fig1-data2.zip › Figure 1-source data 2. Original files for western blot analysis displayed in Figure 1D/F1D plectin/160610_HCC-01-1s.tif]

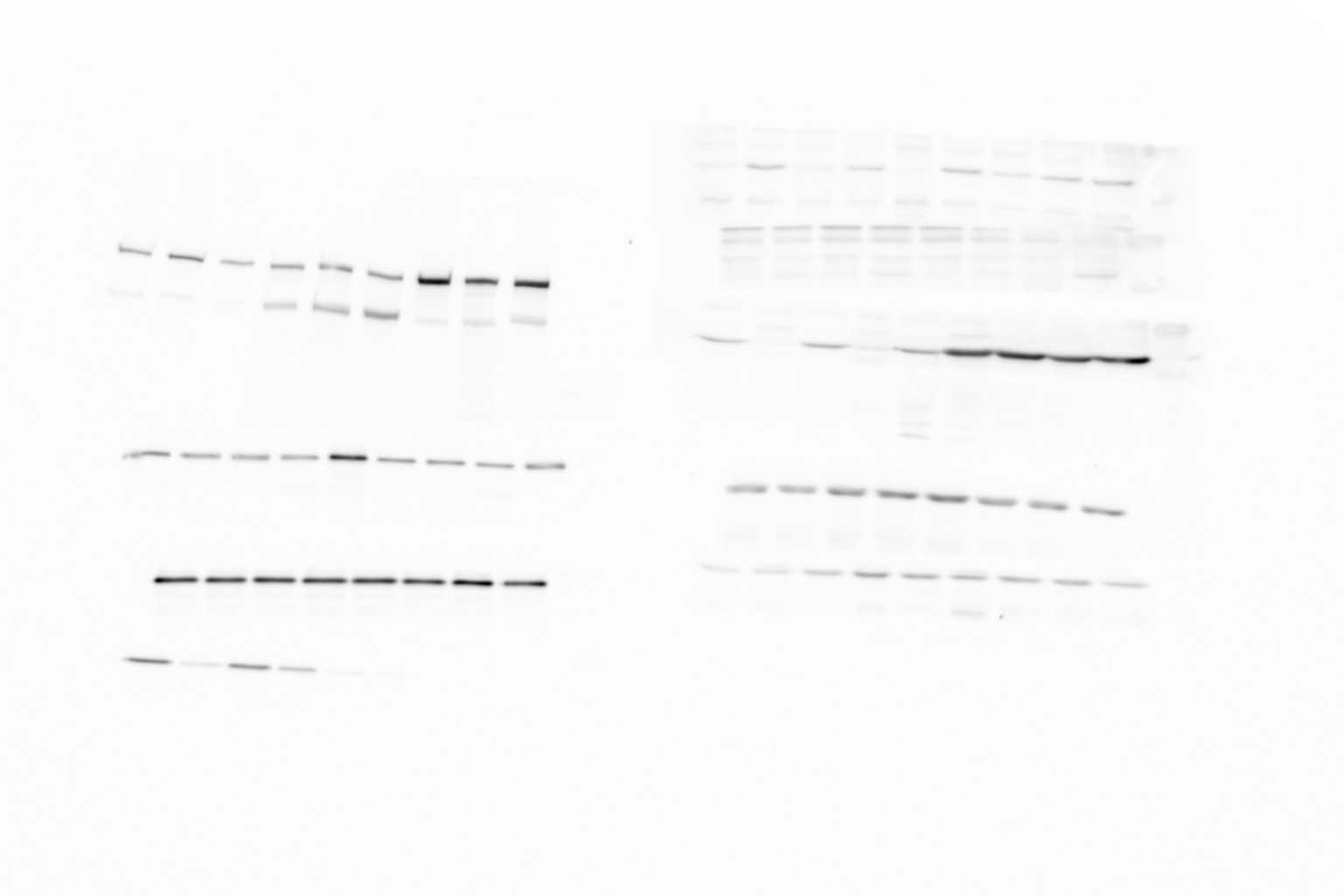

Supplement: Figure 1—source data 2. [file elife-102205-fig1-data2.zip › Figure 1-source data 2. Original files for western blot analysis displayed in Figure 1D/F1D plectin/160610_HCC-02-10s.tif]

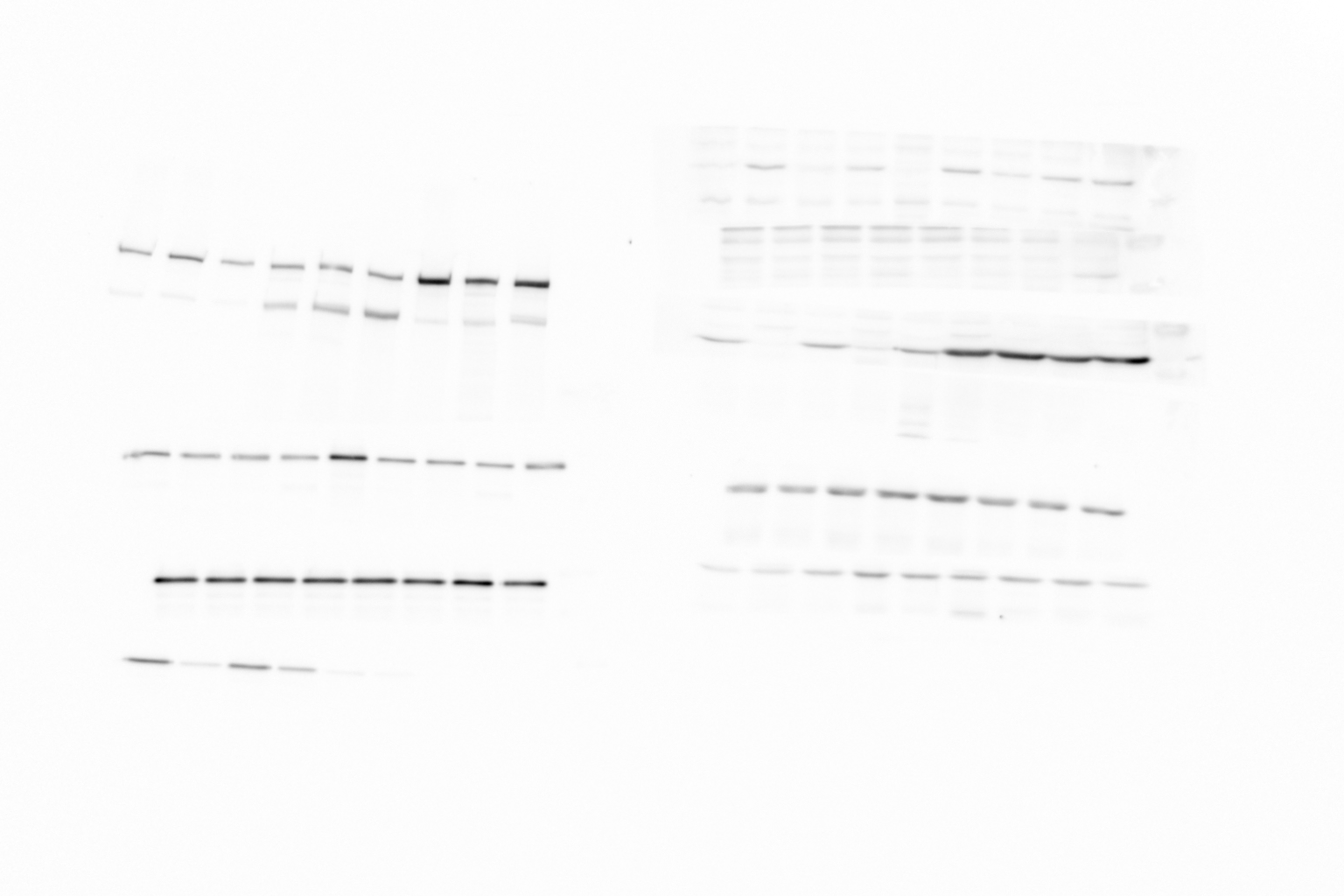

Supplement: Figure 1—source data 2. [file elife-102205-fig1-data2.zip › Figure 1-source data 2. Original files for western blot analysis displayed in Figure 1D/F1D plectin/160610_HCC-03-10s.tif]

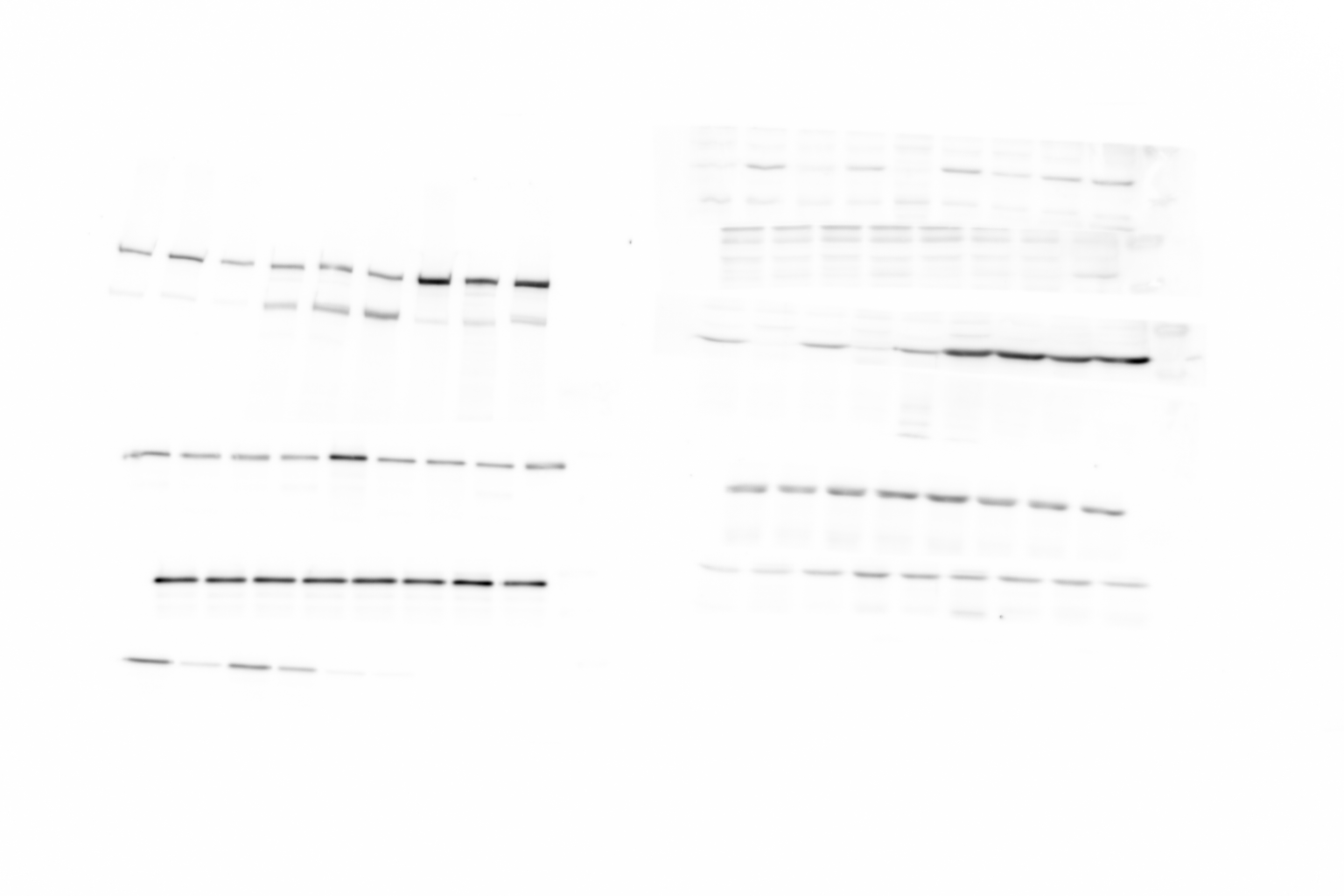

Supplement: Figure 1—source data 2. [file elife-102205-fig1-data2.zip › Figure 1-source data 2. Original files for western blot analysis displayed in Figure 1D/F1D plectin/160610_HCC-04-30s.tif]

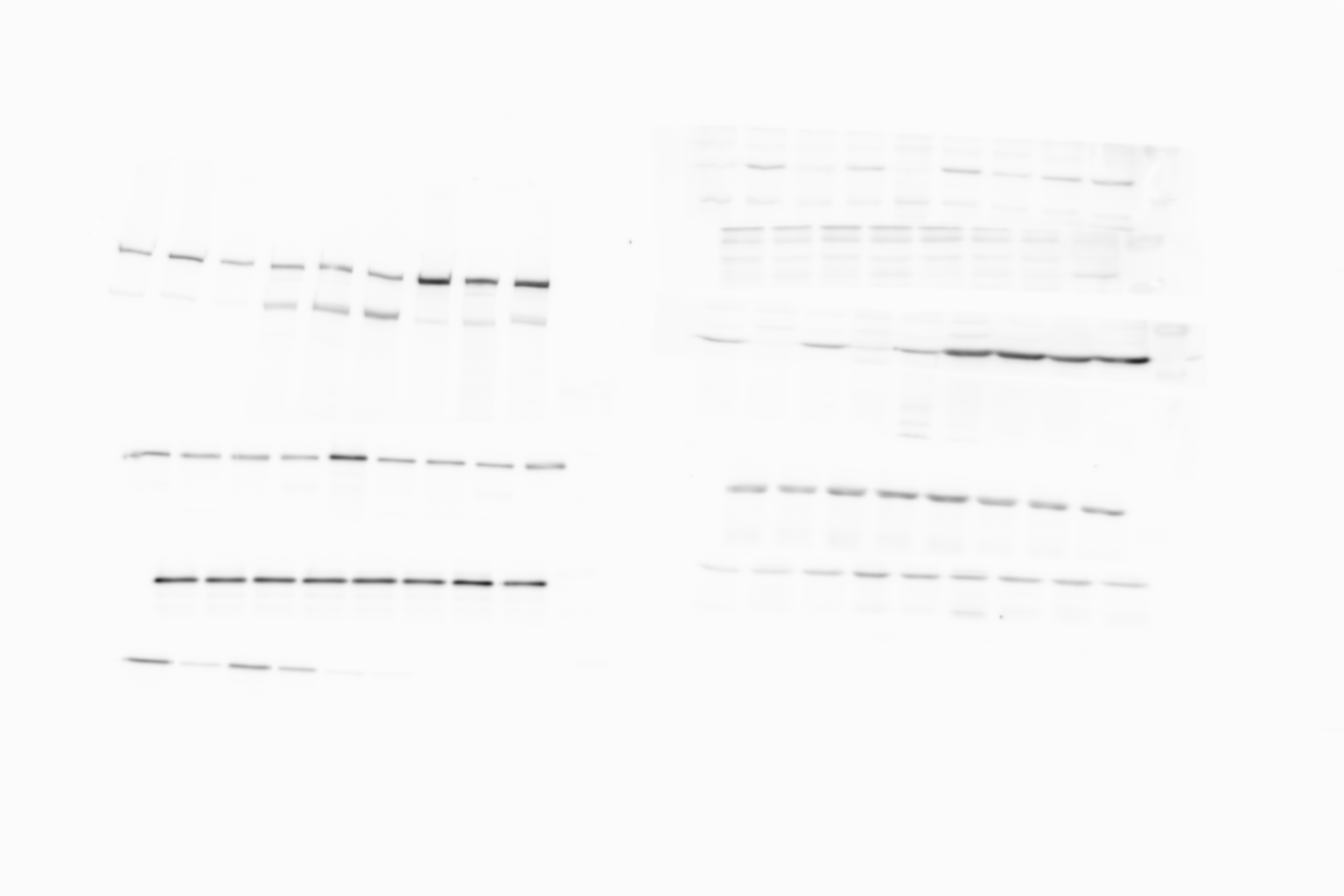

Supplement: Figure 1—source data 2. [file elife-102205-fig1-data2.zip › Figure 1-source data 2. Original files for western blot analysis displayed in Figure 1D/F1D plectin/160610_HCC-05-1m.tif]

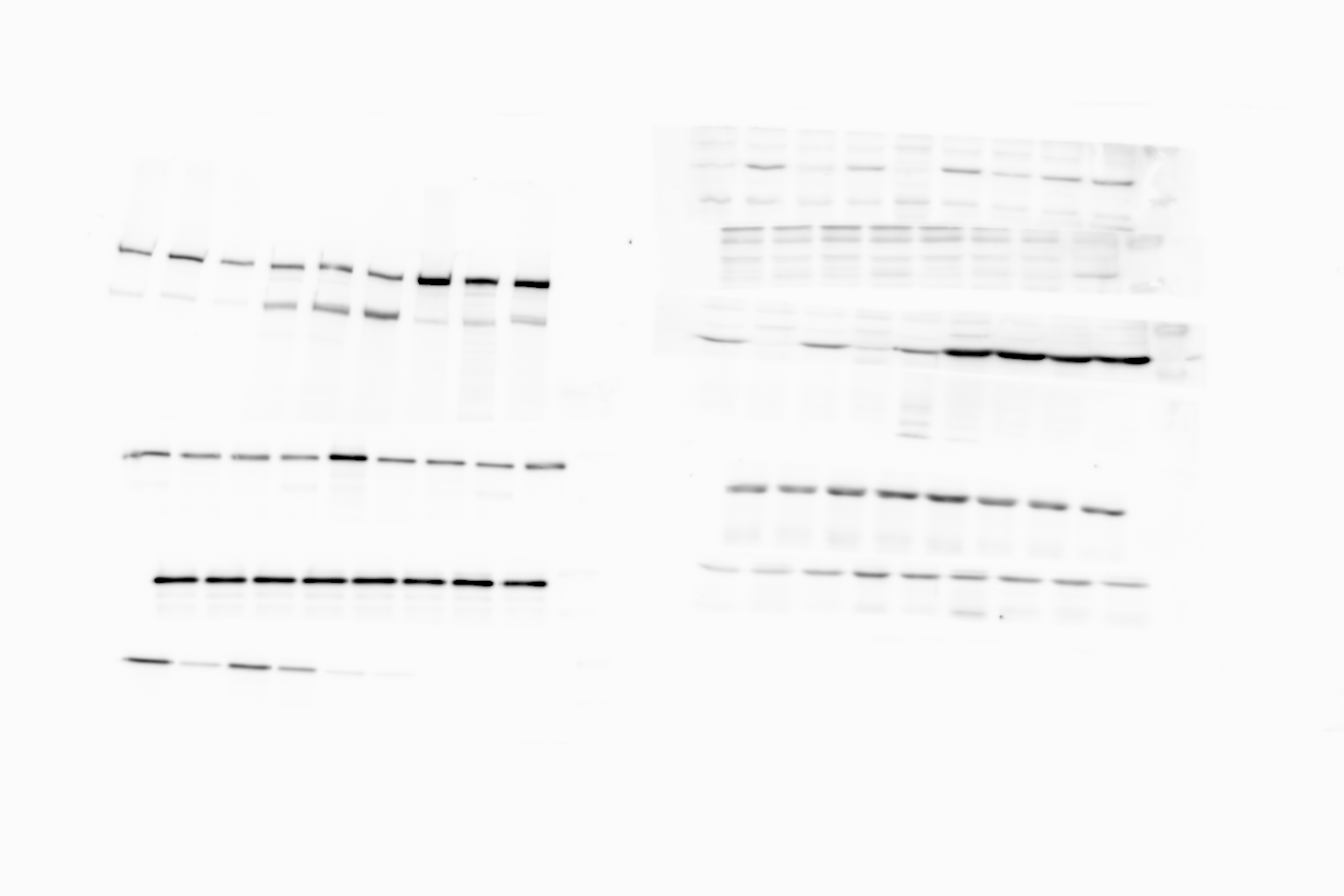

Supplement: Figure 1—source data 2. [file elife-102205-fig1-data2.zip › Figure 1-source data 2. Original files for western blot analysis displayed in Figure 1D/F1D plectin/160610_HCC-06-2m.tif]

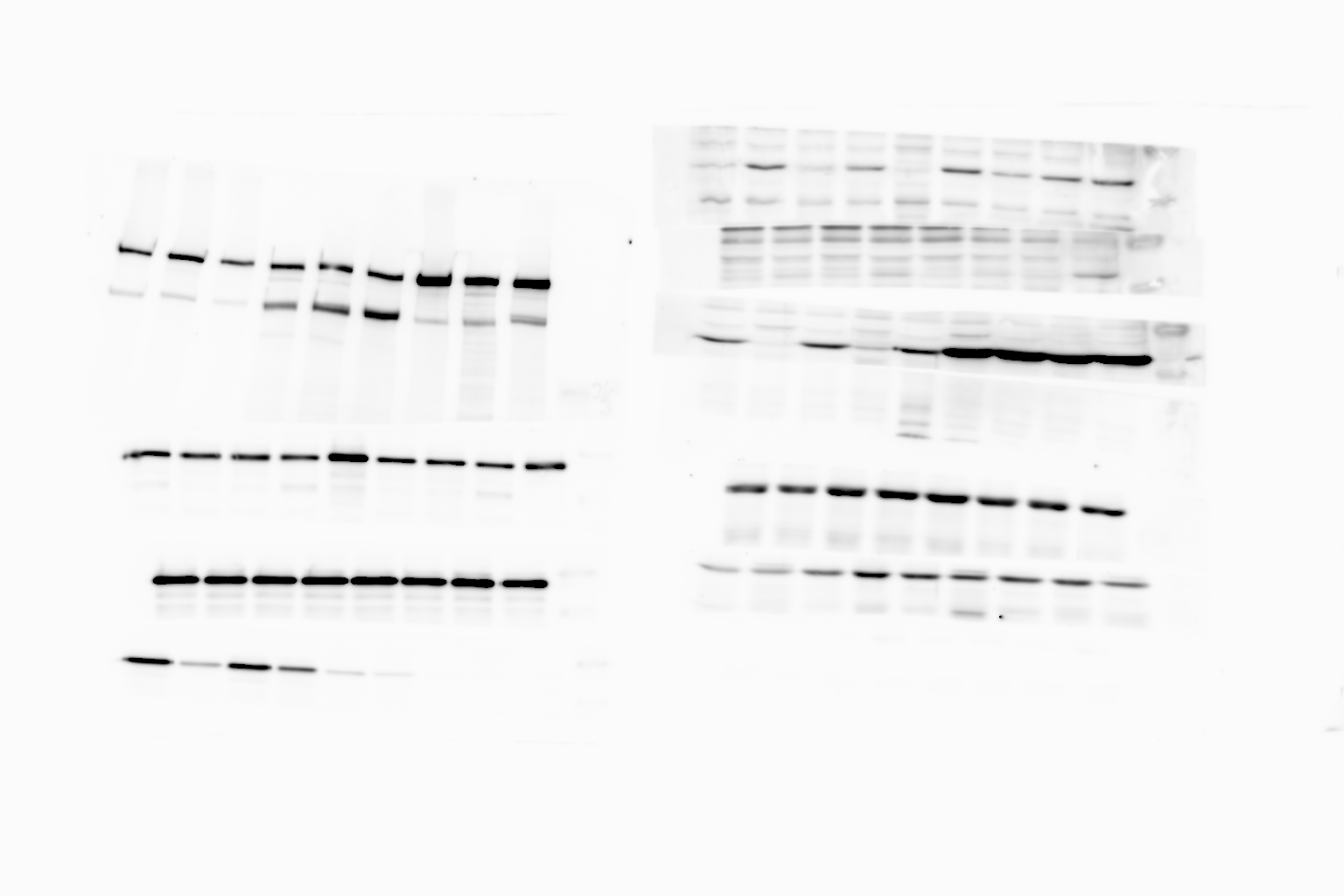

Supplement: Figure 1—source data 2. [file elife-102205-fig1-data2.zip › Figure 1-source data 2. Original files for western blot analysis displayed in Figure 1D/F1D plectin/160610_HCC-07-5m.tif]

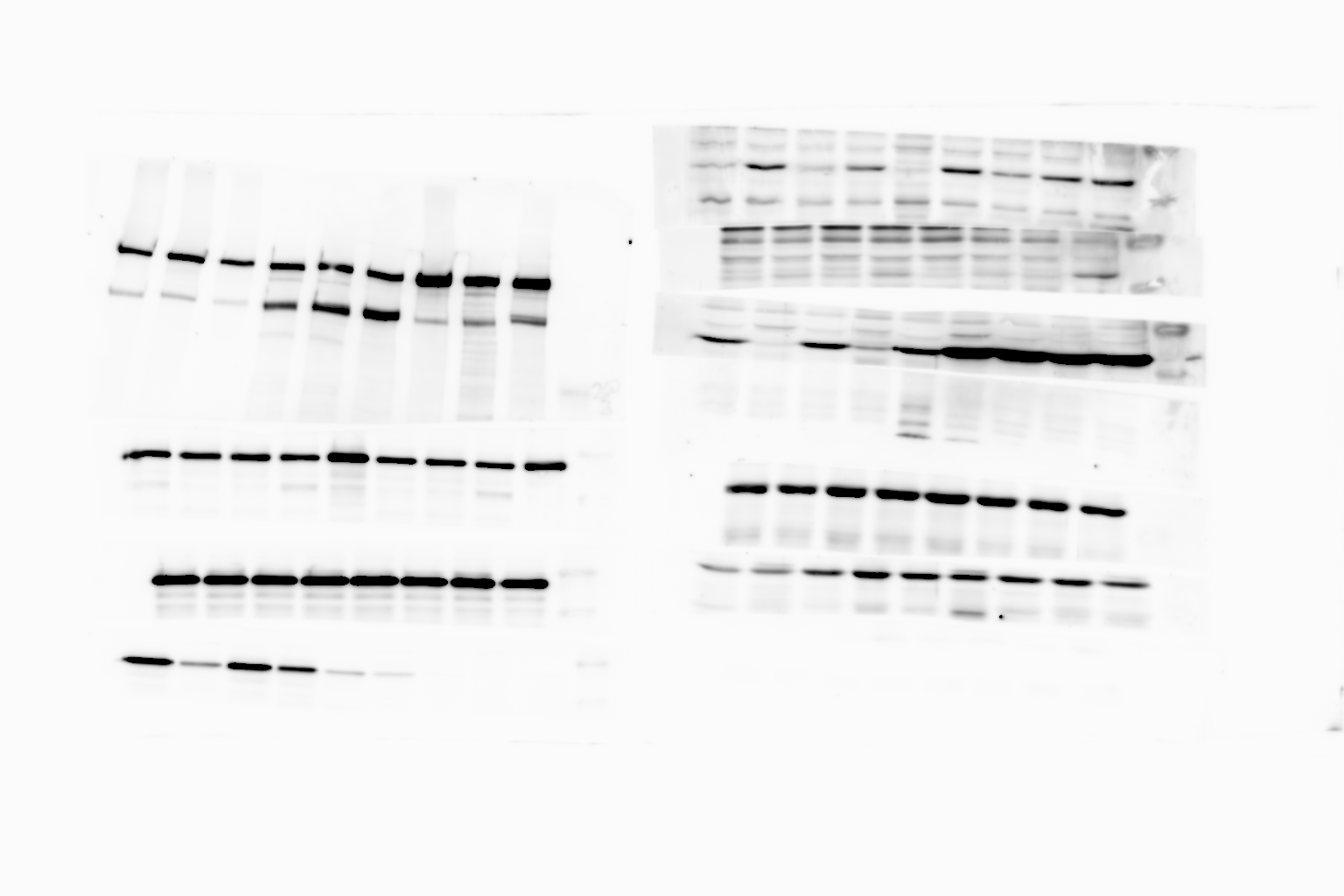

Supplement: Figure 1—source data 2. [file elife-102205-fig1-data2.zip › Figure 1-source data 2. Original files for western blot analysis displayed in Figure 1D/F1D plectin/160610_HCC-08-10m.tif]

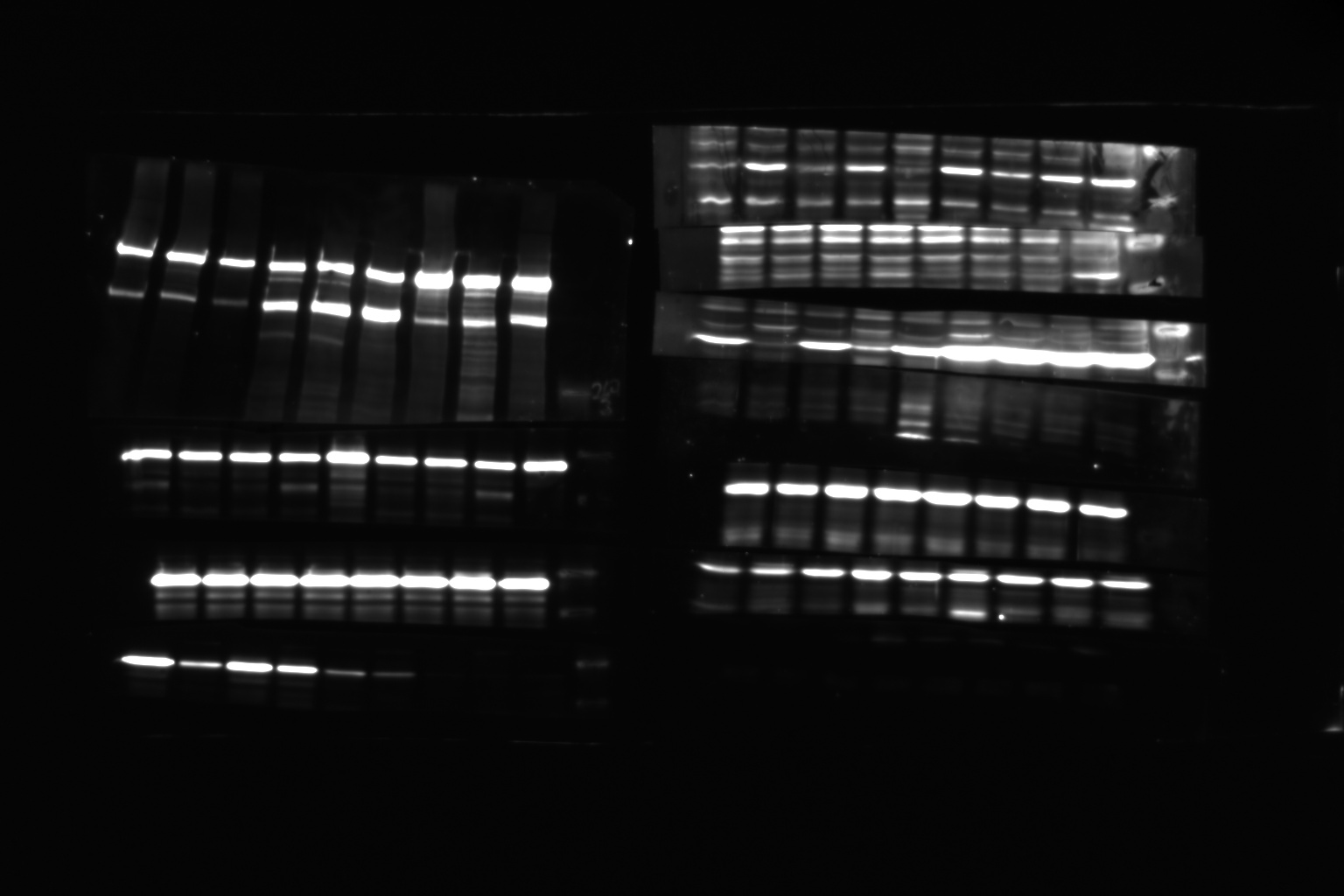

Supplement: Figure 1—source data 2. [file elife-102205-fig1-data2.zip › Figure 1-source data 2. Original files for western blot analysis displayed in Figure 1D/F1D plectin/160610_HCC-SUM-1131s.tif]

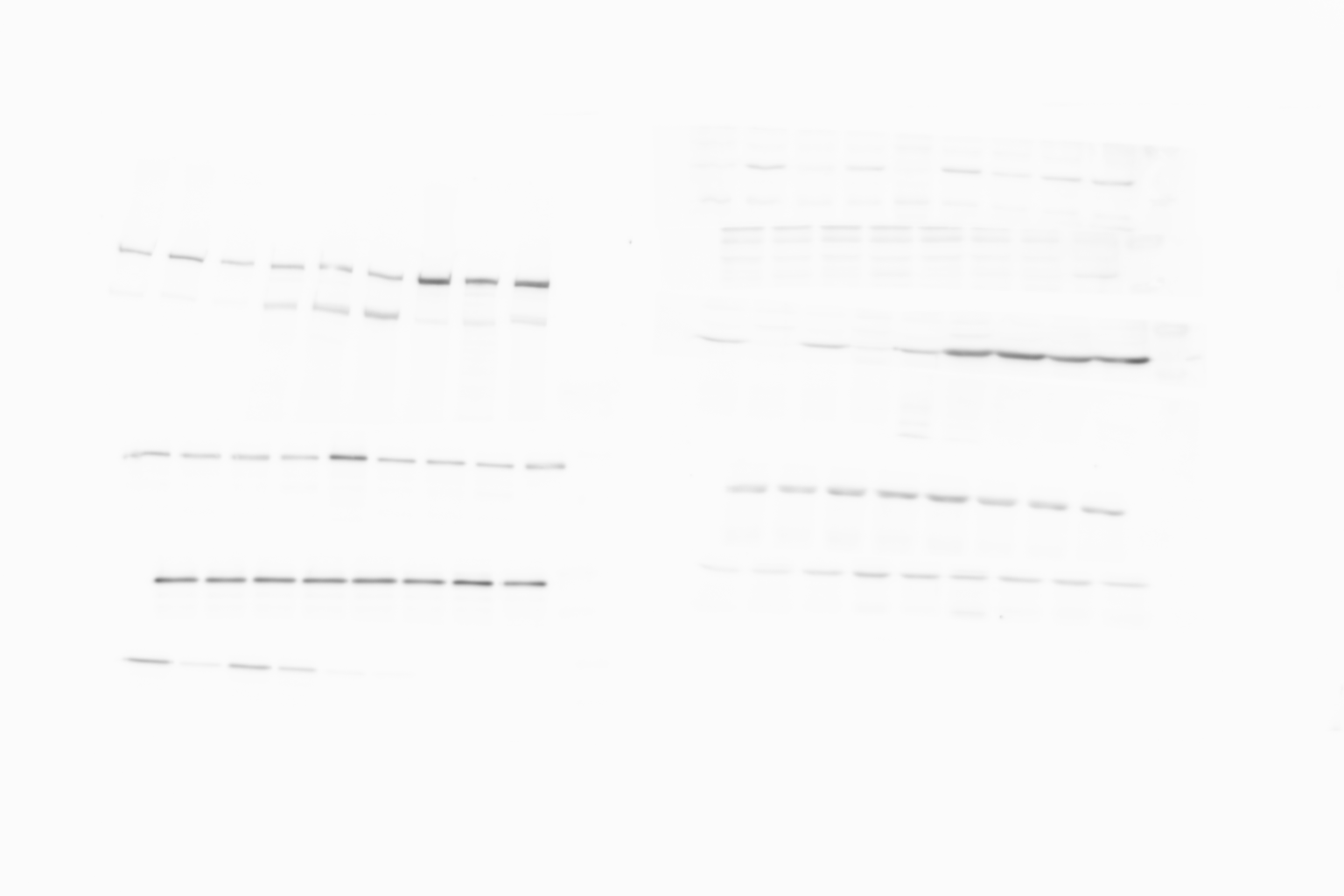

Supplement: Figure 1—source data 2. [file elife-102205-fig1-data2.zip › Figure 1-source data 2. Original files for western blot analysis displayed in Figure 1D/F1D plectin/20160610_1636.tif]

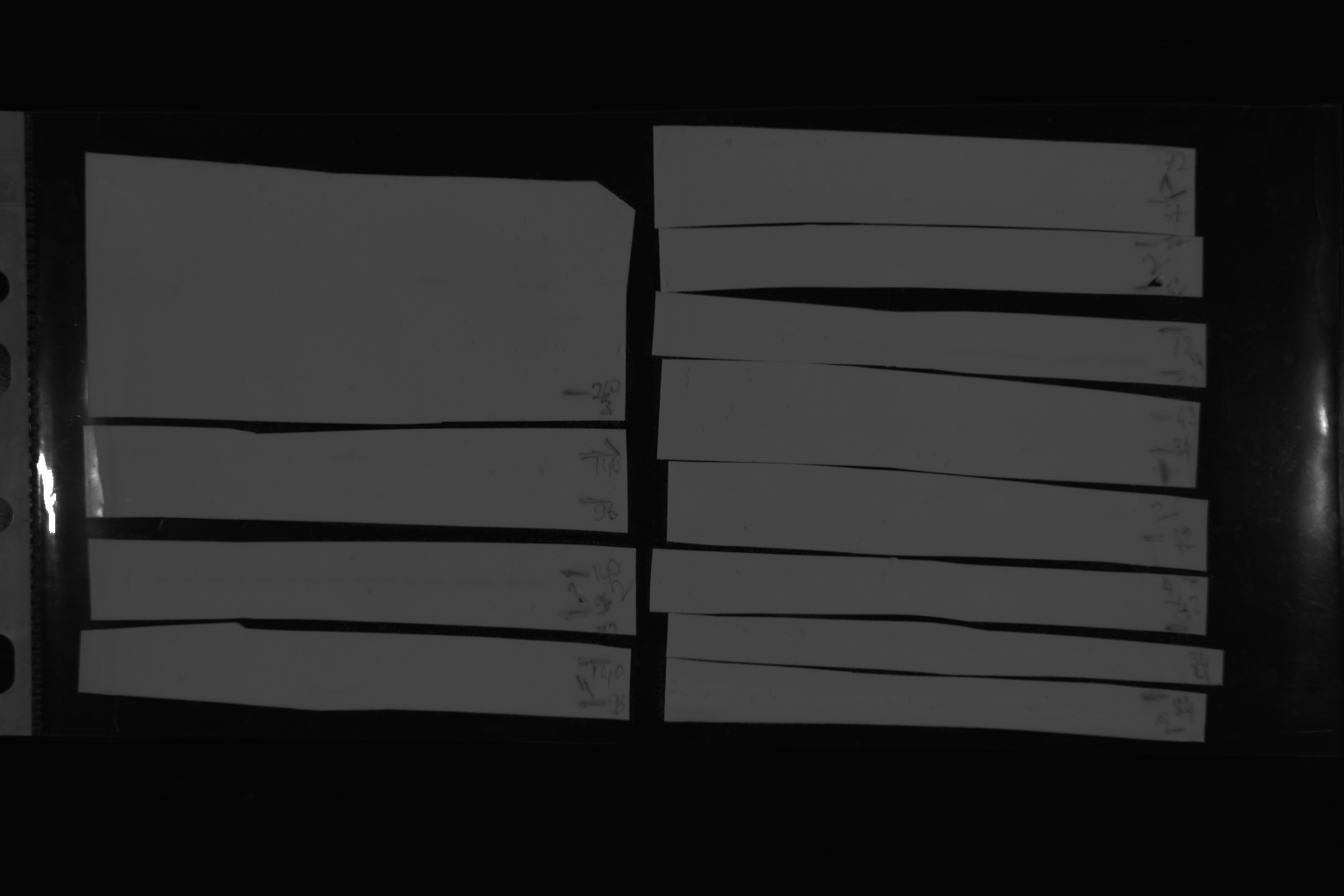

Supplement: Figure 1—source data 2. [file elife-102205-fig1-data2.zip › Figure 1-source data 2. Original files for western blot analysis displayed in Figure 1D/F1D plectin/V_20160610_1636.tif]

Figure 1H

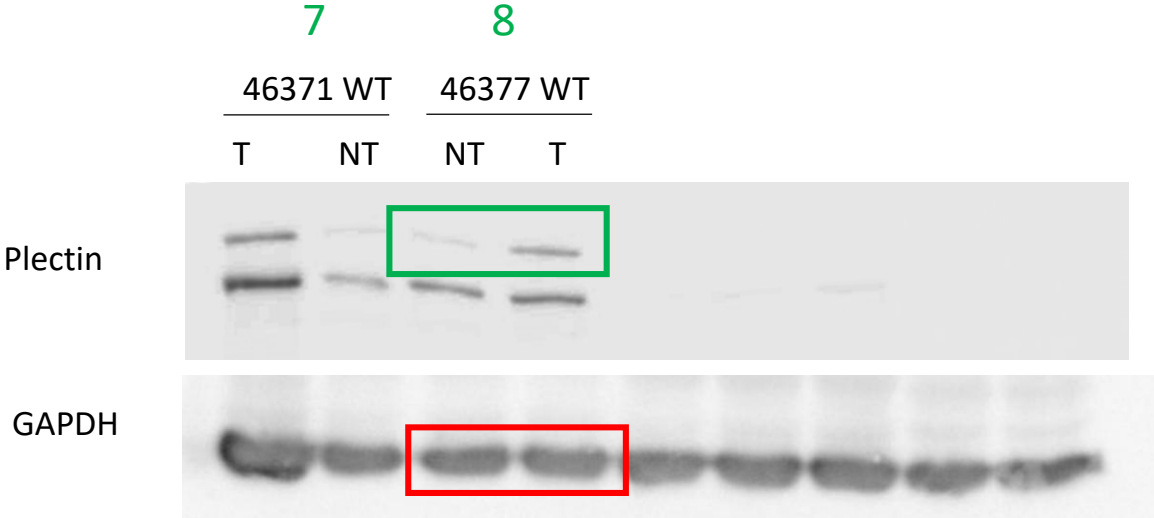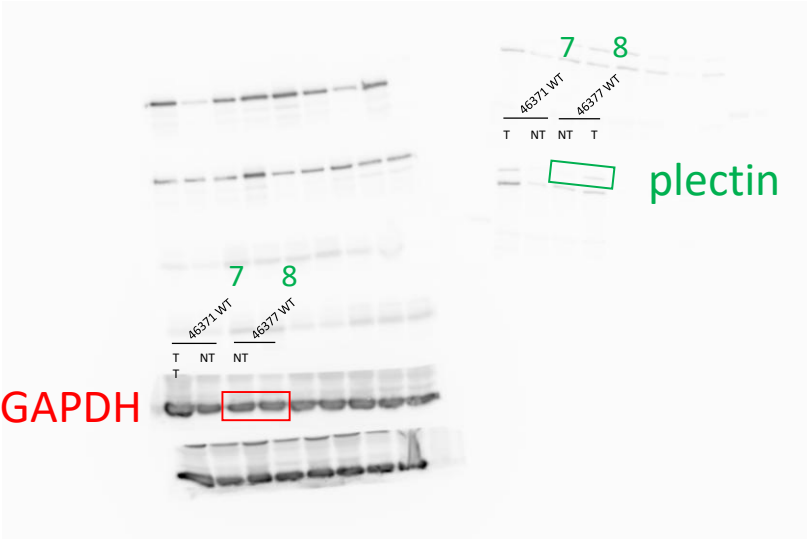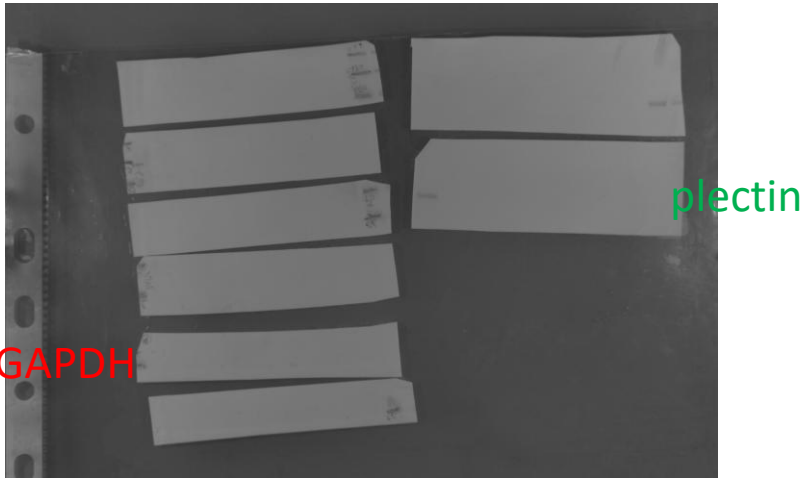

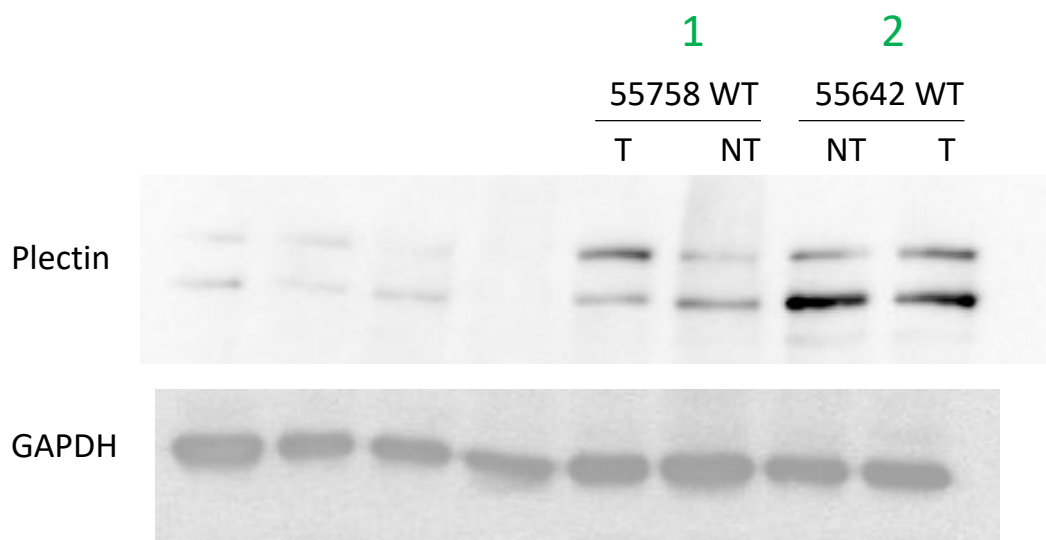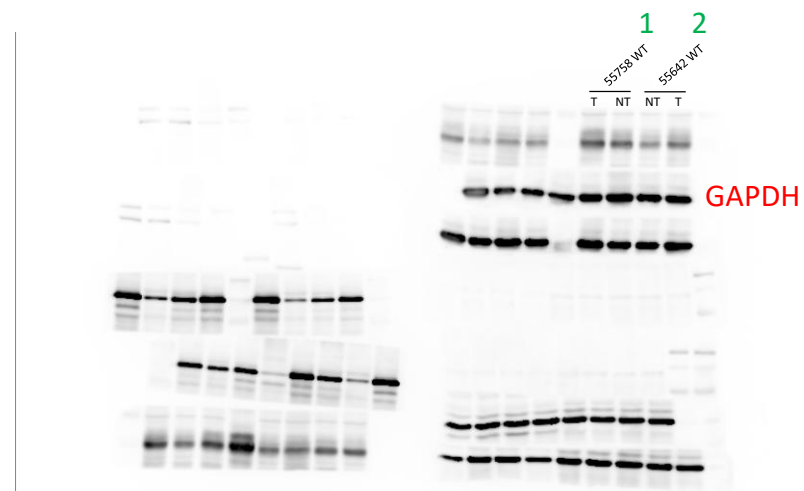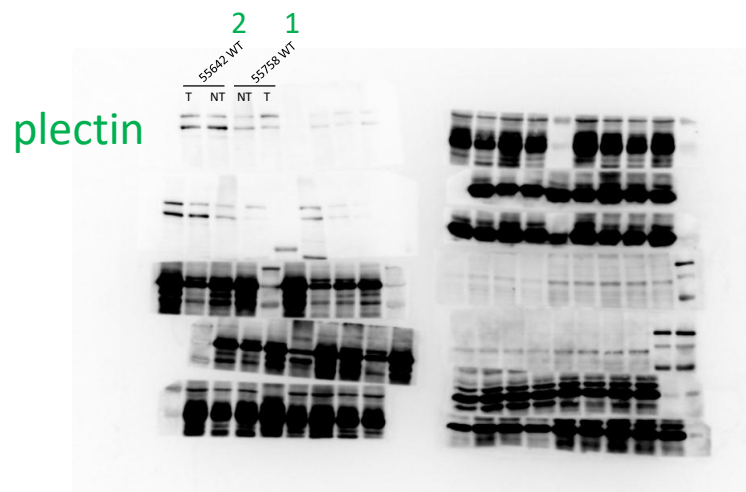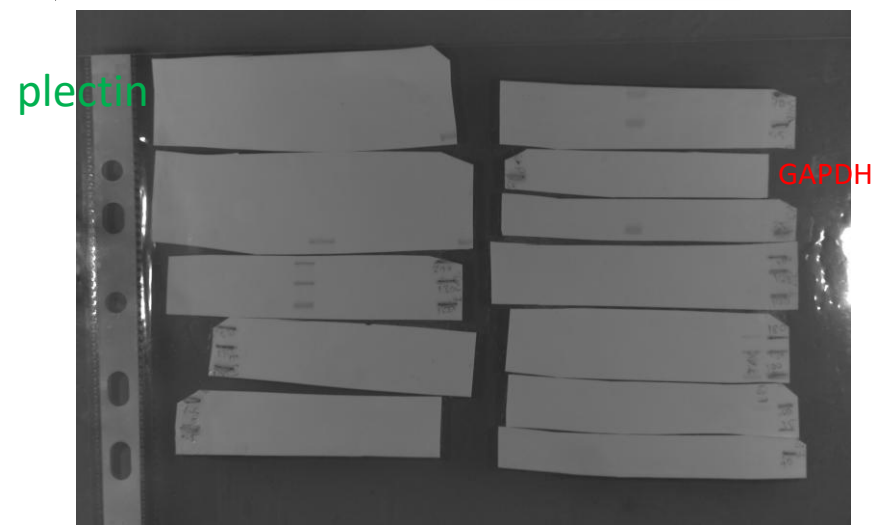

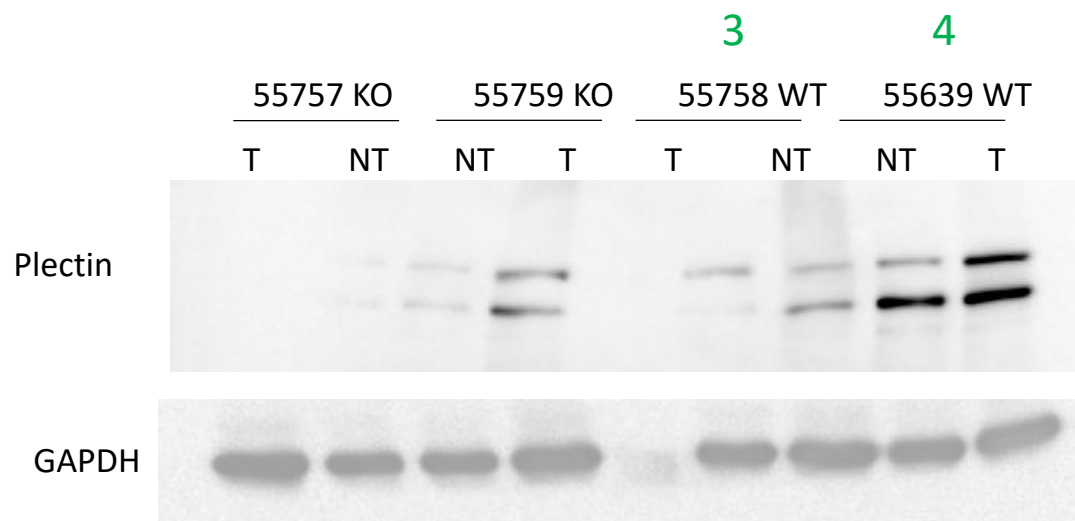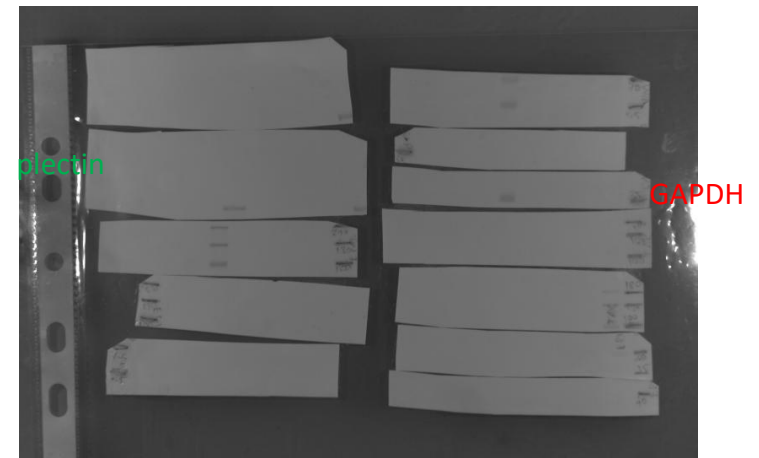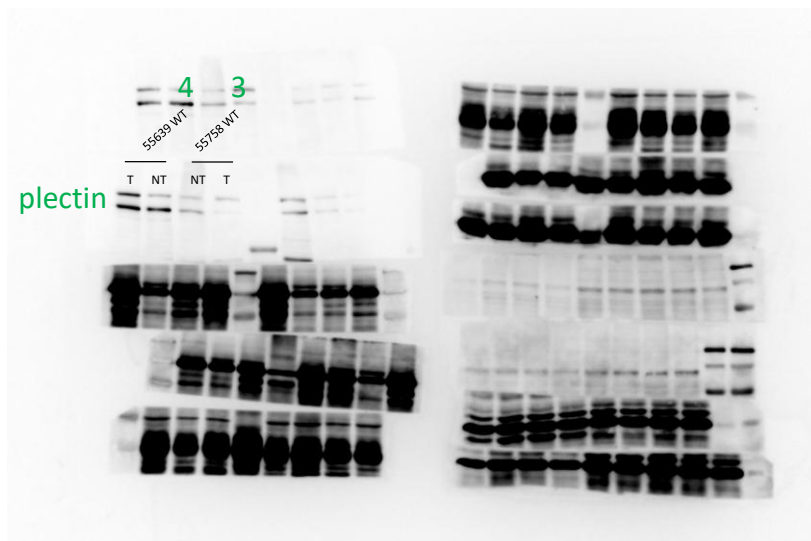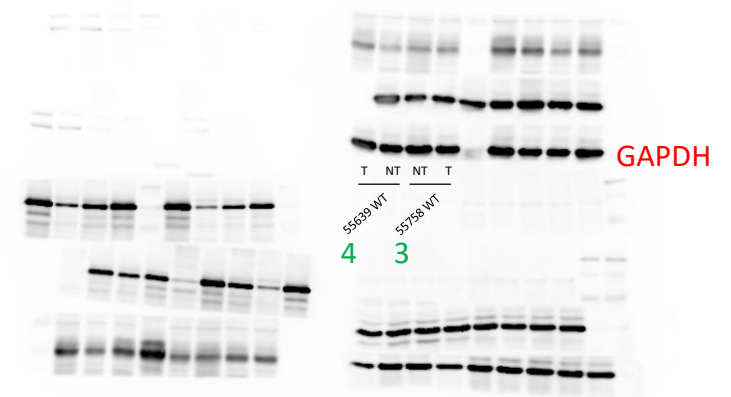

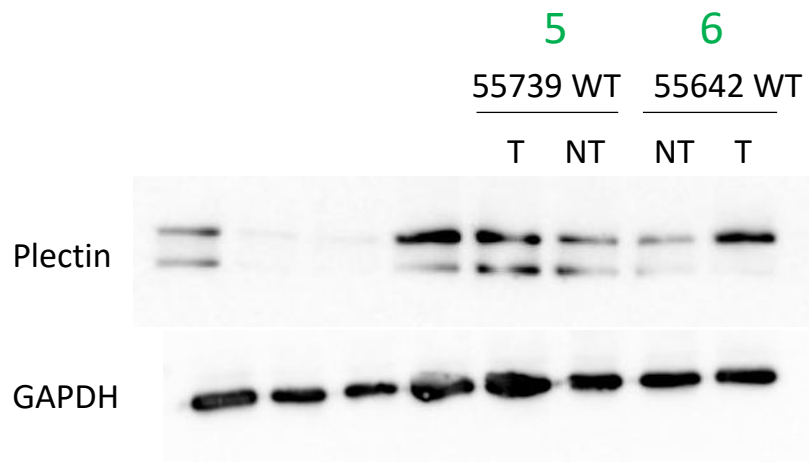

reprobed (see subfolder „reprobe“)

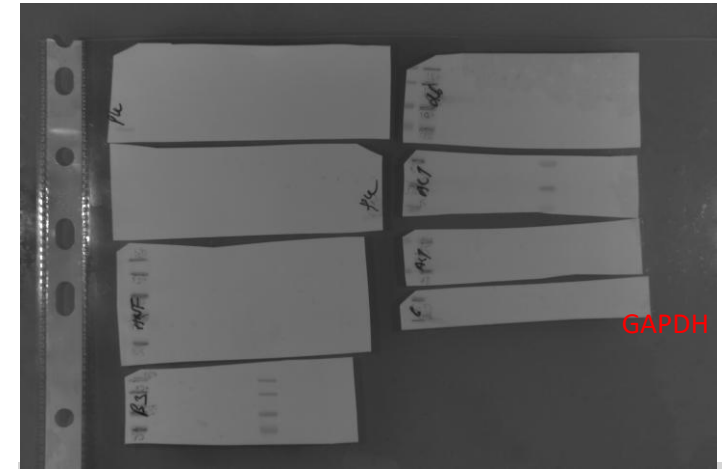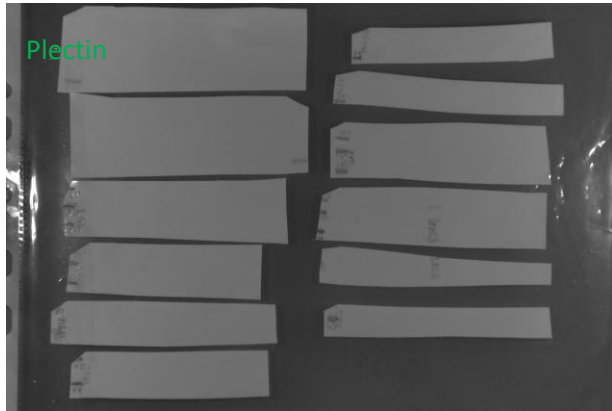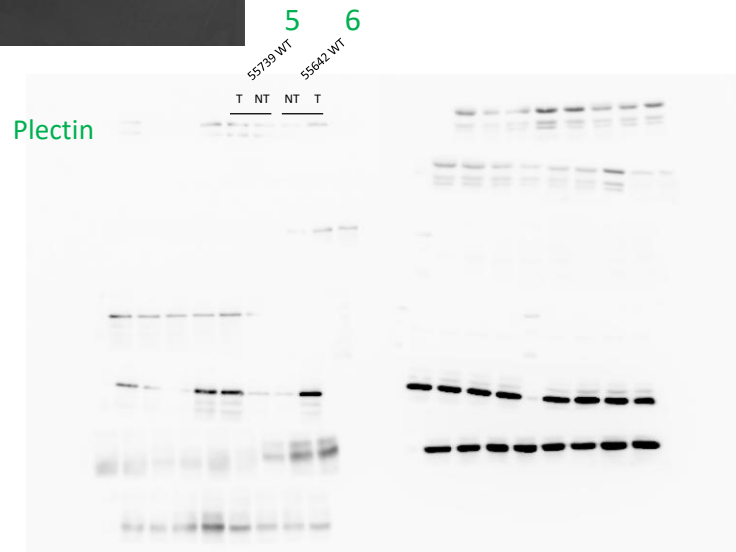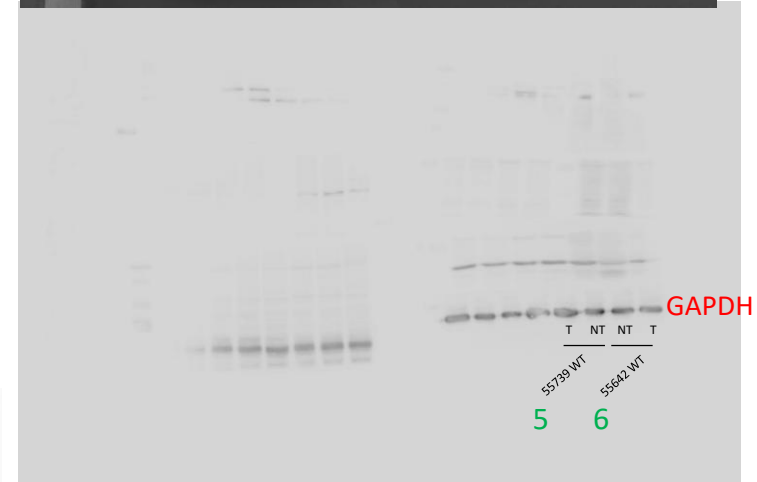

Supplement: Figure 1—source data 3. [file elife-102205-fig1-data3.pdf]

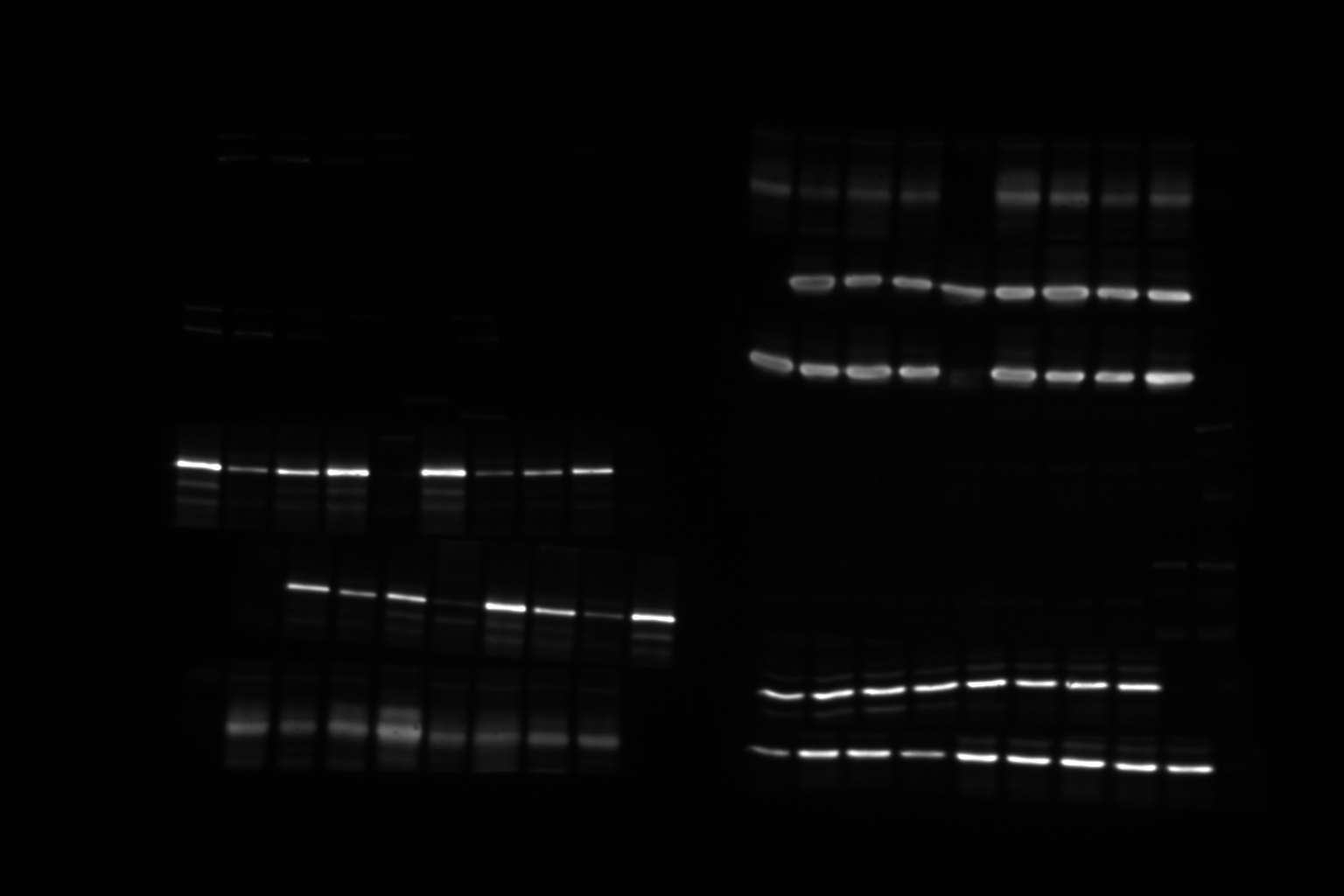

Supplement: Figure 1—source data 4. [file elife-102205-fig1-data4.zip › Figure 1-source data 4. Original files for western blot analysis displayed in Figure 1H/1 2 3 4/1min.tif]

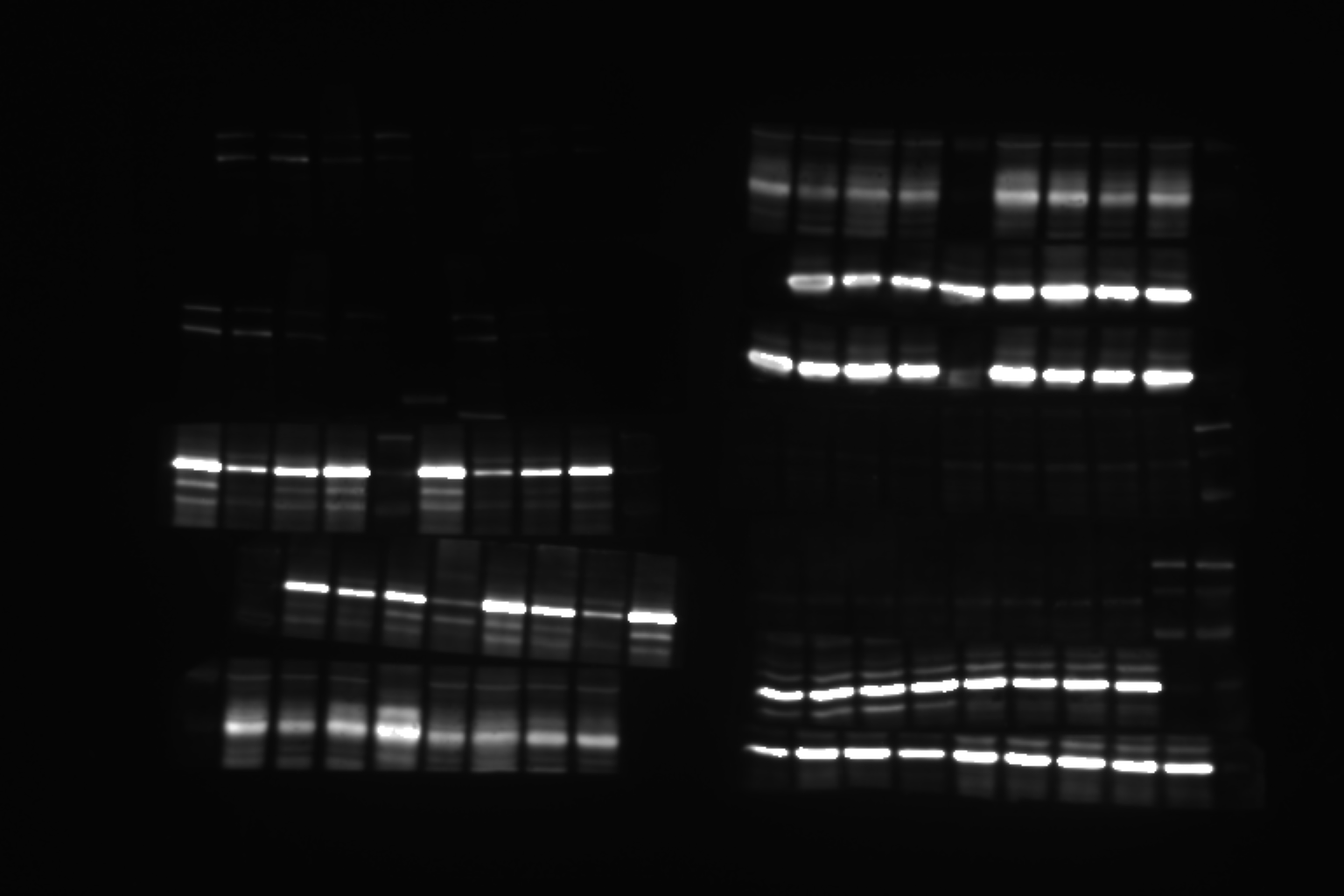

Supplement: Figure 1—source data 4. [file elife-102205-fig1-data4.zip › Figure 1-source data 4. Original files for western blot analysis displayed in Figure 1H/1 2 3 4/5min.tif]

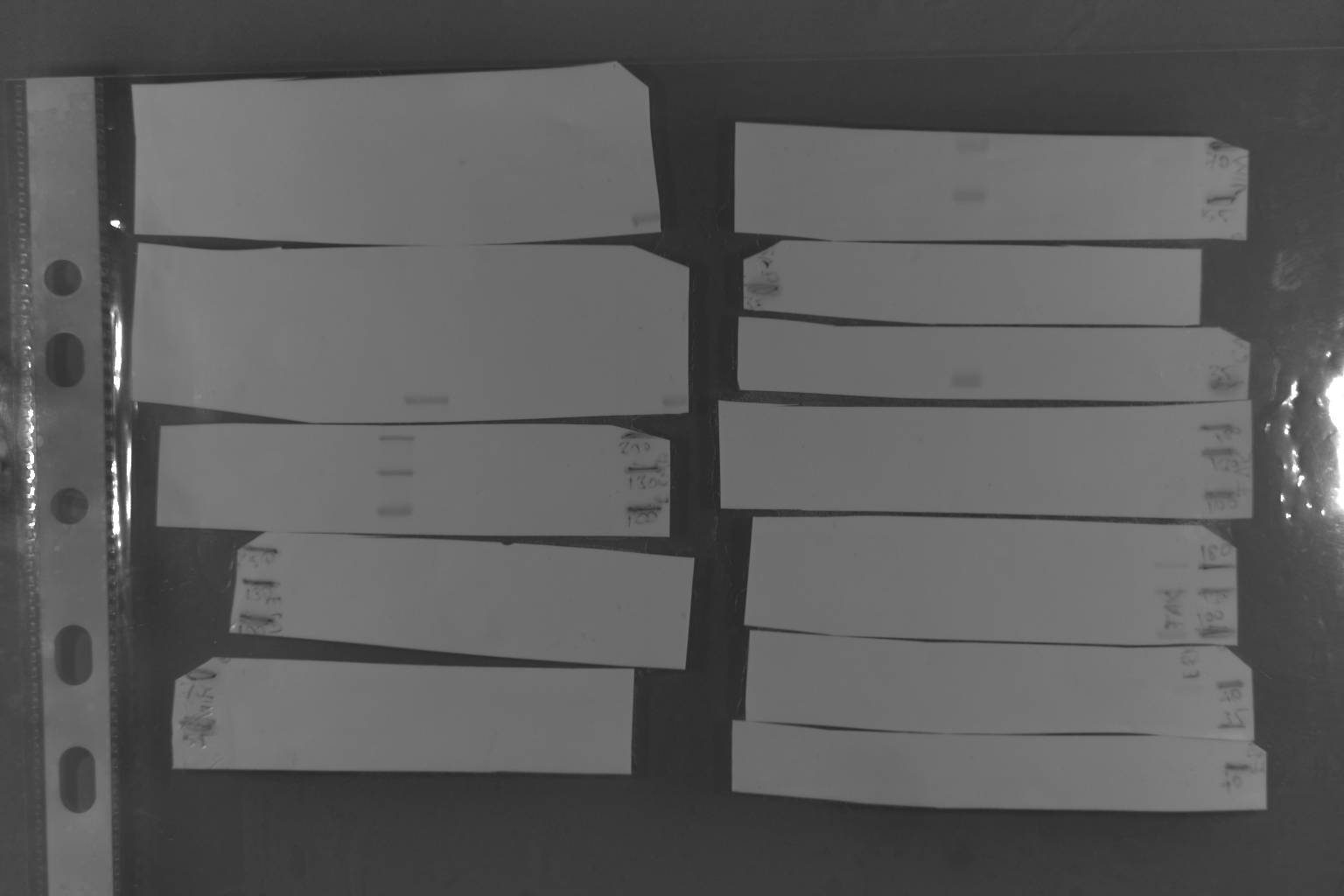

Supplement: Figure 1—source data 4. [file elife-102205-fig1-data4.zip › Figure 1-source data 4. Original files for western blot analysis displayed in Figure 1H/1 2 3 4/V_1sec.tif]

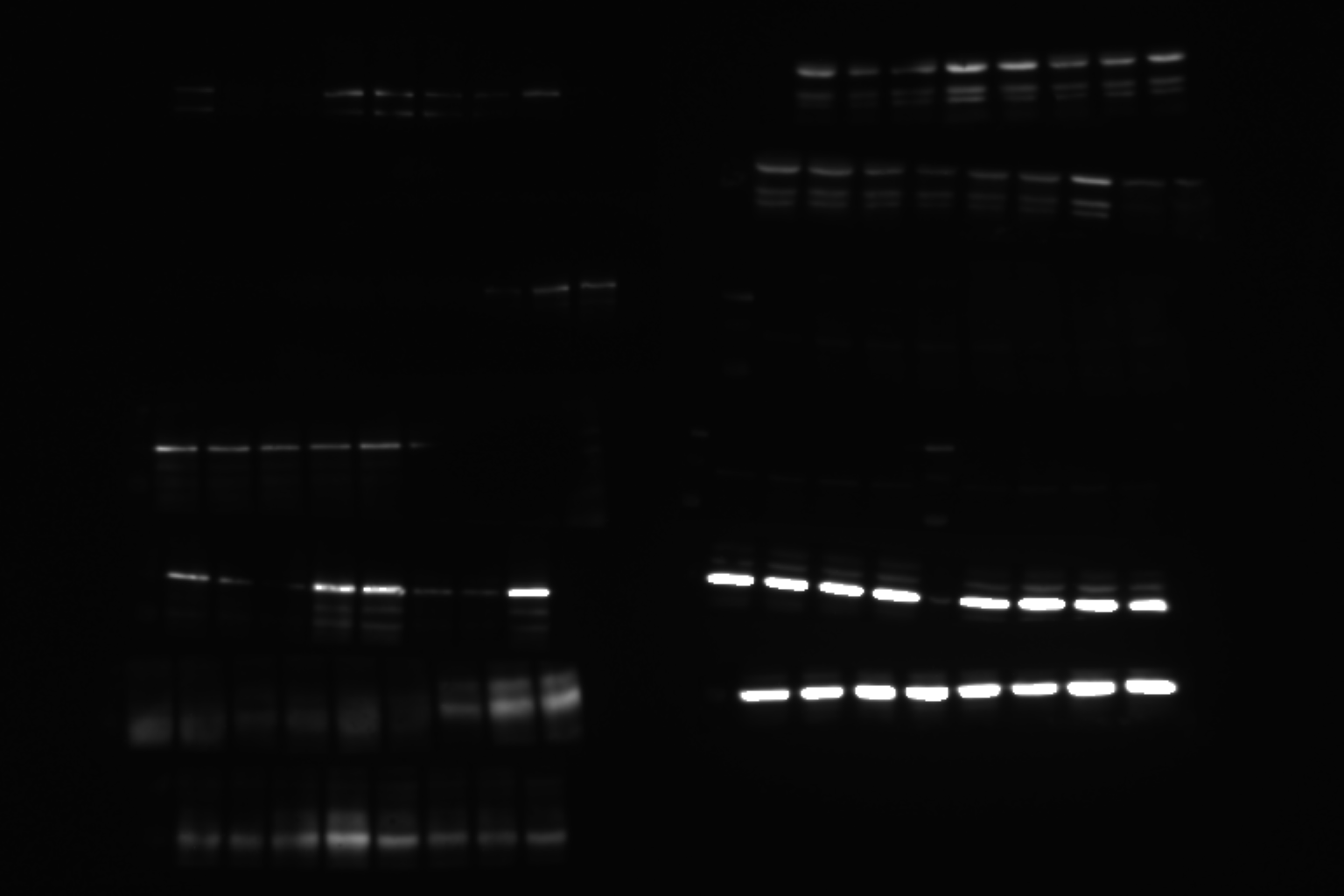

Supplement: Figure 1—source data 4. [file elife-102205-fig1-data4.zip › Figure 1-source data 4. Original files for western blot analysis displayed in Figure 1H/5 6/10min.tif]

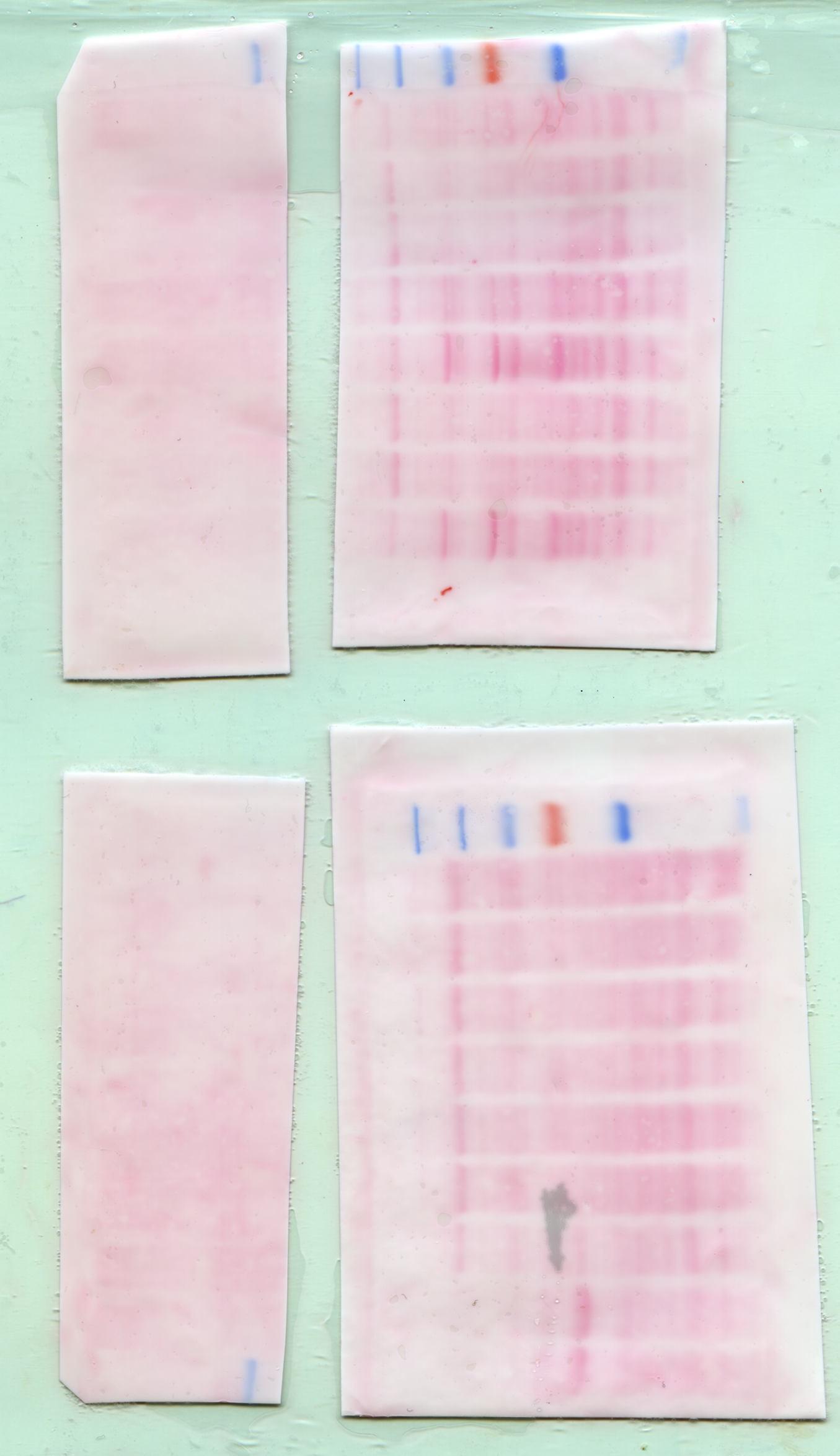

Supplement: Figure 1—source data 4. [file elife-102205-fig1-data4.zip › Figure 1-source data 4. Original files for western blot analysis displayed in Figure 1H/5 6/170623_Ponceau_002.tif]

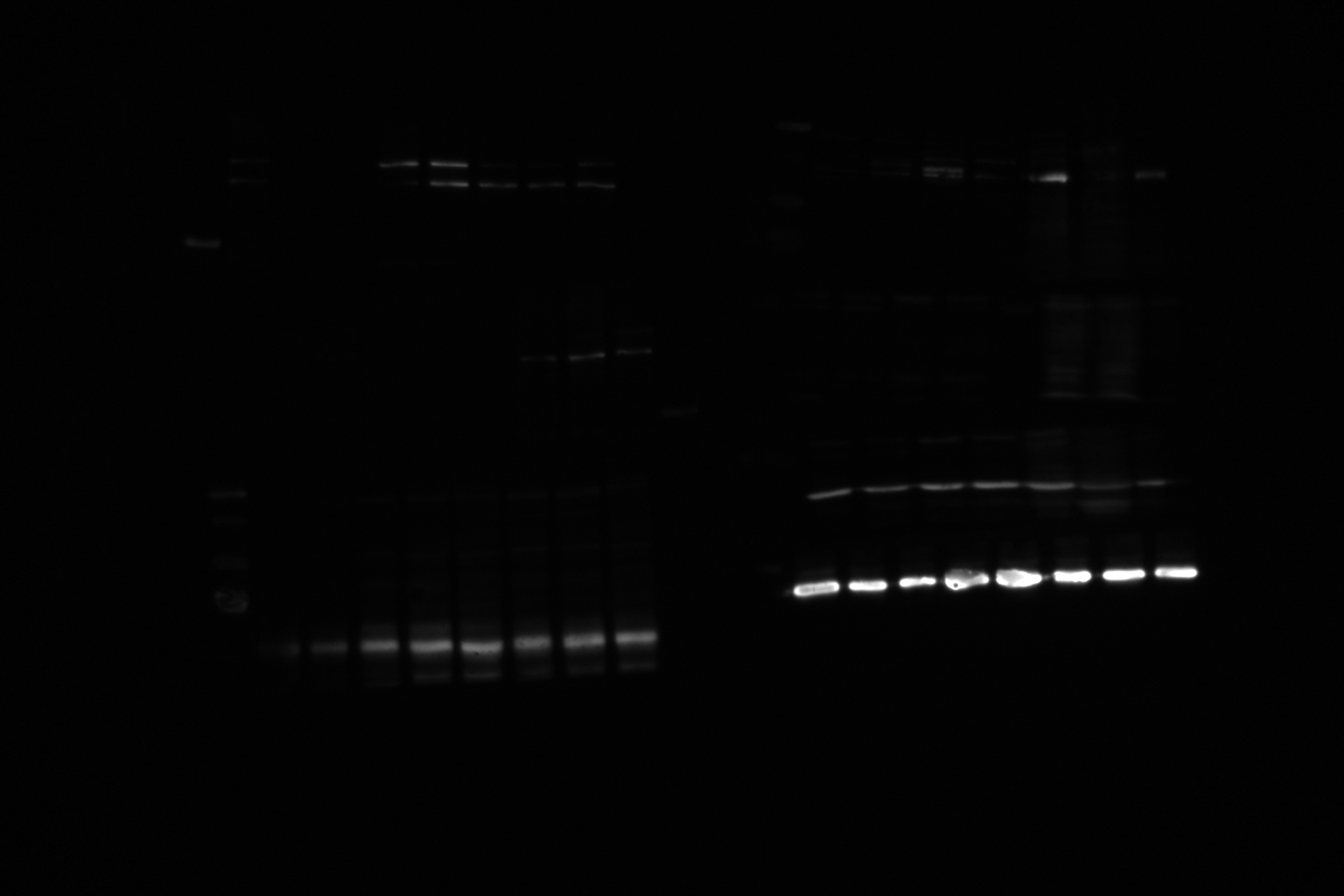

Supplement: Figure 1—source data 4. [file elife-102205-fig1-data4.zip › Figure 1-source data 4. Original files for western blot analysis displayed in Figure 1H/5 6/reprobe/1min.tif]

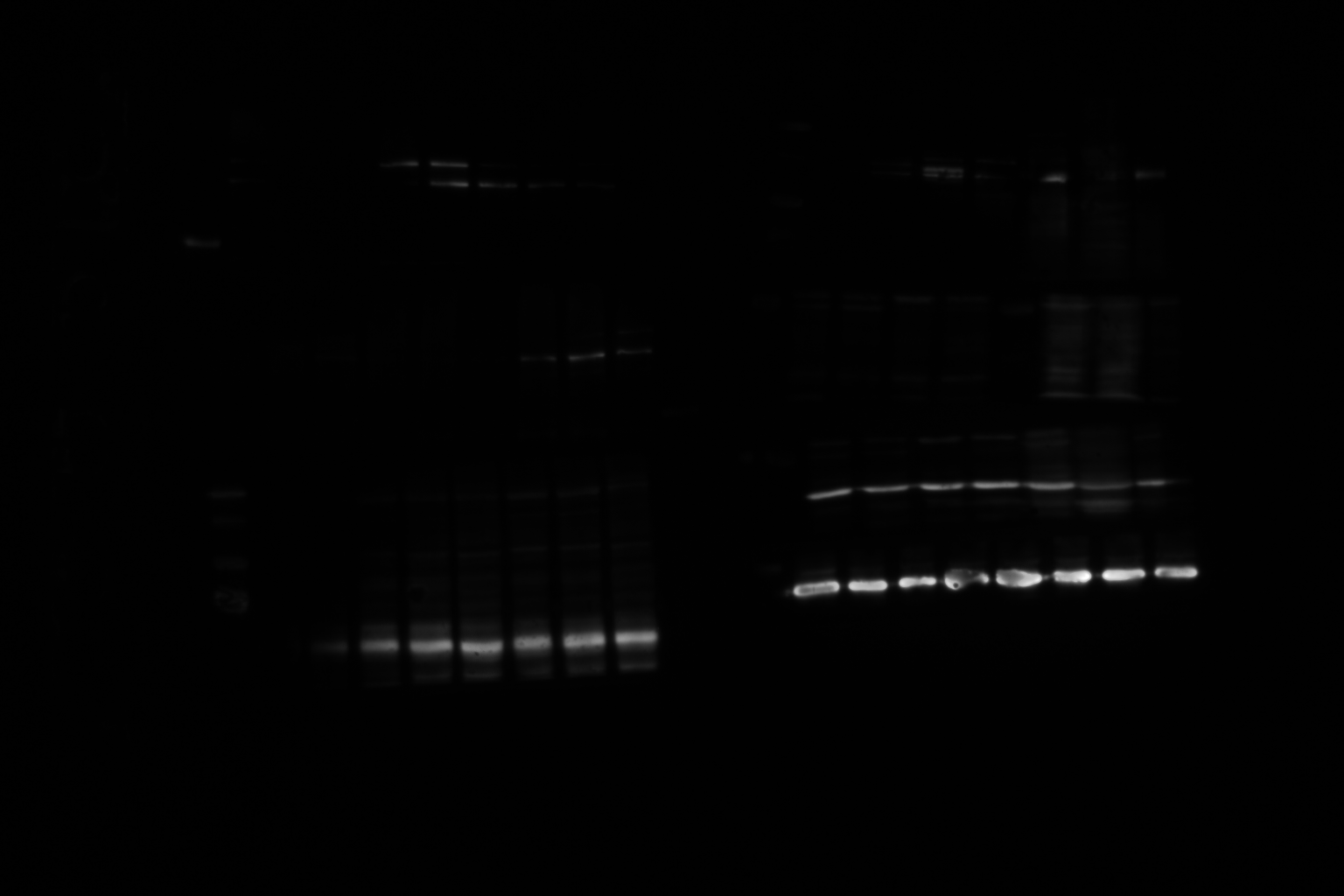

Supplement: Figure 1—source data 4. [file elife-102205-fig1-data4.zip › Figure 1-source data 4. Original files for western blot analysis displayed in Figure 1H/5 6/reprobe/5min.tif]

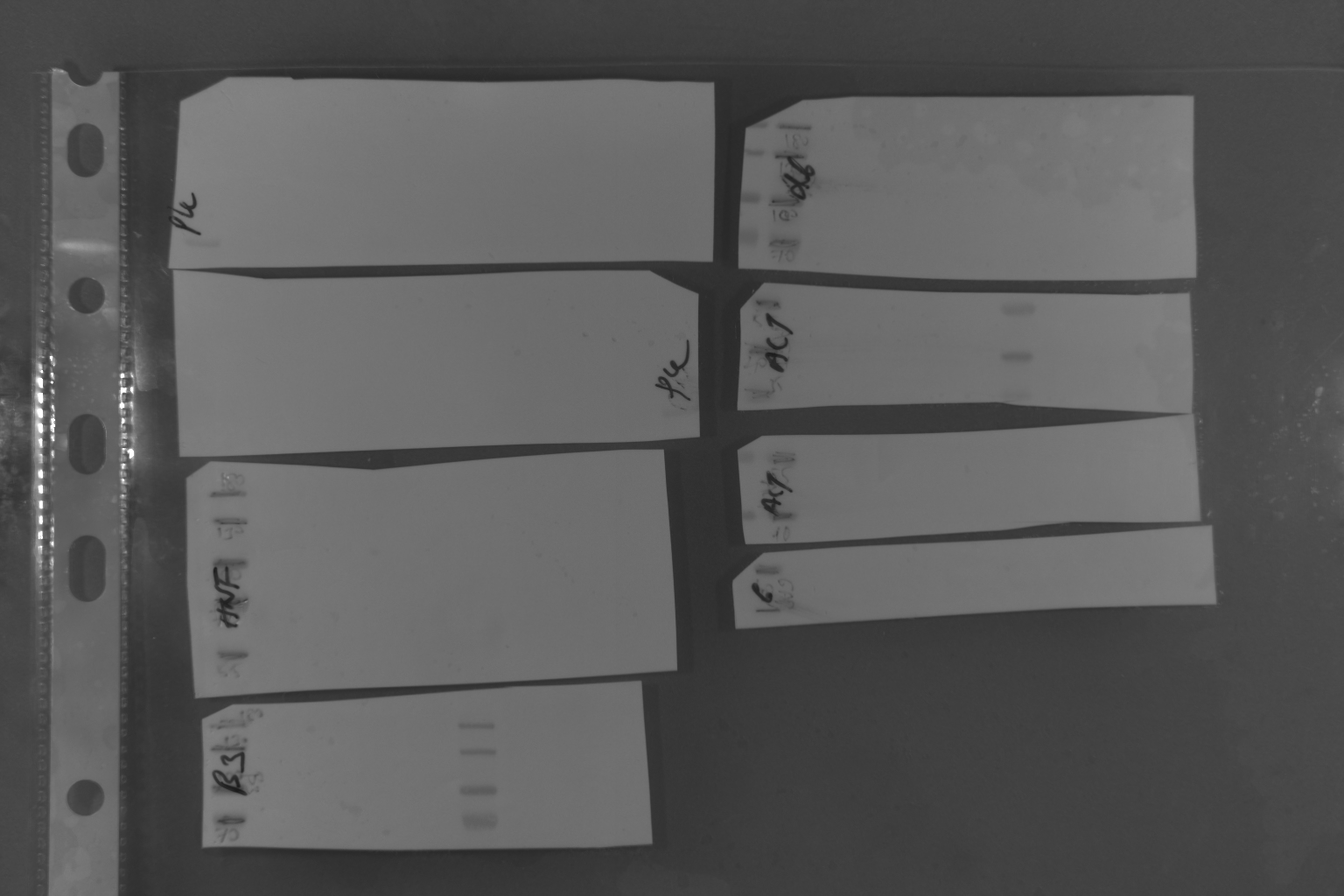

Supplement: Figure 1—source data 4. [file elife-102205-fig1-data4.zip › Figure 1-source data 4. Original files for western blot analysis displayed in Figure 1H/5 6/reprobe/V_1sec.tif]

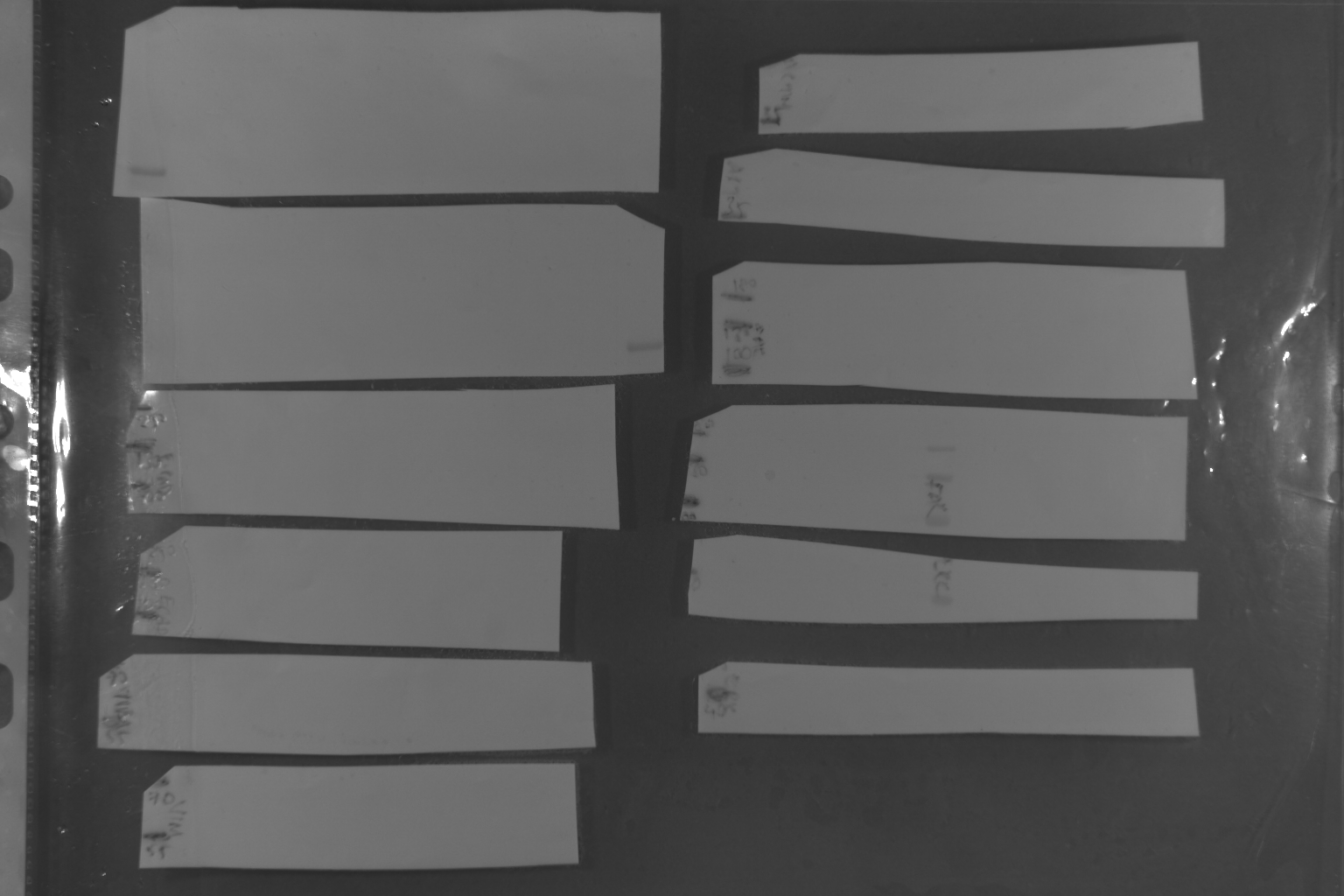

Supplement: Figure 1—source data 4. [file elife-102205-fig1-data4.zip › Figure 1-source data 4. Original files for western blot analysis displayed in Figure 1H/5 6/V_1sec.tif]

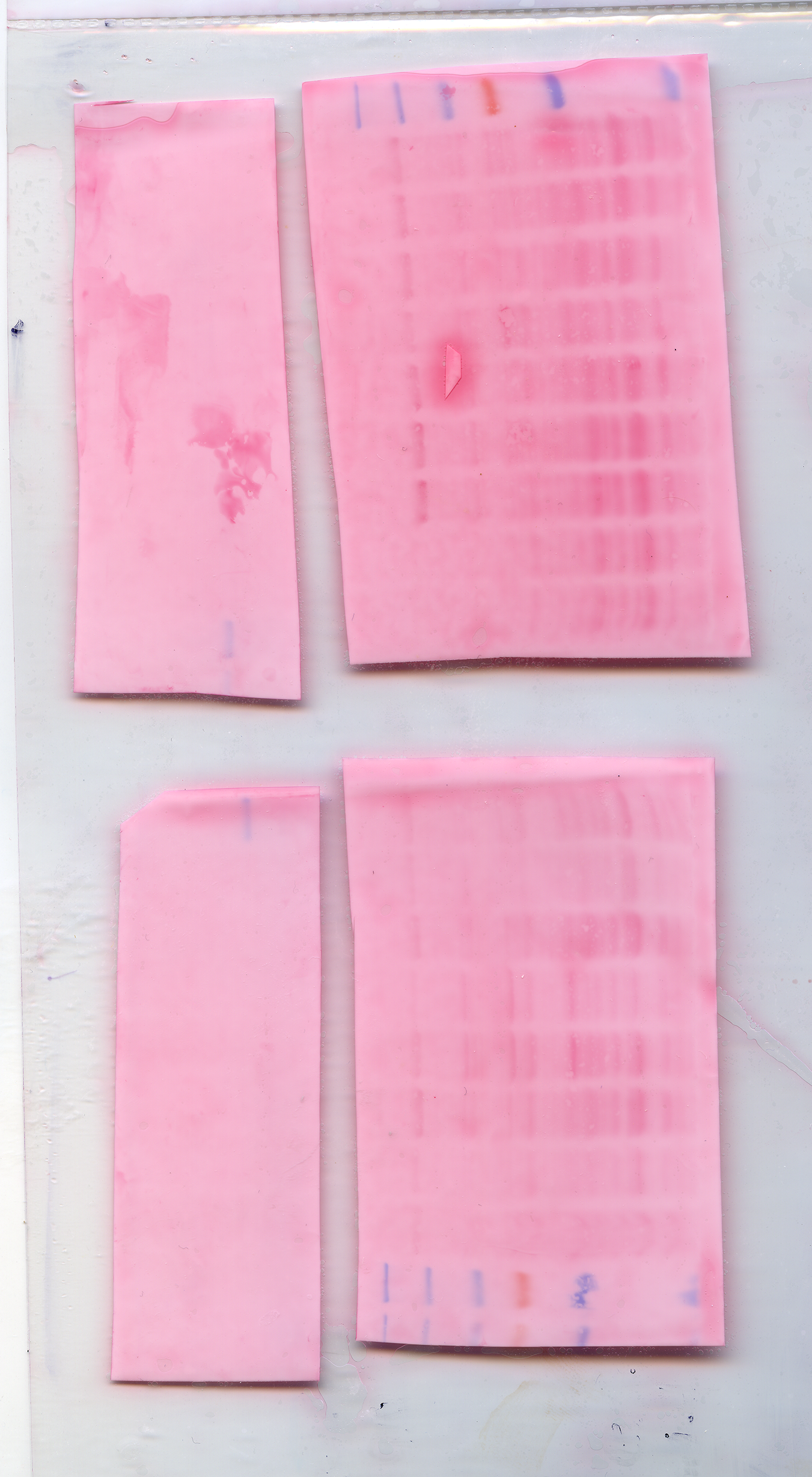

Supplement: Figure 1—source data 4. [file elife-102205-fig1-data4.zip › Figure 1-source data 4. Original files for western blot analysis displayed in Figure 1H/7 8/170714_pomc005.tif]

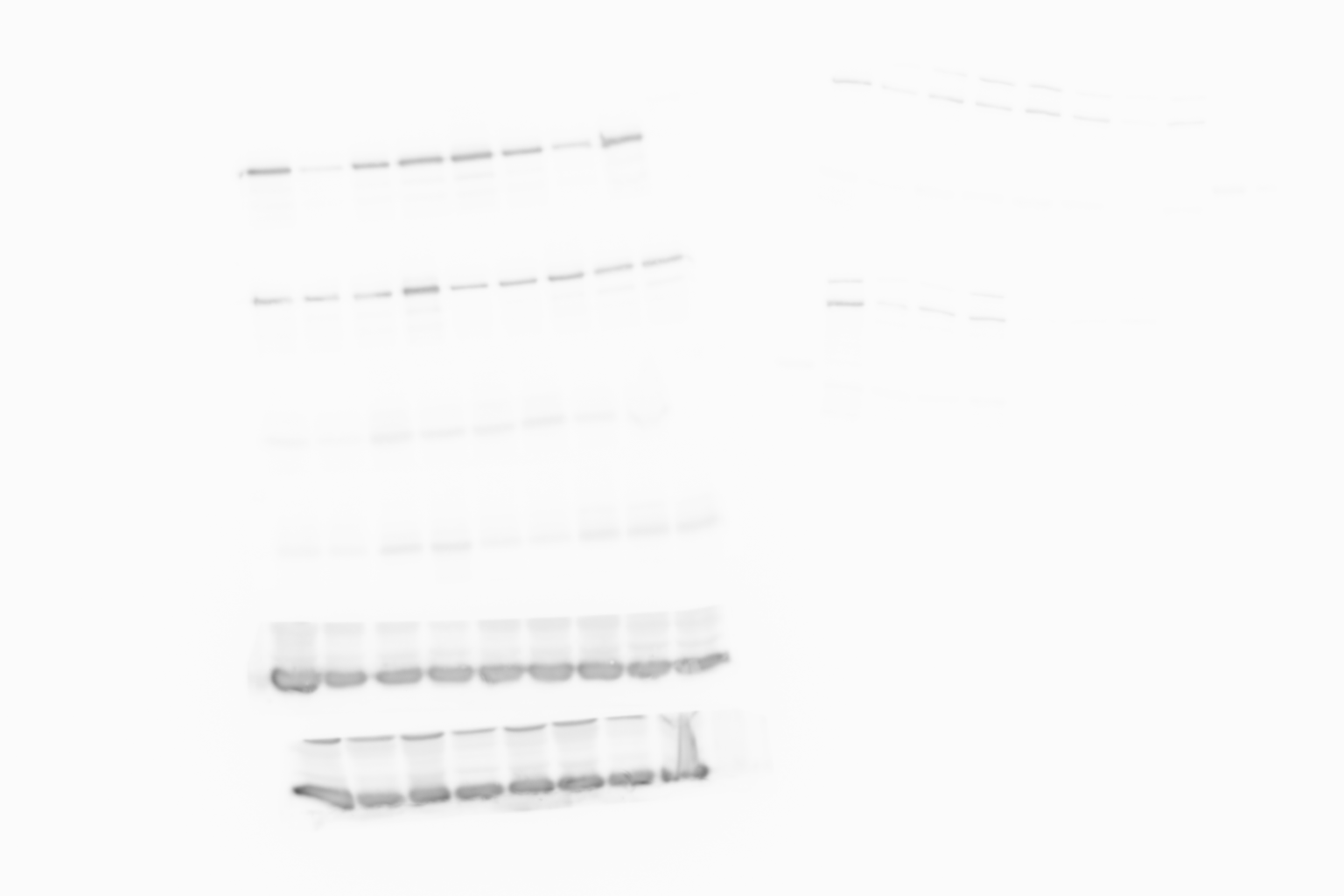

Supplement: Figure 1—source data 4. [file elife-102205-fig1-data4.zip › Figure 1-source data 4. Original files for western blot analysis displayed in Figure 1H/7 8/2min.tif]

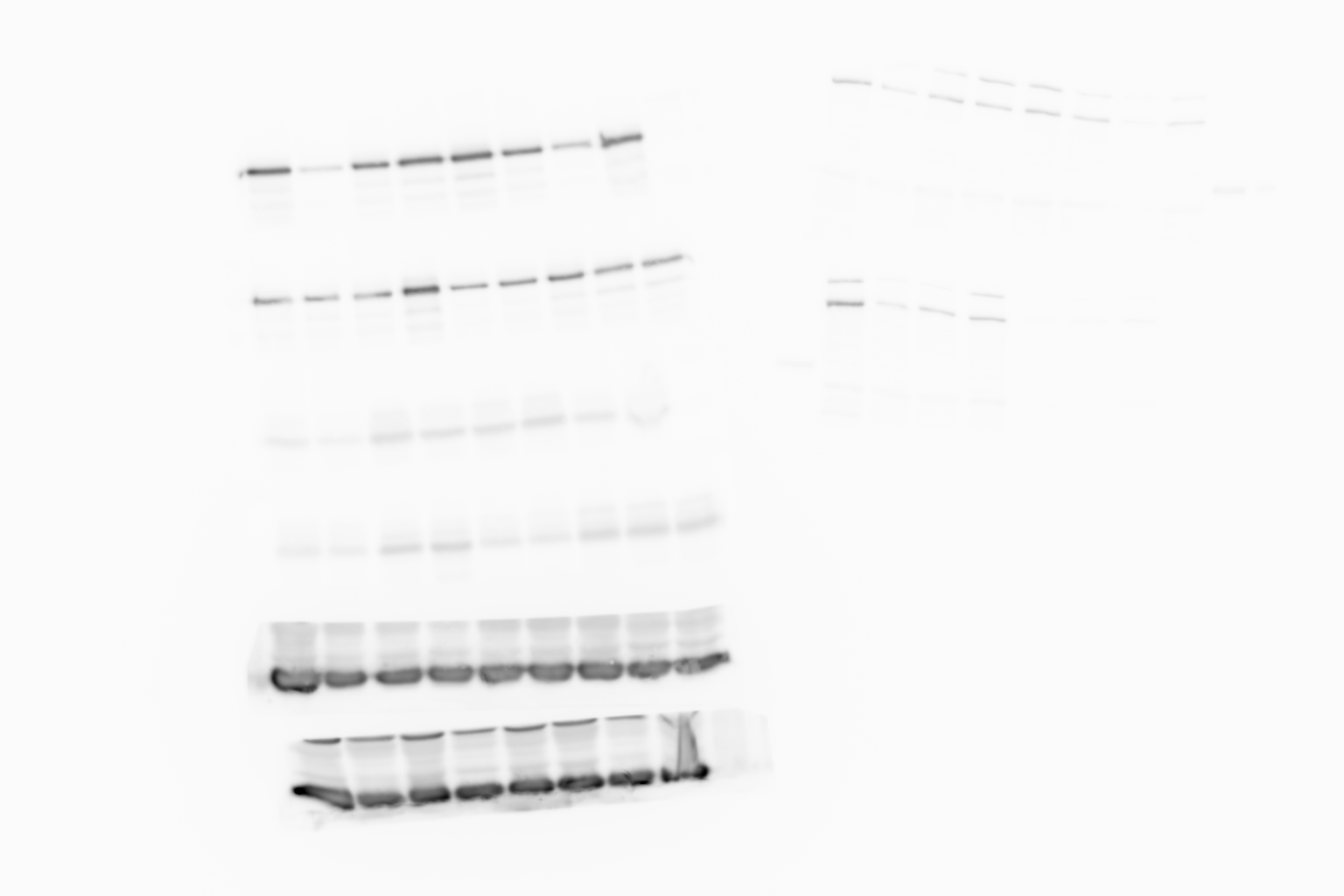

Supplement: Figure 1—source data 4. [file elife-102205-fig1-data4.zip › Figure 1-source data 4. Original files for western blot analysis displayed in Figure 1H/7 8/5min.tif]

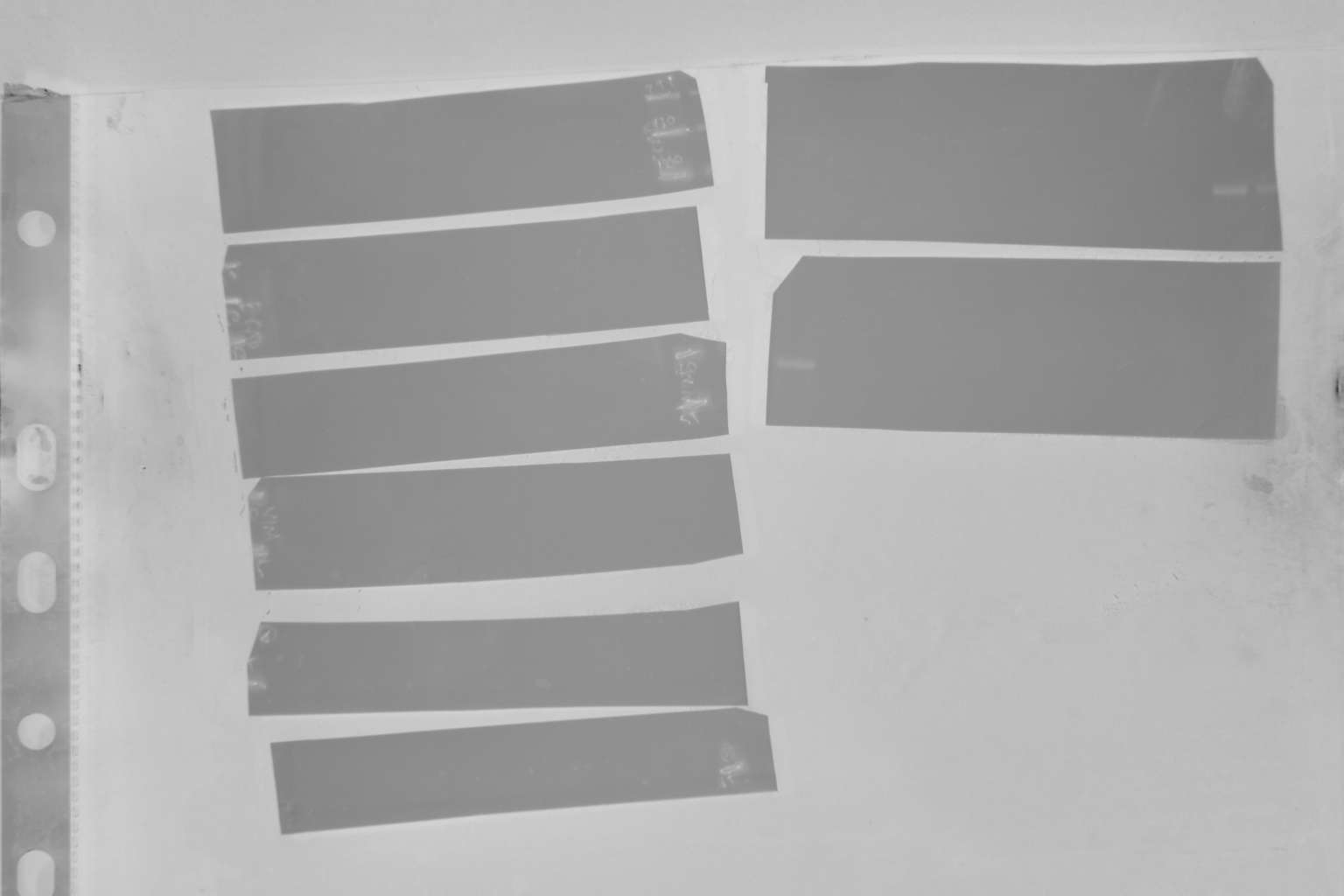

Supplement: Figure 1—source data 4. [file elife-102205-fig1-data4.zip › Figure 1-source data 4. Original files for western blot analysis displayed in Figure 1H/7 8/V_1sec.tif]

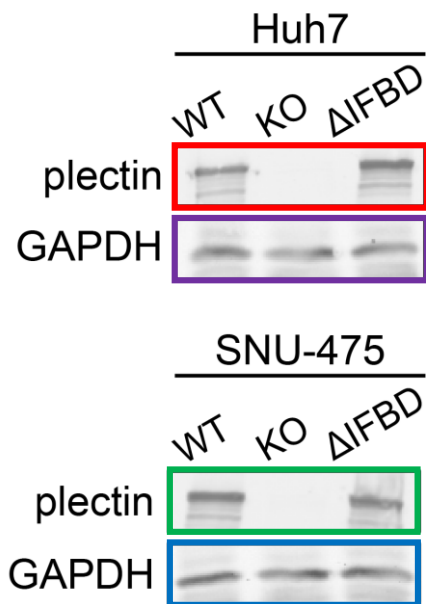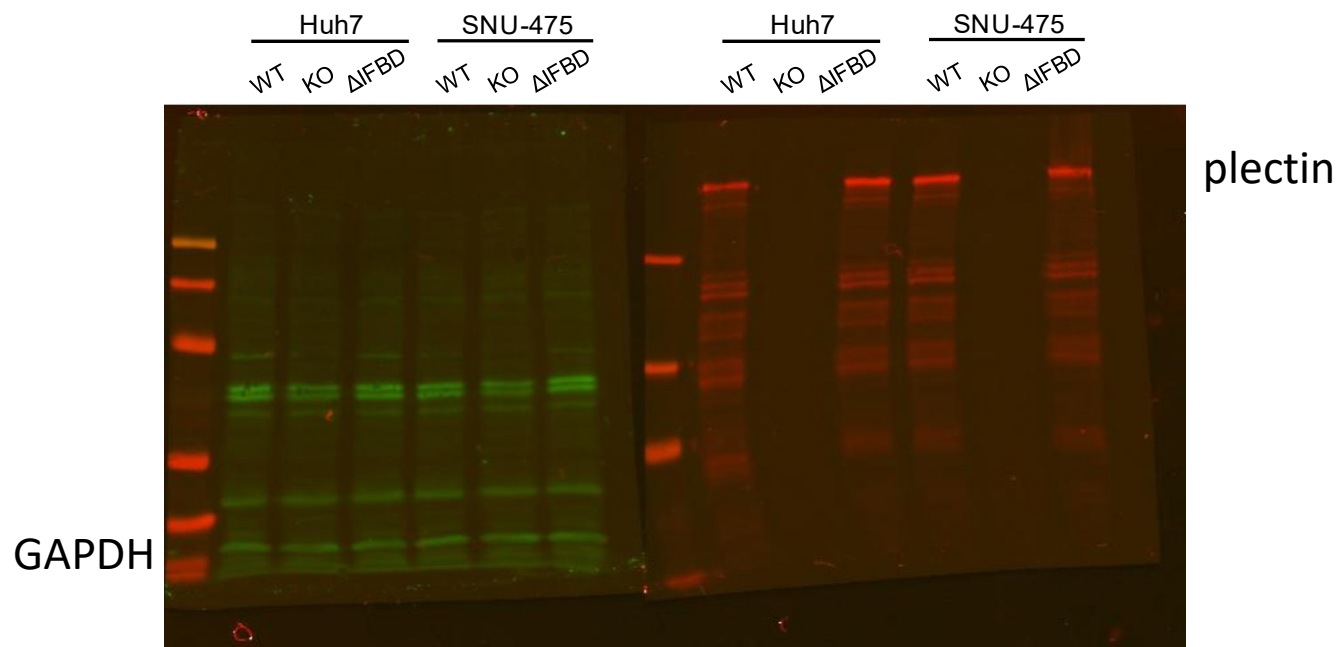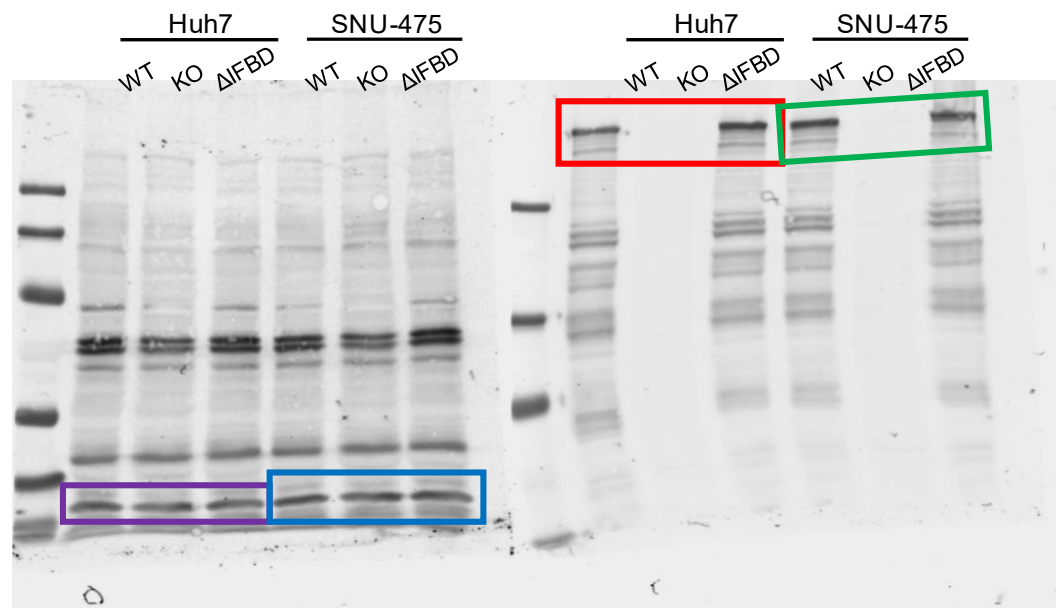

Supplement: Figure 2—figure supplement 1—source data 1. [file elife-102205-fig2-figsupp1-data1.pdf]

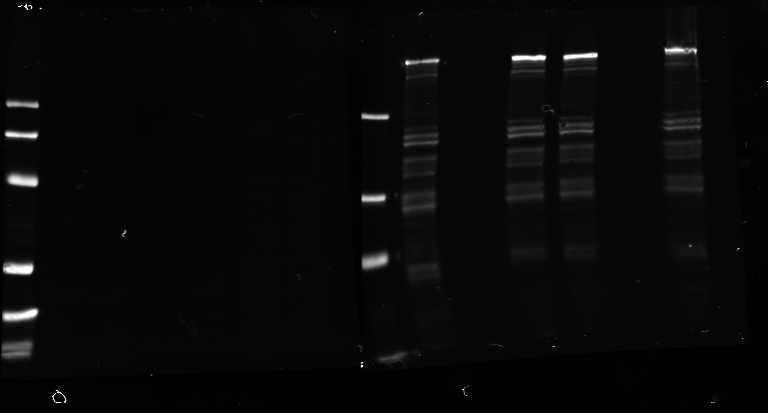

Supplement: Figure 2—figure supplement 1—source data 2. [file elife-102205-fig2-figsupp1-data2.zip › Figure 2-figure supplement 1-source data 1/240119_Huh_Snu_WT_KO_IFBD_gp21_GAPDH-700.tif]

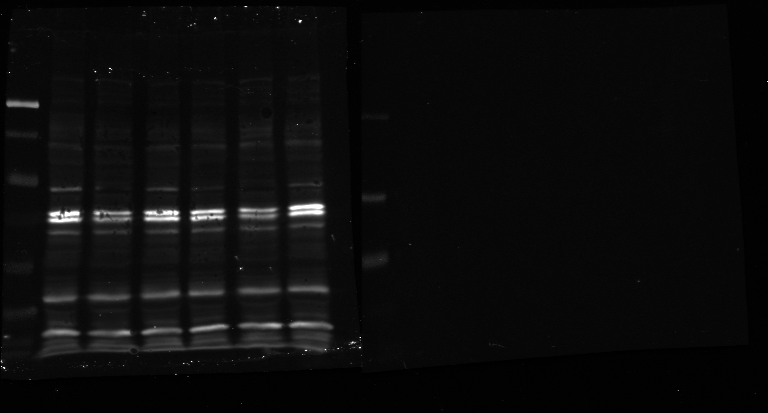

Supplement: Figure 2—figure supplement 1—source data 2. [file elife-102205-fig2-figsupp1-data2.zip › Figure 2-figure supplement 1-source data 1/240119_Huh_Snu_WT_KO_IFBD_gp21_GAPDH-800.tif]

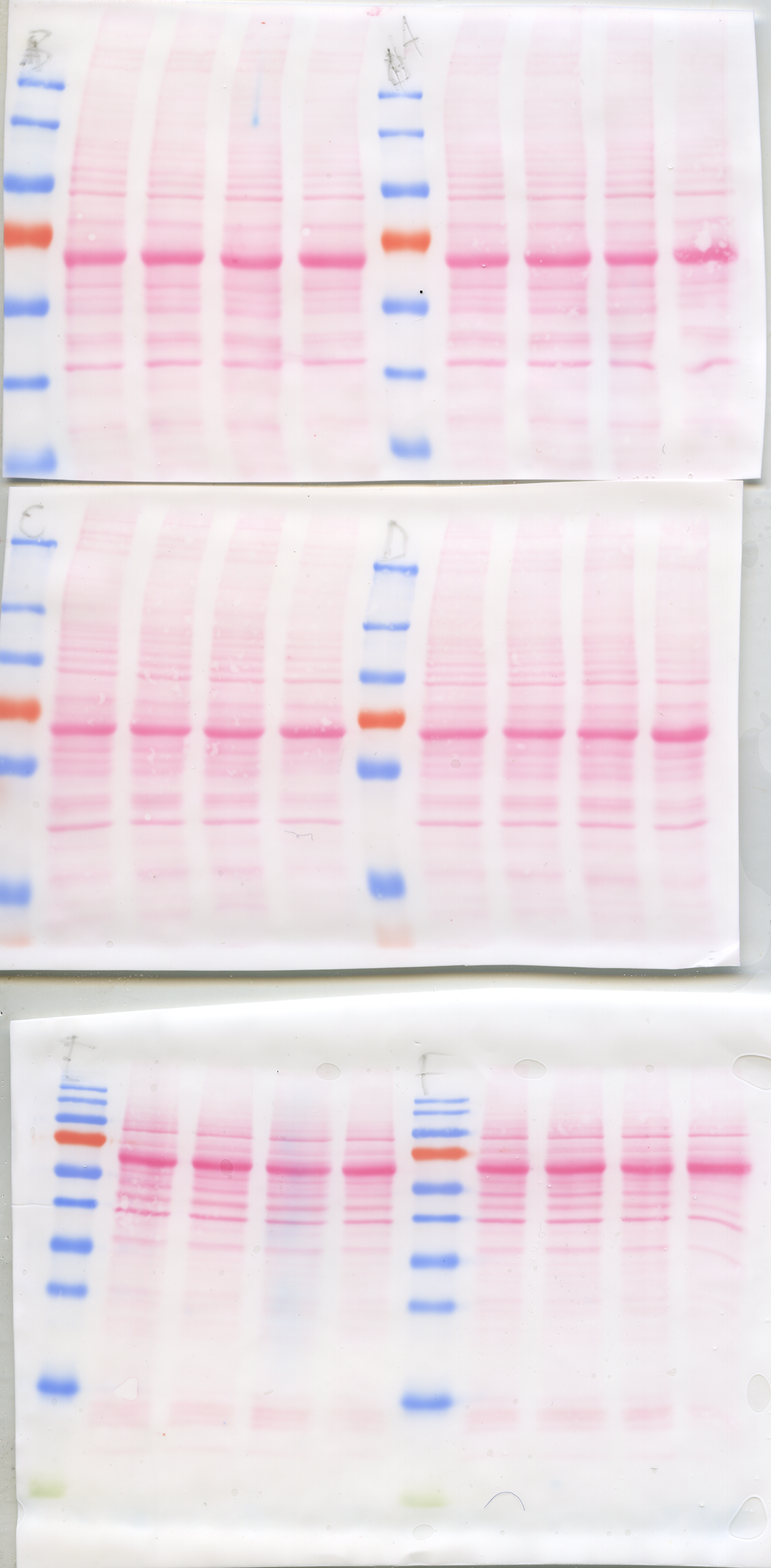

Supplement: Figure 3—source data 2. — Huh7 replicate 1 and SNU475 replicate 1–9. [file elife-102205-fig3-data2.zip › Figure 3-source data 2. Original files for western blot analysis displayed in Figure 3D and Figure 3-figure supplement 1C. Huh7 1st and SNU475/1st Snu475/221208_Snu475 1st WT KO IFBD WT+PST.tif]

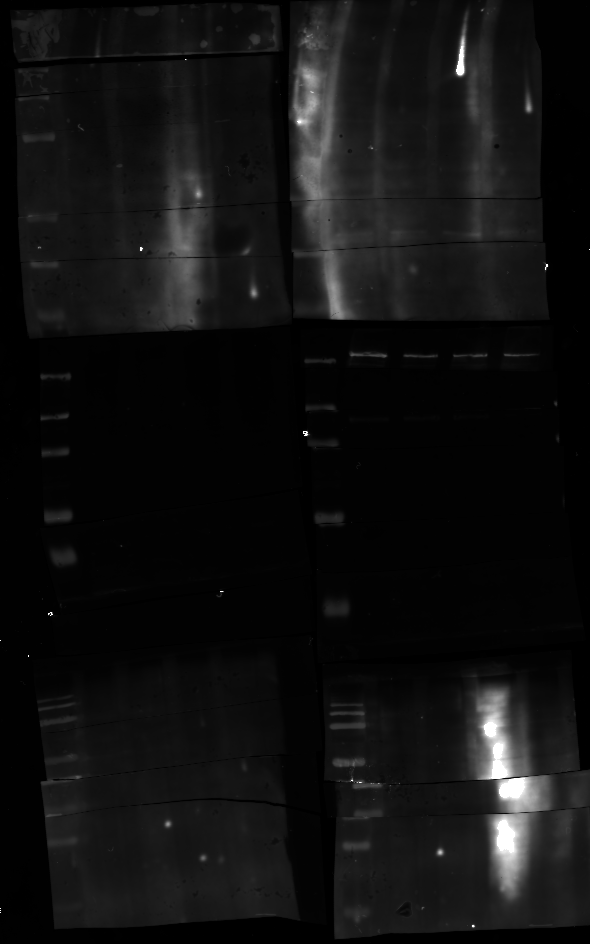

Supplement: Figure 3—source data 2. — Huh7 replicate 1 and SNU475 replicate 1–9. [file elife-102205-fig3-data2.zip › Figure 3-source data 2. Original files for western blot analysis displayed in Figure 3D and Figure 3-figure supplement 1C. Huh7 1st and SNU475/1st Snu475/221209-700.tif]

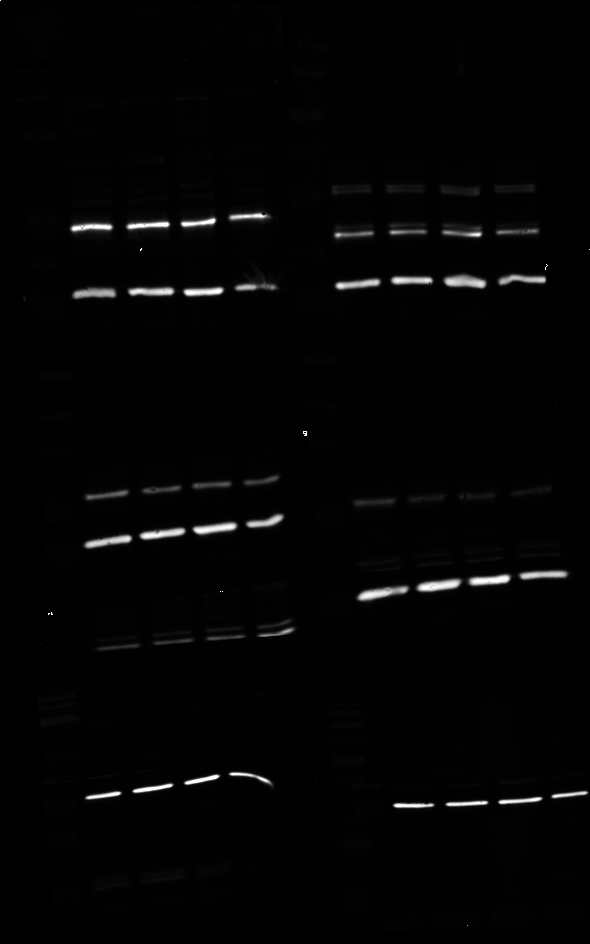

Supplement: Figure 3—source data 2. — Huh7 replicate 1 and SNU475 replicate 1–9. [file elife-102205-fig3-data2.zip › Figure 3-source data 2. Original files for western blot analysis displayed in Figure 3D and Figure 3-figure supplement 1C. Huh7 1st and SNU475/1st Snu475/221209-800.tif]

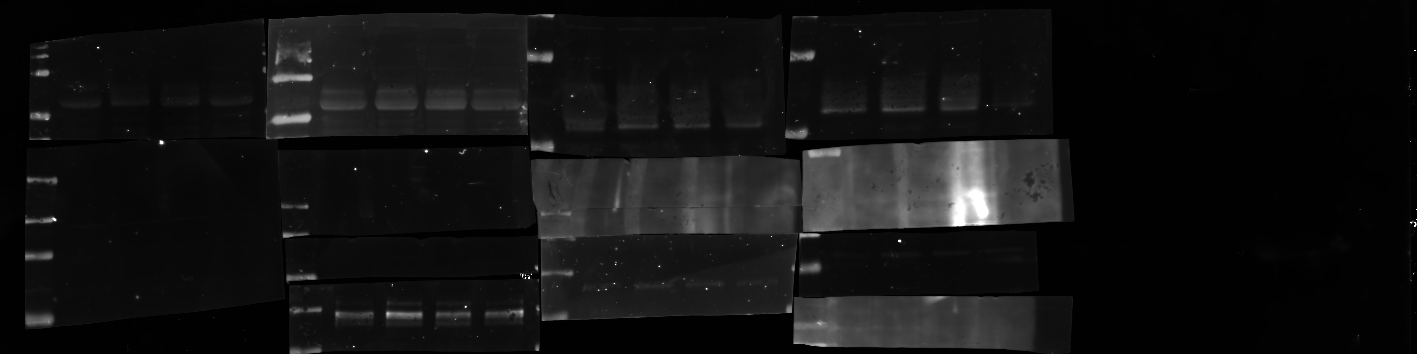

Supplement: Figure 3—source data 2. — Huh7 replicate 1 and SNU475 replicate 1–9. [file elife-102205-fig3-data2.zip › Figure 3-source data 2. Original files for western blot analysis displayed in Figure 3D and Figure 3-figure supplement 1C. Huh7 1st and SNU475/1st Snu475/230105-700.tif]

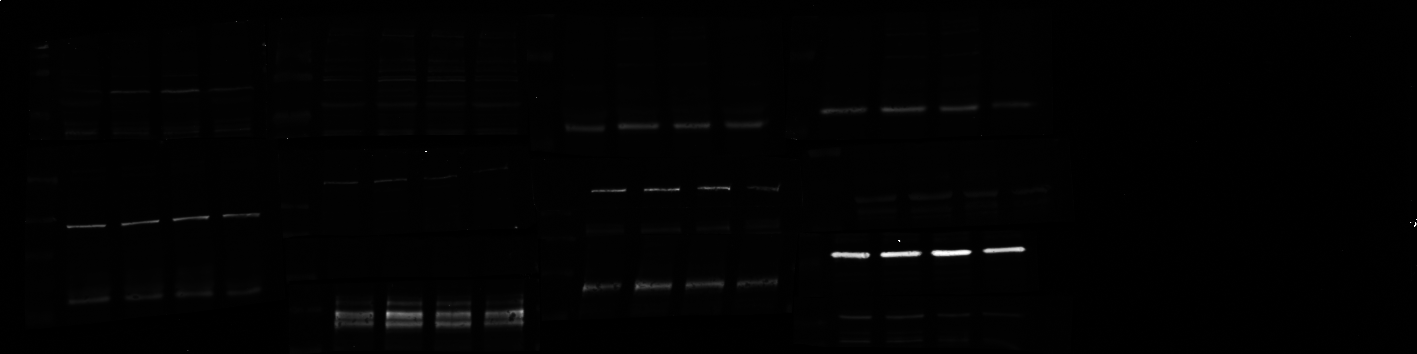

Supplement: Figure 3—source data 2. — Huh7 replicate 1 and SNU475 replicate 1–9. [file elife-102205-fig3-data2.zip › Figure 3-source data 2. Original files for western blot analysis displayed in Figure 3D and Figure 3-figure supplement 1C. Huh7 1st and SNU475/1st Snu475/230105-800.tif]

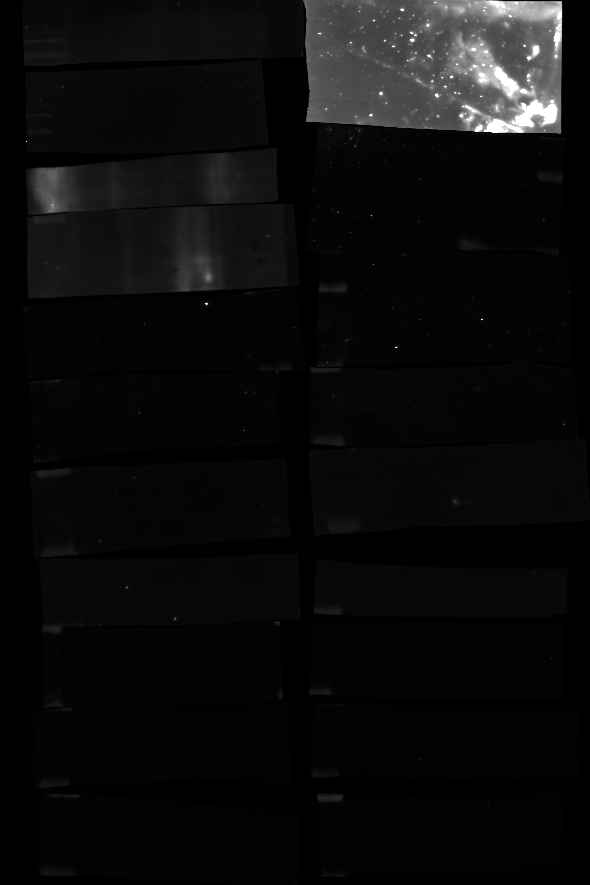

Supplement: Figure 3—source data 2. — Huh7 replicate 1 and SNU475 replicate 1–9. [file elife-102205-fig3-data2.zip › Figure 3-source data 2. Original files for western blot analysis displayed in Figure 3D and Figure 3-figure supplement 1C. Huh7 1st and SNU475/1st Snu475/230310-700.tif]

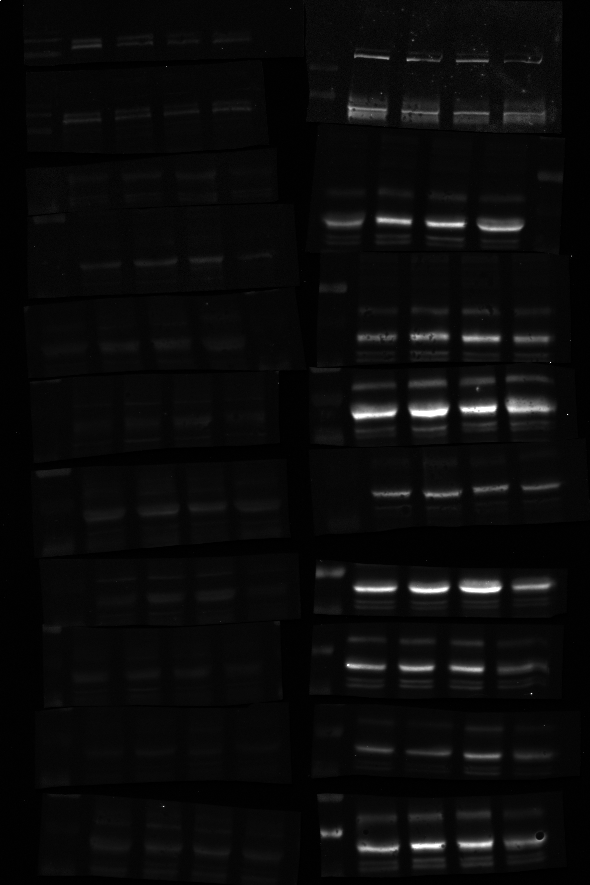

Supplement: Figure 3—source data 2. — Huh7 replicate 1 and SNU475 replicate 1–9. [file elife-102205-fig3-data2.zip › Figure 3-source data 2. Original files for western blot analysis displayed in Figure 3D and Figure 3-figure supplement 1C. Huh7 1st and SNU475/1st Snu475/230310-800.tif]

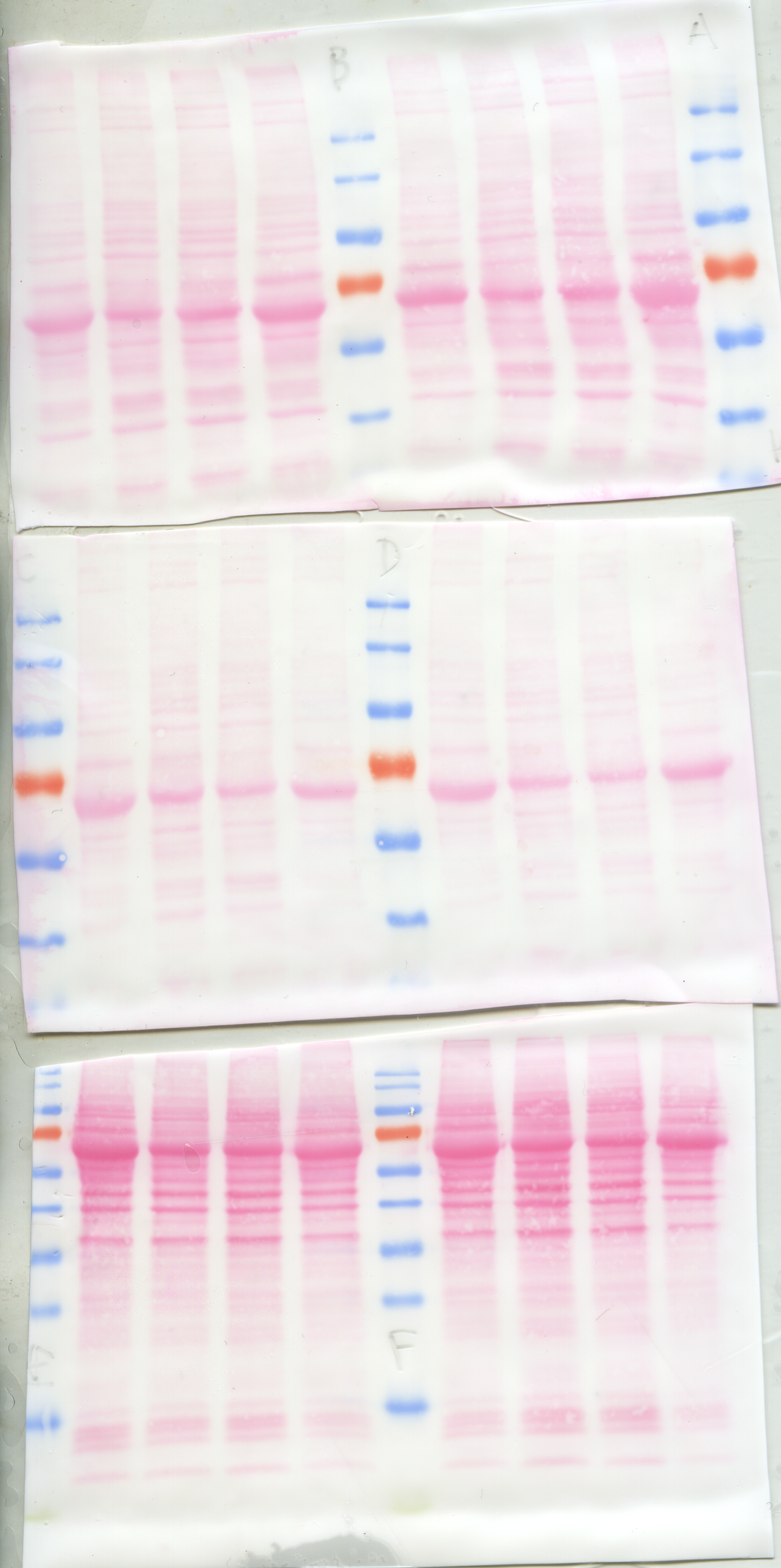

Supplement: Figure 3—source data 2. — Huh7 replicate 1 and SNU475 replicate 1–9. [file elife-102205-fig3-data2.zip › Figure 3-source data 2. Original files for western blot analysis displayed in Figure 3D and Figure 3-figure supplement 1C. Huh7 1st and SNU475/2nd + 3rd Snu475/221215 Snu475 2nd WT KO IFBD WT+PST.tif]

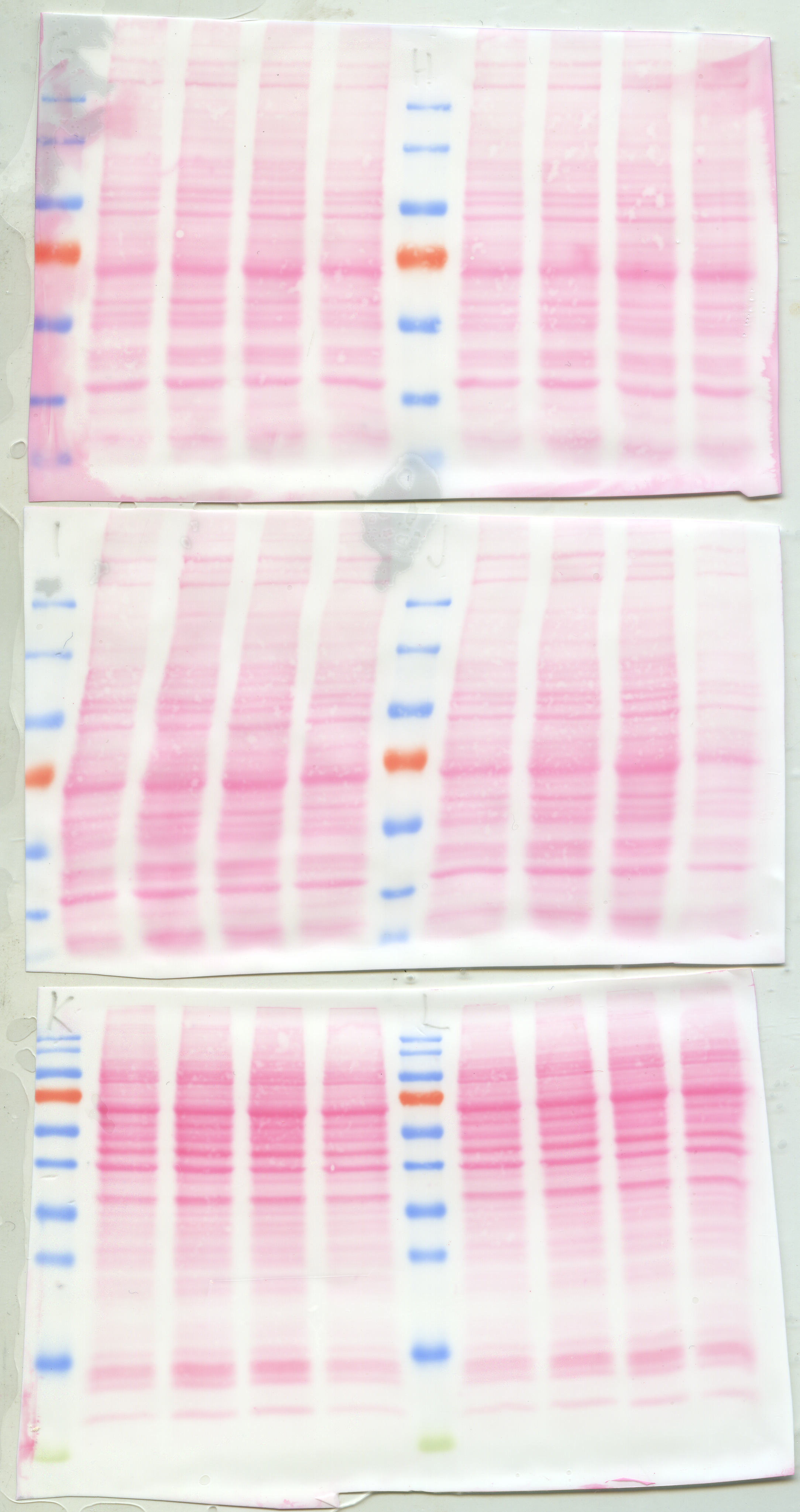

Supplement: Figure 3—source data 2. — Huh7 replicate 1 and SNU475 replicate 1–9. [file elife-102205-fig3-data2.zip › Figure 3-source data 2. Original files for western blot analysis displayed in Figure 3D and Figure 3-figure supplement 1C. Huh7 1st and SNU475/2nd + 3rd Snu475/221215 Snu475 3rd WT KO IFBD WT+PST.tif]

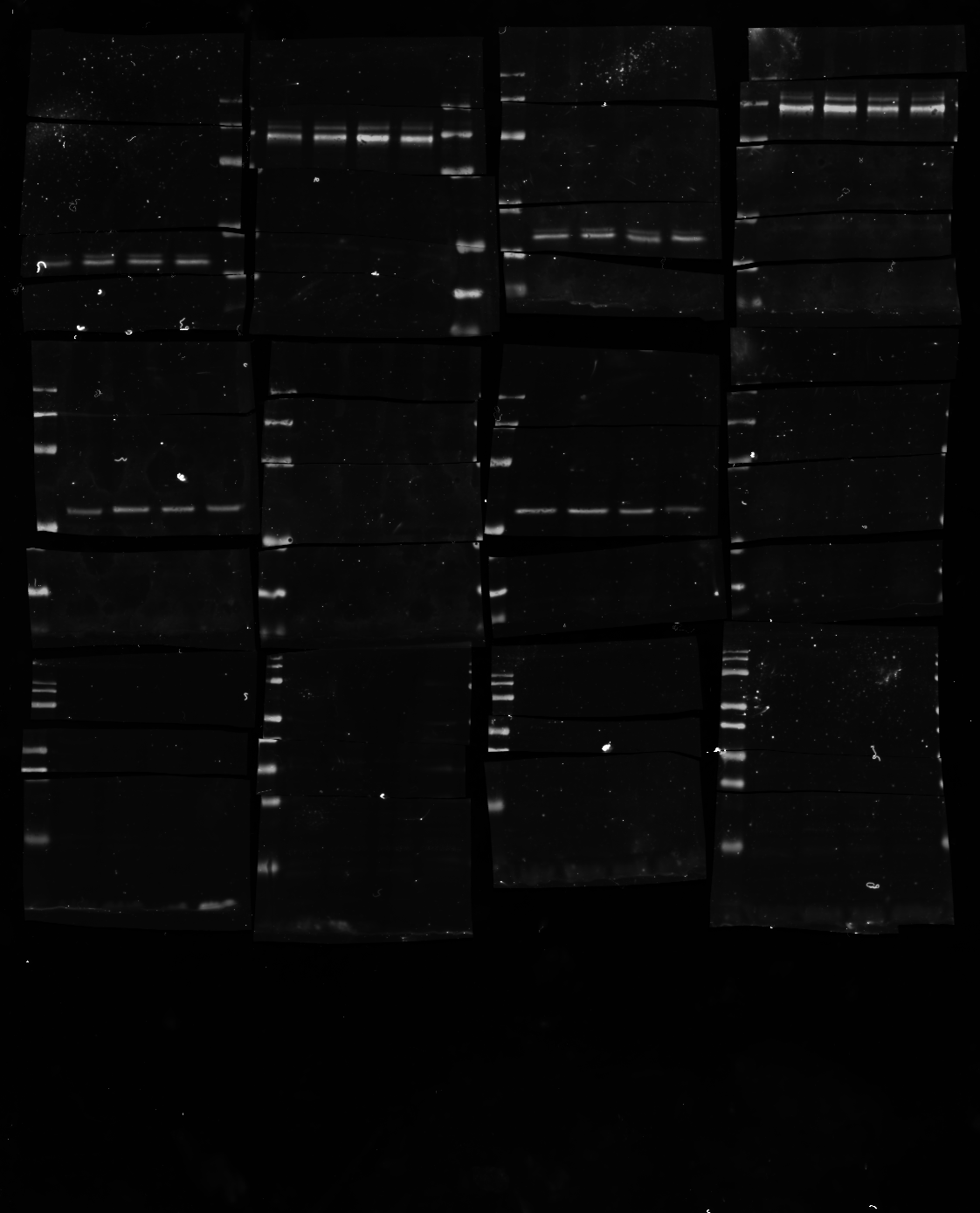

Supplement: Figure 3—source data 2. — Huh7 replicate 1 and SNU475 replicate 1–9. [file elife-102205-fig3-data2.zip › Figure 3-source data 2. Original files for western blot analysis displayed in Figure 3D and Figure 3-figure supplement 1C. Huh7 1st and SNU475/2nd + 3rd Snu475/221216-700.tiff]

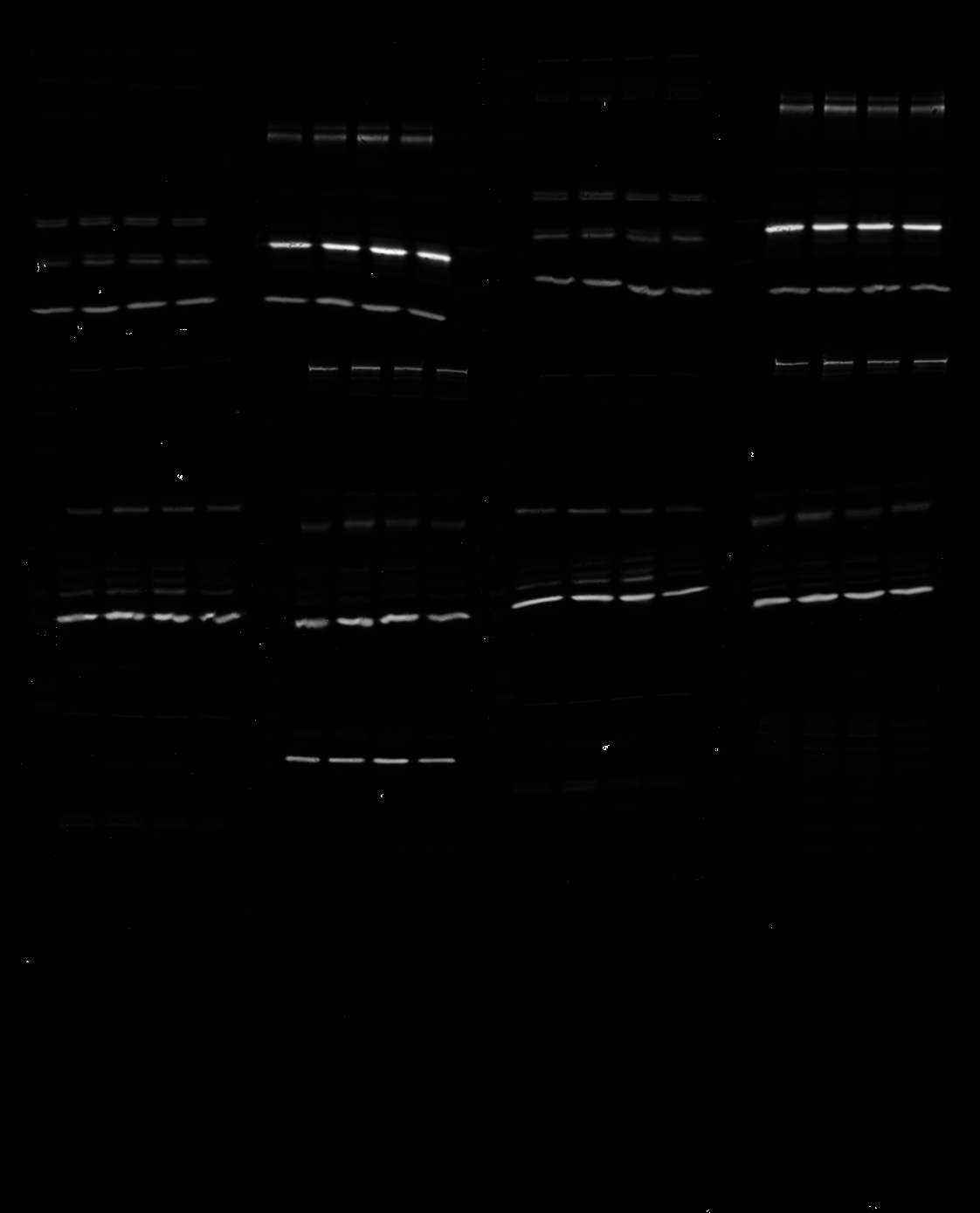

Supplement: Figure 3—source data 2. — Huh7 replicate 1 and SNU475 replicate 1–9. [file elife-102205-fig3-data2.zip › Figure 3-source data 2. Original files for western blot analysis displayed in Figure 3D and Figure 3-figure supplement 1C. Huh7 1st and SNU475/2nd + 3rd Snu475/221216-800.tiff]

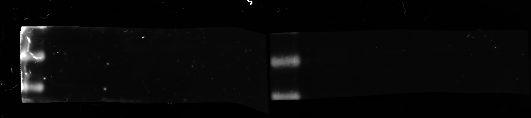

Supplement: Figure 3—source data 2. — Huh7 replicate 1 and SNU475 replicate 1–9. [file elife-102205-fig3-data2.zip › Figure 3-source data 2. Original files for western blot analysis displayed in Figure 3D and Figure 3-figure supplement 1C. Huh7 1st and SNU475/2nd + 3rd Snu475/221216b-700.tif]

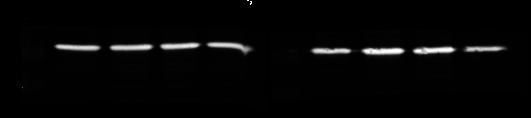

Supplement: Figure 3—source data 2. — Huh7 replicate 1 and SNU475 replicate 1–9. [file elife-102205-fig3-data2.zip › Figure 3-source data 2. Original files for western blot analysis displayed in Figure 3D and Figure 3-figure supplement 1C. Huh7 1st and SNU475/2nd + 3rd Snu475/221216b-800.tif]

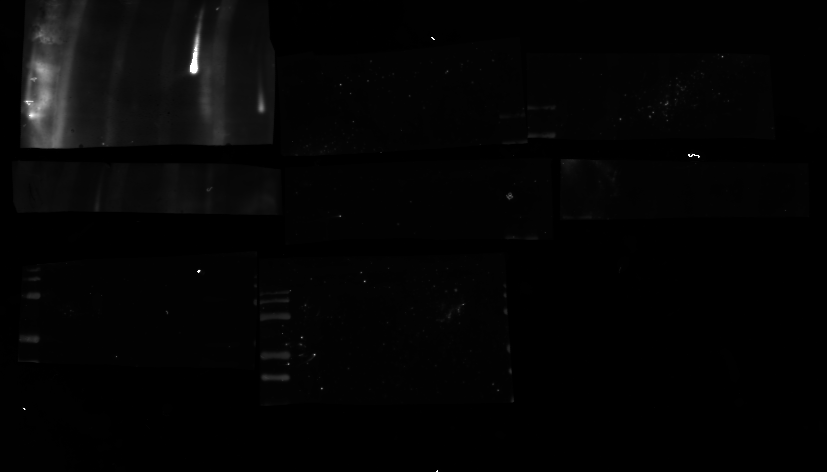

Supplement: Figure 3—source data 2. — Huh7 replicate 1 and SNU475 replicate 1–9. [file elife-102205-fig3-data2.zip › Figure 3-source data 2. Original files for western blot analysis displayed in Figure 3D and Figure 3-figure supplement 1C. Huh7 1st and SNU475/2nd + 3rd Snu475/221217-700.tiff]

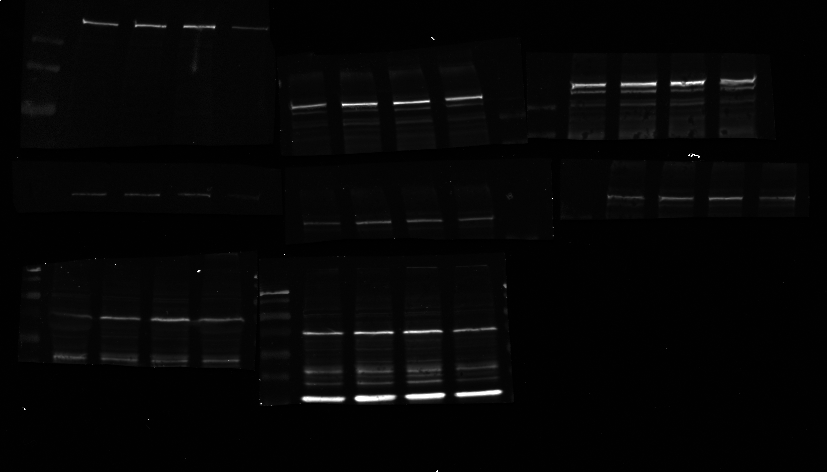

Supplement: Figure 3—source data 2. — Huh7 replicate 1 and SNU475 replicate 1–9. [file elife-102205-fig3-data2.zip › Figure 3-source data 2. Original files for western blot analysis displayed in Figure 3D and Figure 3-figure supplement 1C. Huh7 1st and SNU475/2nd + 3rd Snu475/221217-800.tiff]

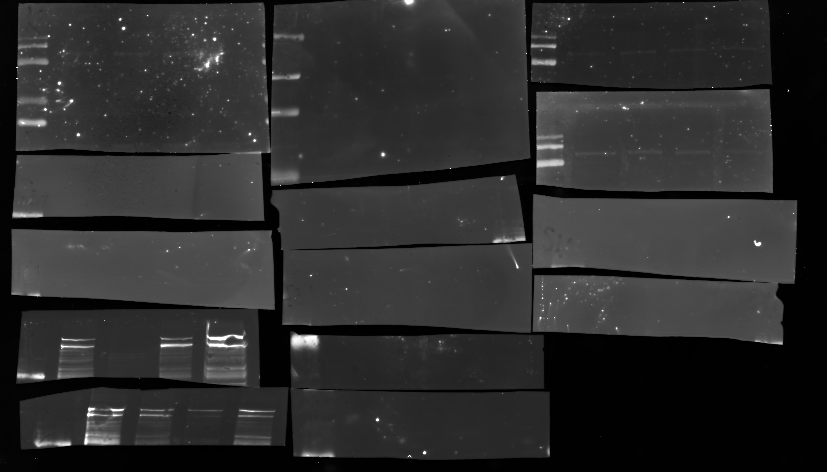

Supplement: Figure 3—source data 2. — Huh7 replicate 1 and SNU475 replicate 1–9. [file elife-102205-fig3-data2.zip › Figure 3-source data 2. Original files for western blot analysis displayed in Figure 3D and Figure 3-figure supplement 1C. Huh7 1st and SNU475/2nd + 3rd Snu475/230126-700.tif]

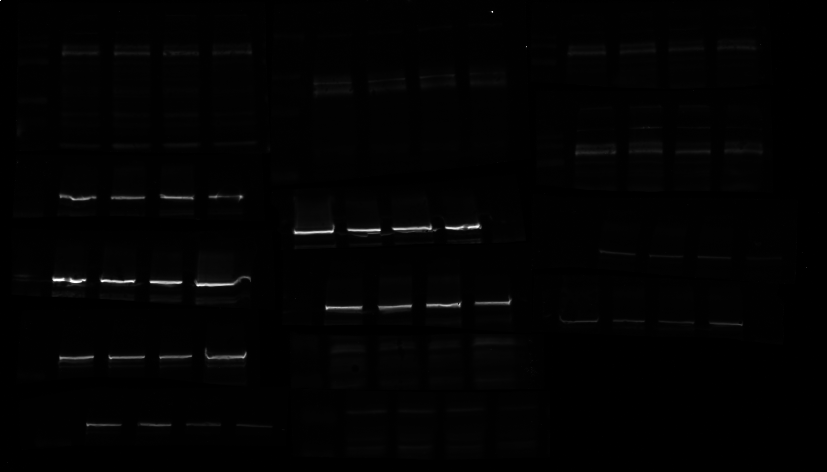

Supplement: Figure 3—source data 2. — Huh7 replicate 1 and SNU475 replicate 1–9. [file elife-102205-fig3-data2.zip › Figure 3-source data 2. Original files for western blot analysis displayed in Figure 3D and Figure 3-figure supplement 1C. Huh7 1st and SNU475/2nd + 3rd Snu475/230126-800.tif]

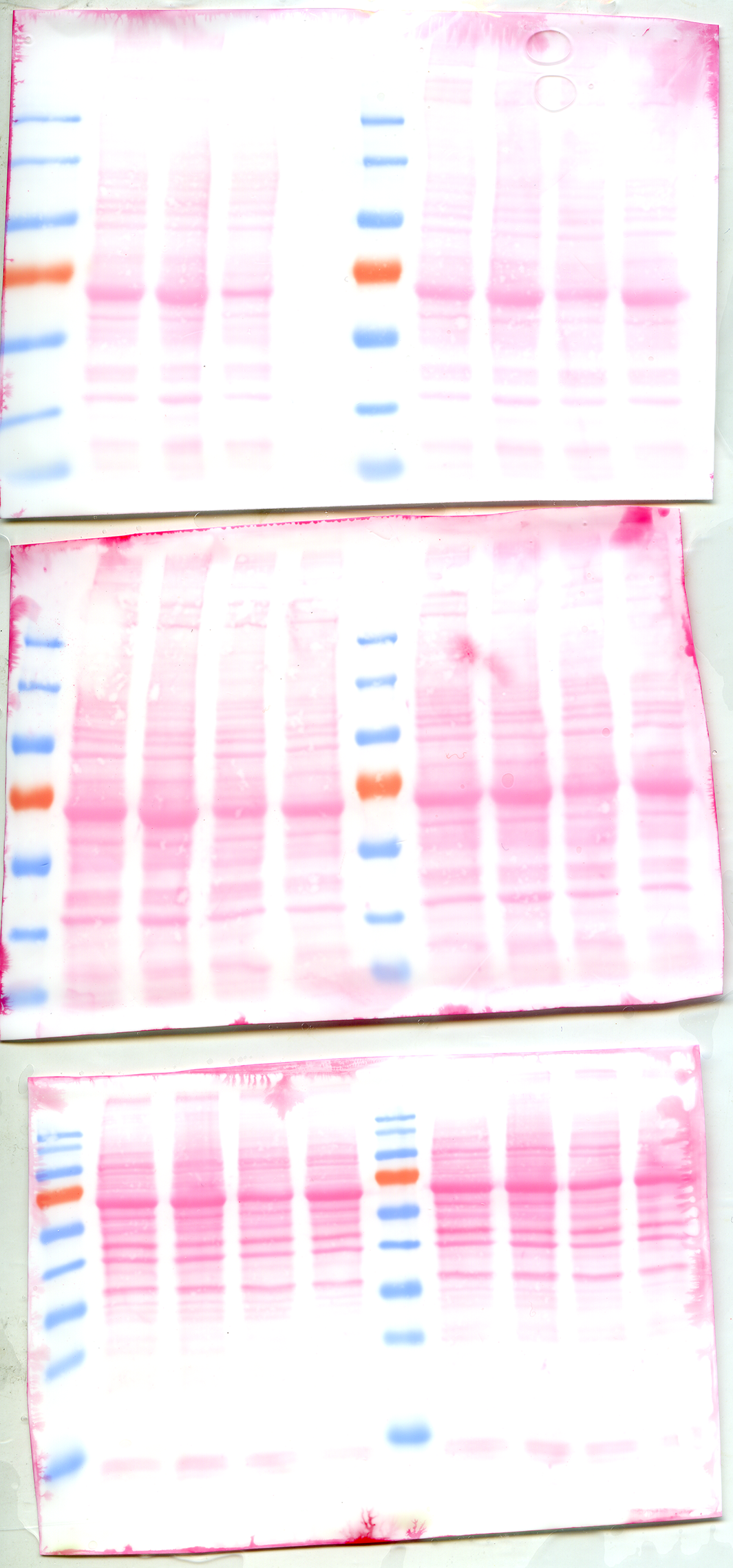

Supplement: Figure 3—source data 2. — Huh7 replicate 1 and SNU475 replicate 1–9. [file elife-102205-fig3-data2.zip › Figure 3-source data 2. Original files for western blot analysis displayed in Figure 3D and Figure 3-figure supplement 1C. Huh7 1st and SNU475/4th + 5th Snu475/221220 Snu475 4th WT KO IFBD WT+PST A-F.tif]

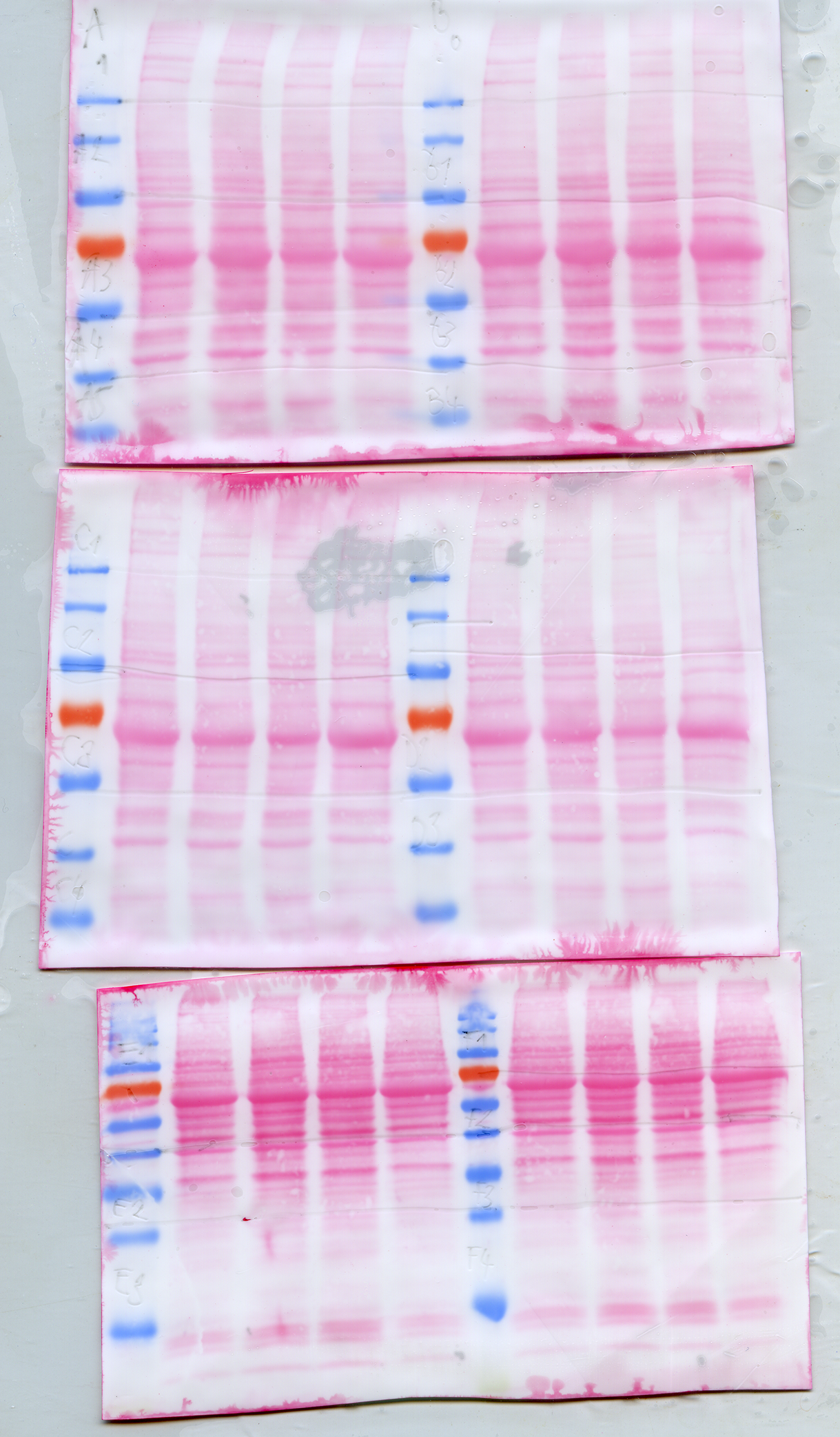

Supplement: Figure 3—source data 2. — Huh7 replicate 1 and SNU475 replicate 1–9. [file elife-102205-fig3-data2.zip › Figure 3-source data 2. Original files for western blot analysis displayed in Figure 3D and Figure 3-figure supplement 1C. Huh7 1st and SNU475/4th + 5th Snu475/221220 Snu475 5th WT KO IFBD WT+PST G-L marked.tif]

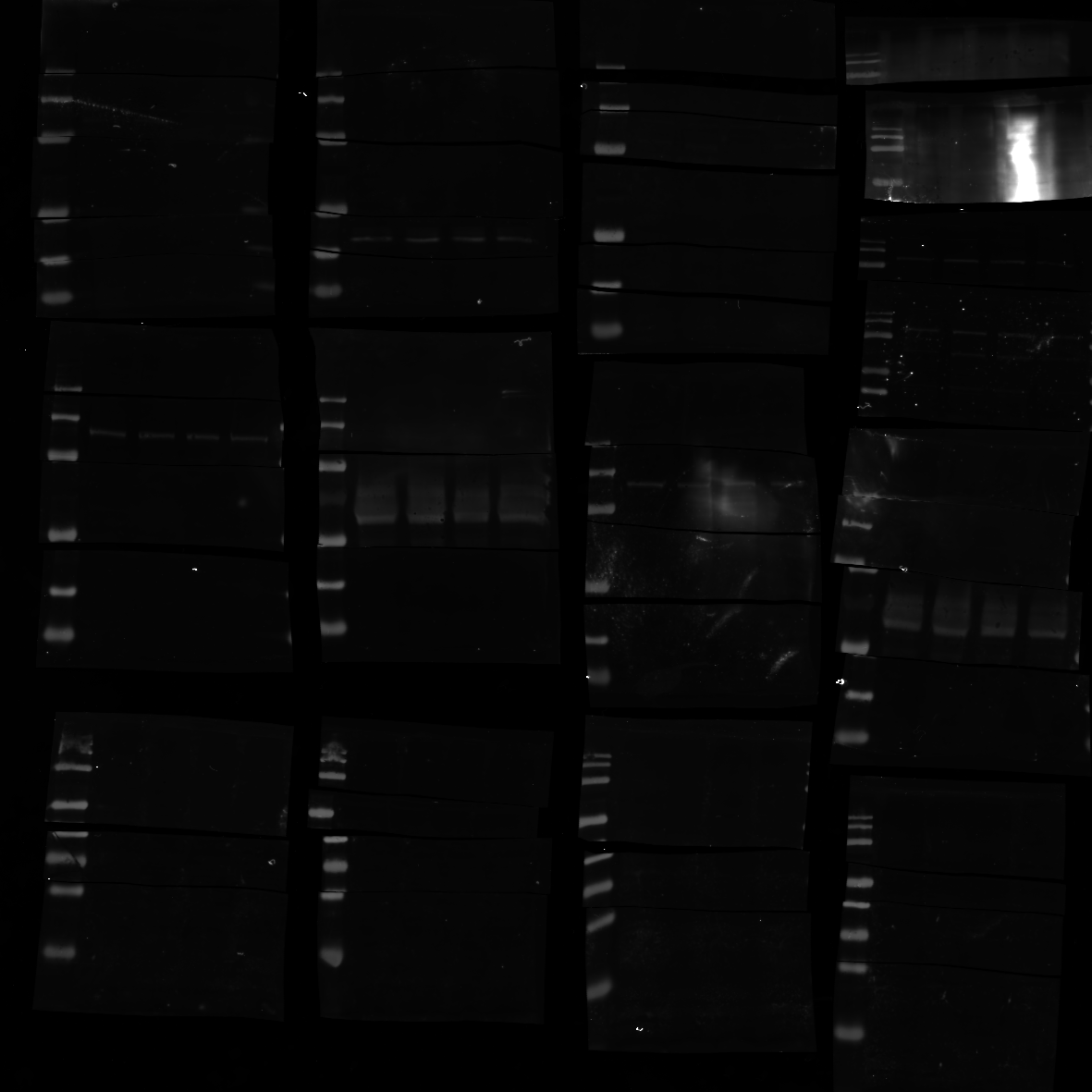

Supplement: Figure 3—source data 2. — Huh7 replicate 1 and SNU475 replicate 1–9. [file elife-102205-fig3-data2.zip › Figure 3-source data 2. Original files for western blot analysis displayed in Figure 3D and Figure 3-figure supplement 1C. Huh7 1st and SNU475/4th + 5th Snu475/221221-700.tif]

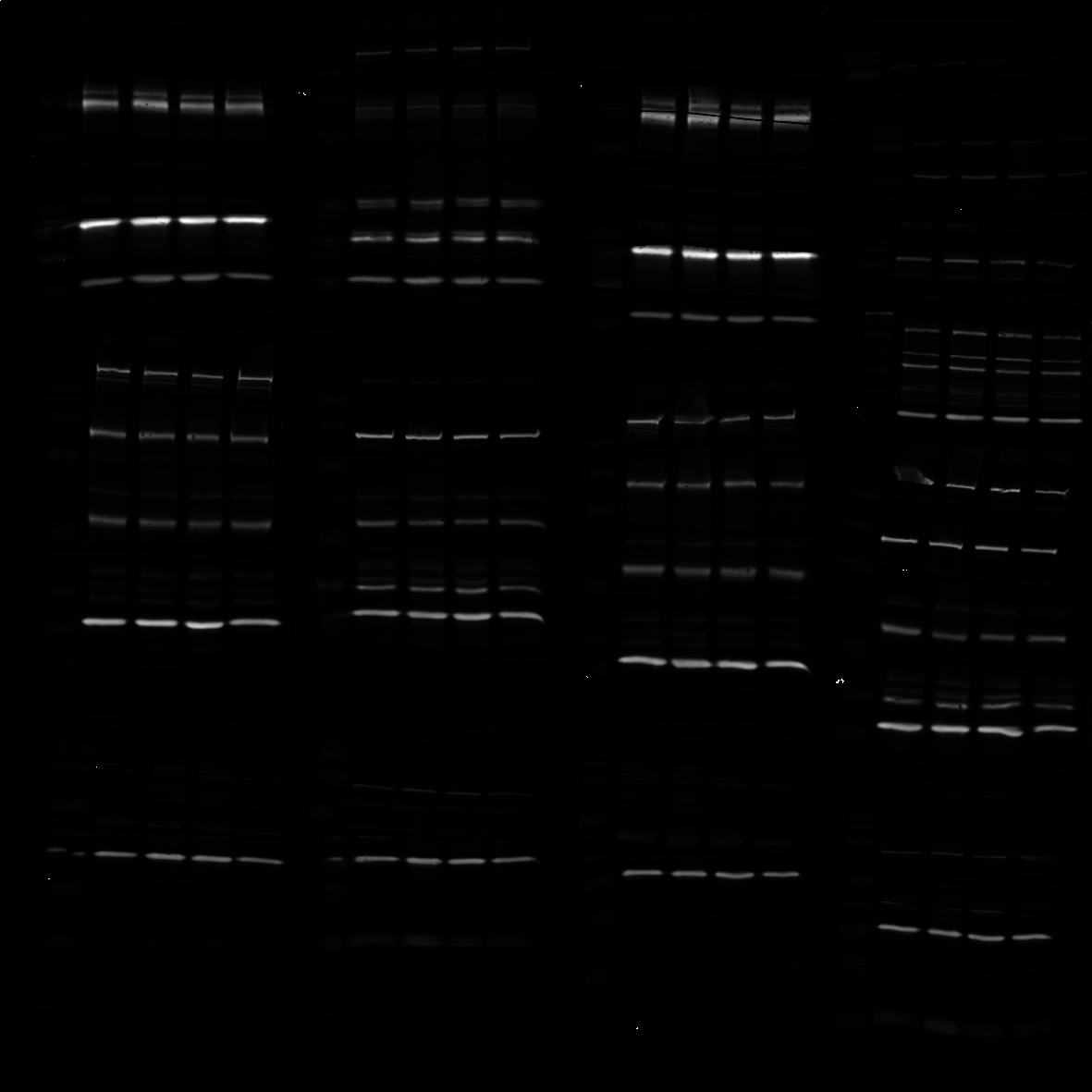

Supplement: Figure 3—source data 2. — Huh7 replicate 1 and SNU475 replicate 1–9. [file elife-102205-fig3-data2.zip › Figure 3-source data 2. Original files for western blot analysis displayed in Figure 3D and Figure 3-figure supplement 1C. Huh7 1st and SNU475/4th + 5th Snu475/221221-800.tif]

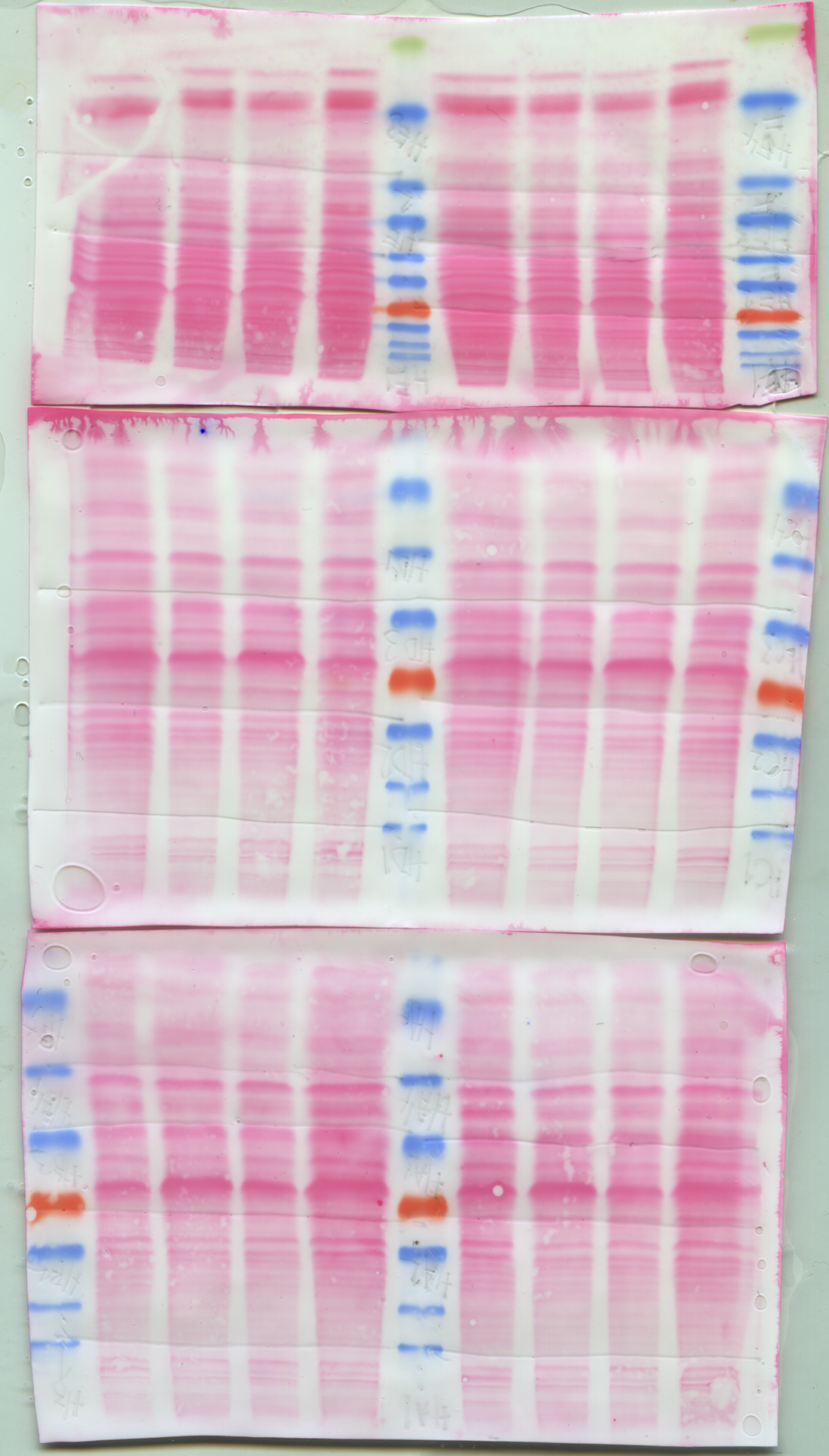

Supplement: Figure 3—source data 2. — Huh7 replicate 1 and SNU475 replicate 1–9. [file elife-102205-fig3-data2.zip › Figure 3-source data 2. Original files for western blot analysis displayed in Figure 3D and Figure 3-figure supplement 1C. Huh7 1st and SNU475/6th Snu475 + 1st Huh7/230111 Huh7 1st replicate marker WT KO IFBD WT+PST marked.tif]

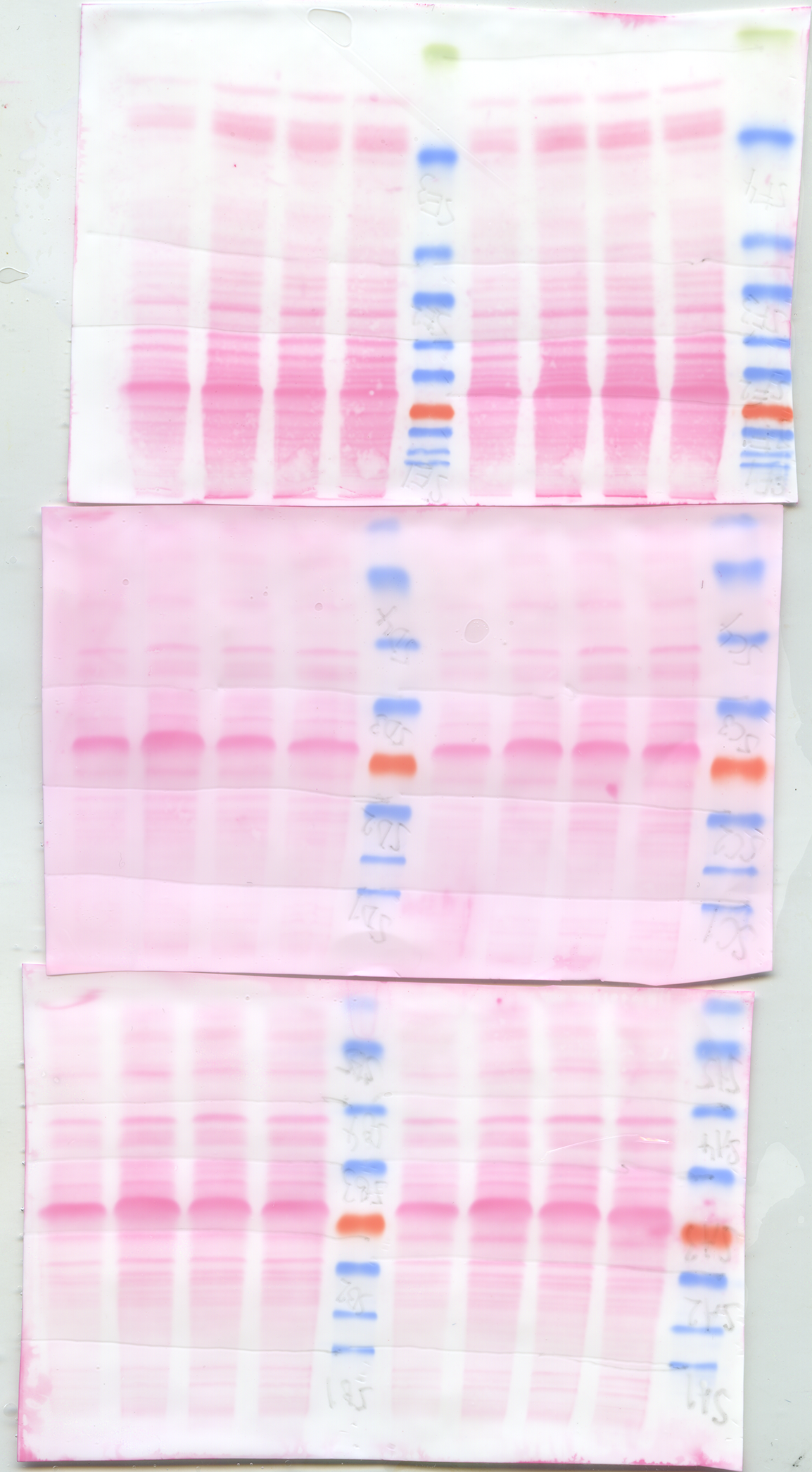

Supplement: Figure 3—source data 2. — Huh7 replicate 1 and SNU475 replicate 1–9. [file elife-102205-fig3-data2.zip › Figure 3-source data 2. Original files for western blot analysis displayed in Figure 3D and Figure 3-figure supplement 1C. Huh7 1st and SNU475/6th Snu475 + 1st Huh7/230111 Snu475 6th replicate marker WT KO IFBD WT+PST marked.tif]

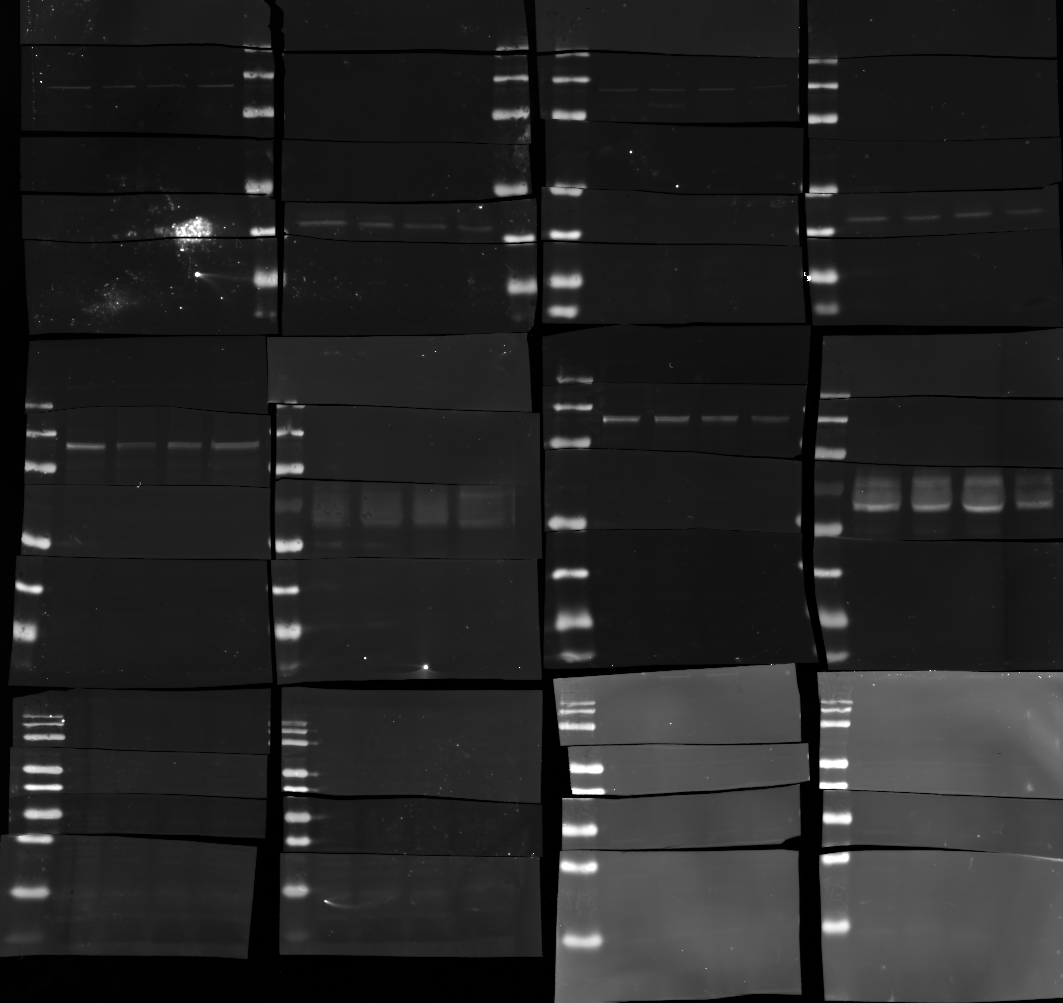

Supplement: Figure 3—source data 2. — Huh7 replicate 1 and SNU475 replicate 1–9. [file elife-102205-fig3-data2.zip › Figure 3-source data 2. Original files for western blot analysis displayed in Figure 3D and Figure 3-figure supplement 1C. Huh7 1st and SNU475/6th Snu475 + 1st Huh7/230125-700.tif]

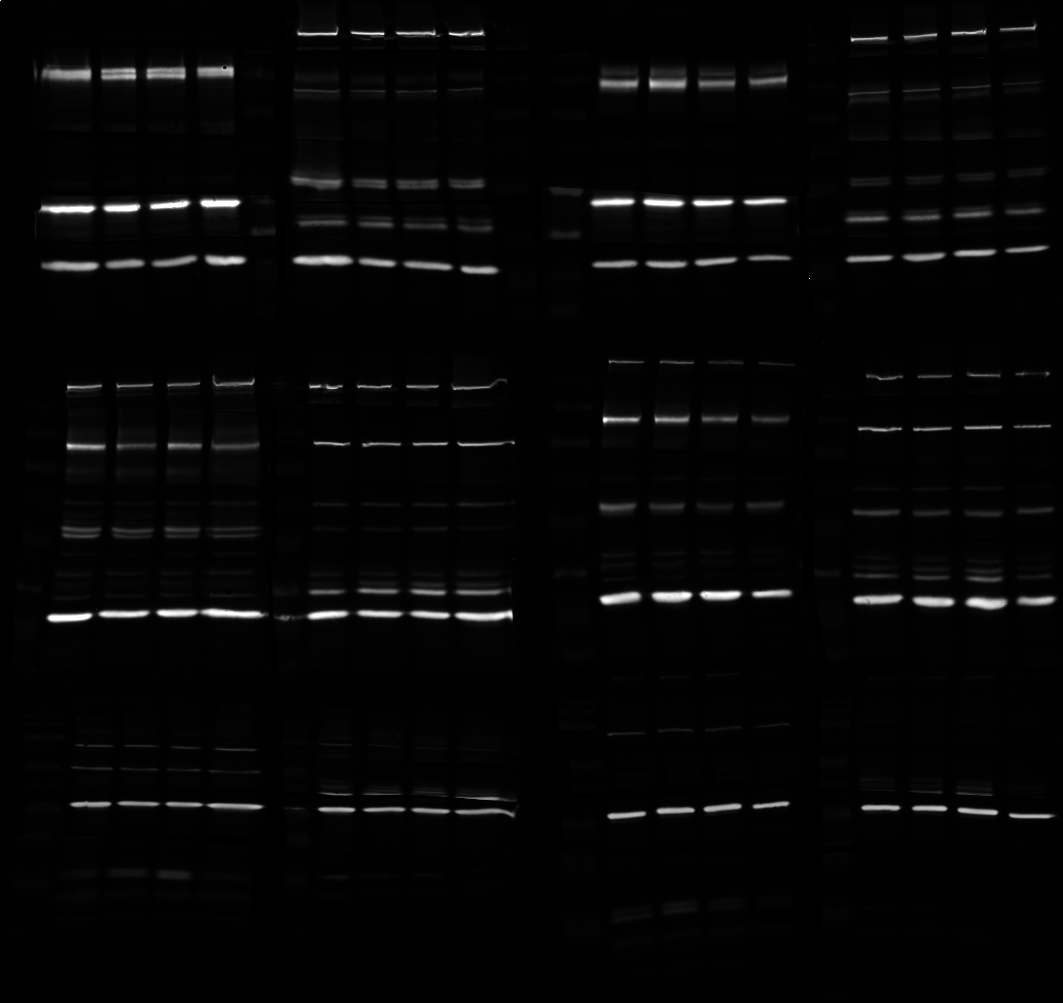

Supplement: Figure 3—source data 2. — Huh7 replicate 1 and SNU475 replicate 1–9. [file elife-102205-fig3-data2.zip › Figure 3-source data 2. Original files for western blot analysis displayed in Figure 3D and Figure 3-figure supplement 1C. Huh7 1st and SNU475/6th Snu475 + 1st Huh7/230125-800.tif]

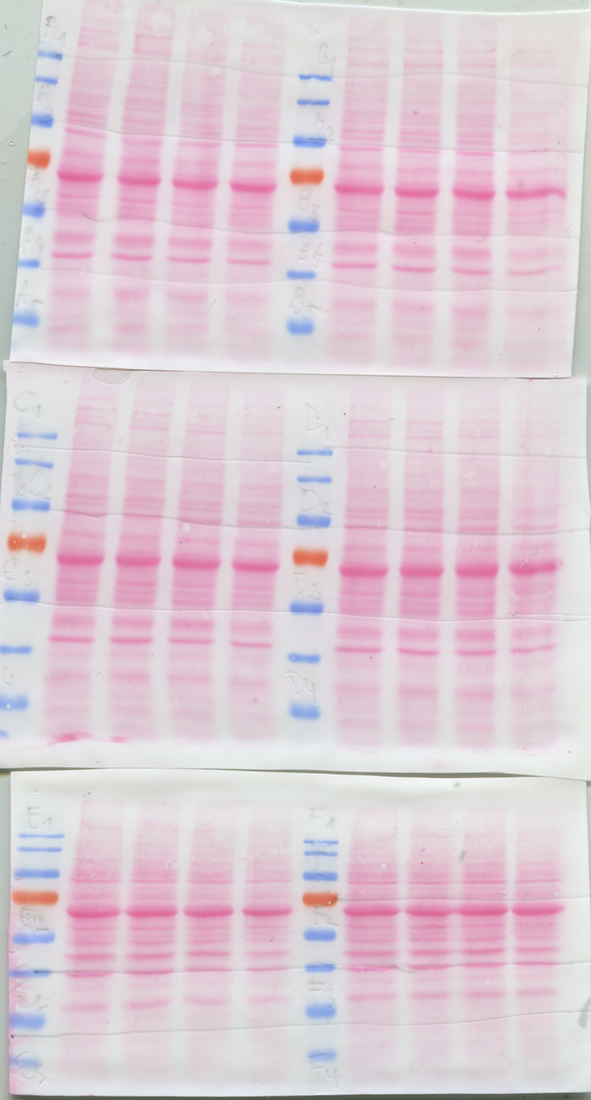

Supplement: Figure 3—source data 2. — Huh7 replicate 1 and SNU475 replicate 1–9. [file elife-102205-fig3-data2.zip › Figure 3-source data 2. Original files for western blot analysis displayed in Figure 3D and Figure 3-figure supplement 1C. Huh7 1st and SNU475/7th + 8th/230223_Snu475 7th AB CD EF Ponceau marked.tif]

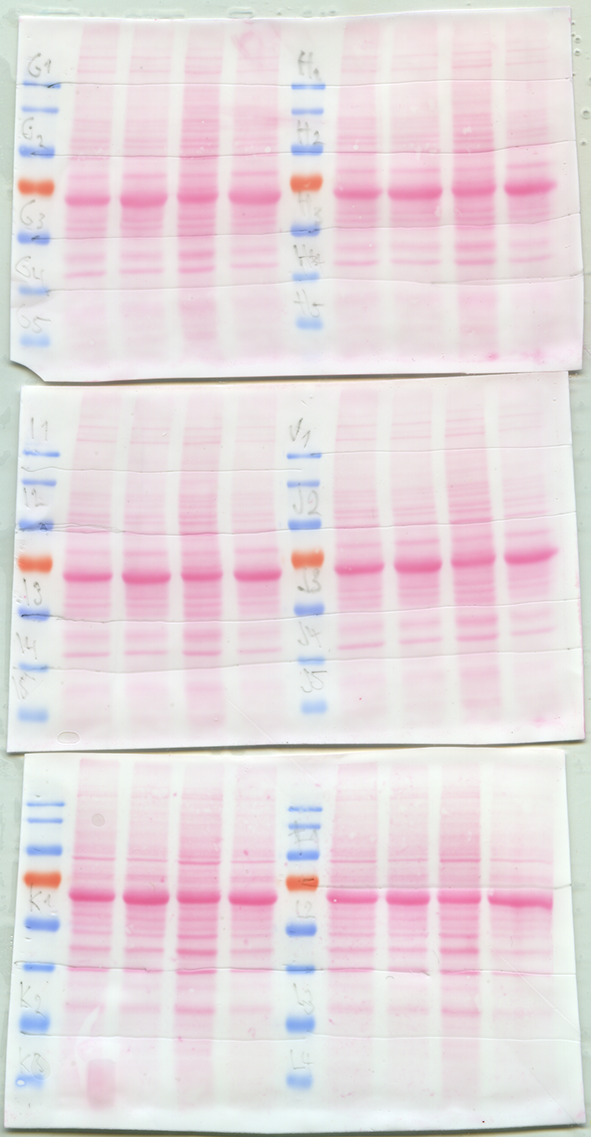

Supplement: Figure 3—source data 2. — Huh7 replicate 1 and SNU475 replicate 1–9. [file elife-102205-fig3-data2.zip › Figure 3-source data 2. Original files for western blot analysis displayed in Figure 3D and Figure 3-figure supplement 1C. Huh7 1st and SNU475/7th + 8th/230223_Snu475 8th GH IJ KL Ponceau marked.tif]

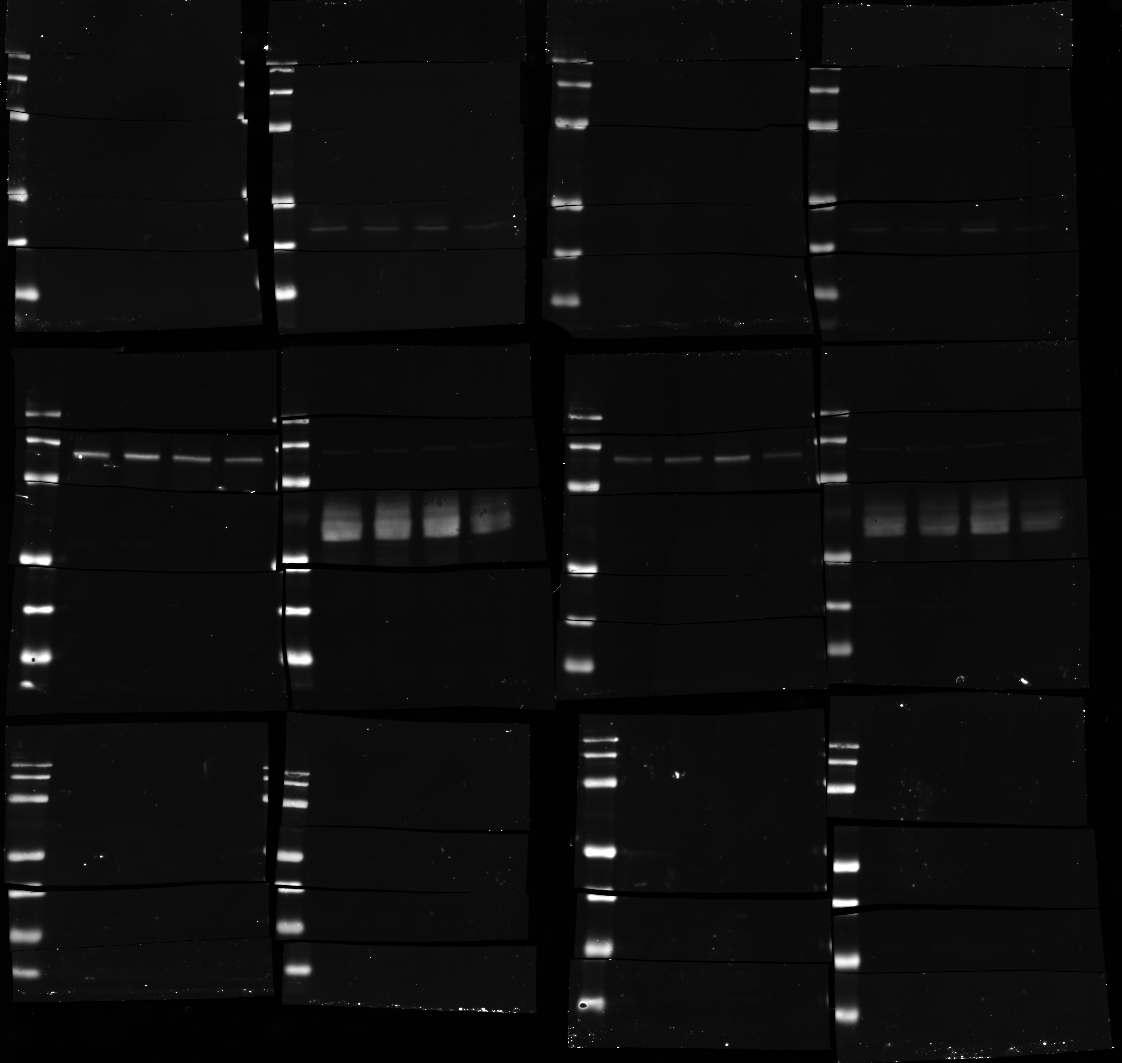

Supplement: Figure 3—source data 2. — Huh7 replicate 1 and SNU475 replicate 1–9. [file elife-102205-fig3-data2.zip › Figure 3-source data 2. Original files for western blot analysis displayed in Figure 3D and Figure 3-figure supplement 1C. Huh7 1st and SNU475/7th + 8th/230224-700.tif]

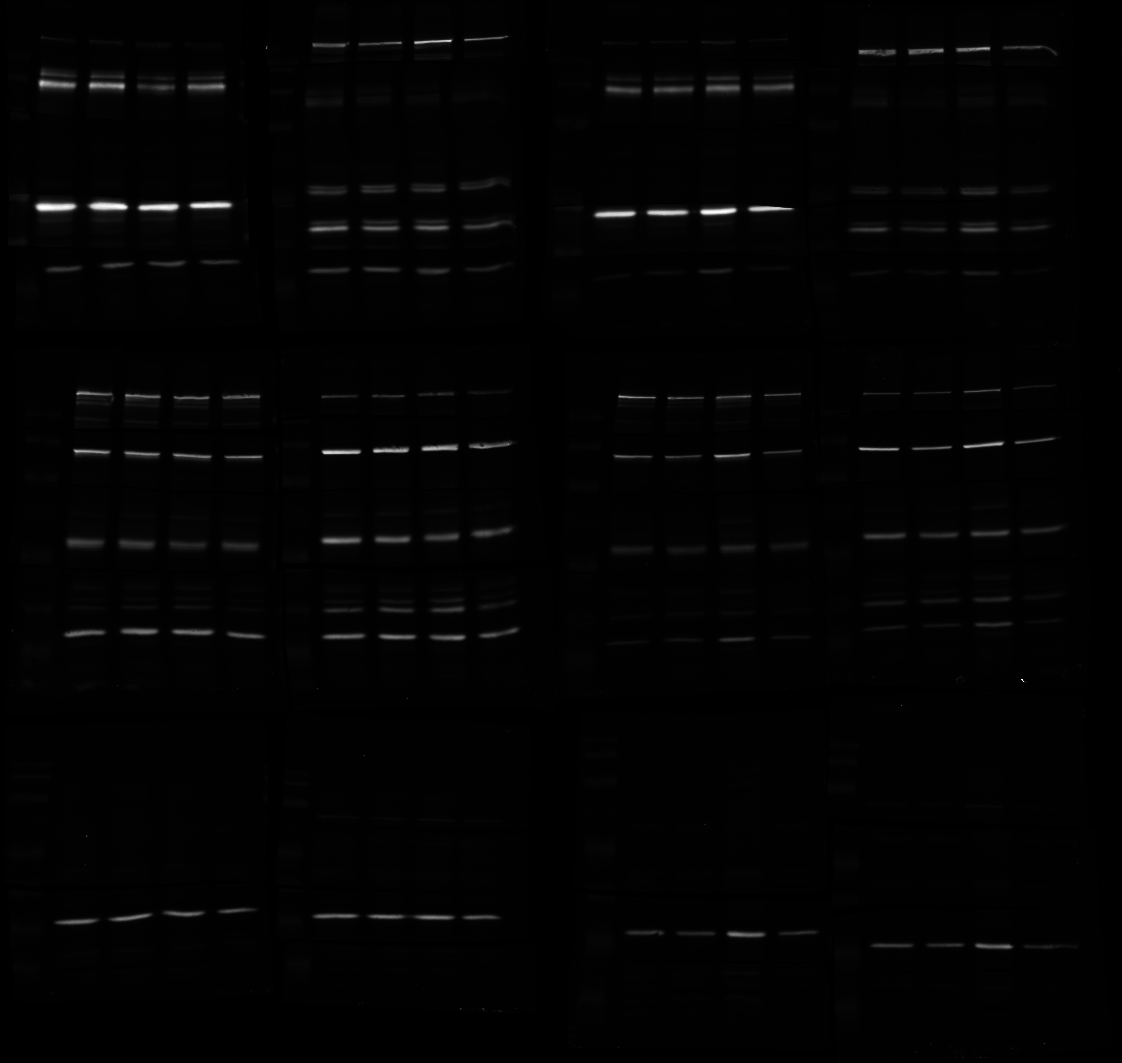

Supplement: Figure 3—source data 2. — Huh7 replicate 1 and SNU475 replicate 1–9. [file elife-102205-fig3-data2.zip › Figure 3-source data 2. Original files for western blot analysis displayed in Figure 3D and Figure 3-figure supplement 1C. Huh7 1st and SNU475/7th + 8th/230224-800.tif]

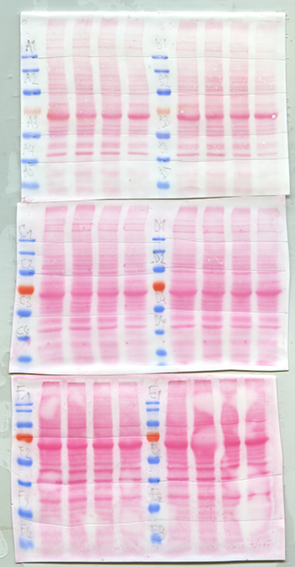

Supplement: Figure 3—source data 2. — Huh7 replicate 1 and SNU475 replicate 1–9. [file elife-102205-fig3-data2.zip › Figure 3-source data 2. Original files for western blot analysis displayed in Figure 3D and Figure 3-figure supplement 1C. Huh7 1st and SNU475/9th/230303_Snu475 9th WT KO IFBD WT+PST Ponceau marked.tif]

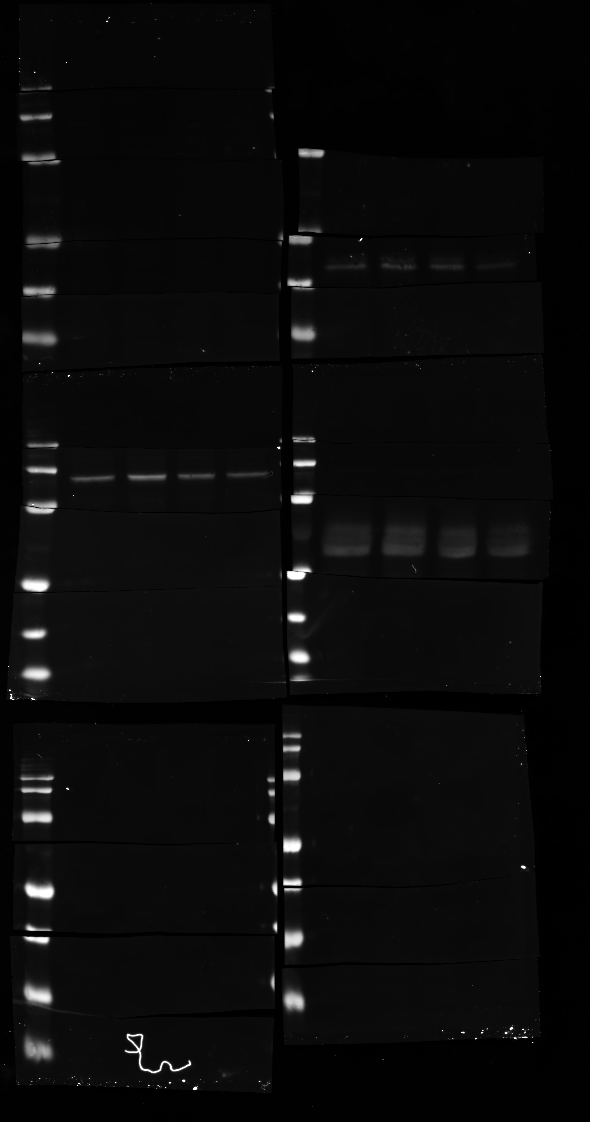

Supplement: Figure 3—source data 2. — Huh7 replicate 1 and SNU475 replicate 1–9. [file elife-102205-fig3-data2.zip › Figure 3-source data 2. Original files for western blot analysis displayed in Figure 3D and Figure 3-figure supplement 1C. Huh7 1st and SNU475/9th/230305-700.tif]

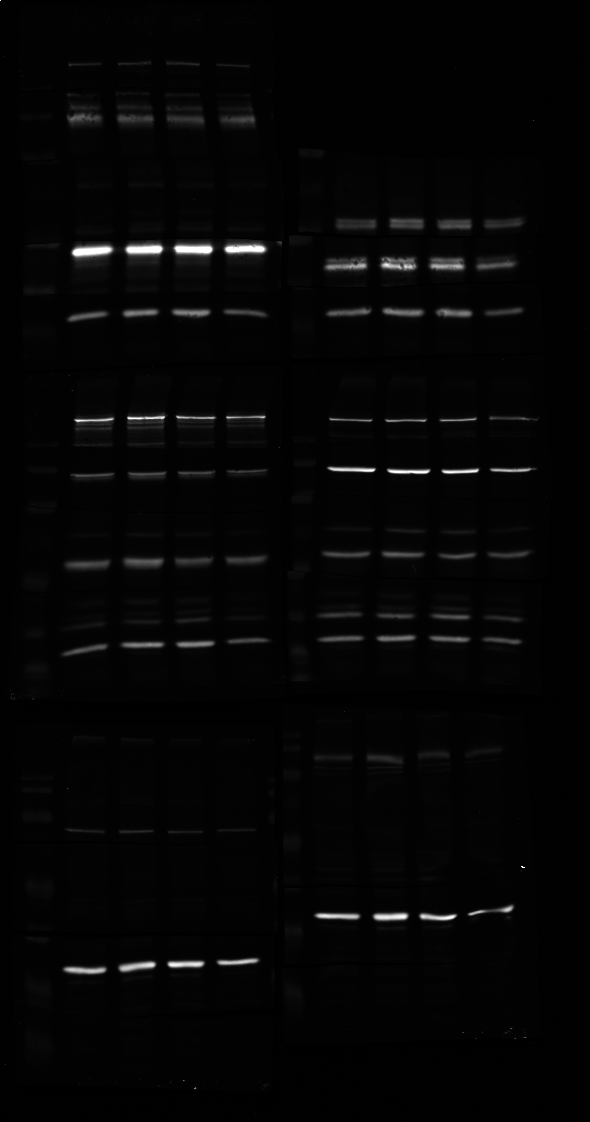

Supplement: Figure 3—source data 2. — Huh7 replicate 1 and SNU475 replicate 1–9. [file elife-102205-fig3-data2.zip › Figure 3-source data 2. Original files for western blot analysis displayed in Figure 3D and Figure 3-figure supplement 1C. Huh7 1st and SNU475/9th/230305-800.tif]

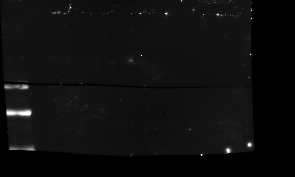

Supplement: Figure 3—source data 2. — Huh7 replicate 1 and SNU475 replicate 1–9. [file elife-102205-fig3-data2.zip › Figure 3-source data 2. Original files for western blot analysis displayed in Figure 3D and Figure 3-figure supplement 1C. Huh7 1st and SNU475/9th/230308-700.tif]

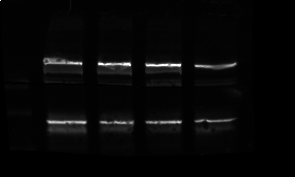

Supplement: Figure 3—source data 2. — Huh7 replicate 1 and SNU475 replicate 1–9. [file elife-102205-fig3-data2.zip › Figure 3-source data 2. Original files for western blot analysis displayed in Figure 3D and Figure 3-figure supplement 1C. Huh7 1st and SNU475/9th/230308-800.tif]

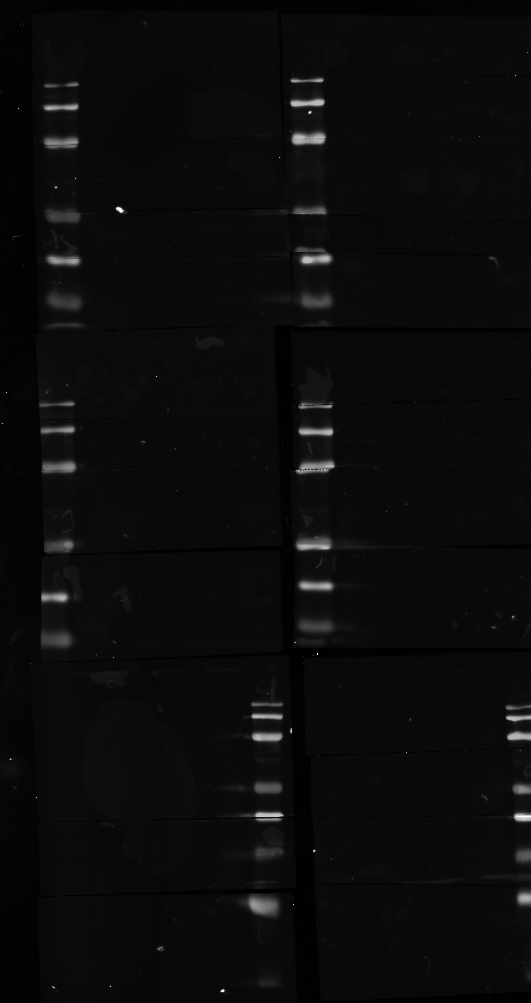

Supplement: Figure 3—source data 3. — Huh7 replicate 2–9. [file elife-102205-fig3-data3.zip › Figure 3-source data 3/230113_Huh7 2nd WT KO IFBD WT+PST/230113_Huh7 1st WT KO IFBD WT+PST 700.tif]

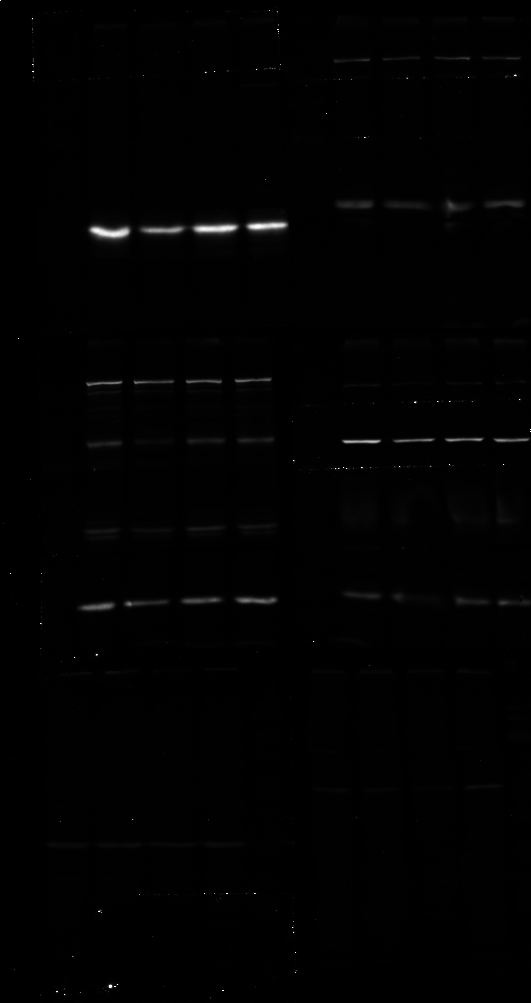

Supplement: Figure 3—source data 3. — Huh7 replicate 2–9. [file elife-102205-fig3-data3.zip › Figure 3-source data 3/230113_Huh7 2nd WT KO IFBD WT+PST/230113_Huh7 1st WT KO IFBD WT+PST 800.tif]

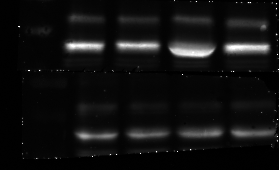

Supplement: Figure 3—source data 3. — Huh7 replicate 2–9. [file elife-102205-fig3-data3.zip › Figure 3-source data 3/230113_Huh7 2nd WT KO IFBD WT+PST/230113_Huh7 1st WT KO IFBD WT+PST GAPDH AB.tif]

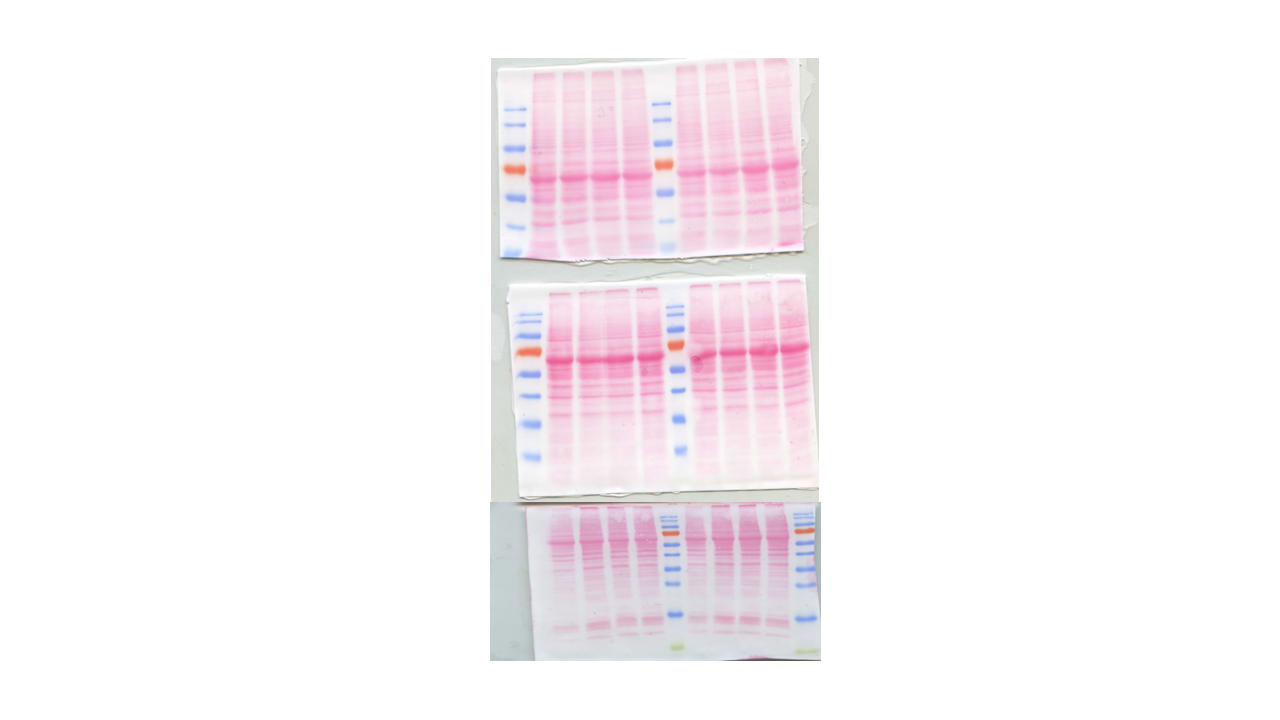

Supplement: Figure 3—source data 3. — Huh7 replicate 2–9. [file elife-102205-fig3-data3.zip › Figure 3-source data 3/230113_Huh7 2nd WT KO IFBD WT+PST/230113_Huh7 2nd WT KO IFBD WT+PST.tif]

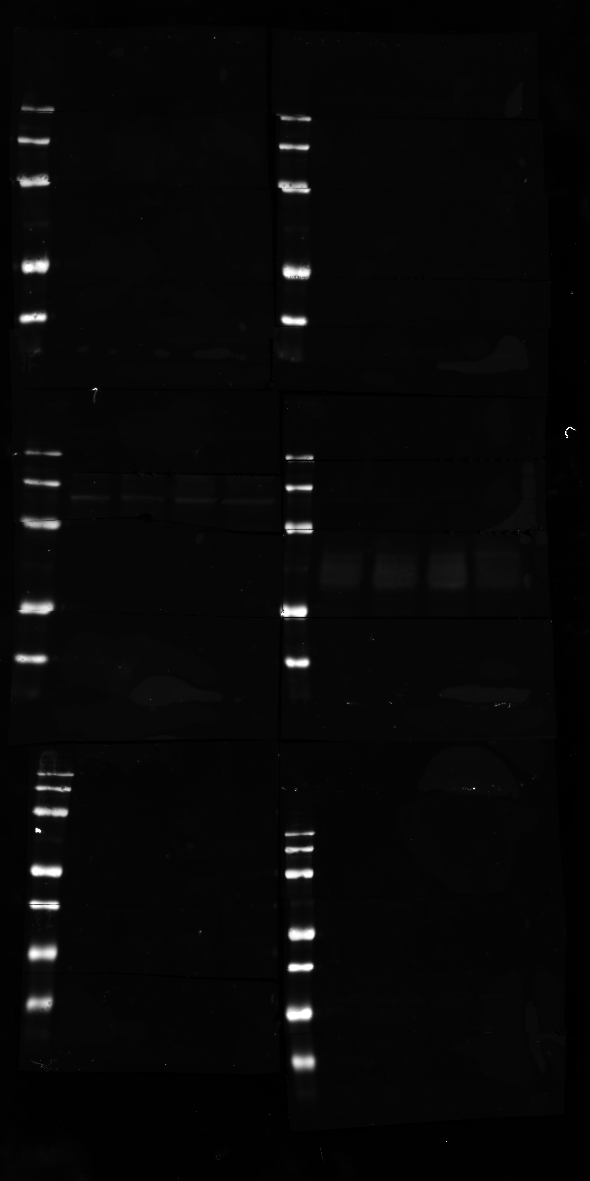

Supplement: Figure 3—source data 3. — Huh7 replicate 2–9. [file elife-102205-fig3-data3.zip › Figure 3-source data 3/230117_Huh7 3rd WT KO IFBD WT+PST/230117_Huh7 2nd WT KO IFBD WT+PST 700.tif]

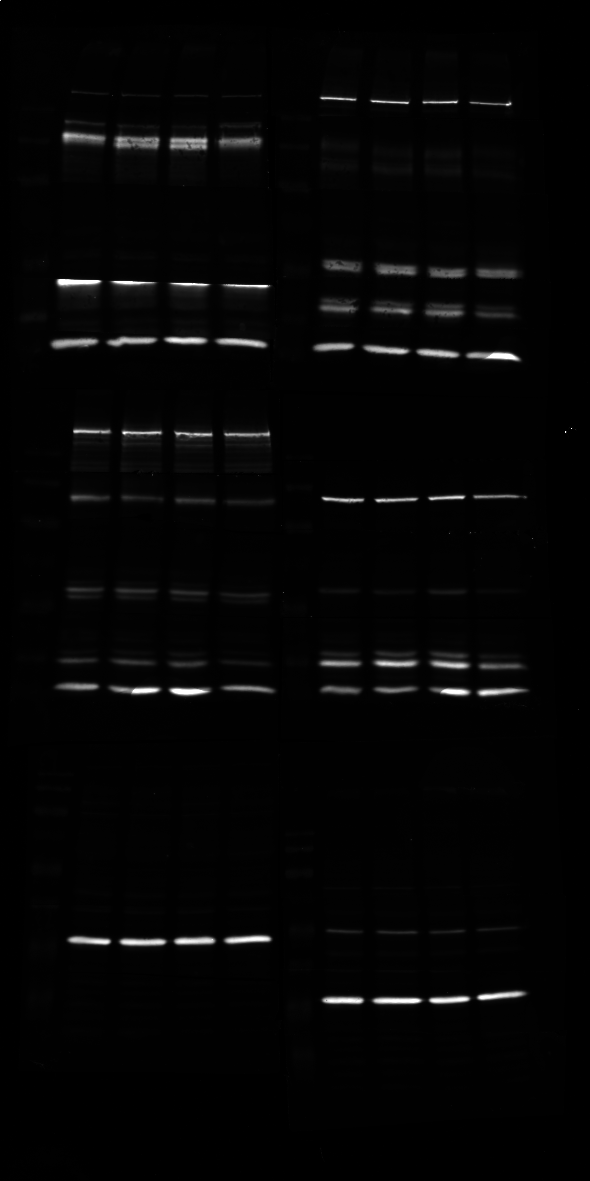

Supplement: Figure 3—source data 3. — Huh7 replicate 2–9. [file elife-102205-fig3-data3.zip › Figure 3-source data 3/230117_Huh7 3rd WT KO IFBD WT+PST/230117_Huh7 2nd WT KO IFBD WT+PST 800.tif]

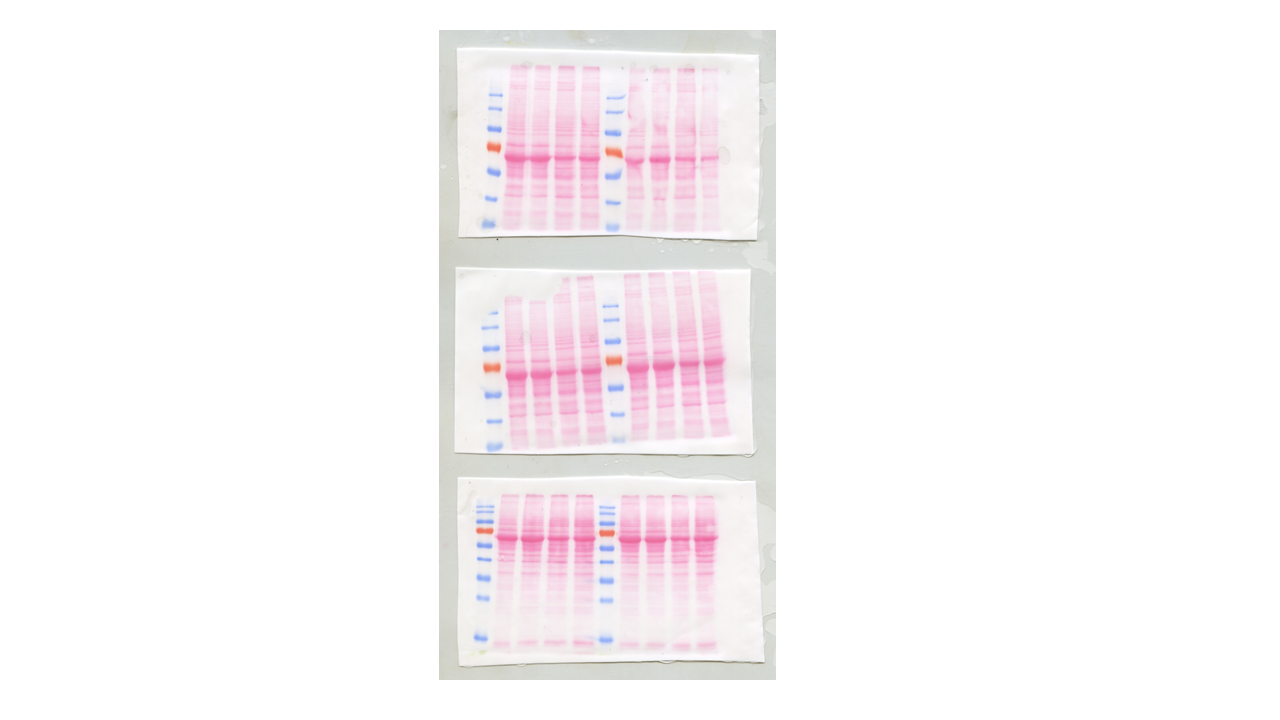

Supplement: Figure 3—source data 3. — Huh7 replicate 2–9. [file elife-102205-fig3-data3.zip › Figure 3-source data 3/230117_Huh7 3rd WT KO IFBD WT+PST/230117_Huh7 3rd WT KO IFBD WT+PST.tif]

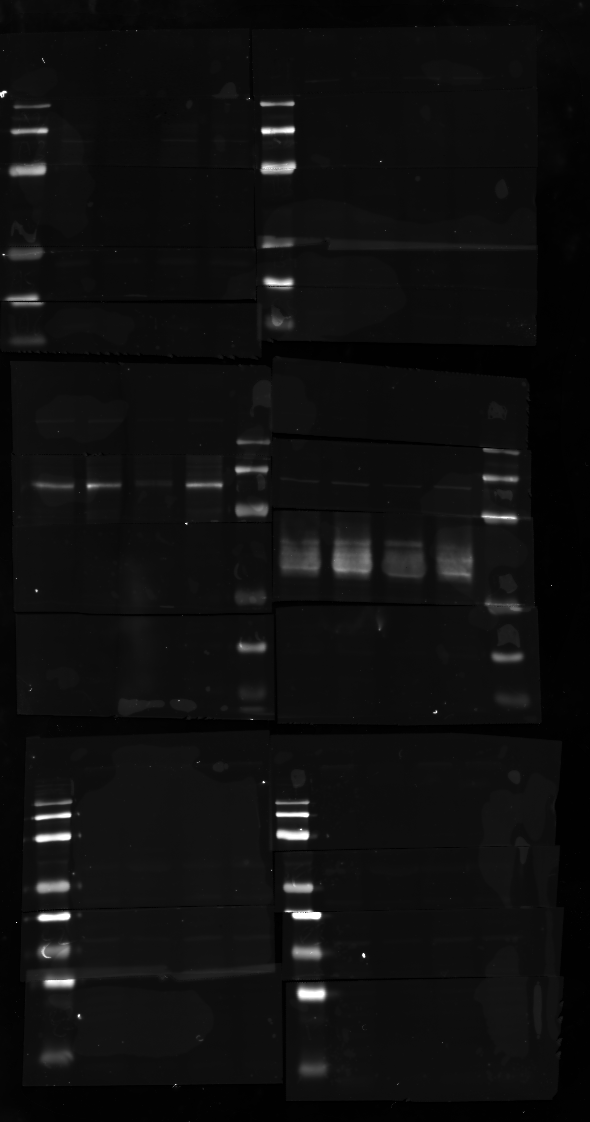

Supplement: Figure 3—source data 3. — Huh7 replicate 2–9. [file elife-102205-fig3-data3.zip › Figure 3-source data 3/230120_Huh7 4th WT KO IFBD WT+PST/230120_Huh7 3rd WT KO IFBD WT+PST 700.tif]

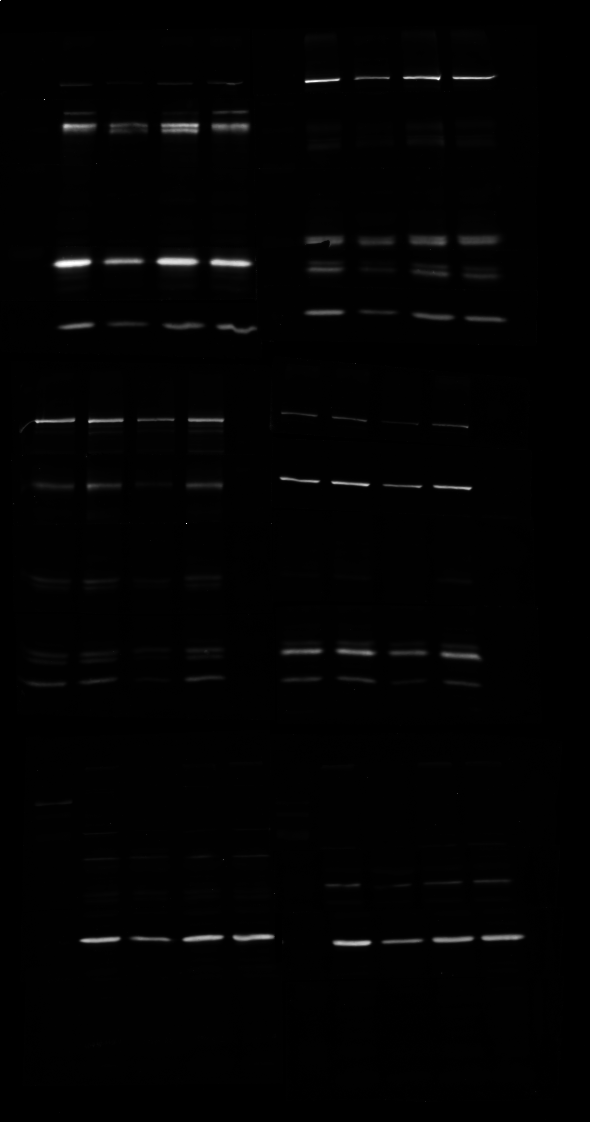

Supplement: Figure 3—source data 3. — Huh7 replicate 2–9. [file elife-102205-fig3-data3.zip › Figure 3-source data 3/230120_Huh7 4th WT KO IFBD WT+PST/230120_Huh7 3rd WT KO IFBD WT+PST 800.tif]

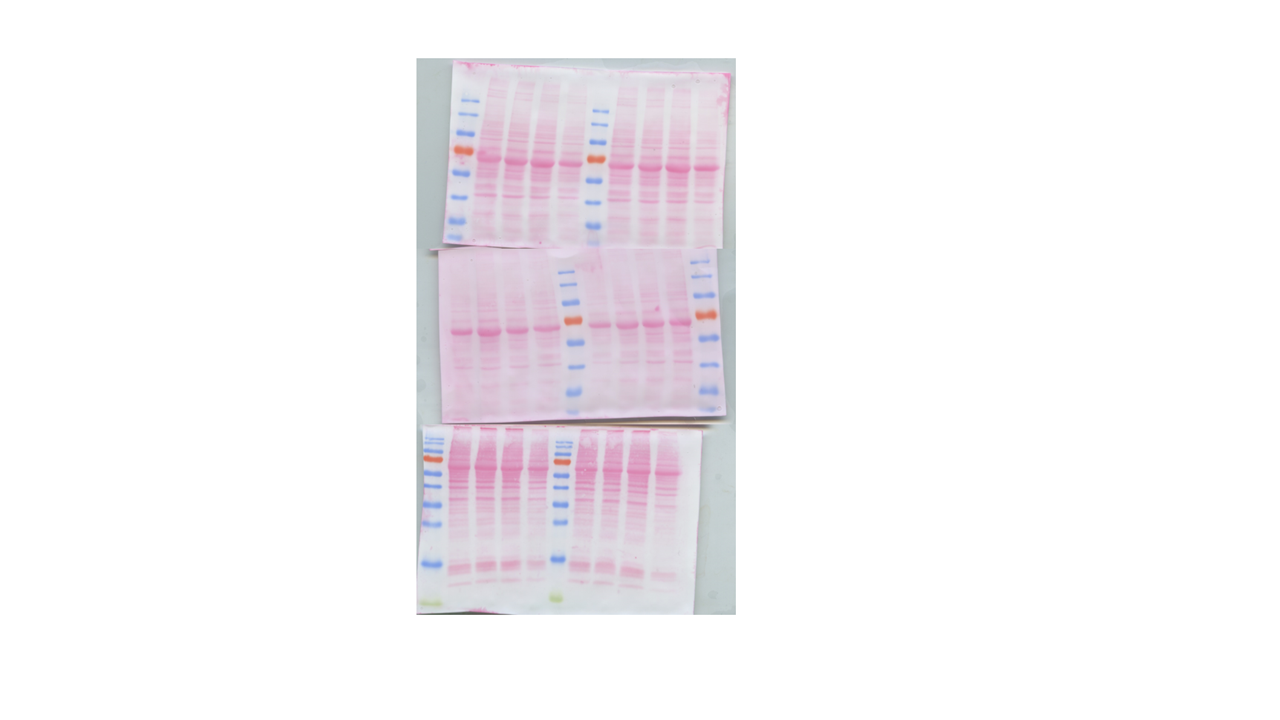

Supplement: Figure 3—source data 3. — Huh7 replicate 2–9. [file elife-102205-fig3-data3.zip › Figure 3-source data 3/230120_Huh7 4th WT KO IFBD WT+PST/230120_Huh7 4th WT KO IFBD WT+PST.tif]

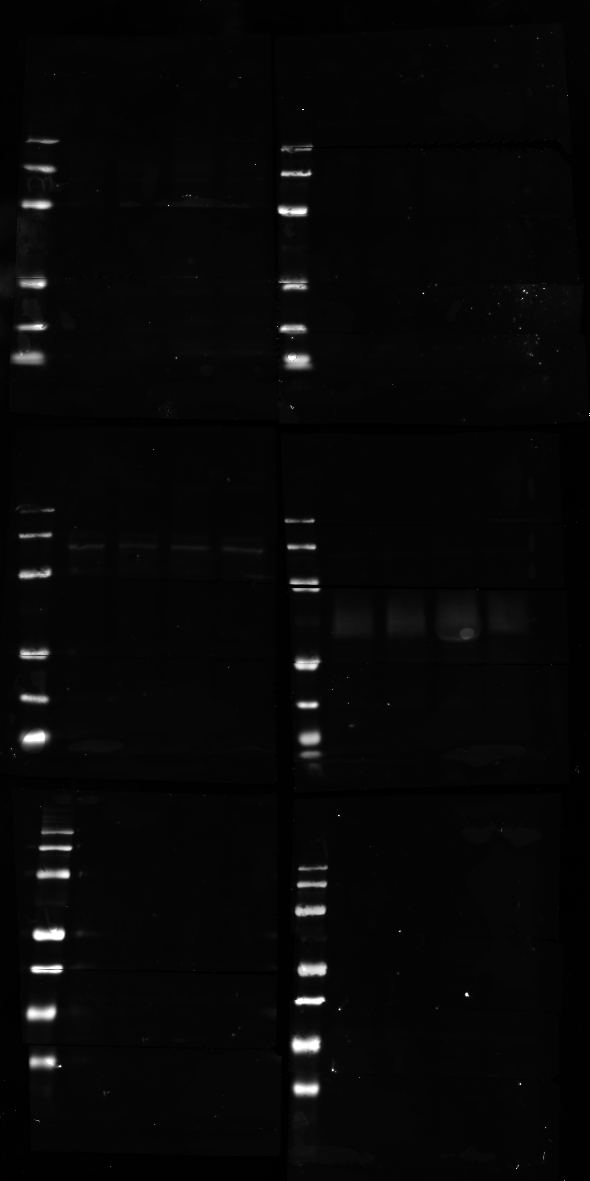

Supplement: Figure 3—source data 3. — Huh7 replicate 2–9. [file elife-102205-fig3-data3.zip › Figure 3-source data 3/230124_Huh7 5th WT KO IFBD WT+PST/230124_Huh7 4th WT KO IFBD WT+PST 700.tif]

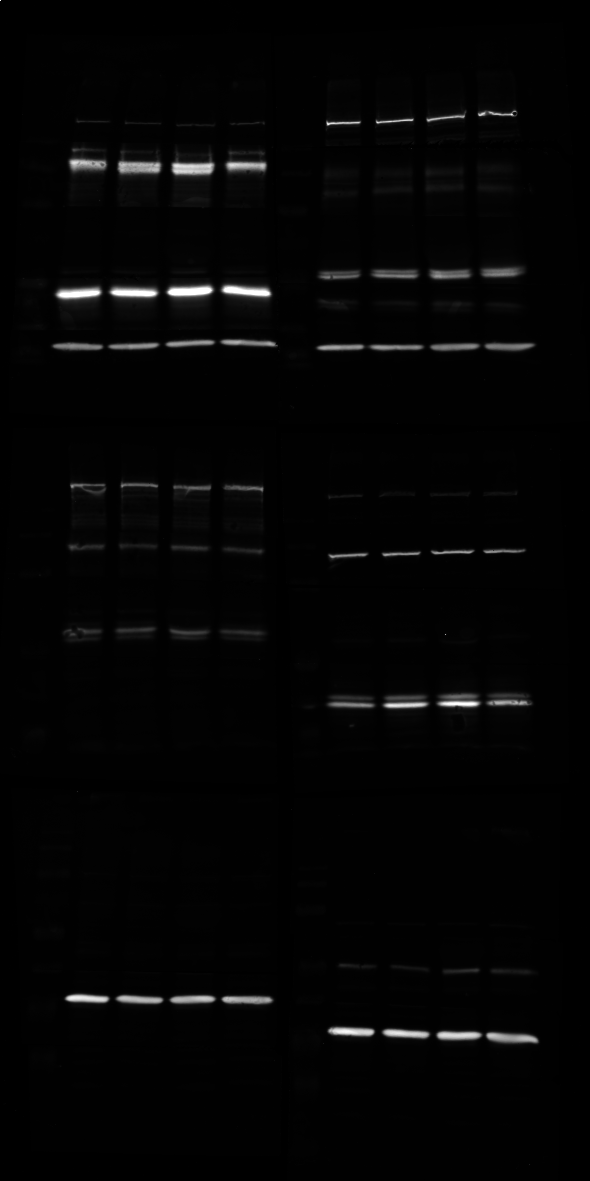

Supplement: Figure 3—source data 3. — Huh7 replicate 2–9. [file elife-102205-fig3-data3.zip › Figure 3-source data 3/230124_Huh7 5th WT KO IFBD WT+PST/230124_Huh7 4th WT KO IFBD WT+PST 800.tif]

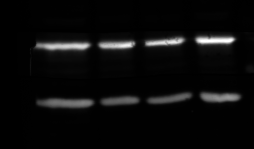

Supplement: Figure 3—source data 3. — Huh7 replicate 2–9. [file elife-102205-fig3-data3.zip › Figure 3-source data 3/230124_Huh7 5th WT KO IFBD WT+PST/230124_Huh7 4th WT KO IFBD WT+PST GAPDH CD.tif]

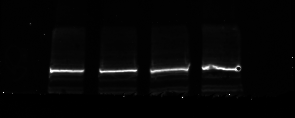

Supplement: Figure 3—source data 3. — Huh7 replicate 2–9. [file elife-102205-fig3-data3.zip › Figure 3-source data 3/230124_Huh7 5th WT KO IFBD WT+PST/230124_Huh7 4th WT KO IFBD WT+PST mTOR.tif]

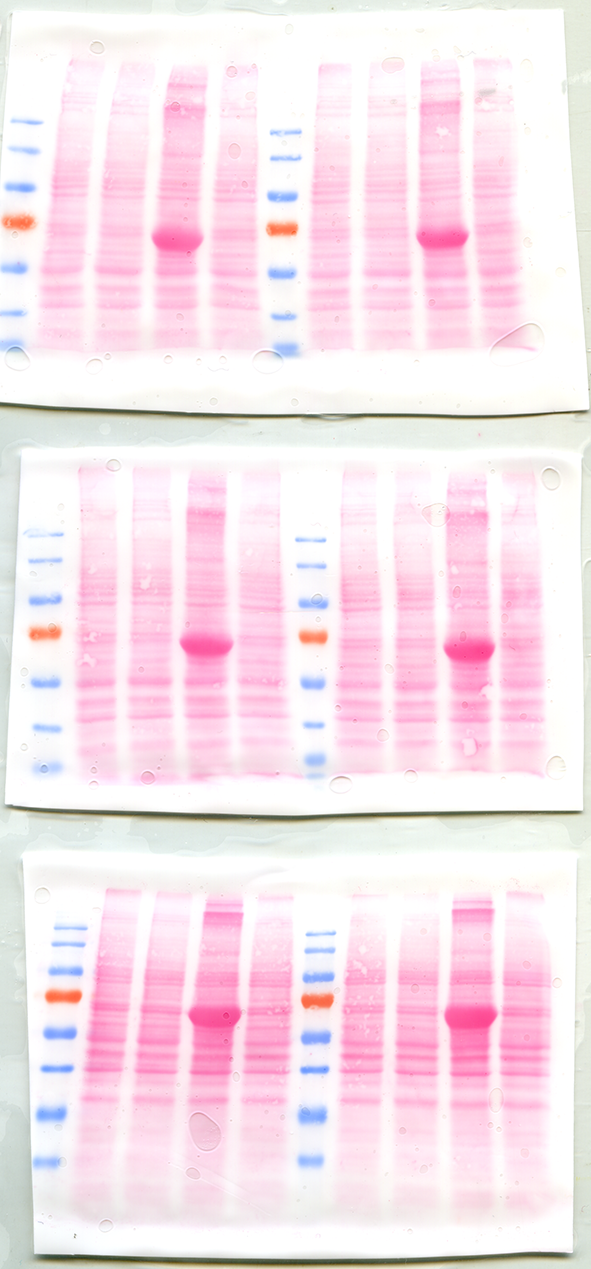

Supplement: Figure 3—source data 3. — Huh7 replicate 2–9. [file elife-102205-fig3-data3.zip › Figure 3-source data 3/230124_Huh7 5th WT KO IFBD WT+PST/230124_Huh7 5th WT KO IFBD WT+PST.tif]

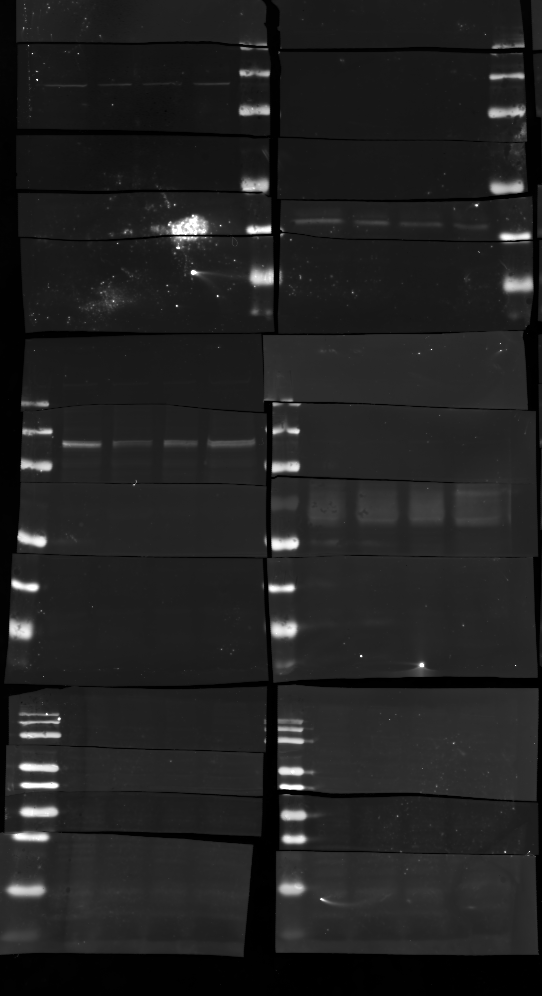

Supplement: Figure 3—source data 3. — Huh7 replicate 2–9. [file elife-102205-fig3-data3.zip › Figure 3-source data 3/230301_Huh7 6th WT KO IFBD WT+PST/230301_Huh7 5th WT KO IFBD WT+PST 700.tif]

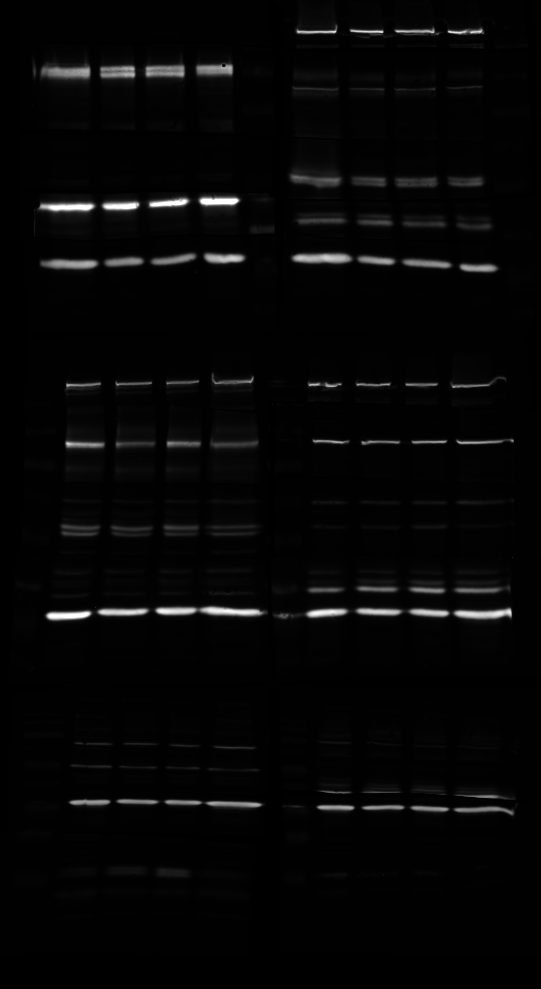

Supplement: Figure 3—source data 3. — Huh7 replicate 2–9. [file elife-102205-fig3-data3.zip › Figure 3-source data 3/230301_Huh7 6th WT KO IFBD WT+PST/230301_Huh7 5th WT KO IFBD WT+PST 800.tif]

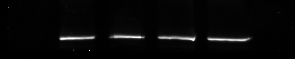

Supplement: Figure 3—source data 3. — Huh7 replicate 2–9. [file elife-102205-fig3-data3.zip › Figure 3-source data 3/230301_Huh7 6th WT KO IFBD WT+PST/230301_Huh7 5th WT KO IFBD WT+PST mTOR.tif]

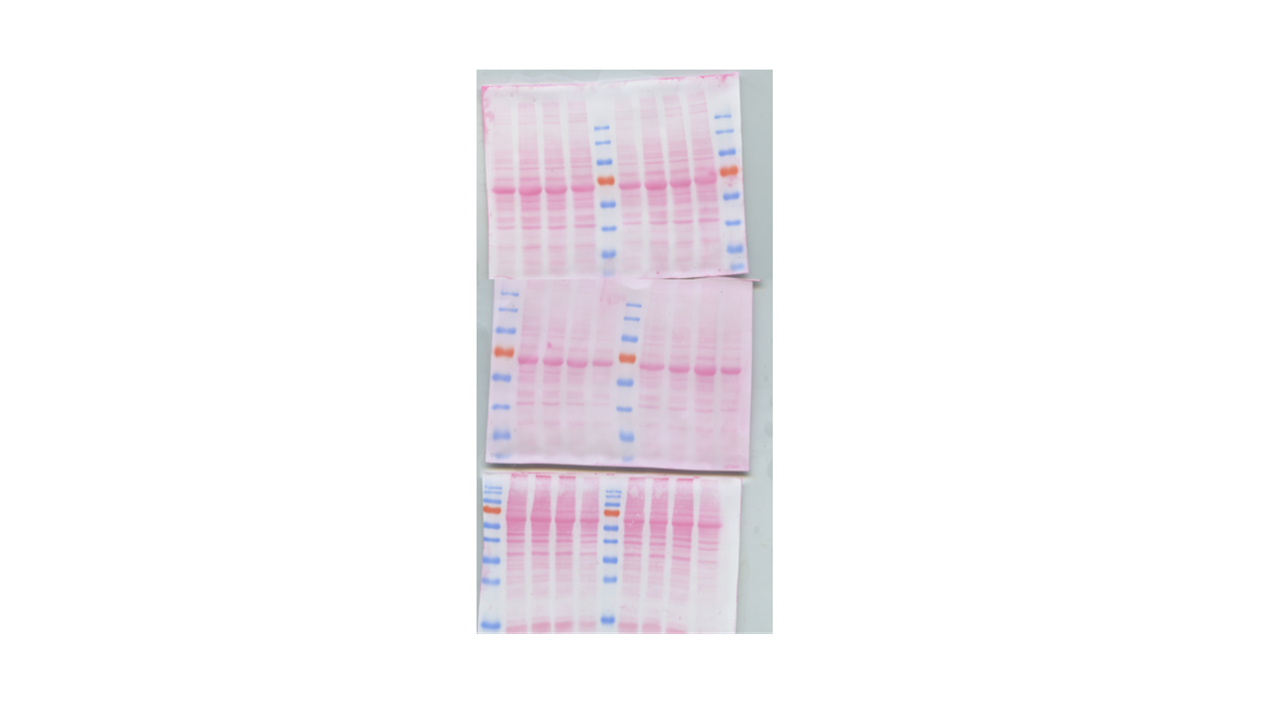

Supplement: Figure 3—source data 3. — Huh7 replicate 2–9. [file elife-102205-fig3-data3.zip › Figure 3-source data 3/230301_Huh7 6th WT KO IFBD WT+PST/230301_Huh7 6th WT KO IFBD WT+PST.tif]

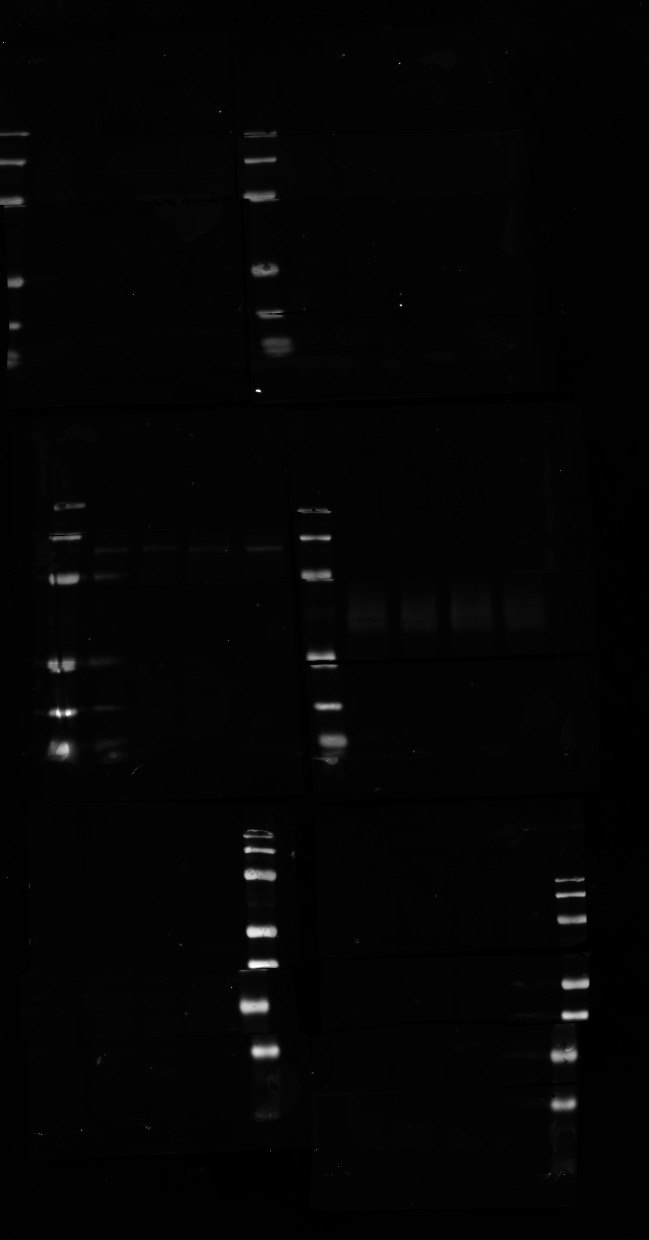

Supplement: Figure 3—source data 3. — Huh7 replicate 2–9. [file elife-102205-fig3-data3.zip › Figure 3-source data 3/230325_Huh7 7th WT KO IFBD WT+PST/230325_Huh7 6th WT KO IFBD WT+PST 700.tif]

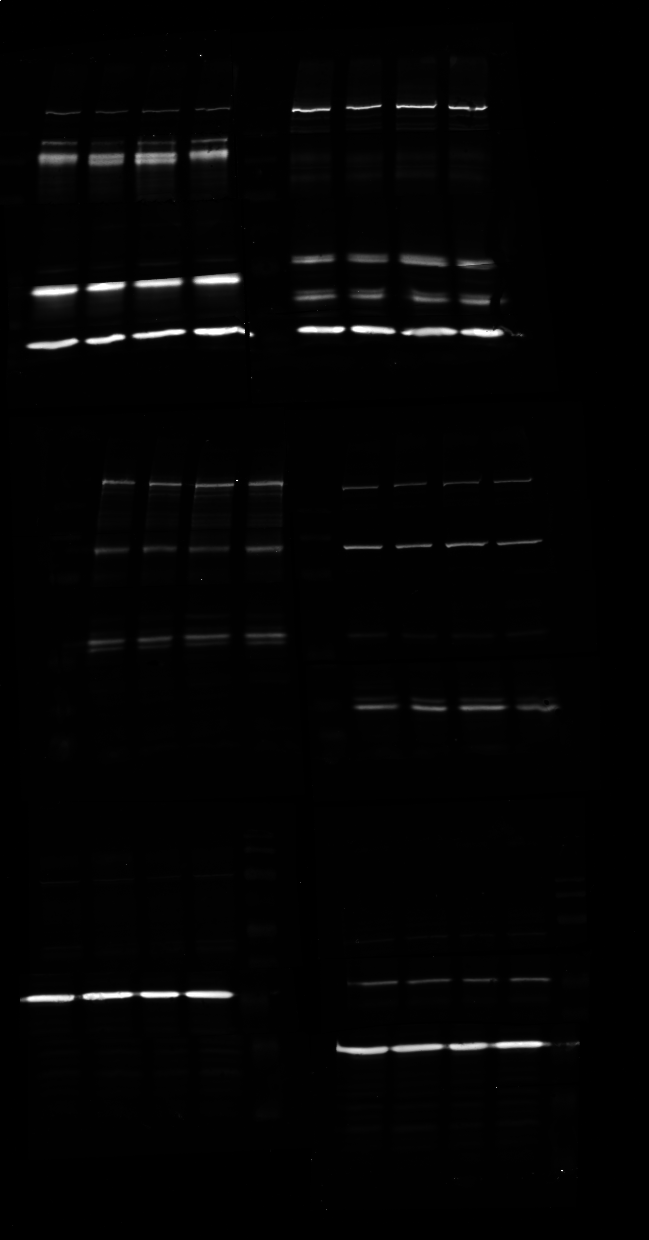

Supplement: Figure 3—source data 3. — Huh7 replicate 2–9. [file elife-102205-fig3-data3.zip › Figure 3-source data 3/230325_Huh7 7th WT KO IFBD WT+PST/230325_Huh7 6th WT KO IFBD WT+PST 800.tif]

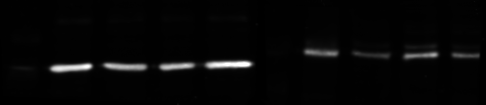

Supplement: Figure 3—source data 3. — Huh7 replicate 2–9. [file elife-102205-fig3-data3.zip › Figure 3-source data 3/230325_Huh7 7th WT KO IFBD WT+PST/230325_Huh7 6th WT KO IFBD WT+PST GAPDH.tif]

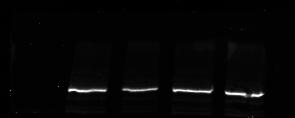

Supplement: Figure 3—source data 3. — Huh7 replicate 2–9. [file elife-102205-fig3-data3.zip › Figure 3-source data 3/230325_Huh7 7th WT KO IFBD WT+PST/230325_Huh7 6th WT KO IFBD WT+PST mTOR.tif]

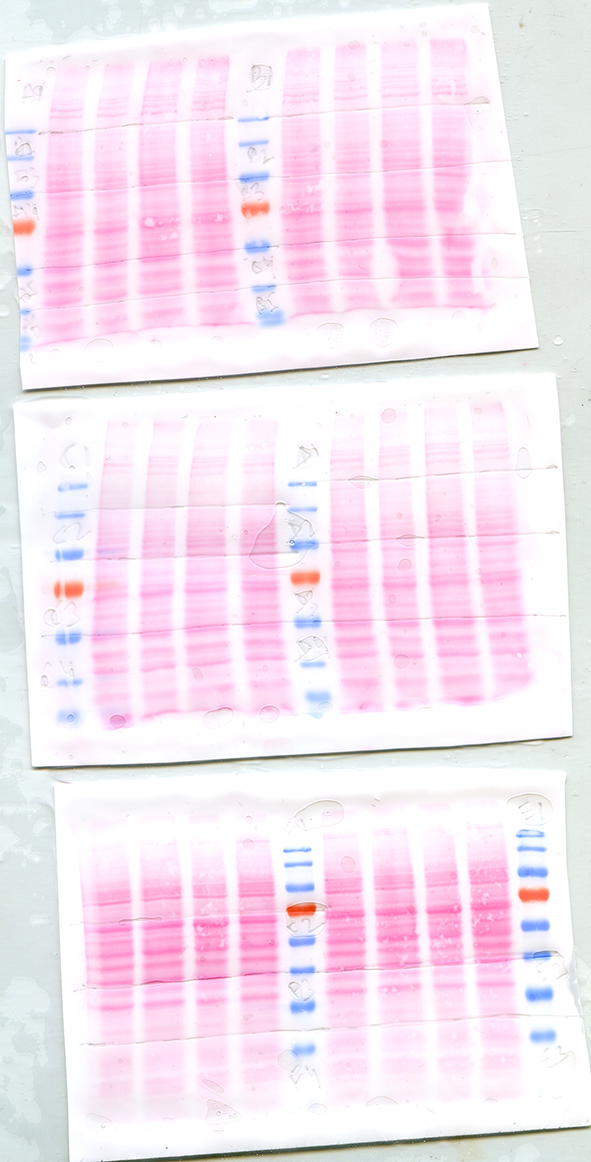

Supplement: Figure 3—source data 3. — Huh7 replicate 2–9. [file elife-102205-fig3-data3.zip › Figure 3-source data 3/230325_Huh7 7th WT KO IFBD WT+PST/230325_Huh7 7th WT KO IFBD WT+PST marked.tif]

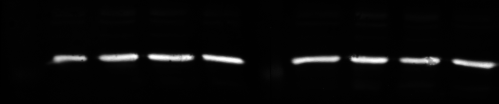

Supplement: Figure 3—source data 3. — Huh7 replicate 2–9. [file elife-102205-fig3-data3.zip › Figure 3-source data 3/230328_Huh7 8th WT KO IFBD WT+PST/230325_Huh7 7th WT KO IFBD WT+PST GAPDH.tif]

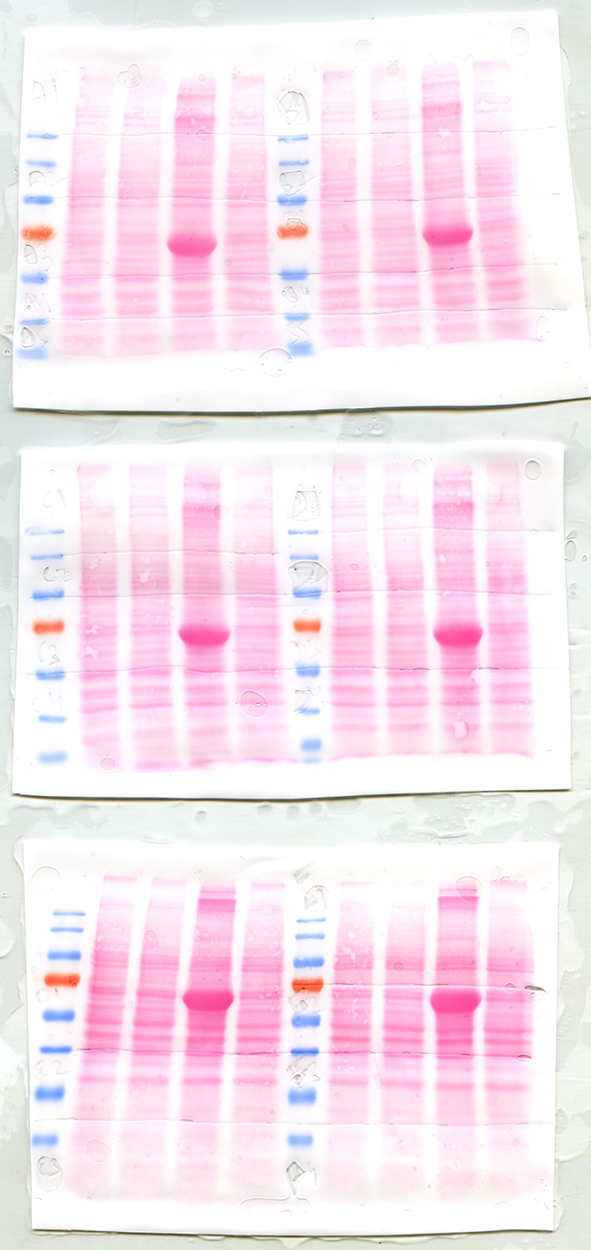

Supplement: Figure 3—source data 3. — Huh7 replicate 2–9. [file elife-102205-fig3-data3.zip › Figure 3-source data 3/230328_Huh7 8th WT KO IFBD WT+PST/230328_Huh7 8th WT KO IFBD WT+PST marked.tif]

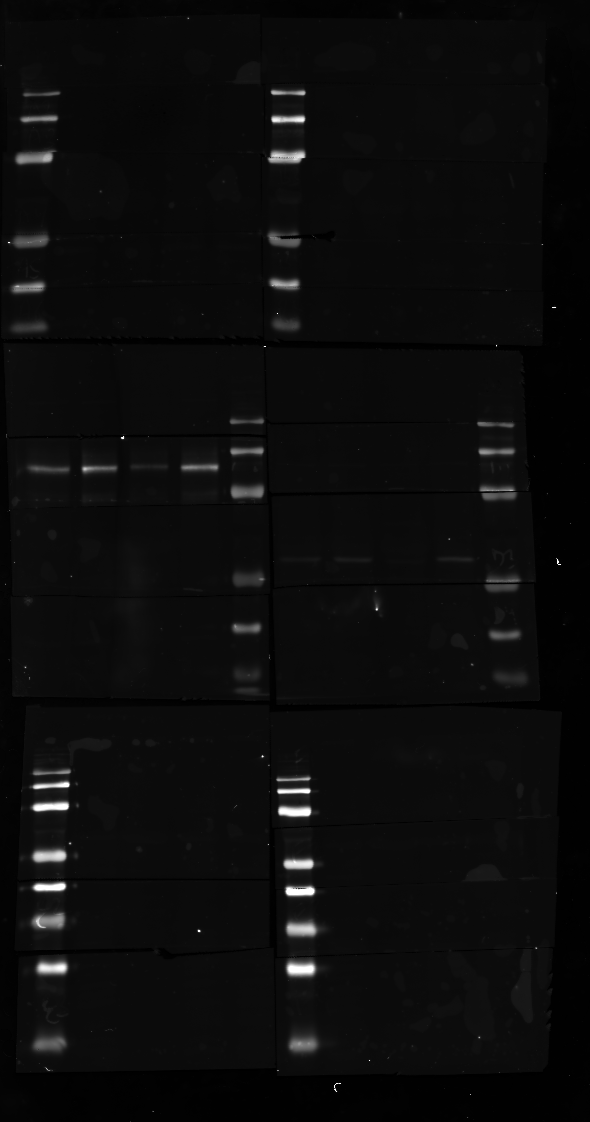

Supplement: Figure 3—source data 3. — Huh7 replicate 2–9. [file elife-102205-fig3-data3.zip › Figure 3-source data 3/230331_Huh7 9th WT KO IFBD WT+PST/230331_Huh7 8th WT KO IFBD WT+PST 700.tif]
