# Supplementary material for: Plectin-mediated cytoskeletal crosstalk as a target for inhibition of hepatocellular carcinoma growth and metastasis
Source: eLife. 2025 Mar 7;13:RP102205. doi: 10.7554/eLife.102205 (PMC11893104; doi:10.7554/eLife.102205)

SNU-475 replicate 1 (221209)

Figure 3D

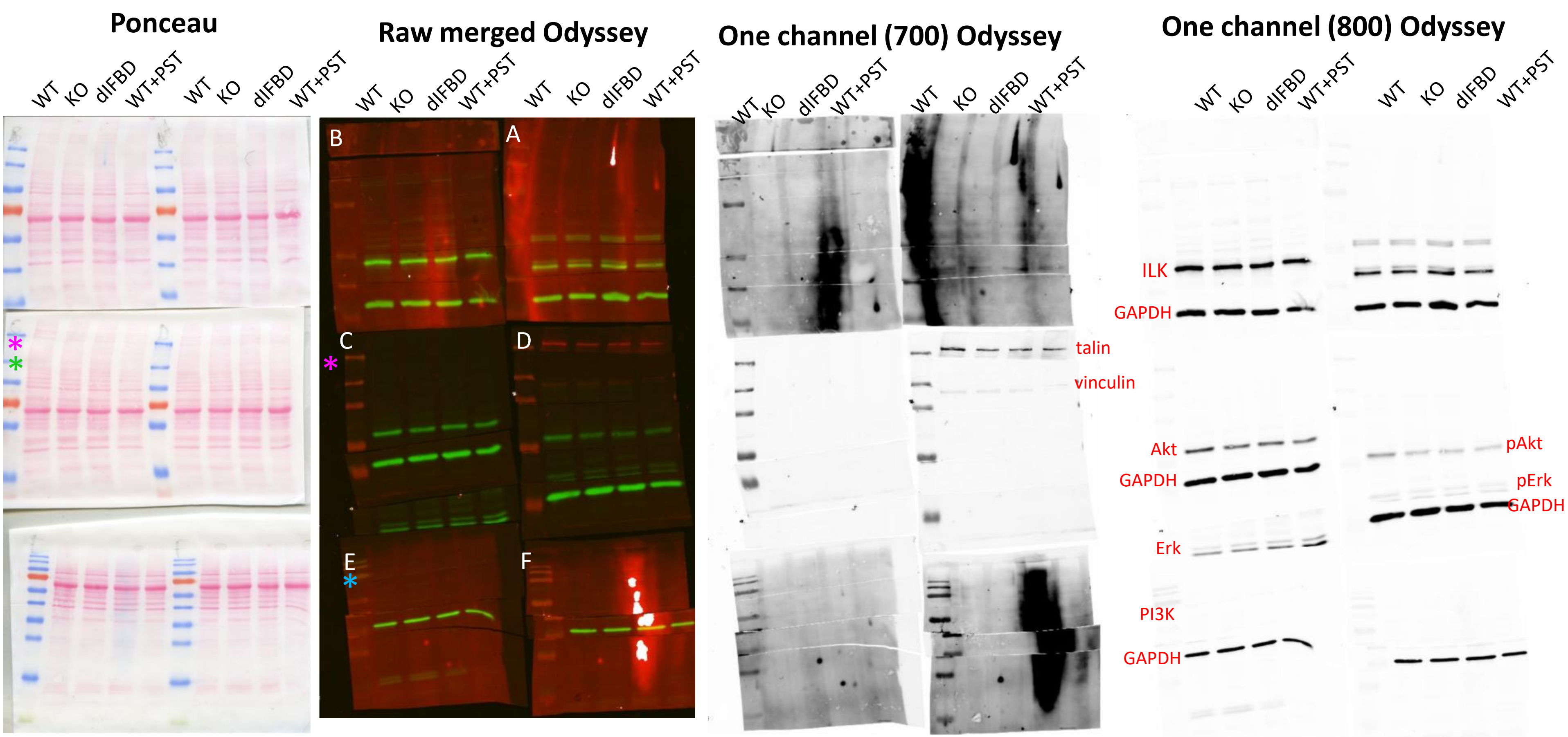

* pFAK (rabbit 800) and FAK (mouse 700) restained on membrane C (file **230105**)

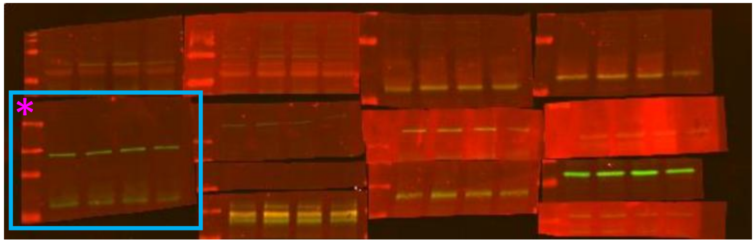

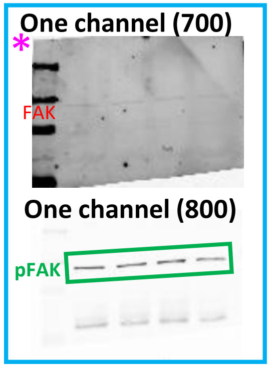

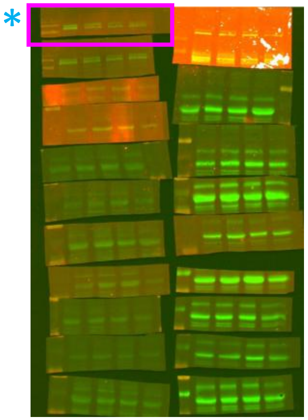

* Integrin b1 stained on membrane E (file **230310**)

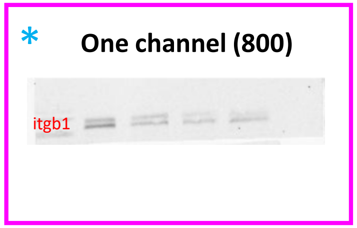

SNU-475 replicate 2 and 3 (221216)

Figure 3D

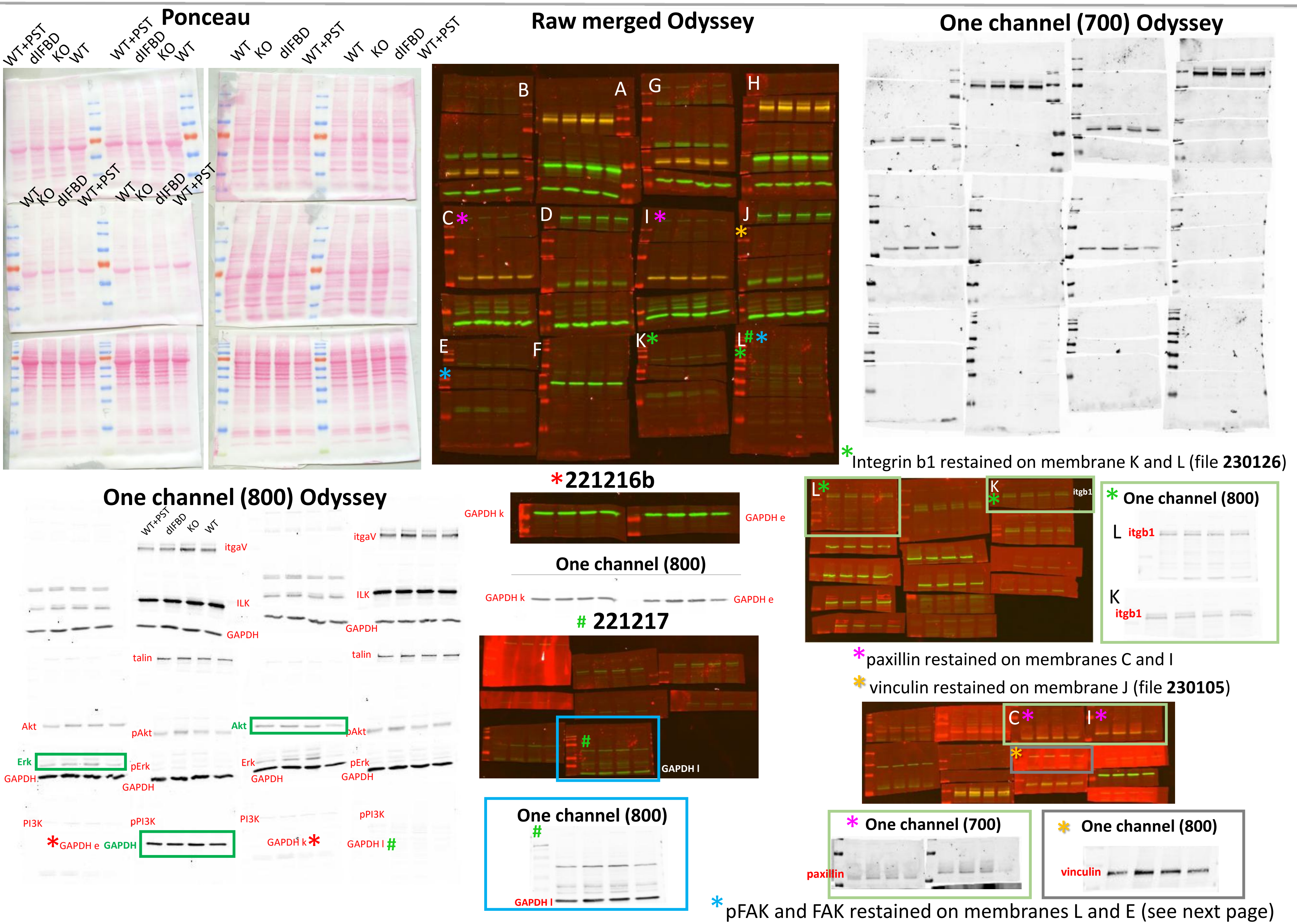

SNU-475 replicate 4 and 5 (221221)

Ponceau

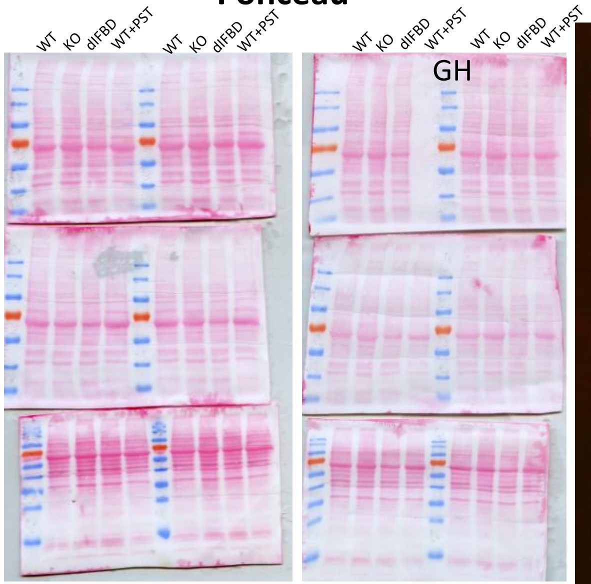

Raw merged Odyssey

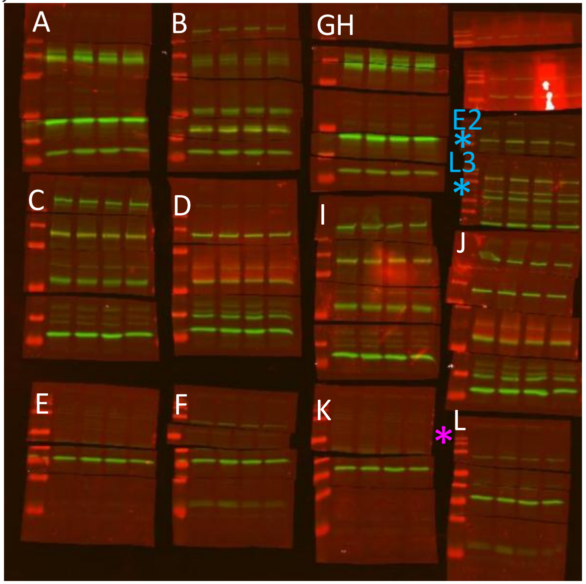

One channel (700) Odyssey

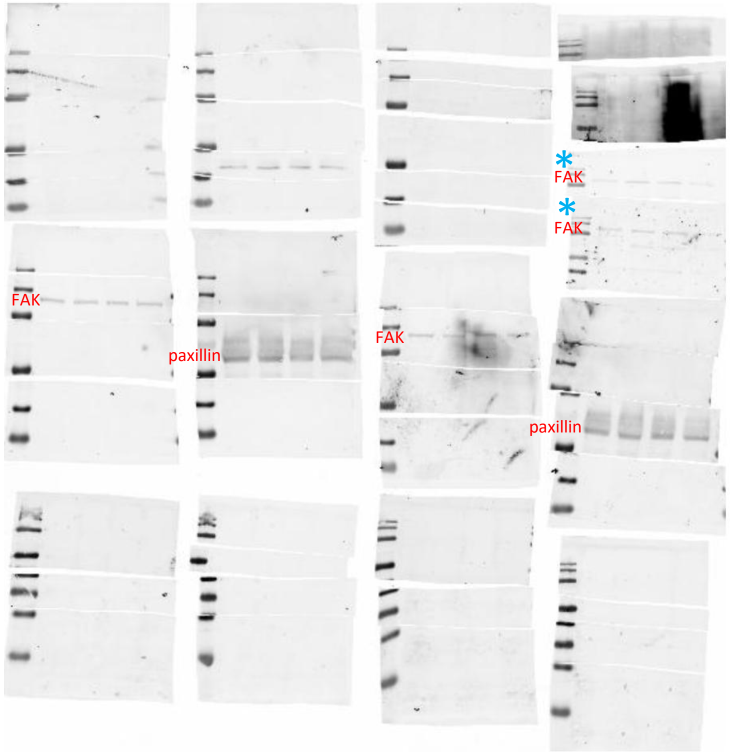

One channel (800) Odyssey

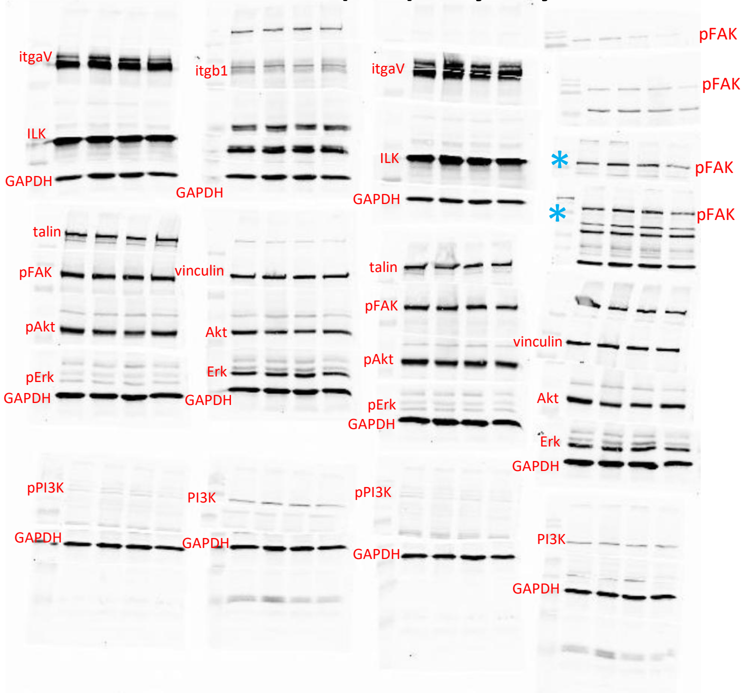

* Integrin b1 of 5th replicate stained on membrane L (file **230126**)

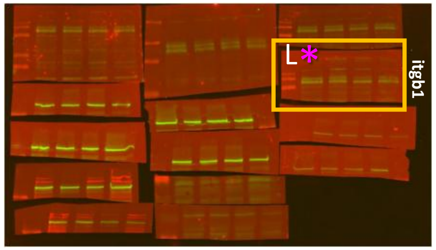

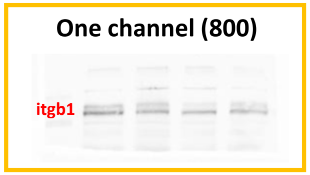

* pFAK and FAK from **221216** restained on membrane E and L (see * on previous page).

Huh7 replicate 1 and SNU-475 replicate 6 (230125)

Figure 3D

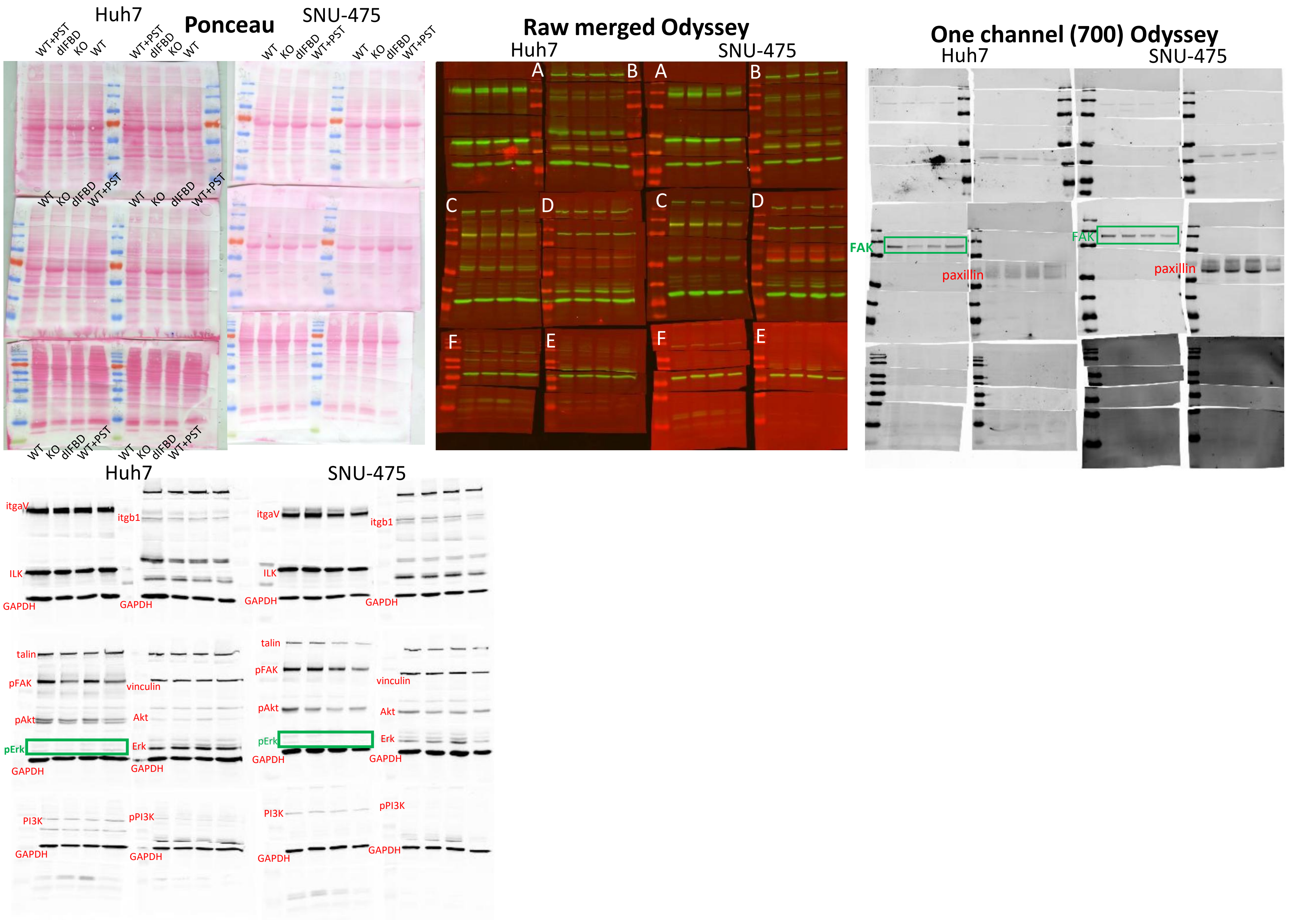

SNU-475 replicate 7 and 8 (230224)

Figure 3D

Ponceau

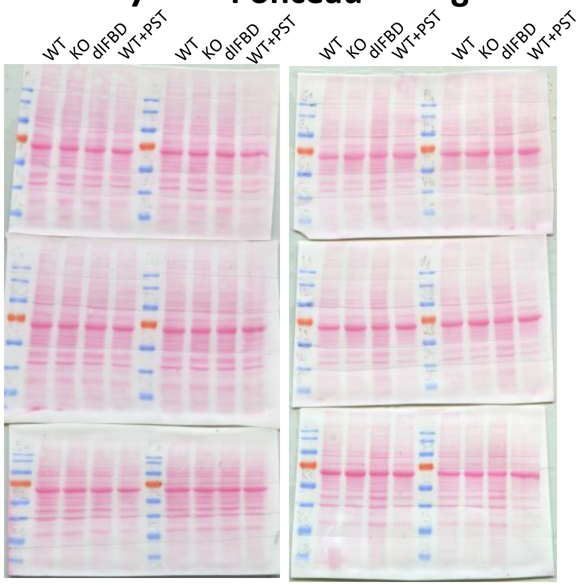

Raw merged Odyssey

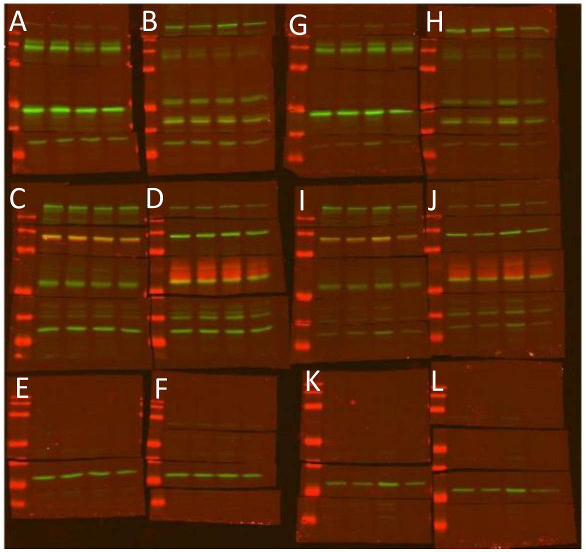

One channel (700) Odyssey

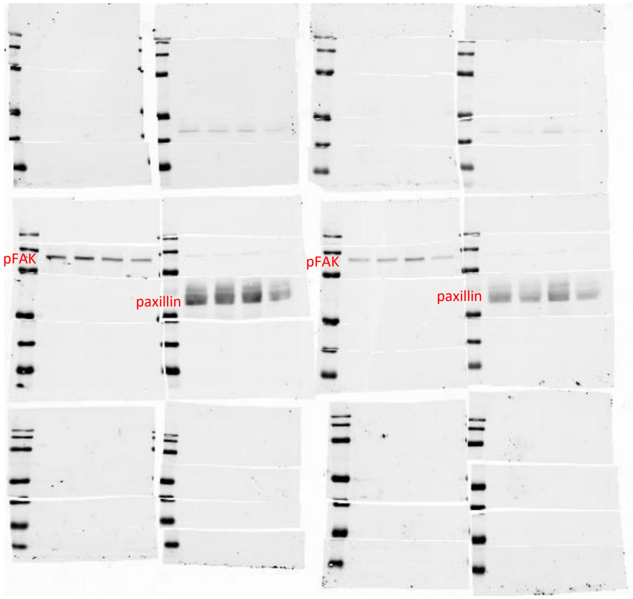

One channel (800) Odyssey

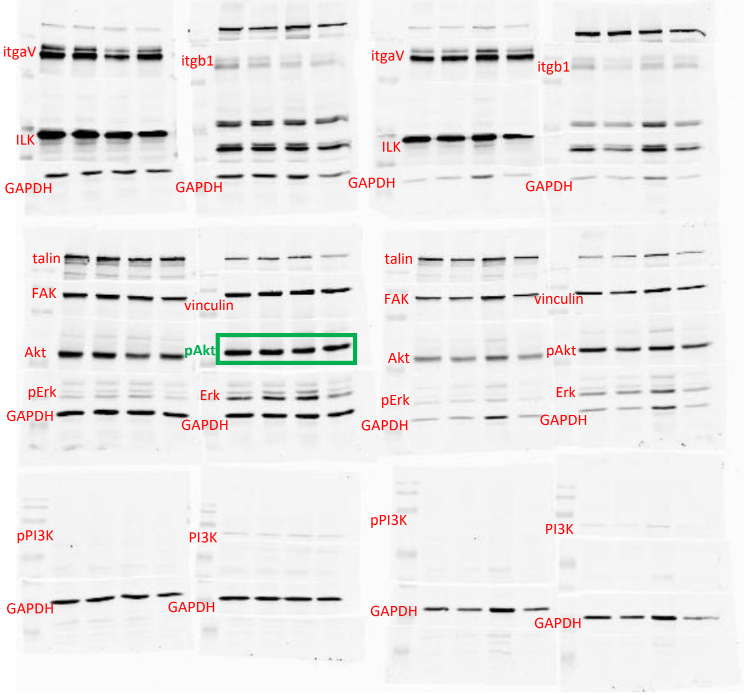

SNU-475 replicate 9 (230305)

Ponceau

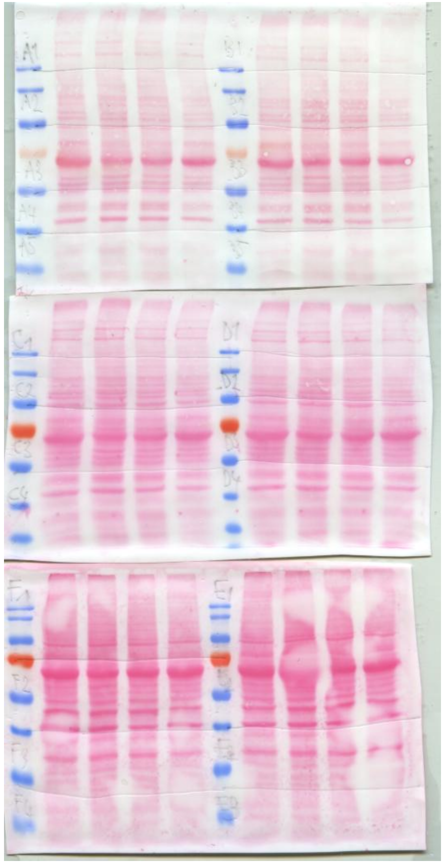

Raw merged Odyssey

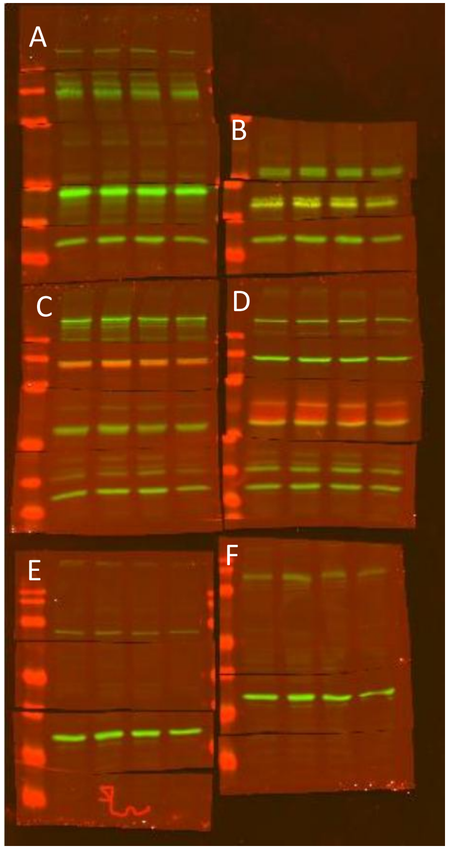

One channel (700) Odyssey

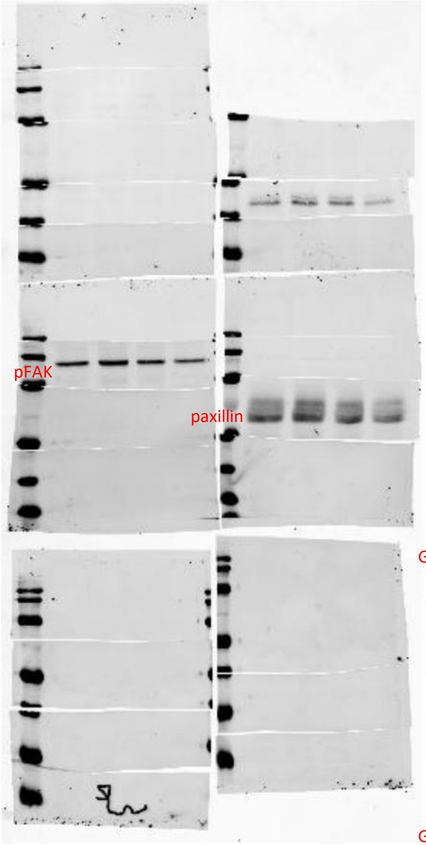

One channel (800) Odyssey

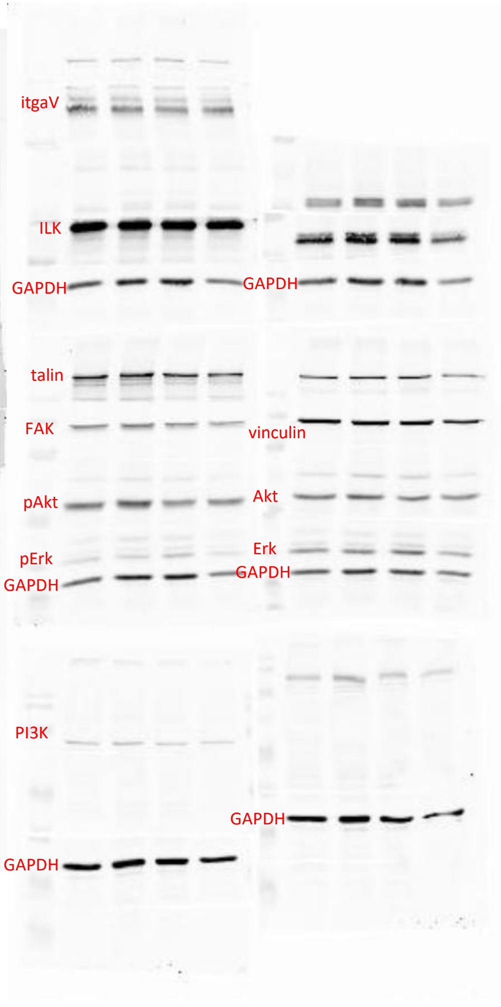

230308

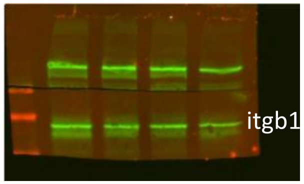

One channel (800) Odyssey

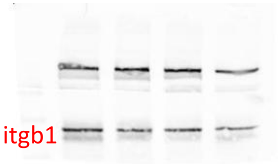

Huh7 replicate 2 (230113)

Ponceau

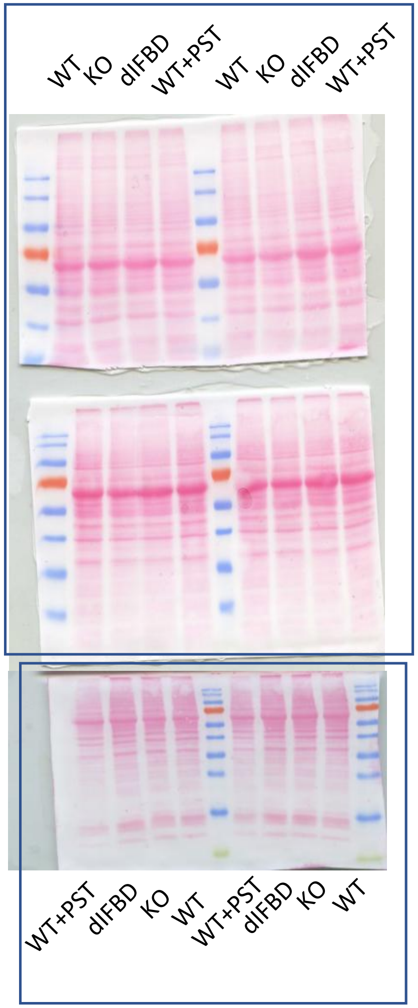

Raw merged Odyssey

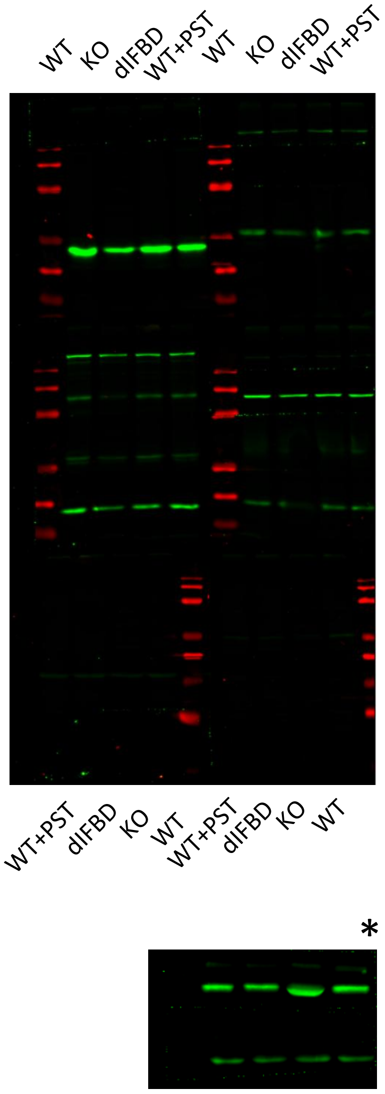

One channel (700) Odyssey

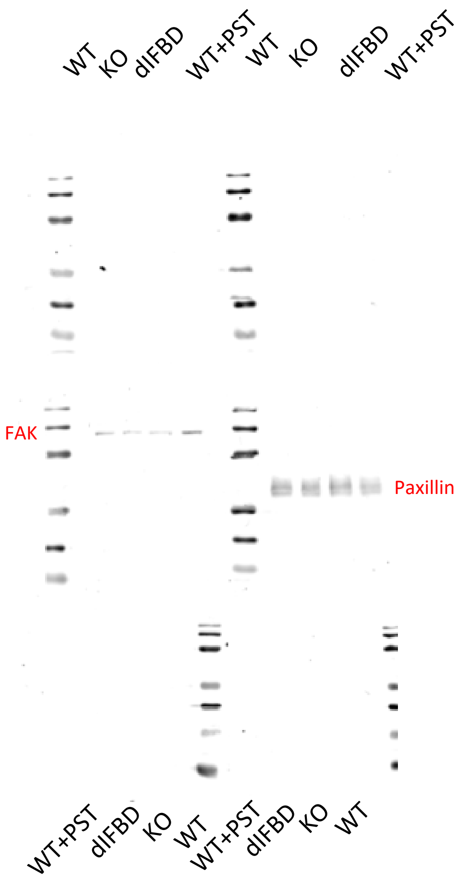

One channel (800) Odyssey

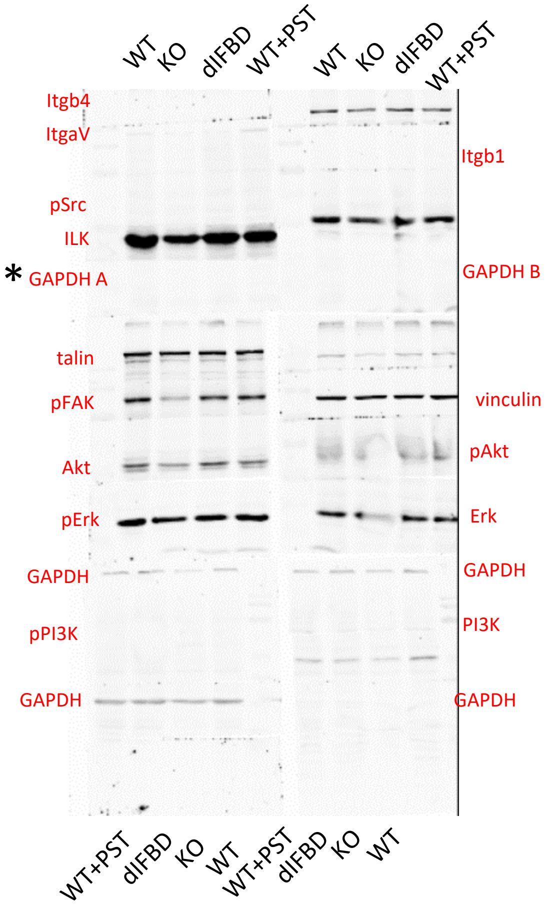

REIMAGED 800 CHANNEL

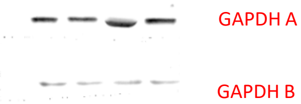

Huh7 replicate 3 (230117)

Figure 3D

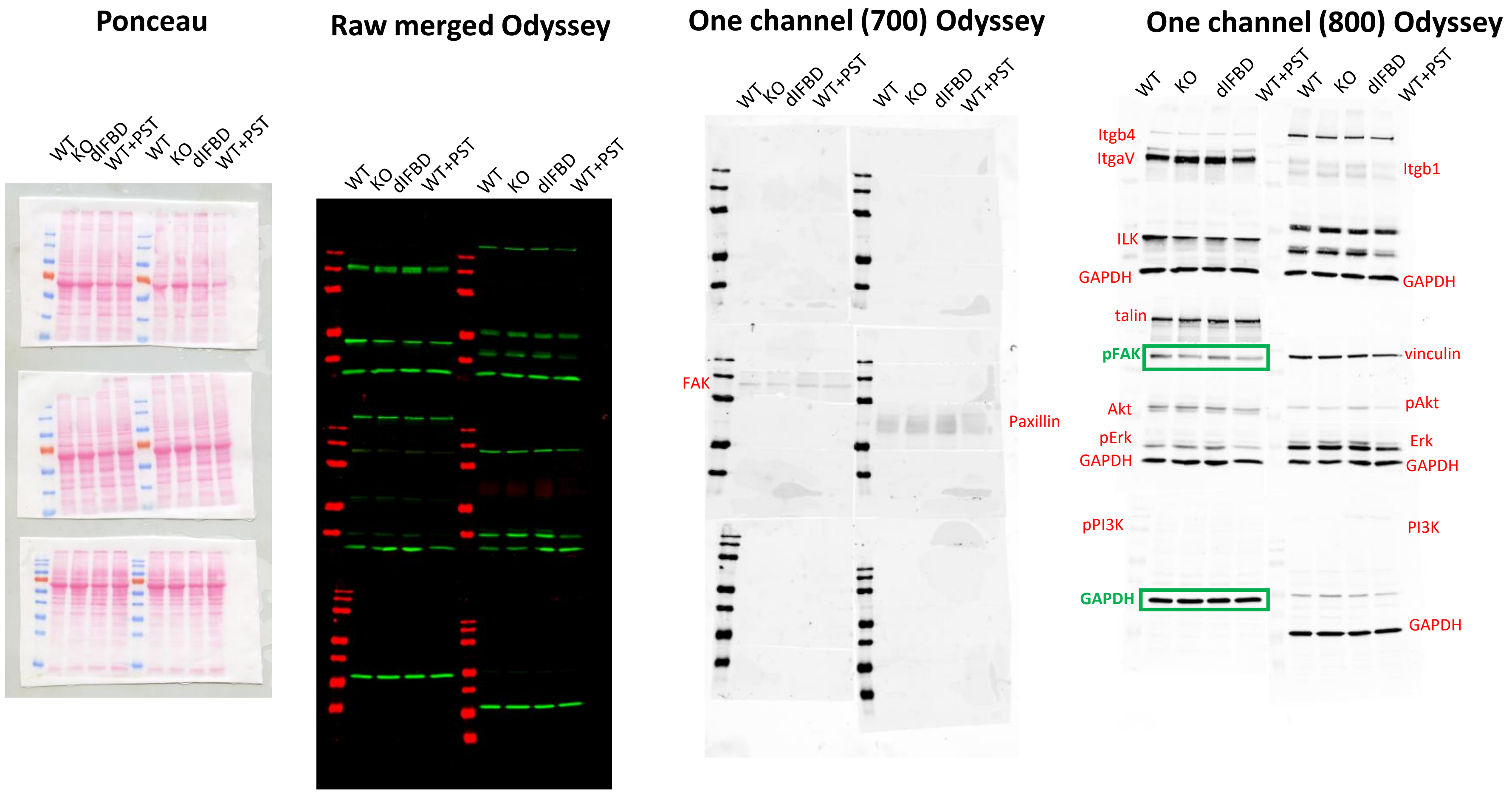

Huh7 replicate 4 (230120)

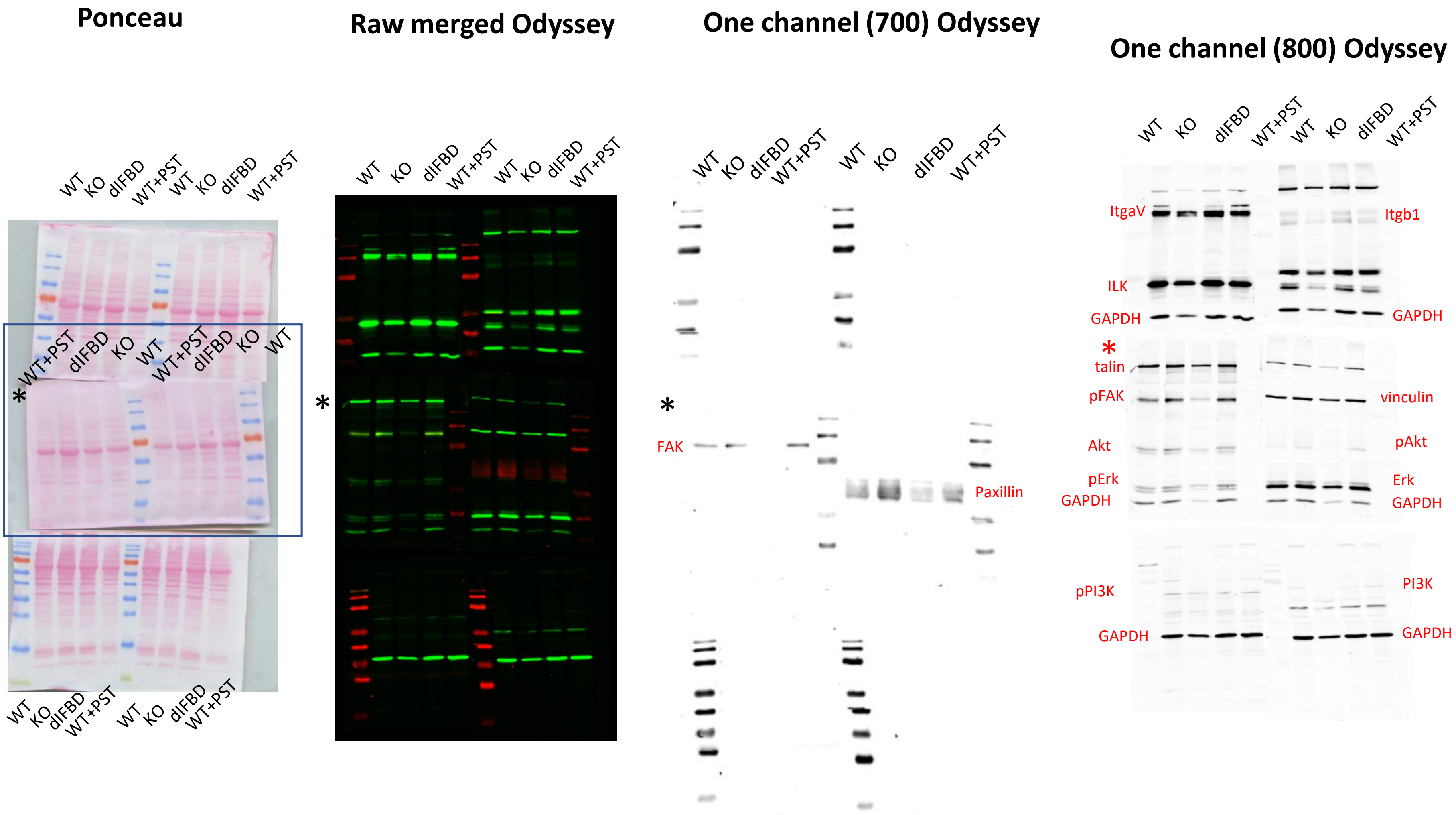

Huh7 replicate 5 (230124)

Figure 3D

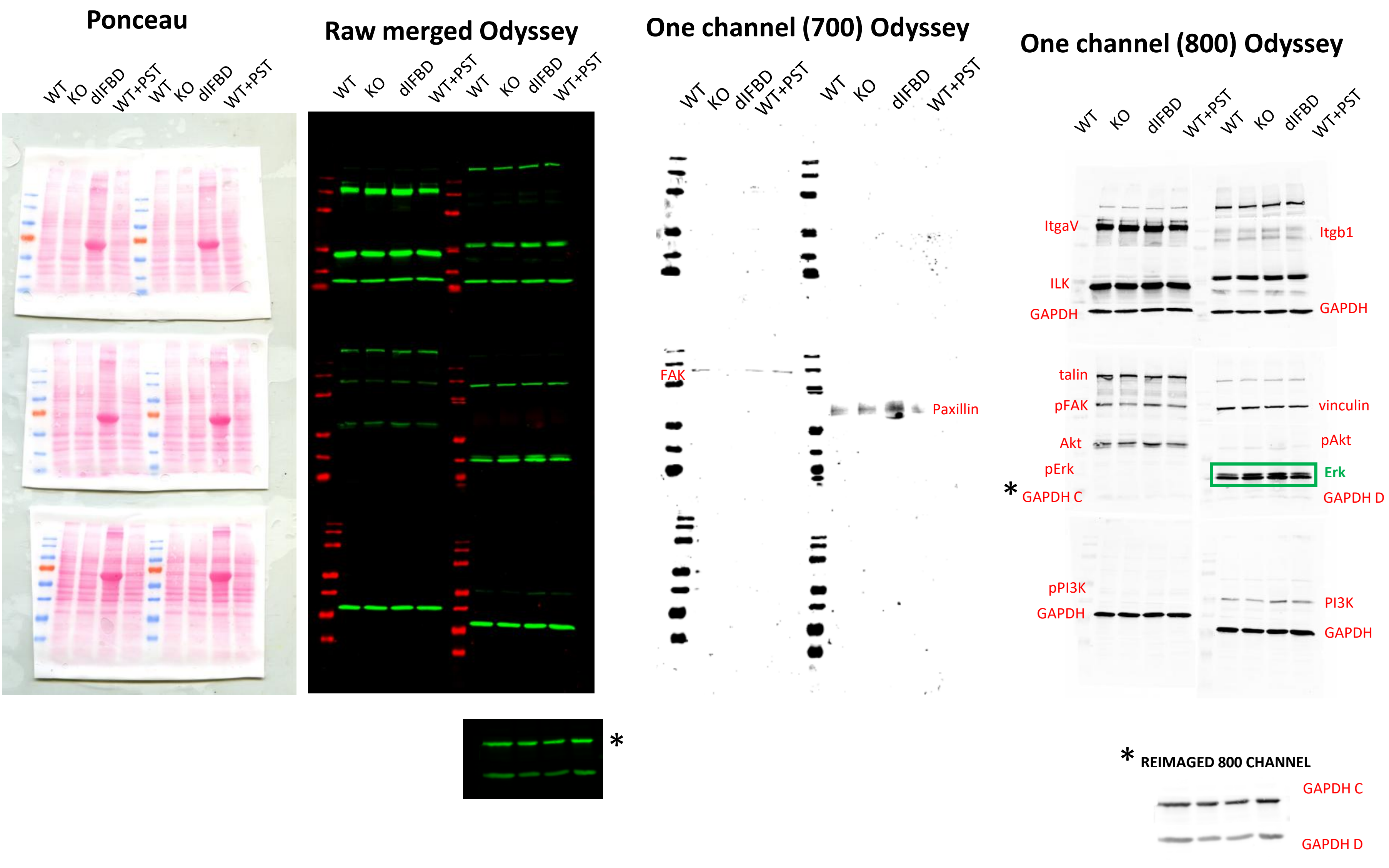

Huh7 replicate 6 (230301)

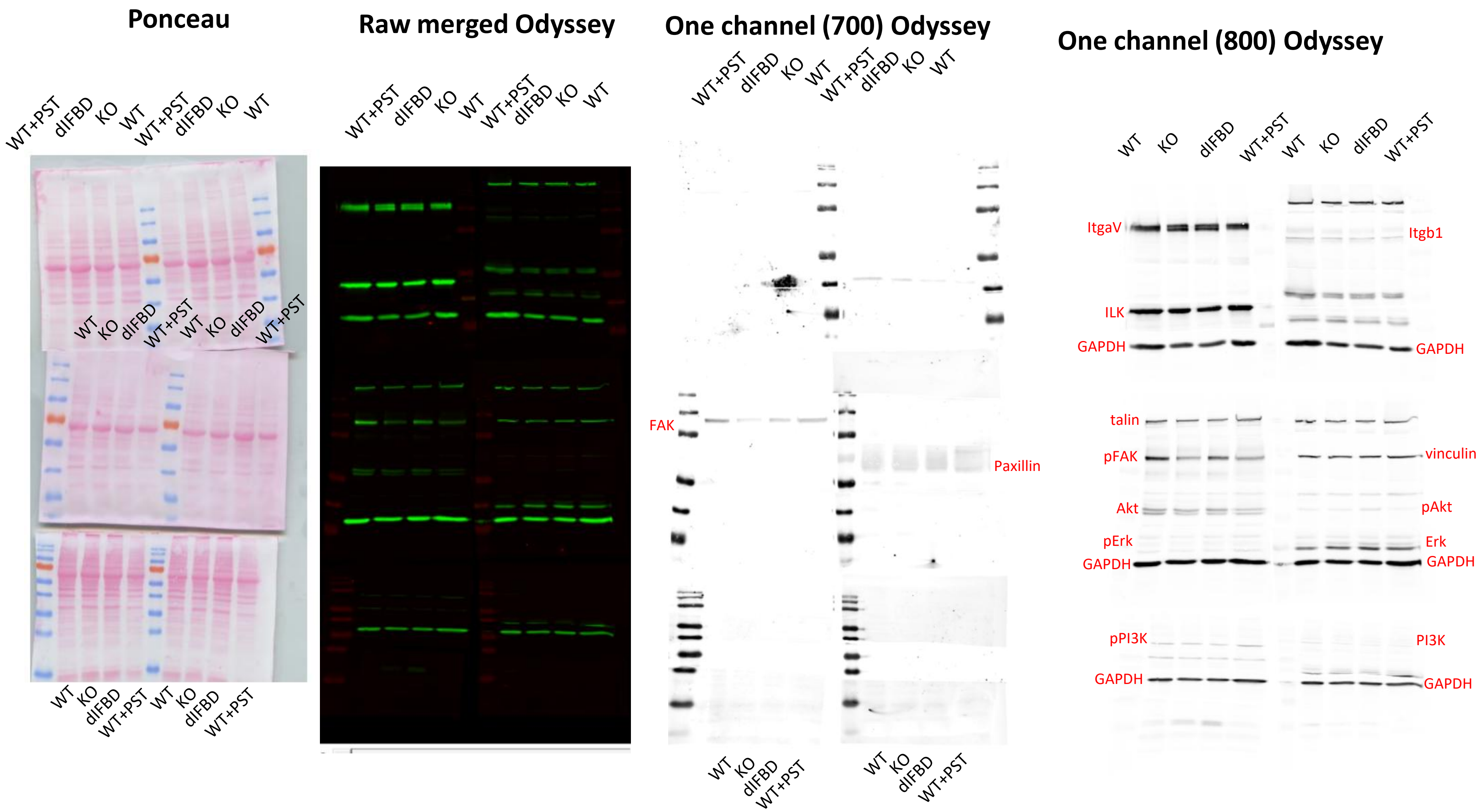

Huh7 replicate 7 (230325)

Figure 3D

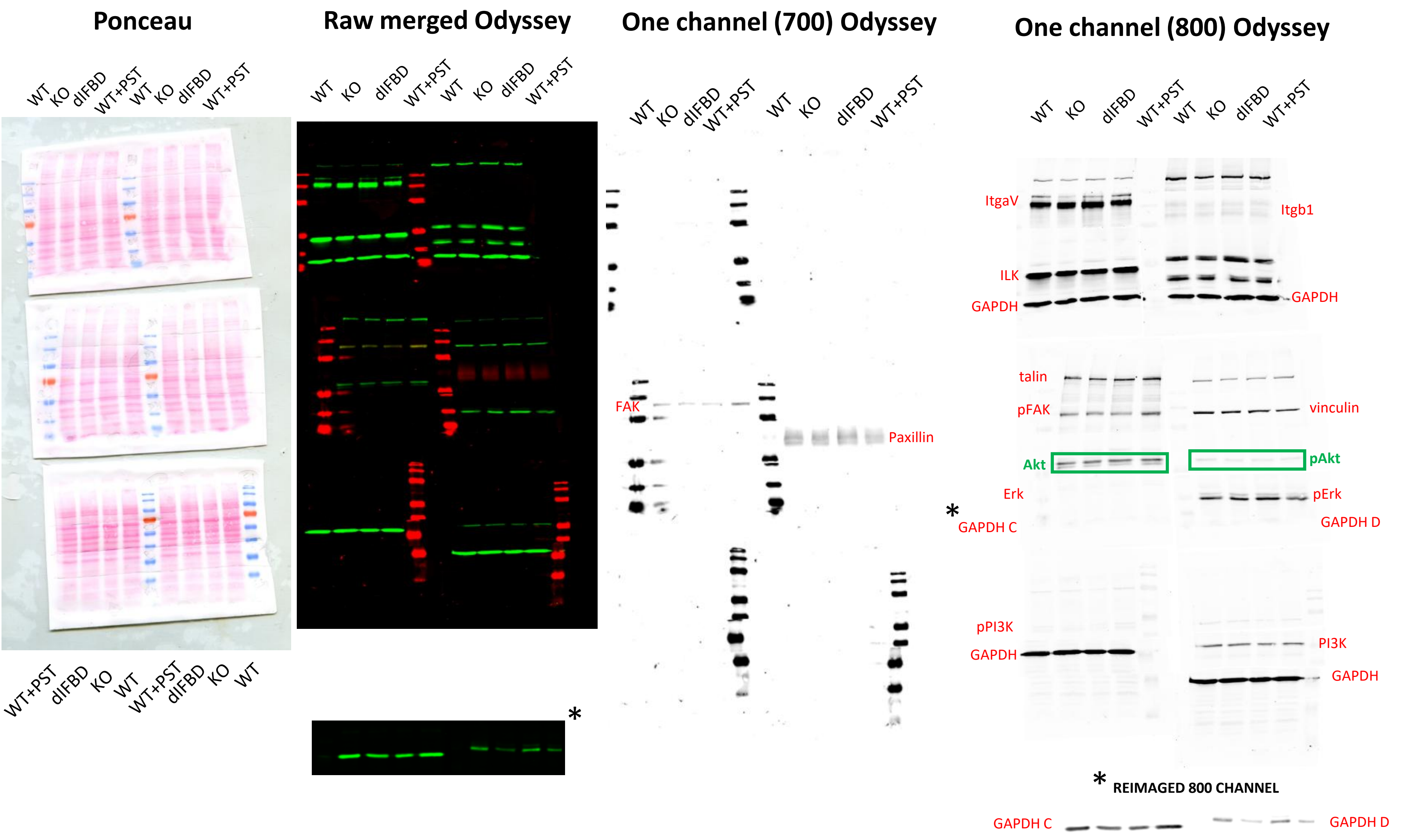

Huh7 replicate 8 (230328)

Ponceau

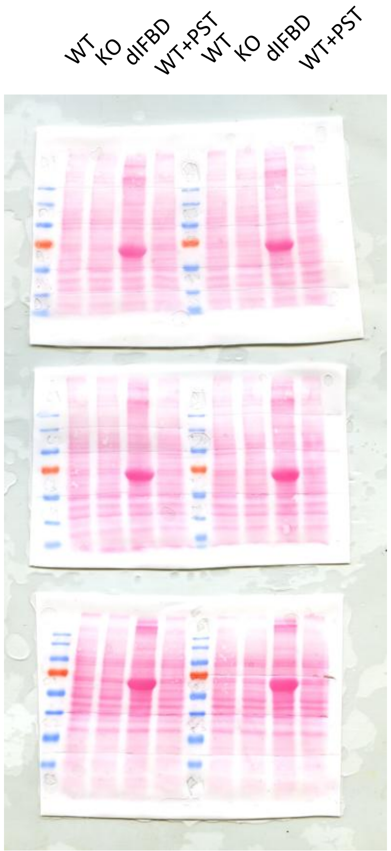

Marked

Raw merged Odyssey

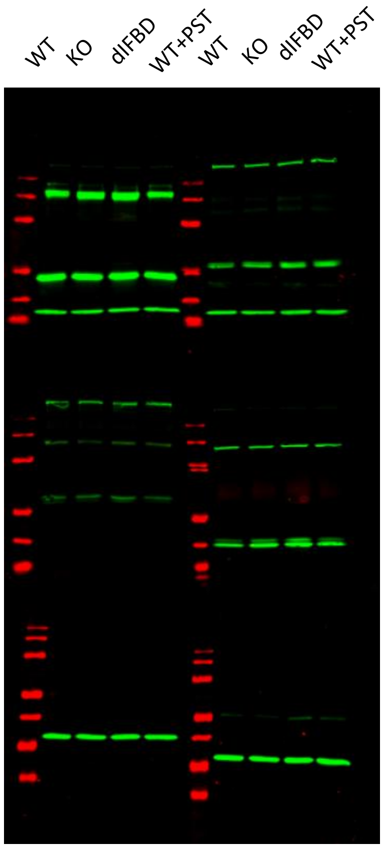

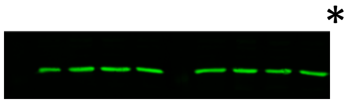

One channel (700) Odyssey

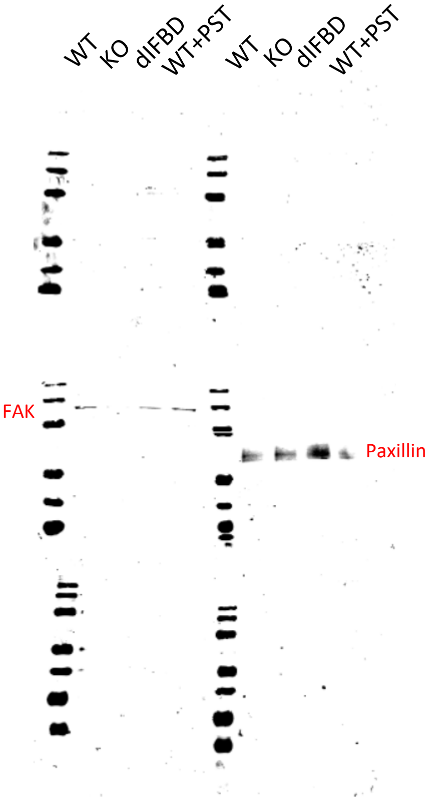

One channel (800) Odyssey

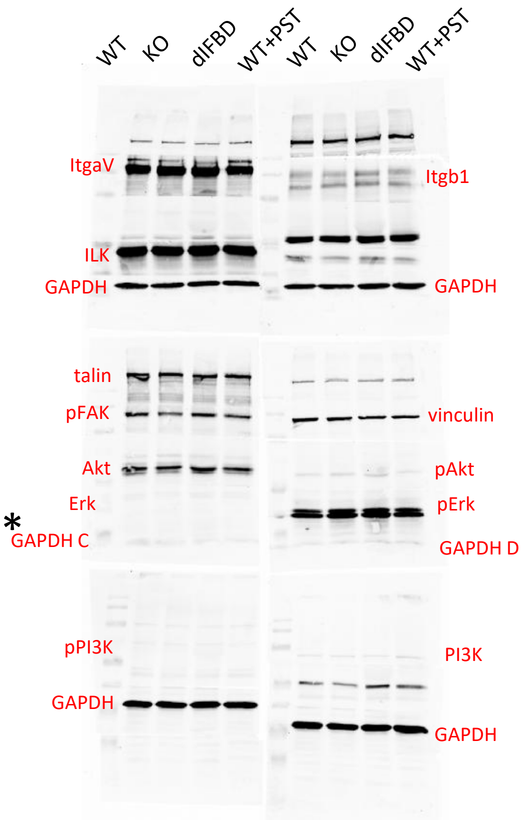

* **REIMAGED 800 CHANNEL**

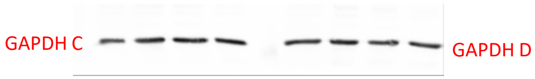

Huh7 replicate 9 (230331)

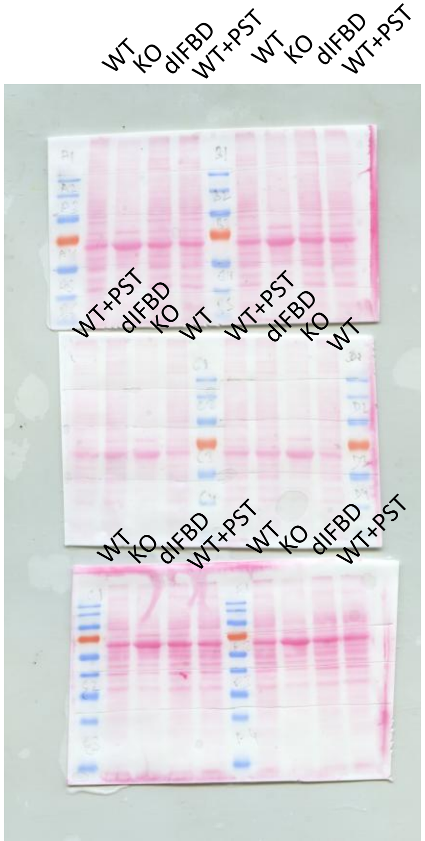

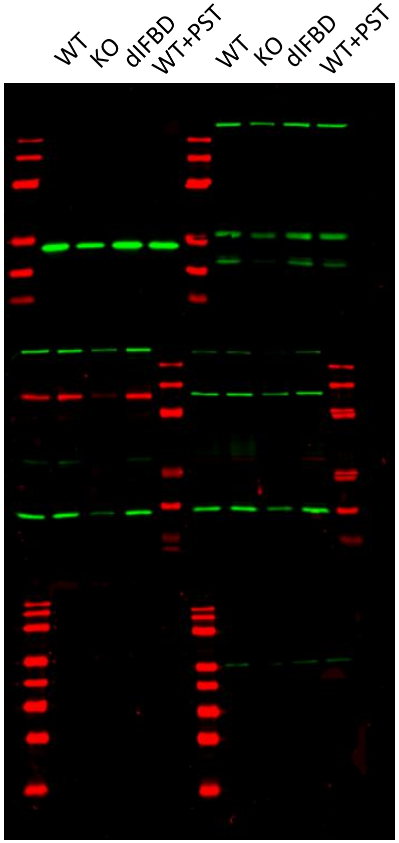

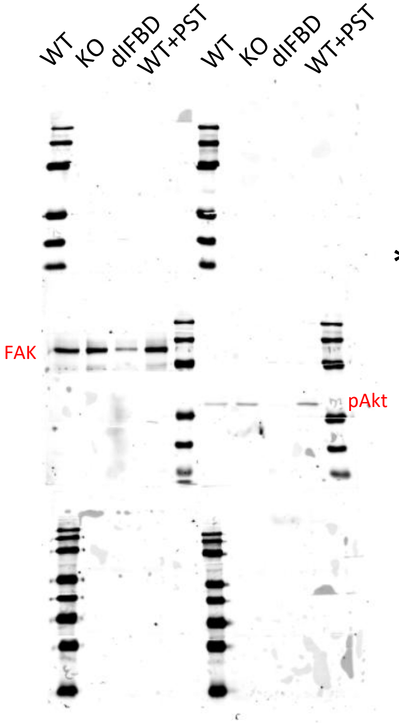

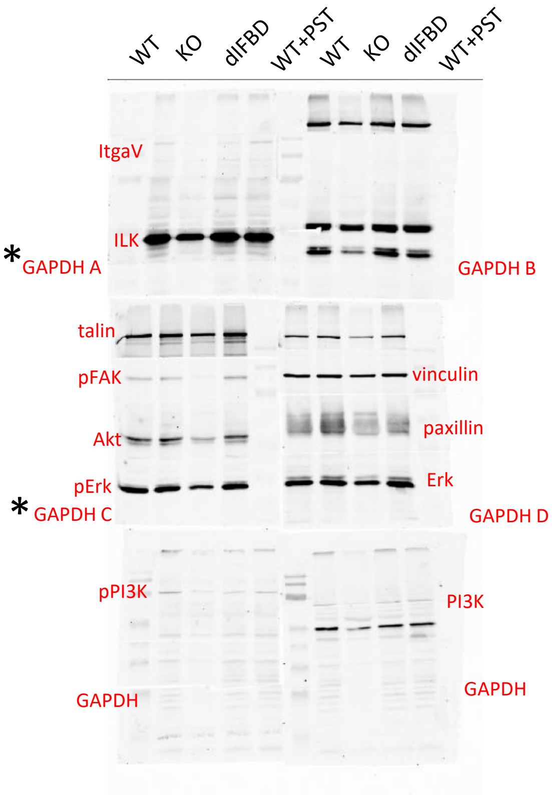

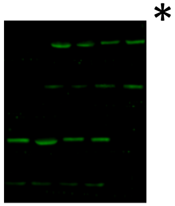

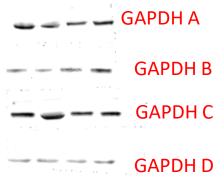

Supplement: Figure 3—source data 1. [file elife-102205-fig3-data1.pdf]
