# Supplementary figures and images for: Plectin-mediated cytoskeletal crosstalk as a target for inhibition of hepatocellular carcinoma growth and metastasis (part 2 of 2)
Source: eLife. 2025 Mar 7;13:RP102205. doi: 10.7554/eLife.102205 (PMC11893104; doi:10.7554/eLife.102205)

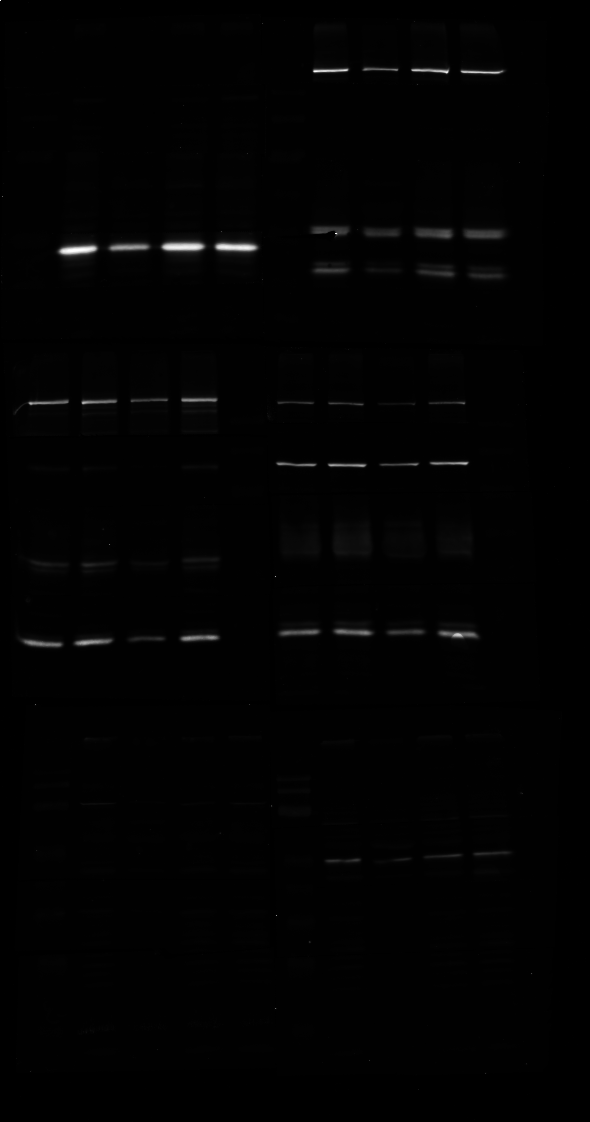

Supplement: Figure 3—source data 3. — Huh7 replicate 2–9. [file elife-102205-fig3-data3.zip › Figure 3-source data 3/230331_Huh7 9th WT KO IFBD WT+PST/230331_Huh7 8th WT KO IFBD WT+PST 800.tif]

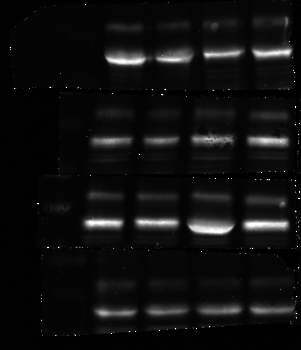

Supplement: Figure 3—source data 3. — Huh7 replicate 2–9. [file elife-102205-fig3-data3.zip › Figure 3-source data 3/230331_Huh7 9th WT KO IFBD WT+PST/230331_Huh7 8th WT KO IFBD WT+PST GAPDH ABCD.tif]

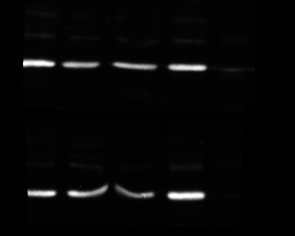

Supplement: Figure 3—source data 3. — Huh7 replicate 2–9. [file elife-102205-fig3-data3.zip › Figure 3-source data 3/230331_Huh7 9th WT KO IFBD WT+PST/230331_Huh7 8th WT KO IFBD WT+PST GAPDH.tif]

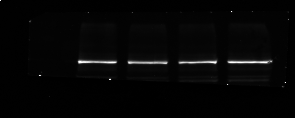

Supplement: Figure 3—source data 3. — Huh7 replicate 2–9. [file elife-102205-fig3-data3.zip › Figure 3-source data 3/230331_Huh7 9th WT KO IFBD WT+PST/230331_Huh7 8th WT KO IFBD WT+PST mTOR.tif]

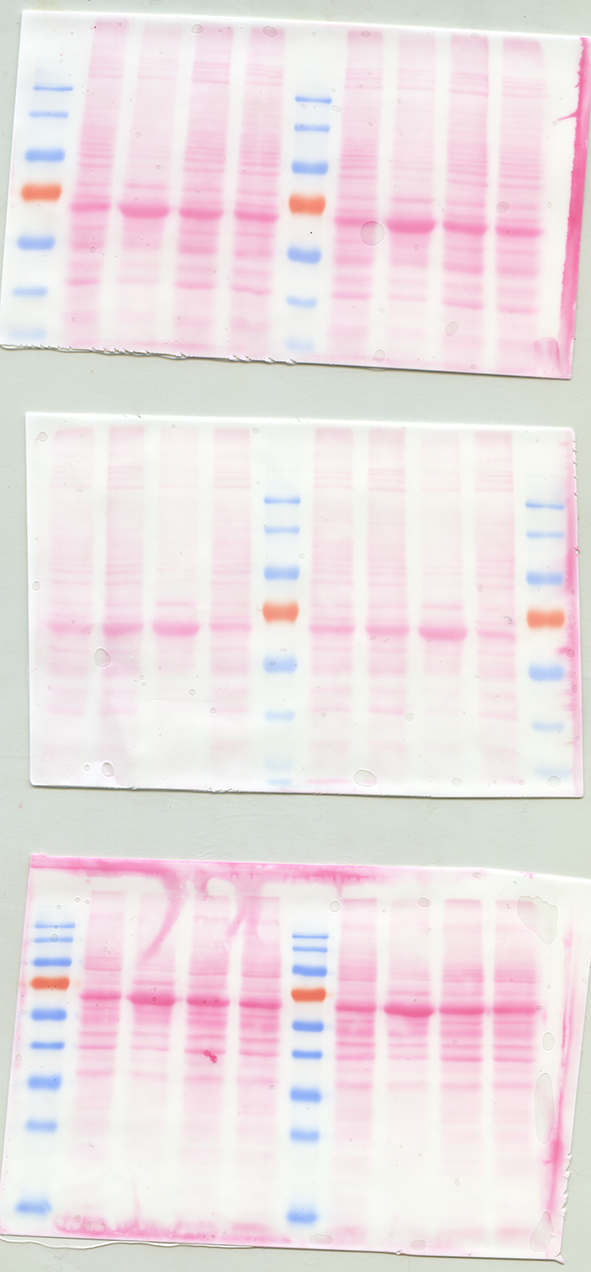

Supplement: Figure 3—source data 3. — Huh7 replicate 2–9. [file elife-102205-fig3-data3.zip › Figure 3-source data 3/230331_Huh7 9th WT KO IFBD WT+PST/230331_Huh7 9th WT KO IFBD WT+PST1.tif]

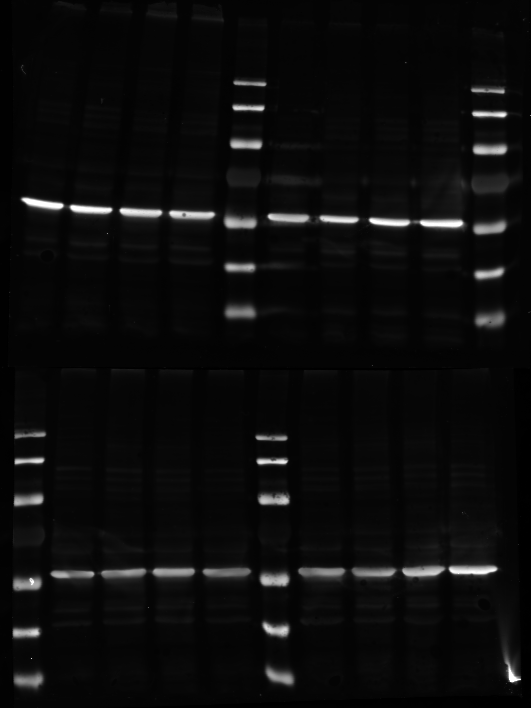

Supplement: Figure 5—figure supplement 1—source data 2. [file elife-102205-fig5-figsupp1-data2.zip › Figure 5-figure supplement 1-source data 2. Original files for western blot analysis displayed in Figure 5-figure supplement 1D/replicate 1-4/240126-700.tif]

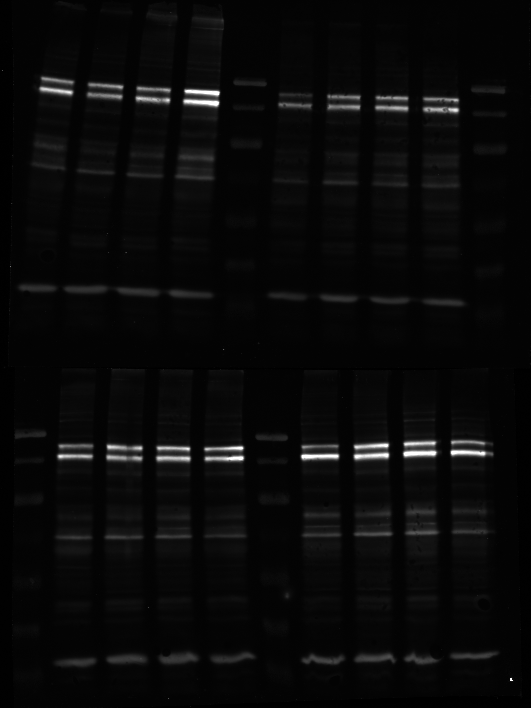

Supplement: Figure 5—figure supplement 1—source data 2. [file elife-102205-fig5-figsupp1-data2.zip › Figure 5-figure supplement 1-source data 2. Original files for western blot analysis displayed in Figure 5-figure supplement 1D/replicate 1-4/240126-800.tif]

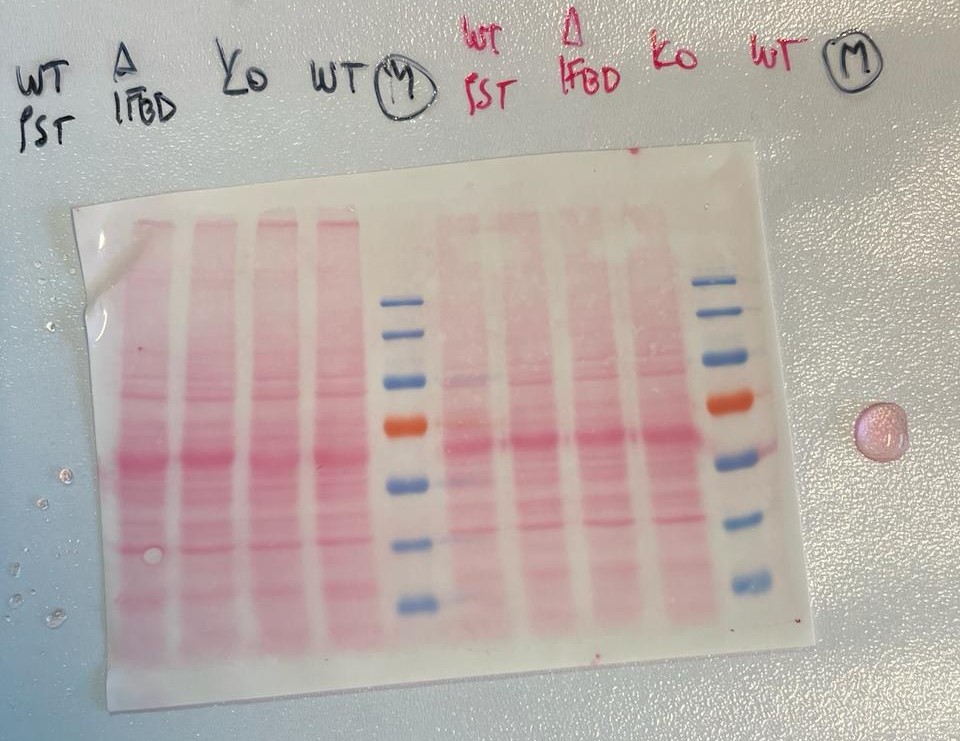

Supplement: Figure 5—figure supplement 1—source data 2. [file elife-102205-fig5-figsupp1-data2.zip › Figure 5-figure supplement 1-source data 2. Original files for western blot analysis displayed in Figure 5-figure supplement 1D/replicate 1-4/ponceau 1.jpeg]

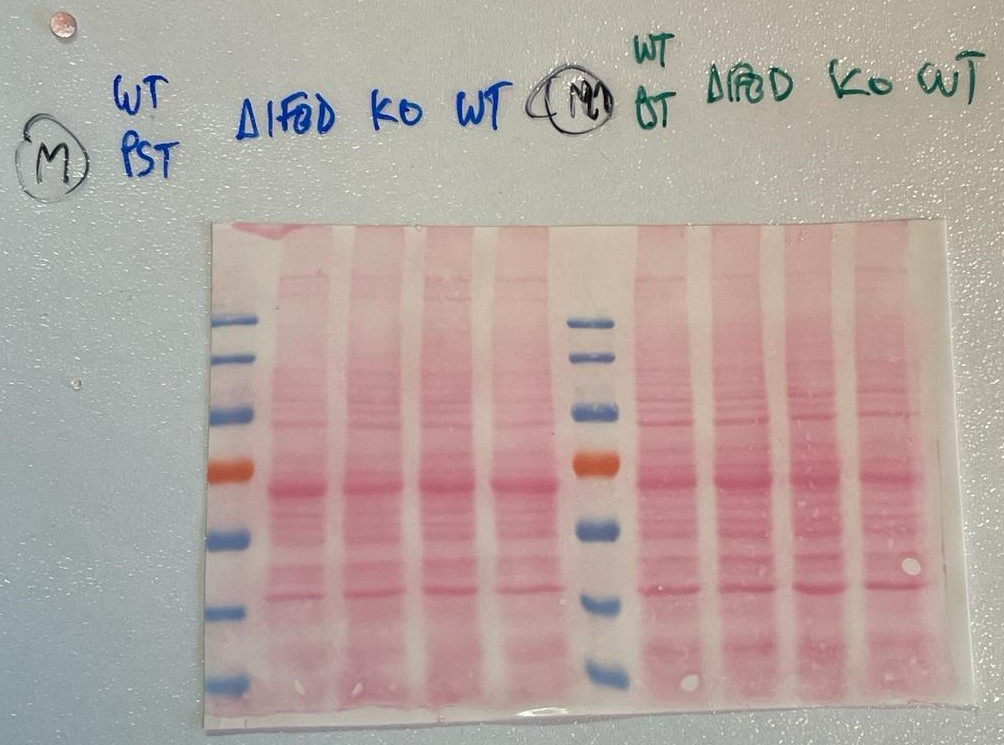

Supplement: Figure 5—figure supplement 1—source data 2. [file elife-102205-fig5-figsupp1-data2.zip › Figure 5-figure supplement 1-source data 2. Original files for western blot analysis displayed in Figure 5-figure supplement 1D/replicate 1-4/ponceau 2.jpeg]

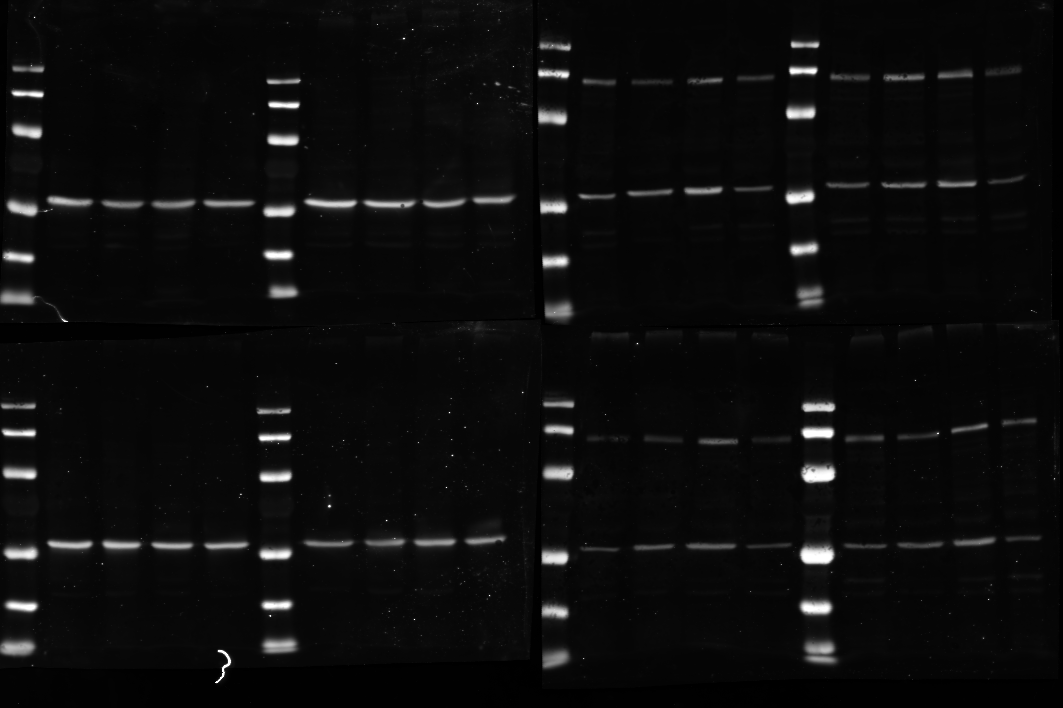

Supplement: Figure 5—figure supplement 1—source data 2. [file elife-102205-fig5-figsupp1-data2.zip › Figure 5-figure supplement 1-source data 2. Original files for western blot analysis displayed in Figure 5-figure supplement 1D/replicate 5-8/240201-700.tiff]

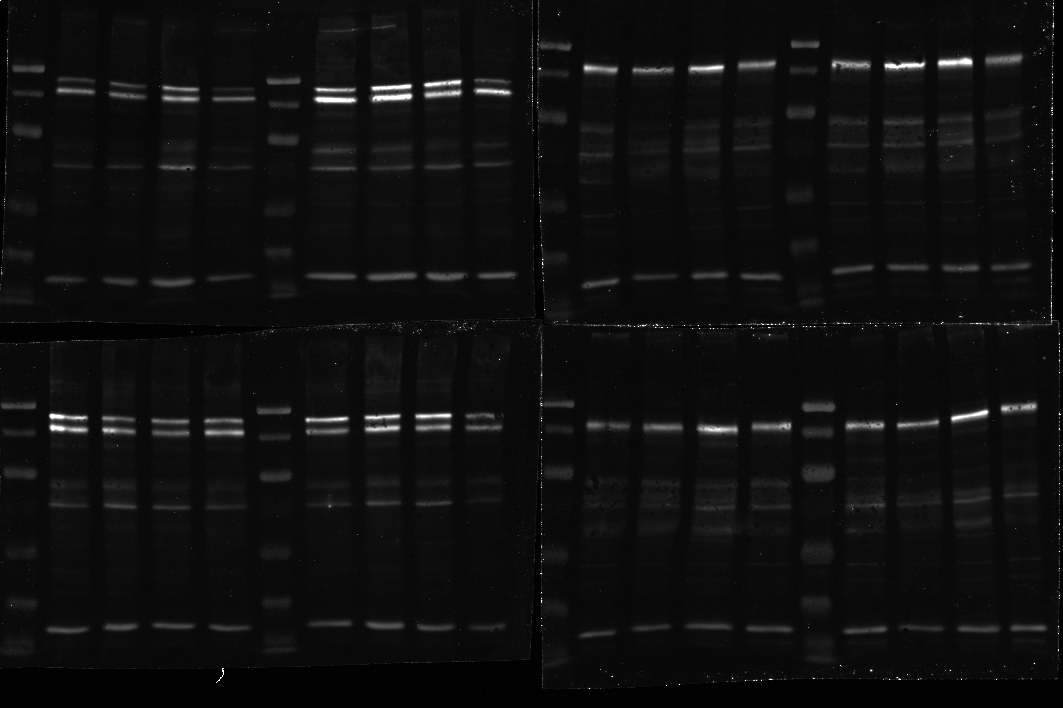

Supplement: Figure 5—figure supplement 1—source data 2. [file elife-102205-fig5-figsupp1-data2.zip › Figure 5-figure supplement 1-source data 2. Original files for western blot analysis displayed in Figure 5-figure supplement 1D/replicate 5-8/240201-800.tiff]

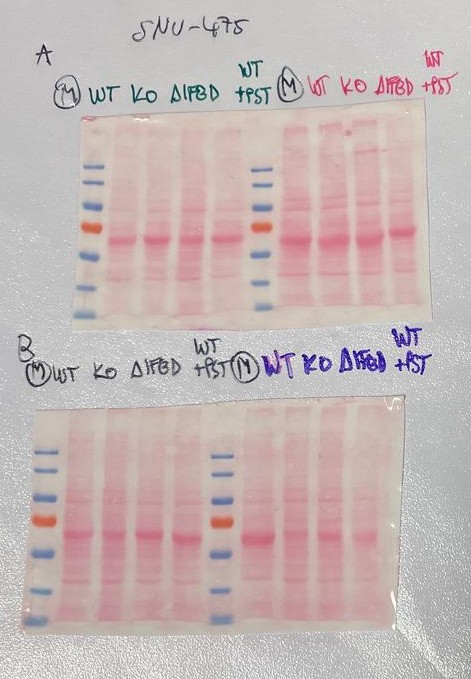

Supplement: Figure 5—figure supplement 1—source data 2. [file elife-102205-fig5-figsupp1-data2.zip › Figure 5-figure supplement 1-source data 2. Original files for western blot analysis displayed in Figure 5-figure supplement 1D/replicate 5-8/ponceau 240130.jpeg]
