# Supplementary material for: Plectin-mediated cytoskeletal crosstalk as a target for inhibition of hepatocellular carcinoma growth and metastasis
Source: eLife. 2025 Mar 7;13:RP102205. doi: 10.7554/eLife.102205 (PMC11893104; doi:10.7554/eLife.102205)

SNU-475 replicate 1 (221209)

Figure 3 – figure supplement 1C

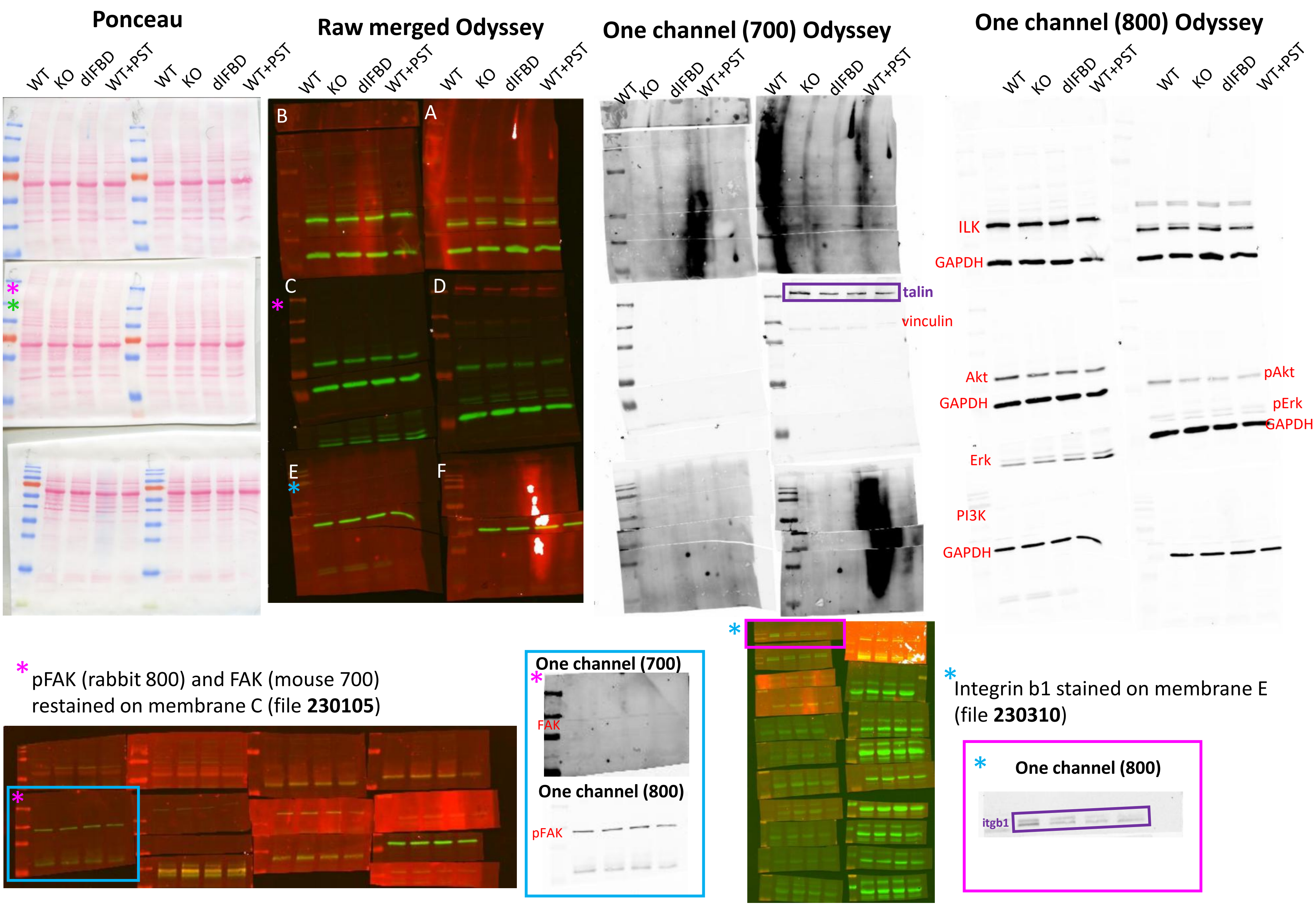

SNU-475 replicate 2 and 3 (221216)

Figure 3 – figure supplement 1C

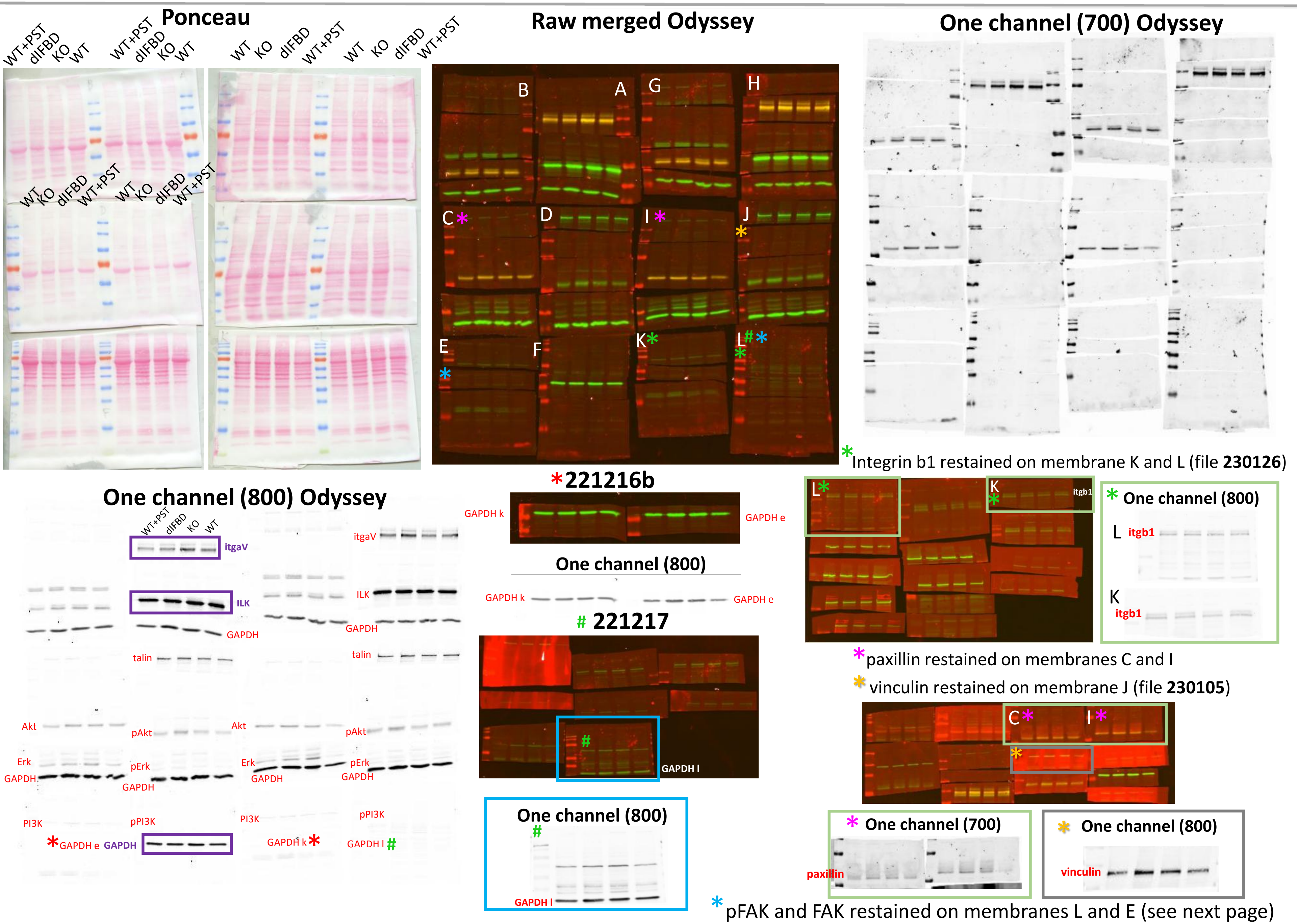

SNU-475 replicate 4 and 5 (221221)

Figure 3 – figure supplement 1C

Ponceau

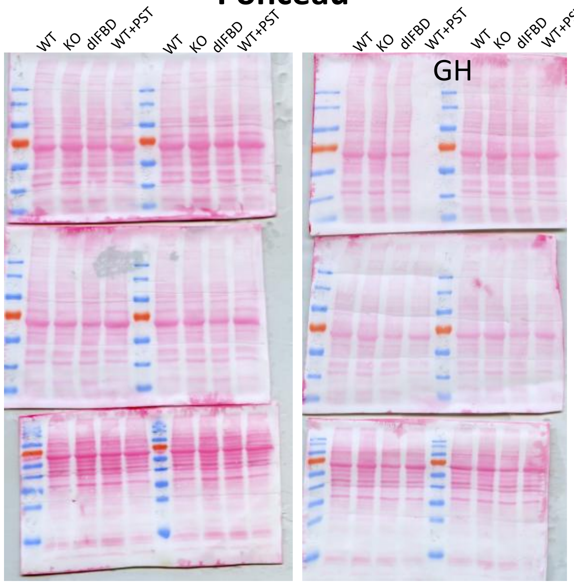

Raw merged Odyssey

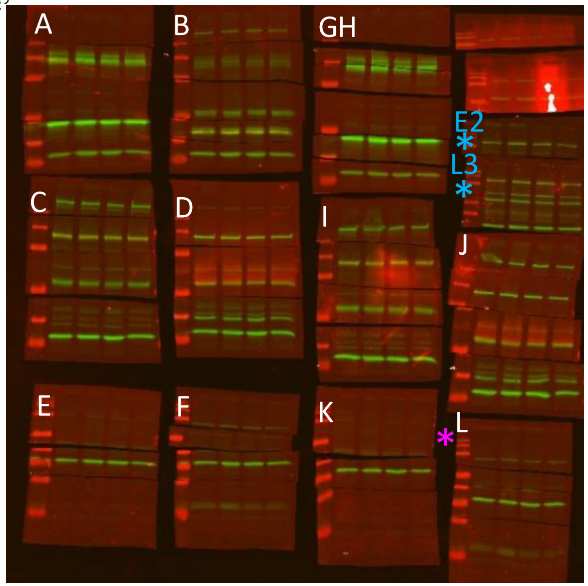

One channel (700) Odyssey

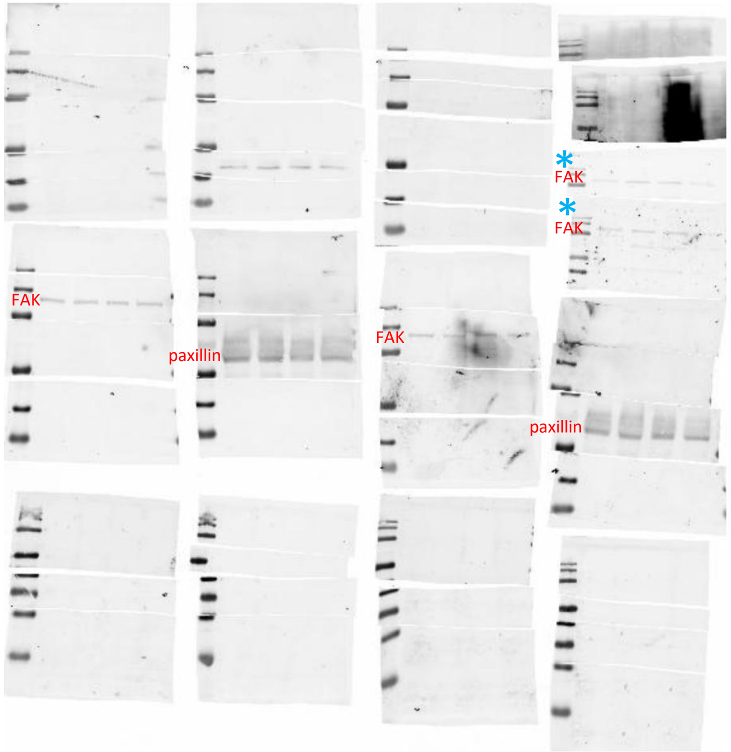

One channel (800) Odyssey

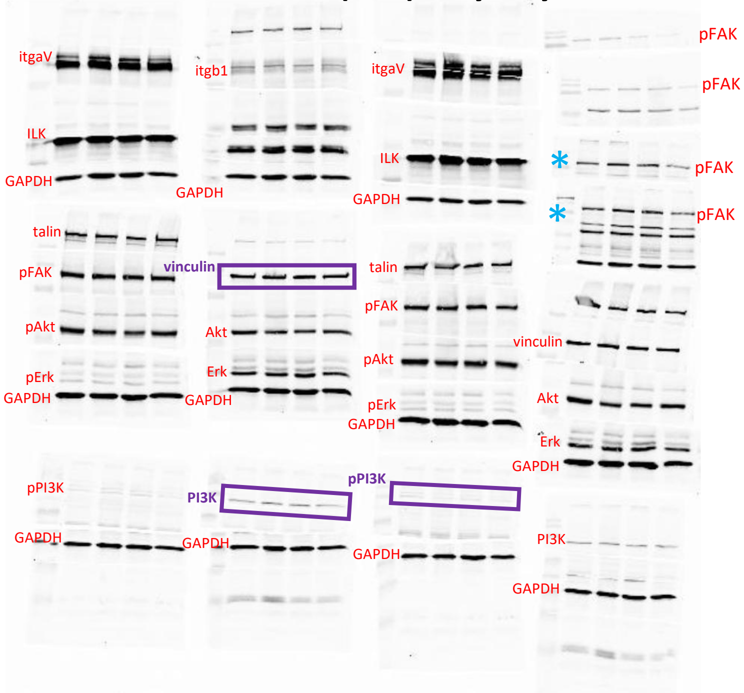

* Integrin b1 of 5th replicate stained on membrane L (file **230126**)

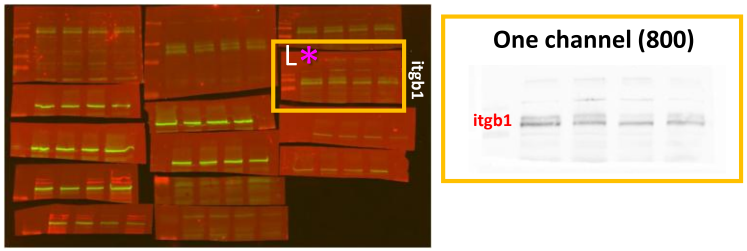

* pFAK and FAK from **221216** restained on membrane E and L (see * on previous page).

Huh7 replicate 1 and SNU-475 replicate 6 (230125)

Ponceau

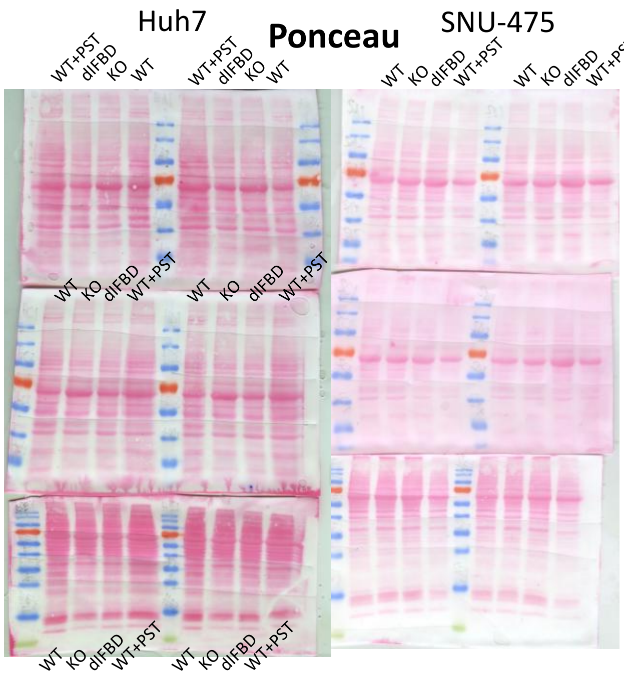

Raw merged Odyssey

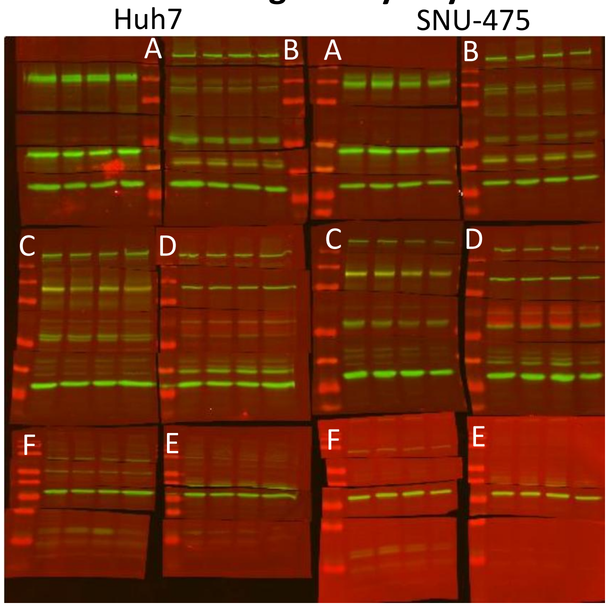

One channel (700) Odyssey

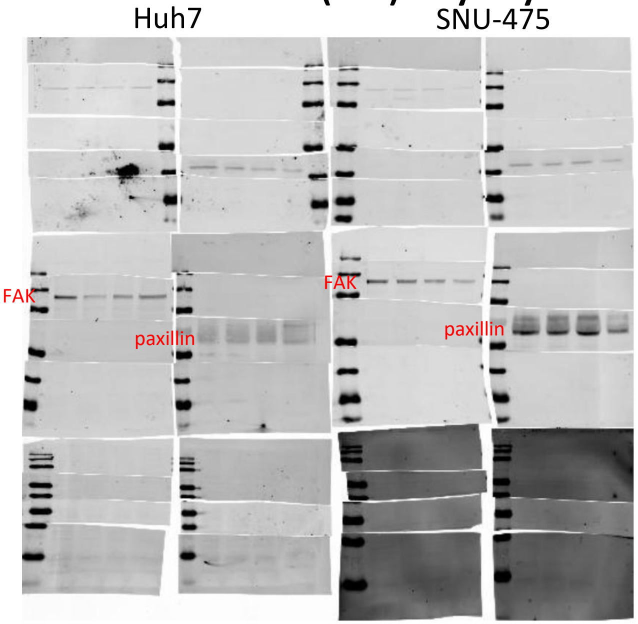

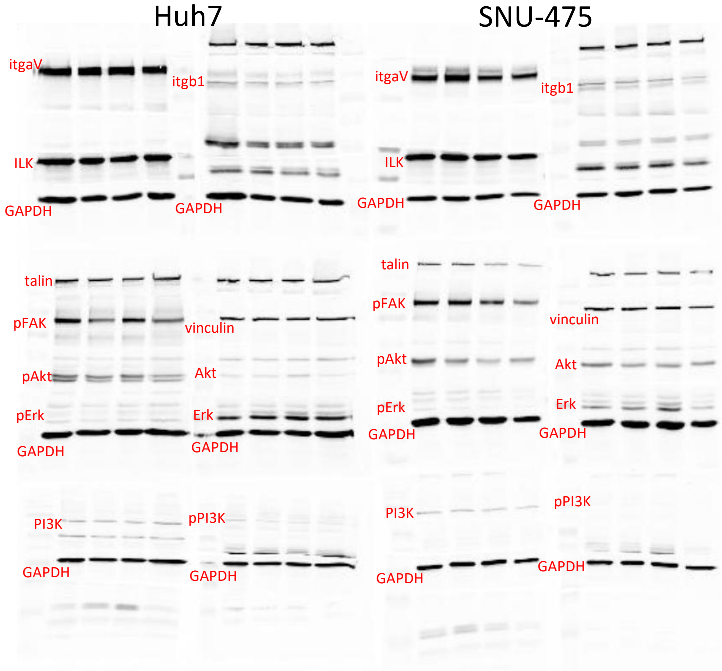

SNU-475 replicate 7 and 8 (230224)

Figure 3 – figure supplement 1C

7 Ponceau 8

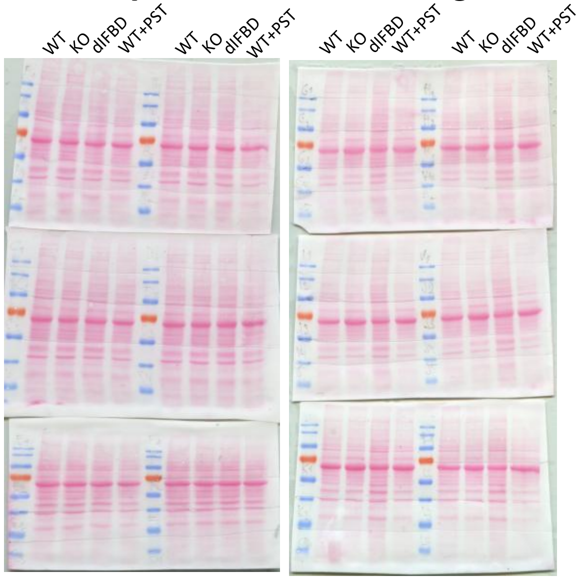

7 Raw merged Odyssey 8

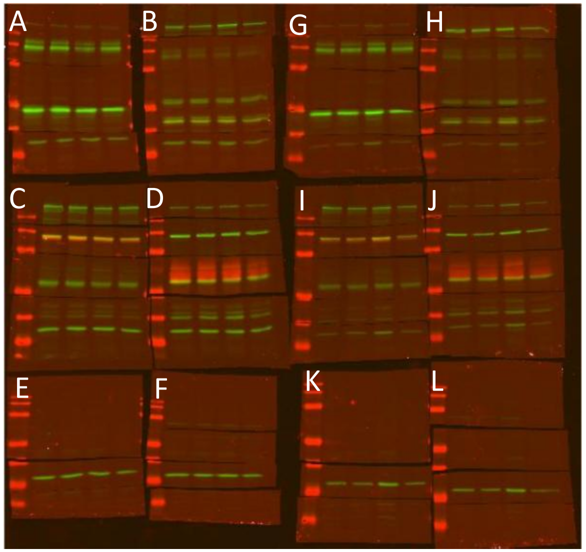

One channel (700) Odyssey

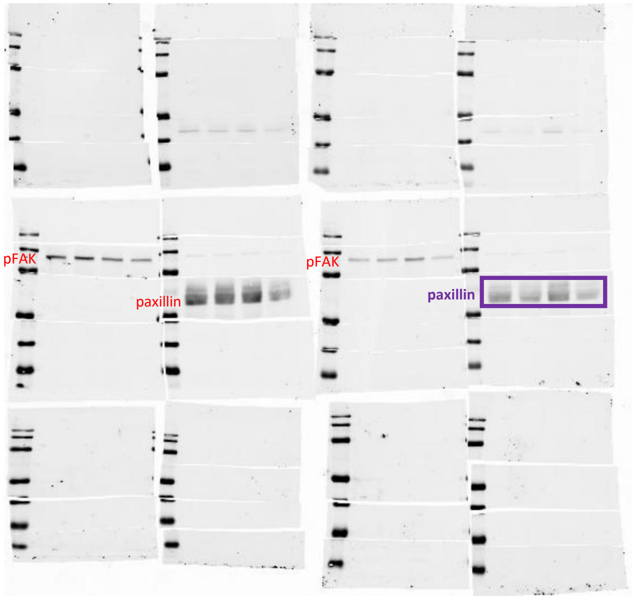

One channel (800) Odyssey

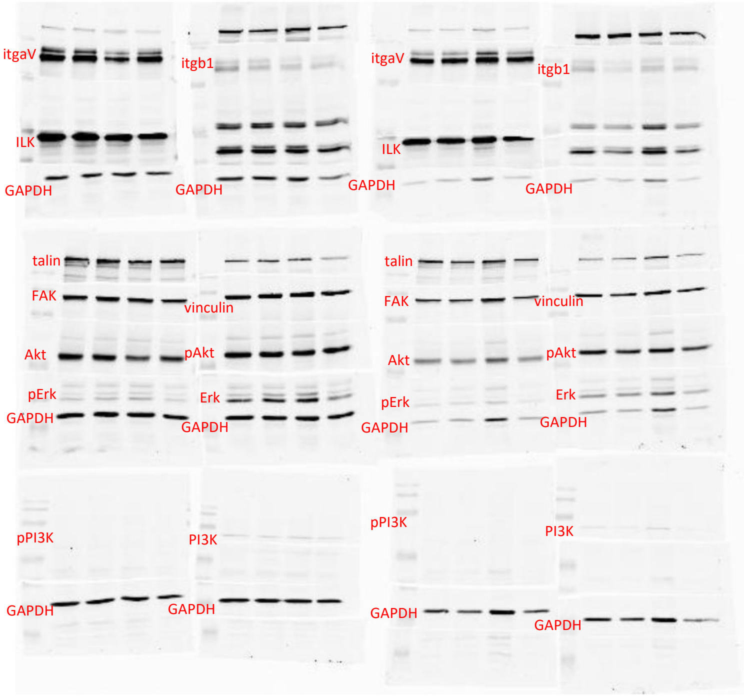

SNU-475 replicate 9 (230305)

Ponceau

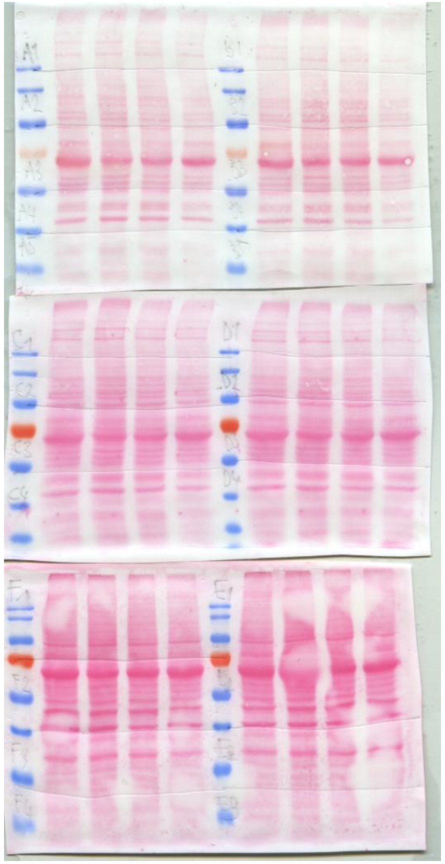

Raw merged Odyssey

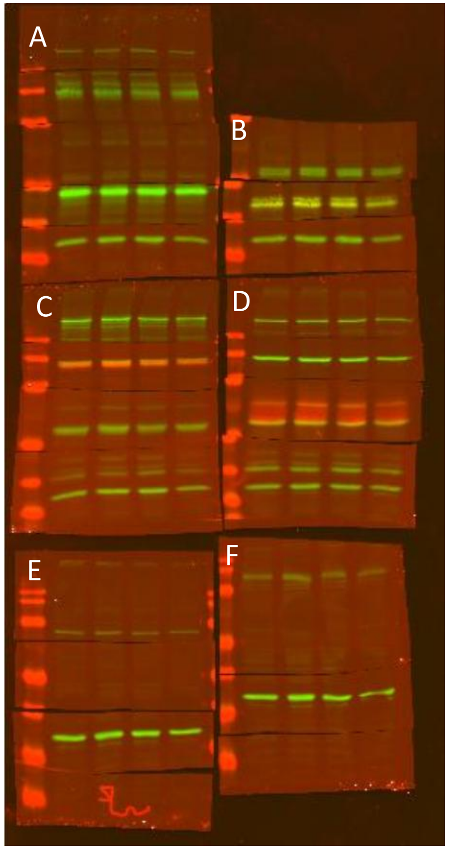

One channel (700) Odyssey

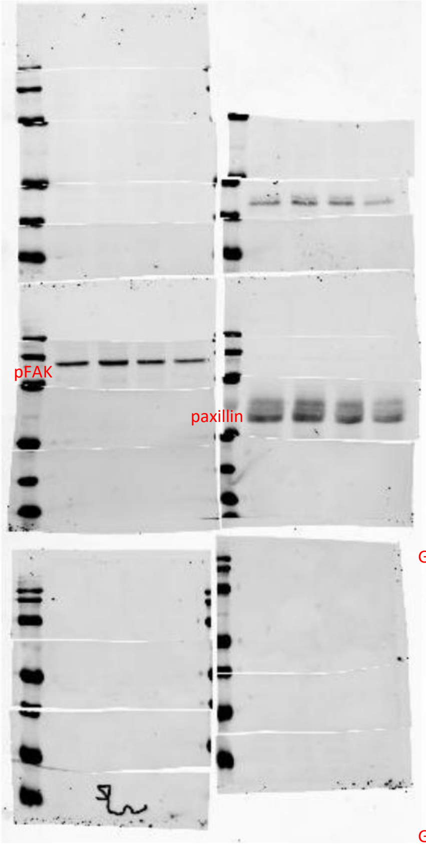

One channel (800) Odyssey

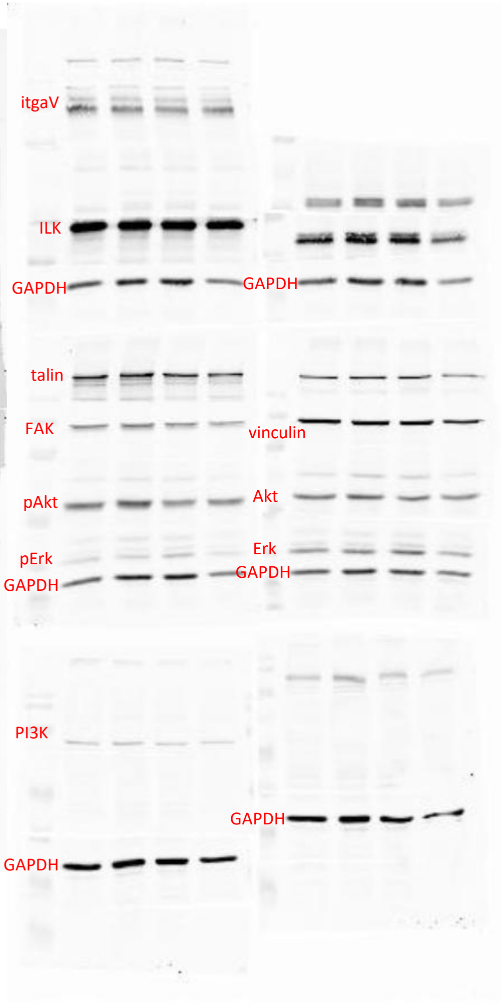

230308

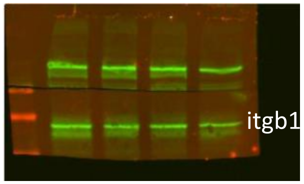

One channel (800) Odyssey

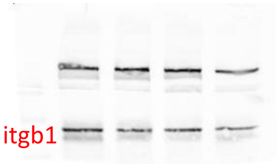

Huh7 replicate 2 (230113)

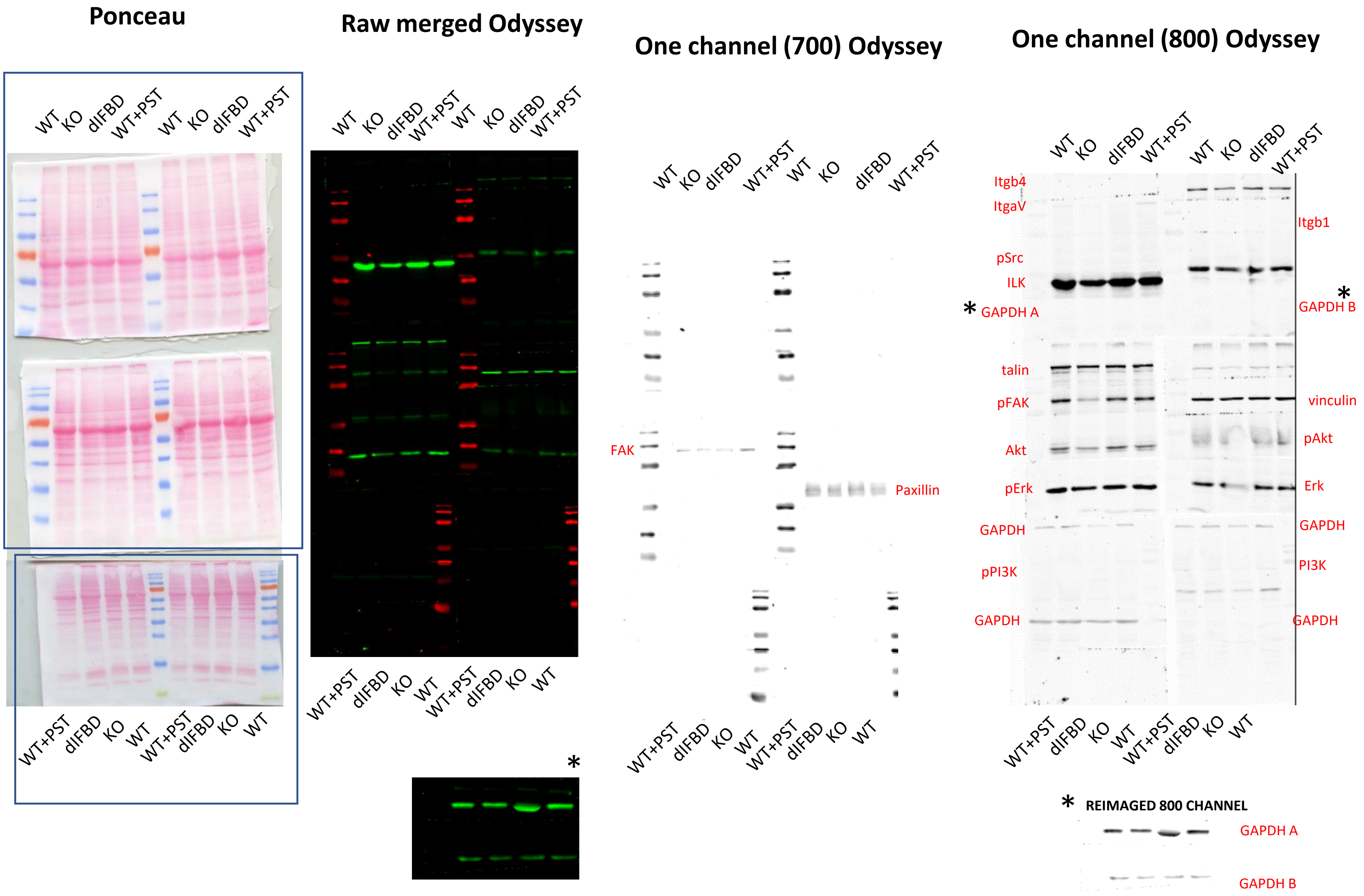

Huh7 replicate 3 (230117)

Ponceau

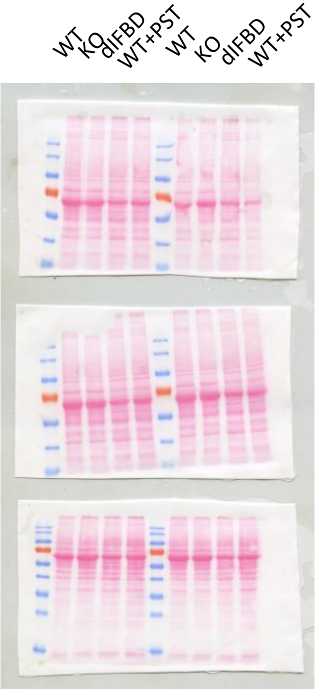

Raw merged Odyssey

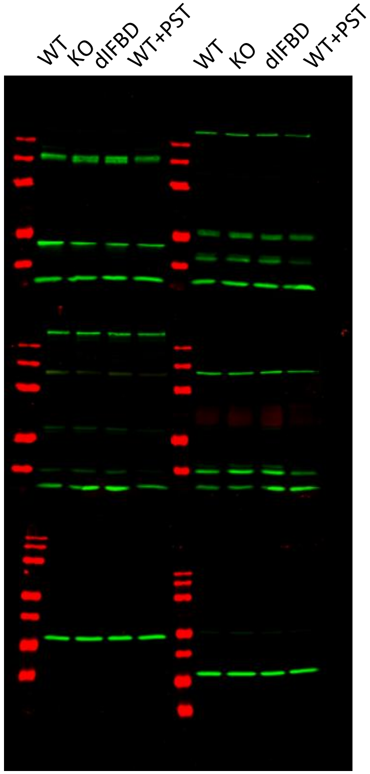

One channel (700) Odyssey

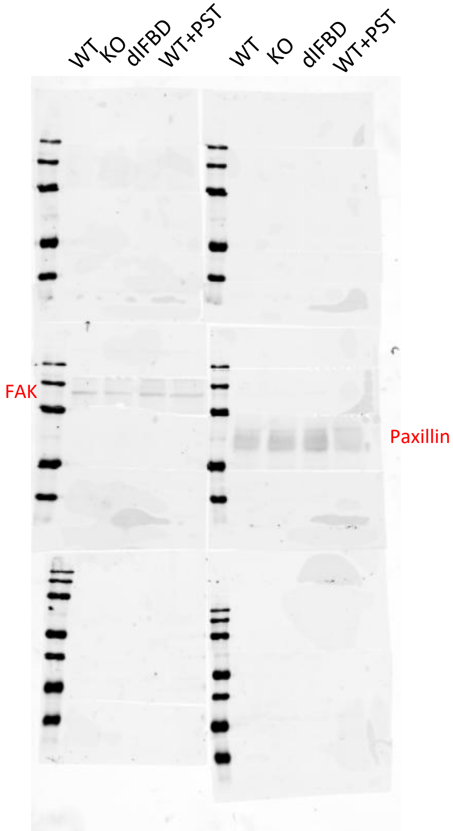

One channel (800) Odyssey

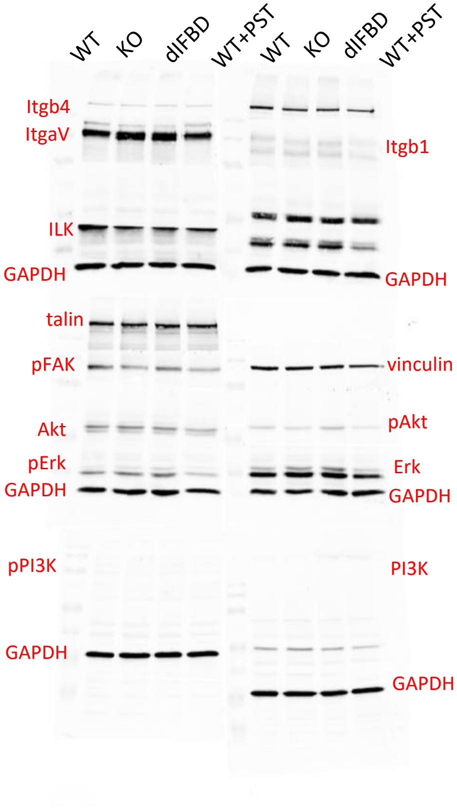

Huh7 replicate 4 (230120)

Figure 3 – figure supplement 1C

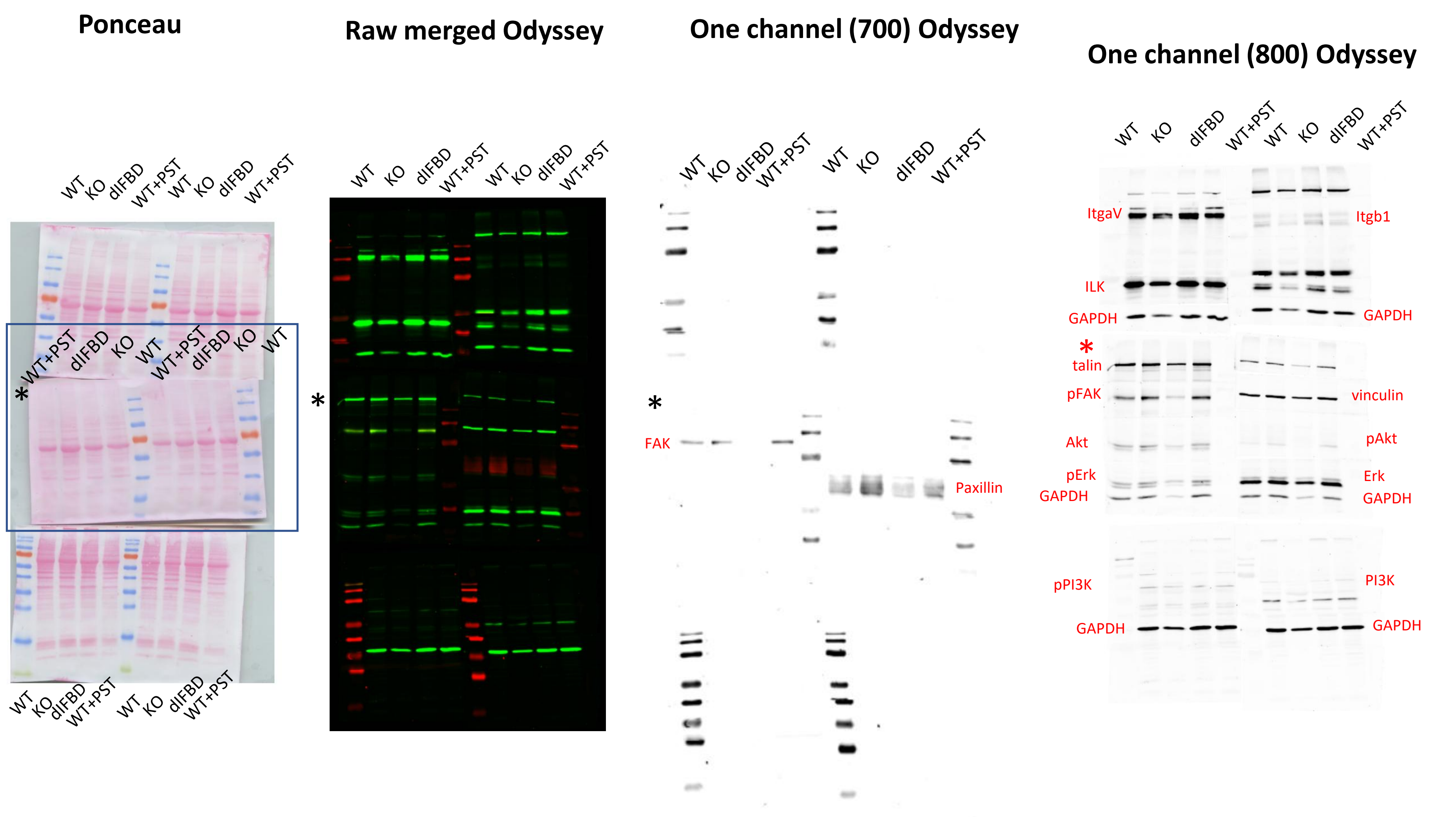

Huh7 replicate 5 (230124)

Figure 3 – figure supplement 1C

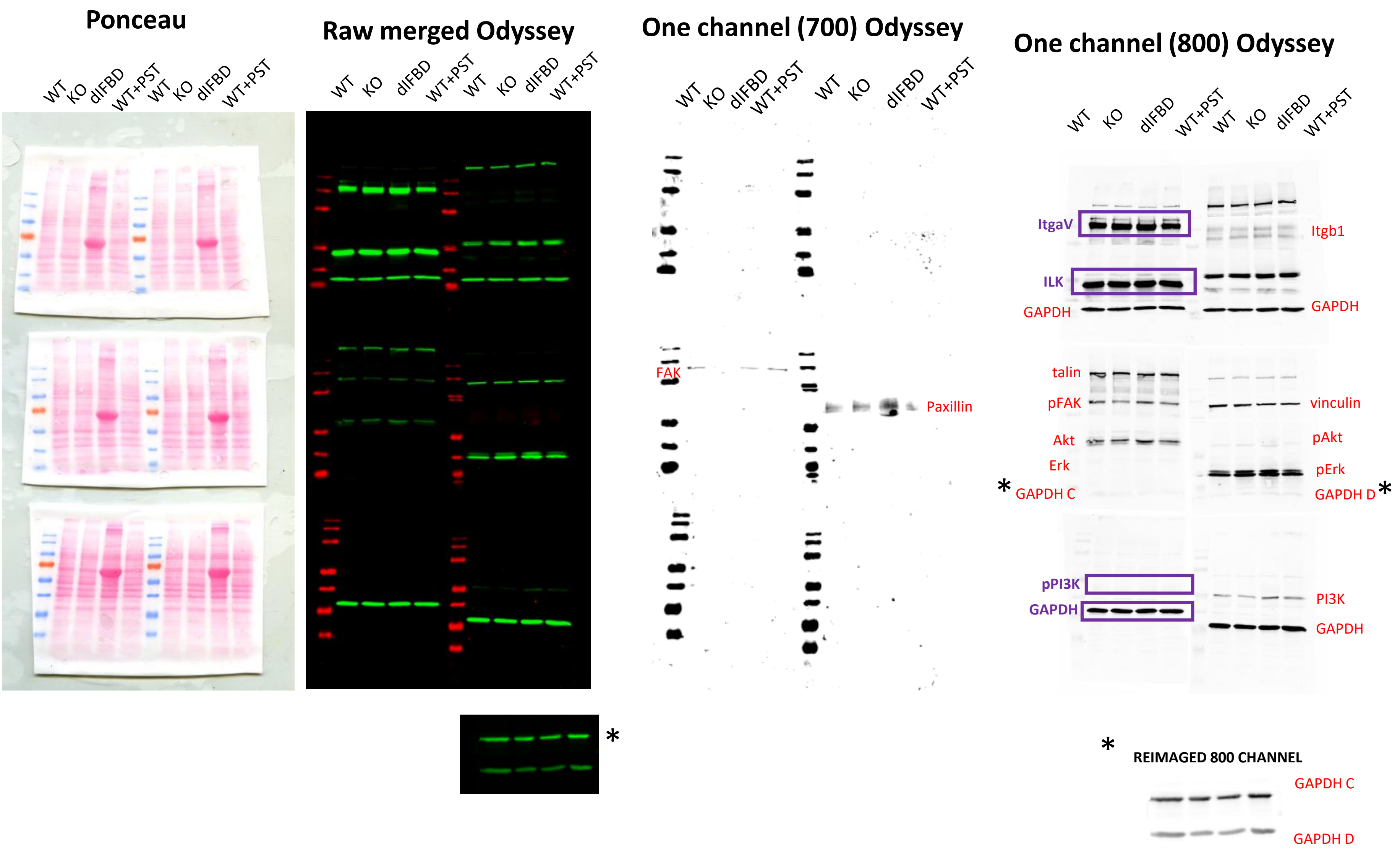

Huh7 replicate 6 (230301)

Figure 3 – figure supplement 1C

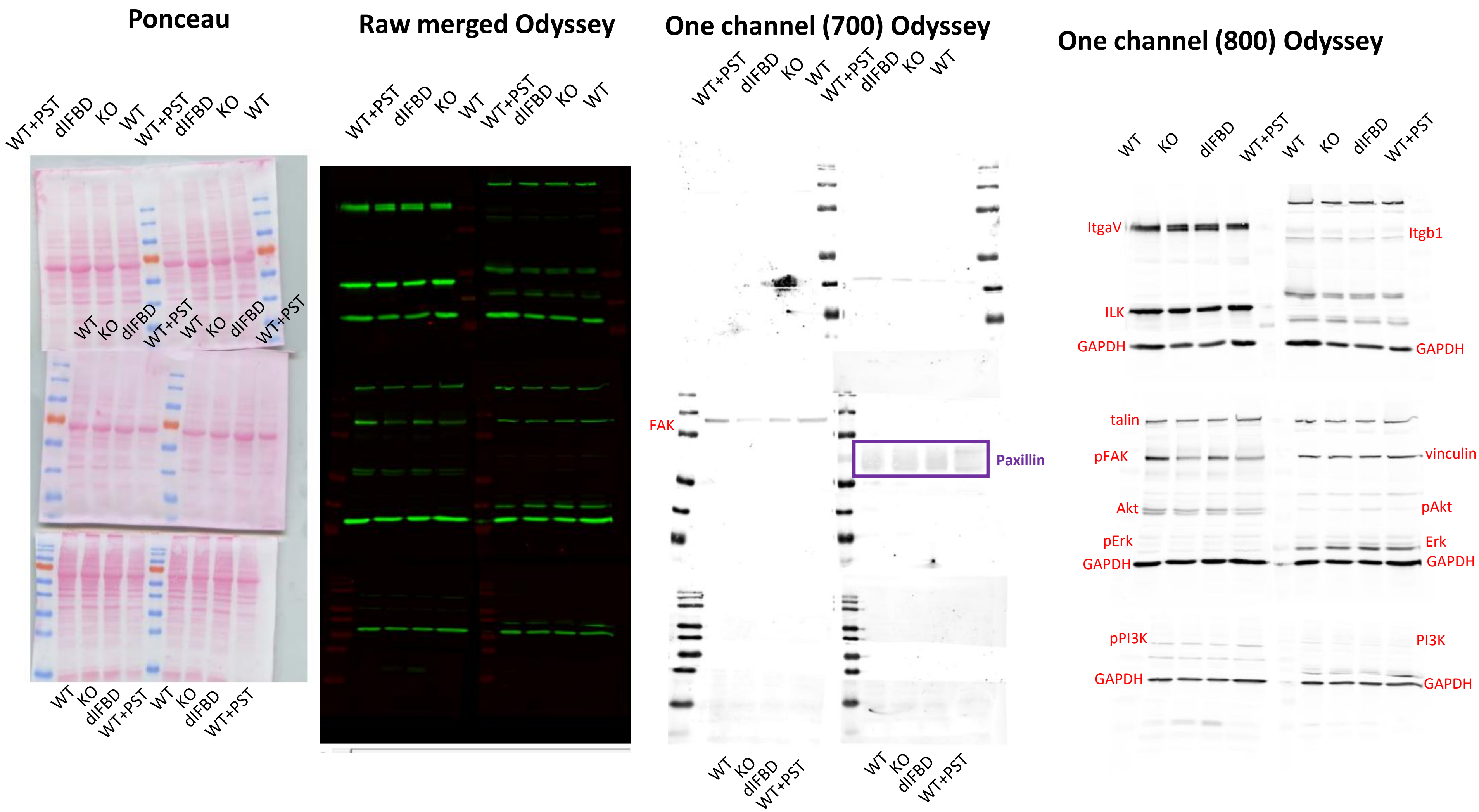

Huh7 replicate 7 (230325)

Figure 3 – figure supplement 1C

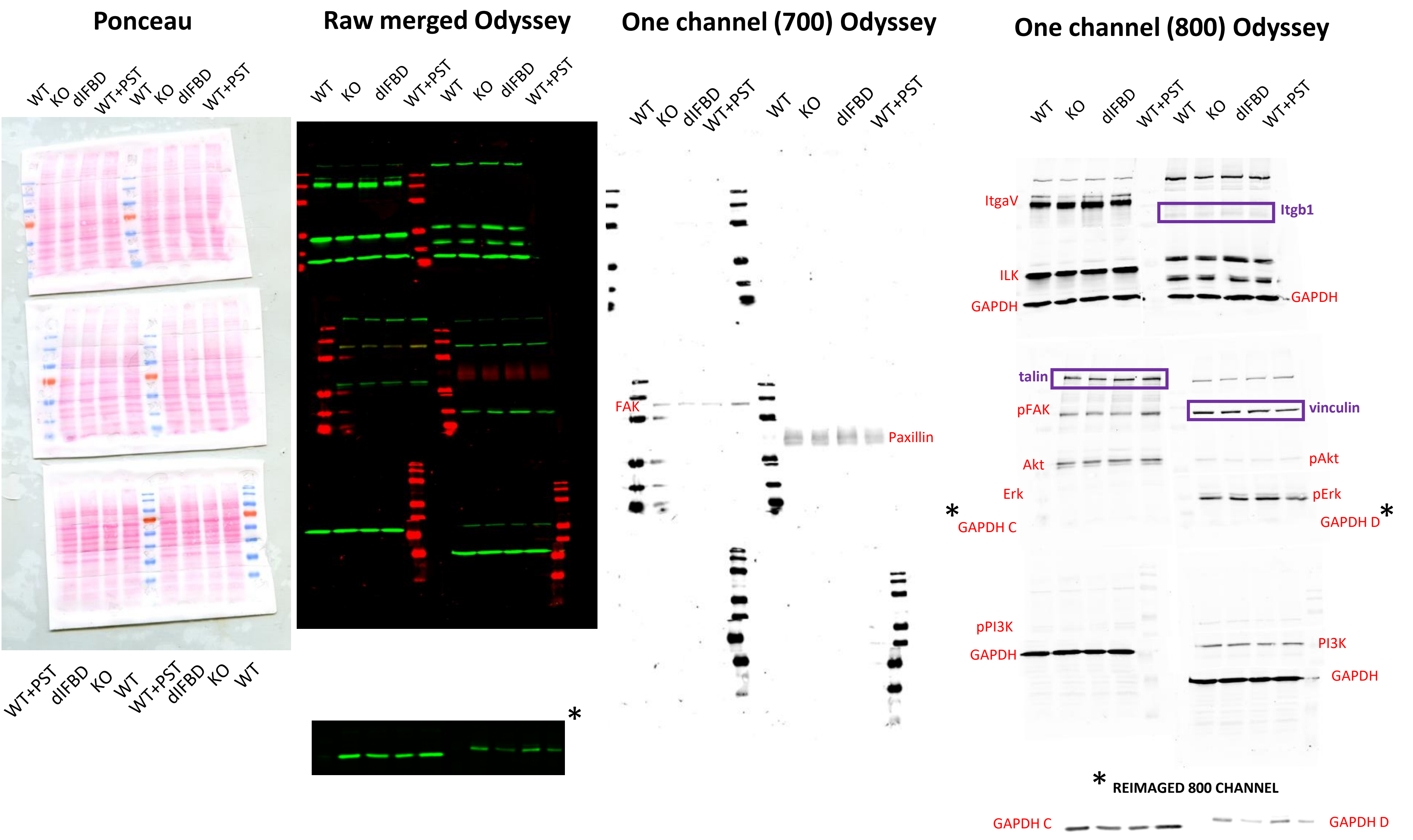

Huh7 replicate 8 (230328)

Figure 3 – figure supplement 1C

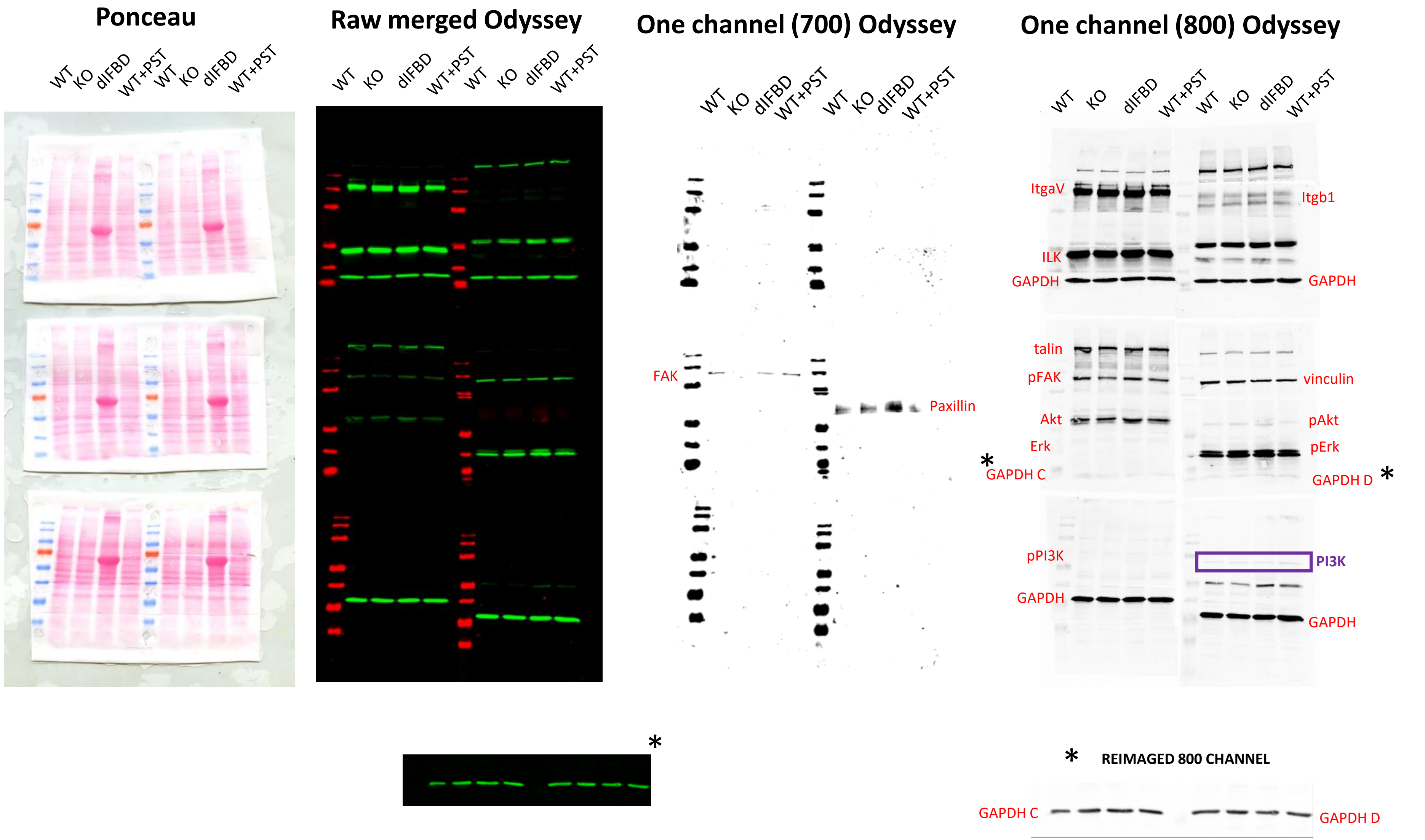

Huh7 replicate 9 (230331)

Ponceau

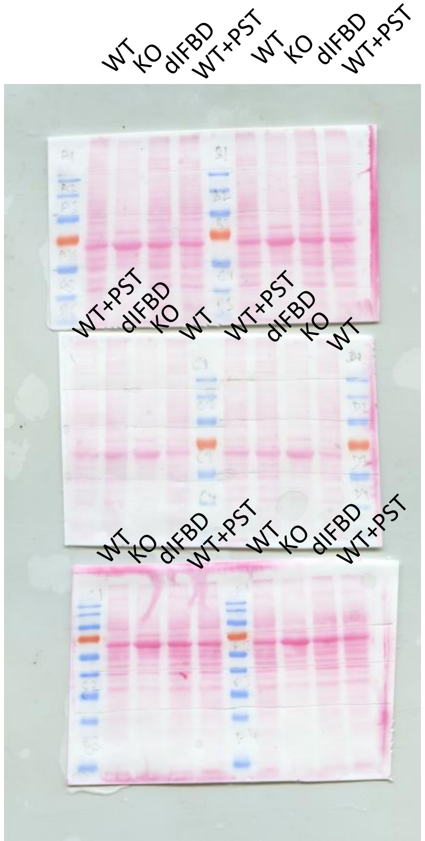

Raw merged Odyssey

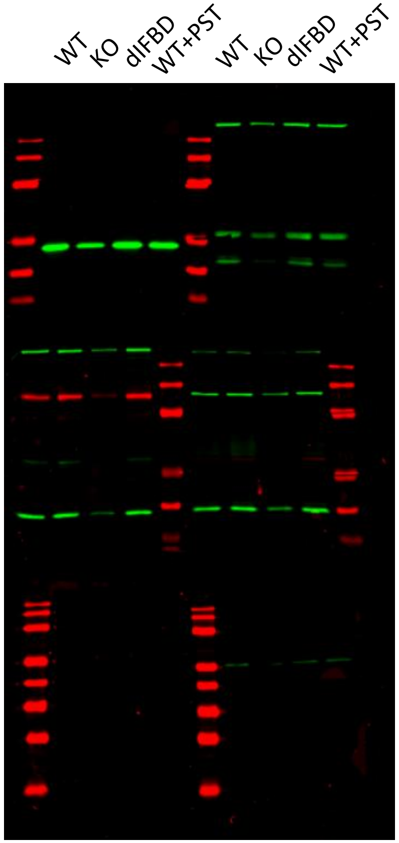

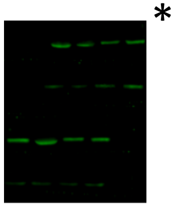

One channel (700) Odyssey

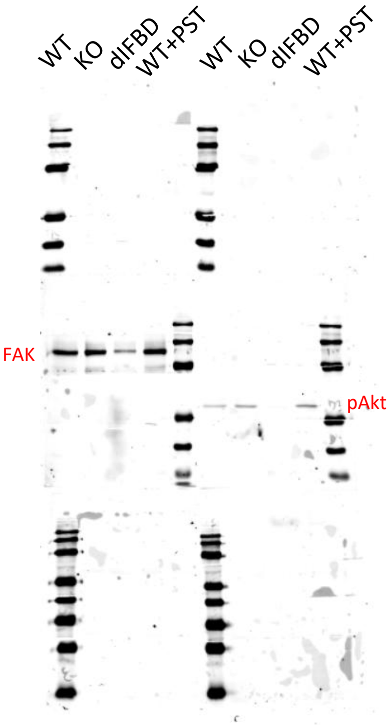

One channel (800) Odyssey

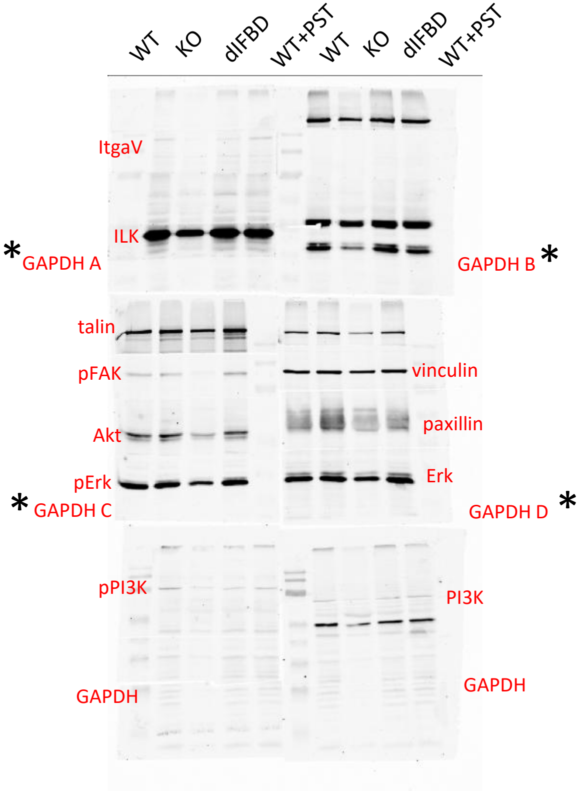

* reimaged GAPDH

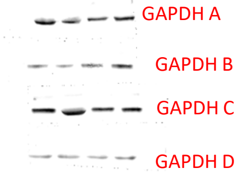

Supplement: Figure 3—figure supplement 1—source data 1. [file elife-102205-fig3-figsupp1-data1.pdf]
