# Supplementary material for: Plectin-mediated cytoskeletal crosstalk as a target for inhibition of hepatocellular carcinoma growth and metastasis
Source: eLife. 2025 Mar 7;13:RP102205. doi: 10.7554/eLife.102205 (PMC11893104; doi:10.7554/eLife.102205)

Figure 5 – figure supplement 1C

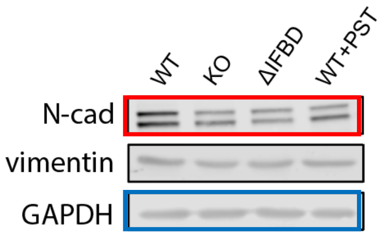

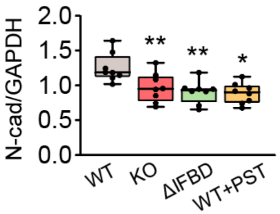

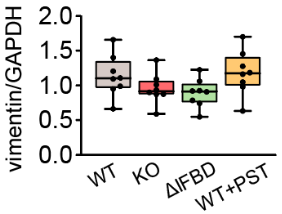

replicate 1-4 (240126)

Ponceau

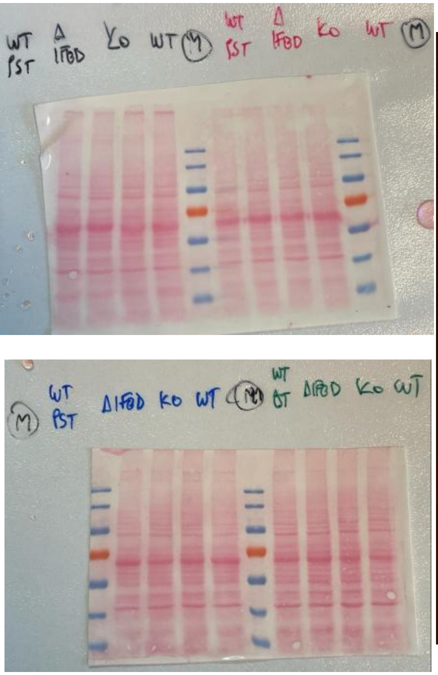

Raw merged Odyssey

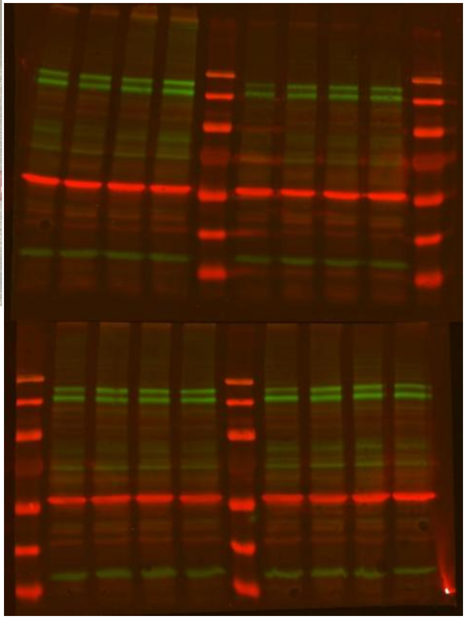

One channel (700) Odyssey

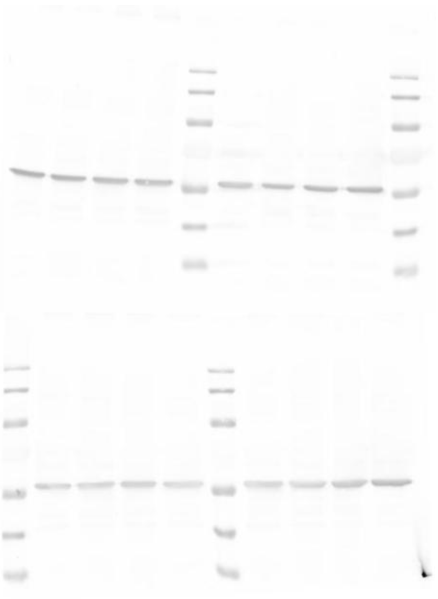

One channel (800) Odyssey

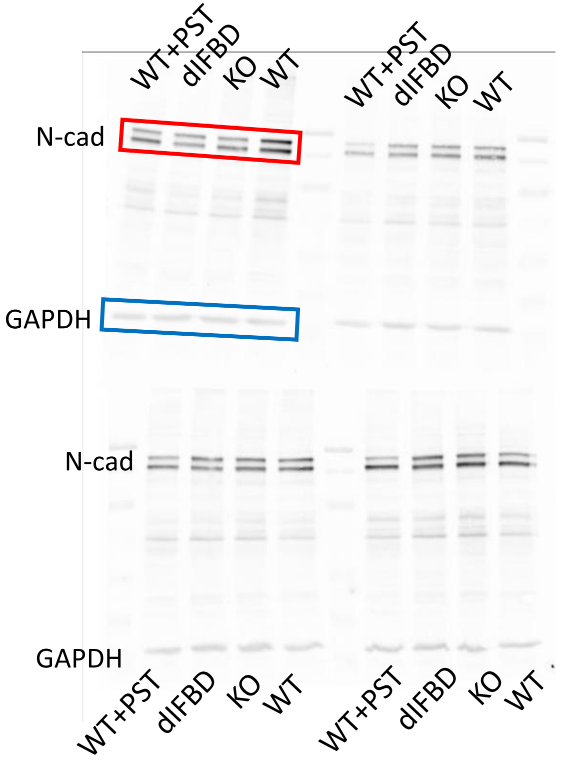

Ponceau

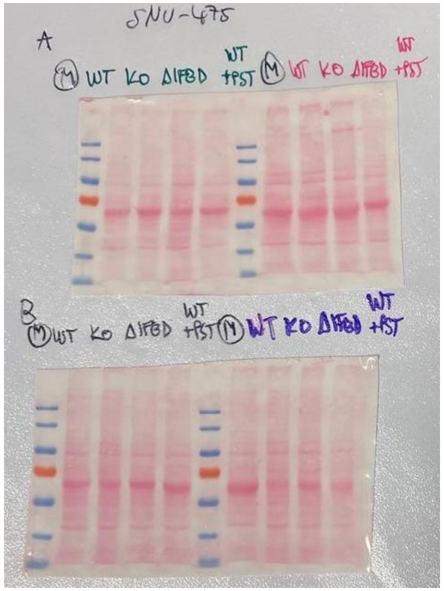

replicate 5-8 (240201)

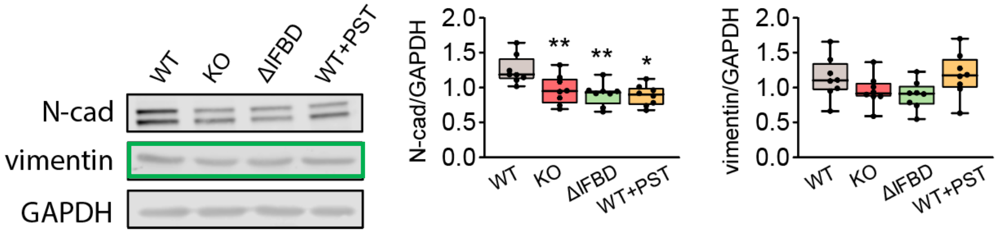

Raw merged Odyssey

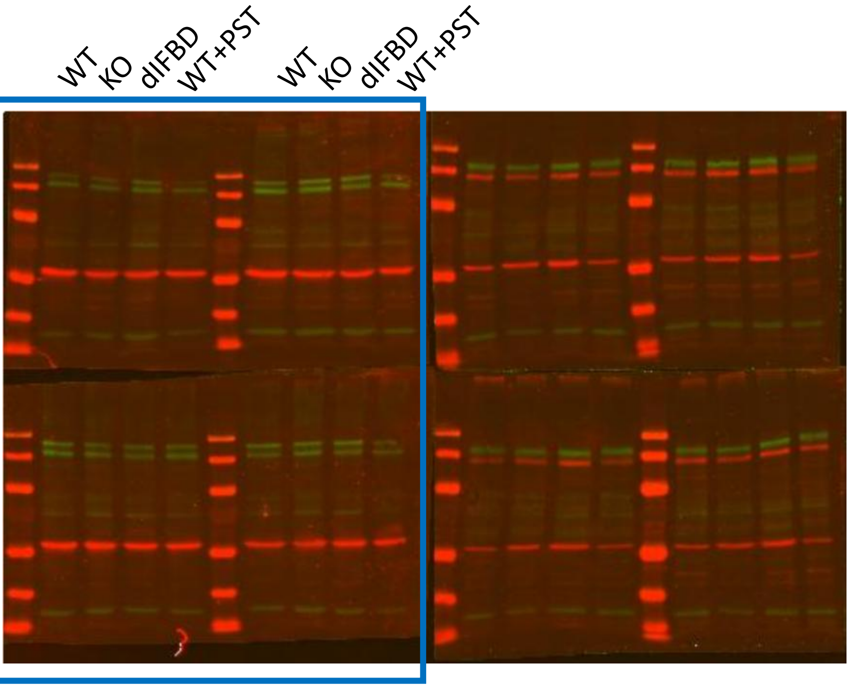

One channel (700) Odyssey

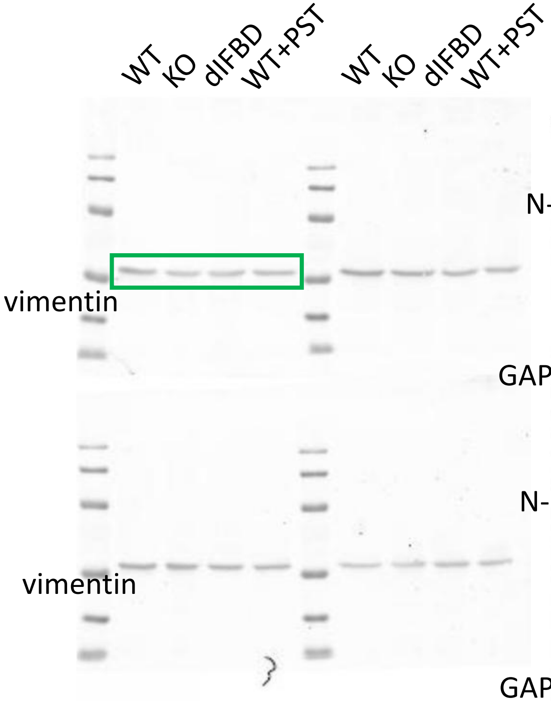

One channel (800) Odyssey

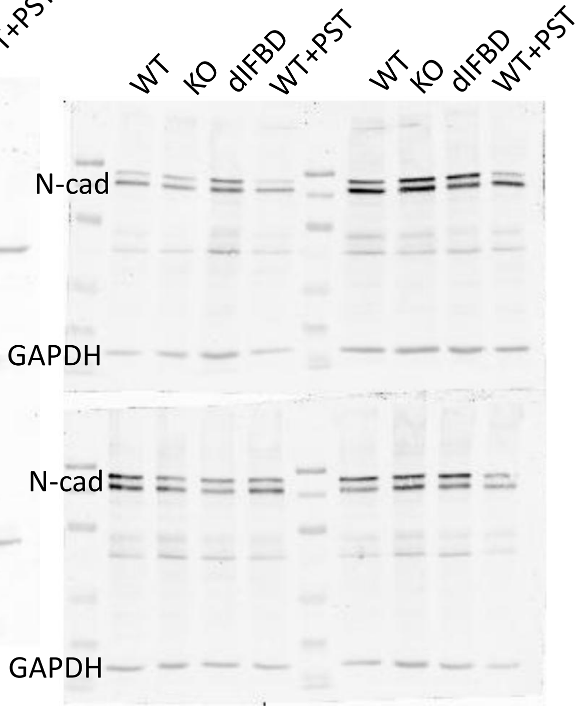

Supplement: Figure 5—figure supplement 1—source data 1. [file elife-102205-fig5-figsupp1-data1.pdf]
