# Supplementary material for: Plectin-mediated cytoskeletal crosstalk as a target for inhibition of hepatocellular carcinoma growth and metastasis
Source: eLife. 2025 Mar 7;13:RP102205. doi: 10.7554/eLife.102205 (PMC11893104; doi:10.7554/eLife.102205)
Supplement: Supplementary file 1. [file elife-102205-supp1.docx]

| **Sample ID** | **Etiology** 0 = unknown 1 = ALD/ASH 2 = NAFLD/NASH 3 = HBV 4 = HCV 5 = Other | **Age** [years] | **Sex** 0 = male 1 = female |
| --- | --- | --- | --- |
| MAP_1_HCC | 4 | 78.5 | 1 |
| MAP_10_HCC | 4 | 51.4 | 0 |
| MAP_11_HCC | 2 | 79.0 | 0 |
| MAP_12_HCC | 1 | 86.2 | 0 |
| MAP_13_HCC | 1 | 69.2 | 0 |
| MAP_14_HCC | 3 + 4 | 49.5 | 0 |
| MAP_15_HCC | 1 | 83.9 | 0 |
| MAP_16_HCC | 4 | 59.2 | 1 |
| MAP_17_HCC | 4 | 54.8 | 0 |
| MAP_18_HCC | 1 + 4 | 68.4 | 0 |
| MAP_19_HCC | 1 + 4 | 56.5 | 1 |
| MAP_2_HCC | 2 + 5 | 61.8 | 1 |
| MAP_20_HCC | 1 + 4 | 48.2 | 0 |
| MAP_21_HCC | 2 + 5 | 63.5 | 1 |
| MAP_3_HCC | 1 | 67.5 | 0 |
| MAP_4_HCC | 1 | 71.9 | 0 |
| MAP_5_HCC | 0 | 66.1 | 0 |
| MAP_6_HCC | 1 | 83.0 | 0 |
| MAP_7_HCC | 0 | 63.3 | 1 |
| MAP_8_HCC | 2 | 68.6 | 0 |
| MAP_9_HCC | 3 | 57.9 | 0 |

**Supplementary file 1**. Table of patients’ clinical data.
