# Supplementary material for: Plectin-mediated cytoskeletal crosstalk as a target for inhibition of hepatocellular carcinoma growth and metastasis
Source: eLife. 2025 Mar 7;13:RP102205. doi: 10.7554/eLife.102205 (PMC11893104; doi:10.7554/eLife.102205)
Supplement: Supplementary file 2. [file elife-102205-supp2.docx]

| **Primary antibodies** | | | | | |
| --- | --- | --- | --- | --- | --- |
| **Antigen** | **Clone** | **Manufacturer** | **Cat. number** | **Application** | **Dilution** |
| rabbit anti-Akt | polyclonal | Cell Signaling | 9272 | WB | 1:1000 |
| rabbit anti-pAkt (Ser473) | D9E | Cell Signaling | 4060 | WB | 1:2000 |
| mouse anti-E-cadherin | 36/E | BD Bioscience | 610181 | WB | 1:4000 |
| mouse anti-Erk2 | D-2 | Santa Cruz | sc-1674 | WB | 1:200 |
| rabbit anti-pErk1/2  (Thr202/Tyr204) | D13.14.4E | Cell Signaling | 4370 | WB | 1:1000 |
| mouse anti-FAK | 77/FAK Ruo | BD Bioscience | 610088 | WB | 1:500 |
| rabbit anti p-FAK (Tyr397) | polyclonal | Thermo-Fisher | 44-624G | WB | 1:1000 |
| rabbit anti-GAPDH | FF26A | Sigma | G9545 | WB | 1:20.000 |
| rabbit anti-GFP | polyclonal | Invitrogen | A-11122 | immunofluorescence | 1:100 |
| rabbit anti-ILK | 4G9 | Cell Signaling | 3856 | WB | 1:1000 |
| rabbit anti- αV integrin | EPR16800 | Abcam | ab179475 | WB | 1:5000 |
| mouse anti-β1 integrin | A-4 | Santa Cruz | sc-374429 | WB | 1:500 |
| rabbit anti-Ki67 | SP6 | GeneTex | GTX16667 | immunofluorescence | 1:100 |
| rabbit anti-N-cadherin | polyclonal | Abcam | ab18203 | WB | 1:1000 |
| mouse anti-paxillin | 349 | BD Bioscience | 612405 | WB | 1:1000 |
| rabbit anti-PI3K p85 | 19H8 | Cell Signaling | 4257 | WB | 1:1000 |
| rabbit anti-pPI3K p85/p55 (Tyr458/Tyr199) | polyclonal | Cell Signaling | 4228 | WB | 1:1000 |
| guinea pig anti-plectin | polyclonal | Progen | GP21 | WB | 1:1000 |
|  |  |  |  | immunofluorescence | 1:250 |
| mouse anti-talin | 8d4 | Sigma | T3287 | WB | 1:1000 |
| mouse anti-vimentin | RV202 | Santa Cruz | sc-32322 | WB | 1:500 |
| rabbit anti-vimentin | polyclonal | GeneTex | GTX100619 | immunofluorescence | 1:200 |
| mouse anti-vinculin | VIN-11-5 | Sigma | V4505 | WB | 1:500 |
|  |  |  |  | immunofluorescence | 1:250 |
| **Secondary antibodies** | | | | | |
| **Name** | **Manufacturer** |  | **Cat. number** | **Application** | **Dilution** |
| anti-mouse AF-488 | Jackson ImmunoResearch | | 715-545-150 | immunofluorescence | 1:500 |
| anti-rabbit AF-488 | Jackson ImmunoResearch | | 711-545-152 | immunofluorescence | 1:500 |
| anti-rabbit AF-594 | Life Tech | | A11037 | immunofluorescence | 1:1000 |
| anti-rabbit AF-647 | Jackson ImmunoResearch | |  | immunofluorescence | 1:500 |
| anti-guinea pig AF-488 | Jackson ImmunoResearch | | 706-545-148 | immunofluorescence | 1:500 |
| anti-mouse IgG IRDye® 680RD | Licor | | 926-68072 | WB | 1:20,000 |
| anti-mouse IRDye® 800CW | Licor | | 926-32212 | WB | 1:20,000 |
| anti-rabbit IRDye® 680RD | Licor | | 926-68073 | WB | 1:20,000 |
| anti-rabbit IgG IRDye® 800CW | Licor | | 926-32213 | WB | 1:20,000 |
| anti-guinea pig IRDye® 680RD | Licor | | 926-68077 | WB | 1:20,000 |
| anti-mouse-IgG-HRP | Jackson ImmunoResearch | | 115-035-146 | WB | 1:5000 |
| anti-rabbit-IgG-HRP | Jackson ImmunoResearch | | 111-035-044 | WB | 1:5000 |
| anti-GuineaPig-IgG-HRP | Sigma | | A7289 | WB | 1:5000 |

**Supplementary File 2.** List of antibodies used in this study.
